# The long-term spatio-temporal trends in burden and attributable risk factors of major depressive disorder at global, regional and national levels during 1990–2019: a systematic analysis for GBD 2019

**DOI:** 10.1017/S2045796024000295

**Published:** 2024-05-20

**Authors:** Zhi-Yang Mo, Ze-Zhen Qin, Jun-Jie Ye, Xin-Xuan Hu, Rui Wang, Ya-Ye Zhao, Ping Zheng, Qiao-Shan Lu, Qiao Li, Xian-Yan Tang

**Affiliations:** Department of Epidemiology and Biostatistics, School of Public Health, Guangxi Medical University, Nanning, Guangxi Zhuang Autonomous Region, China

**Keywords:** age-period-cohort study, bullying victimization, childhood sexual abuse, global burden of disease, intimate partner violence, joinpoint regression analysis, major depressive disorder, systematic analysis

## Abstract

**Aims:**

Caused by multiple risk factors, heavy burden of major depressive disorder (MDD) poses serious challenges to public health worldwide over the past 30 years. Yet the burden and attributable risk factors of MDD were not systematically known. We aimed to reveal the long-term spatio-temporal trends in the burden and attributable risk factors of MDD at global, regional and national levels during 1990–2019.

**Methods:**

We obtained MDD and attributable risk factors data from Global Burden of Disease Study 2019. We used joinpoint regression model to assess the temporal trend in MDD burden, and age–period–cohort model to measure the effects of age, period and birth cohort on MDD incidence rate. We utilized population attributable fractions (PAFs) to estimate the specific proportions of MDD burden attributed to given risk factors.

**Results:**

During 1990–2019, the global number of MDD incident cases, prevalent cases and disability-adjusted life years (DALYs) increased by 59.10%, 59.57% and 58.57%, respectively. Whereas the global age-standardized incidence rate (ASIR), age-standardized prevalence rate (ASPR) and age-standardized DALYs rate (ASDR) of MDD decreased during 1990–2019. The ASIR, ASPR and ASDR in women were 1.62, 1.62 and 1.60 times as that in men in 2019, respectively. The highest age-specific incidence, prevalence and DALYs rate occurred at the age of 60–64 in women, and at the age of 75–84 in men, but the maximum increasing trends in these age-specific rates occurred at the age of 5–9. Population living during 2000–2004 had higher risk of MDD. MDD burden varied by socio-demographic index (SDI), regions and nations. In 2019, low-SDI region, Central sub-Saharan Africa and Uganda had the highest ASIR, ASPR and ASDR. The global PAFs of intimate partner violence (IPV), childhood sexual abuse (CSA) and bullying victimization (BV) were 8.43%, 5.46% and 4.86% in 2019, respectively.

**Conclusions:**

Over the past 30 years, the global ASIR, ASPR and ASDR of MDD had decreased trends, while the burden of MDD was still serious, and multiple disparities in MDD burden remarkably existed. Women, elderly and populations living during 2000–2004 and in low-SDI regions, had more severe burden of MDD. Children were more susceptible to MDD. Up to 18.75% of global MDD burden would be eliminated through early preventing against IPV, CSA and BV. Tailored strategies-and-measures in different regions and demographic groups based on findings in this studywould be urgently needed to eliminate the impacts of modifiable risk factors on MDD, and then mitigate the burden of MDD.

## Introduction

Depression is a common mental disorder, and it is a leading cause of disability and contributes significantly to the global burden of disease (GBD 2019 Mental Disorders Collaborators, 2022). Major depressive disorder (MDD) is one kind of depression. MDD is characterized by depressed mood, or the loss of interest or pleasure in almost all activities for at least 2 weeks. MDD is significantly associated with a wide variety of chronic disorders, including cardiovascular disease, cancer and diabetes (Chapman *et al.*, 2004). According to prediction of the World Health Organization (WHO), MDD is expected to be the second-leading cause of disease burden worldwide by 2030 (Mathers and Loncar, 2006).

Multiple risk factors contribute to the burden of MDD. For MDD patients, the incidence could be affected by age effect, period effect and birth cohort effect. The incidence of MDD varies not only by ages but also by the living eras and birth cohorts experienced different social, political and economic situations (He *et al.*, 2021). Adversity from society and family in early childhood would have adverse impacts on mental health that may last a lifetime (Xie *et al.*, 2018). Health policies would affect all individuals, regardless of age and birth cohort in a certain period. For instance, during the coronavirus disease (COVID-19) pandemic, measures such as social restriction and lockdown had substantially affected the mental health of residents (COVID-19 Mental Disorders Collaborators, 2021). In addition, modifiable risk factors including life stressors and experiences would affect the incidence of MDD (Ettman *et al.*, 2020). As pervasive stressors, intimate partner violence (IPV), childhood sexual abuse (CSA) and bullying victimization (BV) were reported to be significantly associated with mental disorders, particularly increasing the risk of MDD (Beydoun *et al.*, 2012; Jadambaa *et al.*, 2019; Maniglio, 2010). Thus, it would be public health significance to control modifiable risk factors of MDD among vulnerable population.

The Global Burden of Diseases Study (GBD) are systematical studies on the global burden of diseases and risk factors. Based on the GBD database, previous studies mainly focused on regional disparity in MDD burden and gender disparity in MDD burden (Li *et al.*, 2022; Liu *et al.*, 2020), yet few studies systematically explored the long-term trends in MDD burden at global, regional and national levels. Moreover, there was a paucity of studies on the proportions of MDD burden attributed to modifiable risk factors (e.g., IPV, CSA and BV), though one study reported the burden of MDD caused by BV (Hong *et al.*, 2022).

To address above issues, it is necessary to understand the long-term trends in MDD burden worldwide, and measure the proportions of MDD burden attributed to primary and modifiable risk factors. Thus, based on data from the GBD 2019, we aimed to reveal the long-term spatio-temporal trends in incidence, prevalence and disability-adjusted life years (DALYs) of MDD at global, regional and national levels during 1990–2019, assess the proportions of MDD burden attributed to primary and modifiable risk factors including IPV, CSA and BV, understand the effects of age, period and birth cohort on MDD incidence over the past 30 years, and further identify the disparities in MDD burden regarding gender, age group, period, birth cohort and regions. Findings would update evidences for global mental health policies in mitigating the burden of MDD and controlling modifiable risk factors, and developing strategies-and-measures tailored to different regions and countries.

## Methods

### Data sources

We obtained data from the GBD 2019 (http://ghdx.healthdata.org/gbd-results-tool). For the assessment of GBD 2019, MDD was coded as F32.0–9 and F33.0–9 in category of ICD-10, and 296.21–24 and 296.31–34 in category of DSM-IV-TR, respectively. We obtained annual incident cases, age-standardized incidence rate (ASIR), prevalent cases, age-standardized prevalence rate (ASPR), DALYs, age-standardized DALYs rate (ASDR), corresponding percentage change and corresponding 95% uncertainty intervals (UIs) by gender, regions and countries during 1990–2019. In the GBD analysis, only IPV, CSA and BV were viewed as the risk factors of MDD among the identified 87 risk factors (GBD 2019 Risk Factors Collaborators, 2020). The attributable proportions of MDD age-standardized DALYs due to IPV, CSA and BV were measured by population attributable fractions (PAFs). PAFs were calculated by a comparative risk assessment framework in the GBD 2019. DALYs are the sum of years of life lived with disability and years of life lost due to premature death. Countries and territories were categorized by the socio-demographic index (SDI) into five categories, i.e., high, high-middle, middle, low-middle and low SDI. In addition, 204 countries and territories were classified into 21 GBD regions based on the socioeconomic similarity and geographical proximity to each other. Details on SDI regions and GBD regions were presented in the supplementary materials (Supplemental Tables S1 and S2). A total of 20 age groups were defined, i.e., 0–4 years, 5–9 years, …, and ≥ 95 years. More details on methodologies of GBD were described elsewhere (GBD 2019 Risk Factors Collaborators, 2020).

### Measurements

In GBD, IPV was defined as any lifetime experience of physical or sexual violence in women, perpetrated by the current or former intimate partner. CSA was defined as exposure before the age of 15 years to unwanted sexual contact by any perpetrator, or sexual contact by a perpetrator aged at least 5 years older than the victim. BV was defined as the intentional and repeated harm on children and adolescents by peers during periods of attending school. BV incorporated all forms of bullying, but excluded abuse by siblings, intimate partners and adults.

### Statistical analysis

Differences in age structure may lead to heterogeneity of MDD burden, including incidence, prevalence and DALYs. To adjust for the effect of age structure differences, we used age-standardized rates (ASRs) to estimate MDD burden, including ASIR, ASPR and ASDR. We used joinpoint regression analysis model to examine the temporal trends in ASIR, ASPR and ASDR of MDD for the whole period 1990–2019. This analysis was performed in Joinpoint Regression Program version 4.9.1, developed by the National Cancer Institute (United States). We transformed the long-term temporal trends into serial and meaningful segments via joinpoint regression analysis model, and estimate the annual percentage change (APC), the average annual percentage change (AAPC) and corresponding 95% confidence interval (CI) for the given segments (Kim *et al.*, 2000). When describing the temporal trend in ASIR, ASPR and ASDR of MDD, increasing trend was defined as APC or AAPC > 0 and *P* value <0.05, and decreasing trend was defined as APC or AAPC < 0 and *P* value <0.05, and stable trend was defined as the 95% CI containing 0 and *P* value ≥0.05.

We used age–period–cohort model to assess the age effect, period effect and birth cohort effect on incidence of MDD. This model quantitatively assessed the independent effects of age, period and birth cohort on the trends in the incidence of MDD over time. We conducted age–period–cohort analysis via the freely available age–period–cohort Web Tool (http://analysistools.nci.nih.gov/apc/ (Rosenberg *et al.*, 2014). In this model, the net drift is an estimate of overall temporal trend in incidence rates, expressed as the APC of incidence rates. The local drift indicates the temporal trend of incidence rates within each age group, expressed as the APC of age-specific incidence rates. The longitudinal age curve shows expected age-specific rates in the reference cohort adjusted for period effect. The period (or cohort) rate ratio (RR) refers to the ratio of age-specific rate in the given period (or cohort) to that in the reference period (or cohort). To conduct age–period–cohort analysis, age intervals should be equal to period intervals. We divided the population into consecutive 5-year age groups (i.e., 0–4, 5–9, …, and 89–94) and successive 5-year periods (i.e., 1990–1994, …, and 2015–2019). Totally, we had 24 consecutive cohorts to cover all individuals born from 1900–1904 to 2015–2019. The period of 2000–2004 and the birth cohort of 1955–1959 were defined as the reference groups, respectively. We used Wald chi-square test to test the significance of the estimable parameters. All *P* values were two-sided, with a significance level of 0.05.

## Results

### Long-term spatio-temporal trends in MDD burden

At global level, the estimated number of MDD incident cases, prevalent cases and DALYs increased by 59.10%, 59.57% and 58.57% during 1990–2019, respectively (Table 1, and Supplemental Tables S6 and S7). Whereas the corresponding ASIR, ASPR and ASDR had decreased trends, with AAPC −0.11 (95% CI: −0.13, −0.08) in ASIR, AAPC −0.12 (95% CI: −0.14, −0.09) in ASPR and AAPC −0.11 (95% CI: −0.14, −0.08) in ASDR (Table 2). Specifically, the global ASRs experienced a large upward trend during 1990–1994, a slight downward trend with high level during 1994–2001, a stable trend with high level during 2001–2005, a large downward trend during 2005–2010 and a slight upward trend during 2010–2019 (Table 2, and Supplemental Figures S8–S10).

**Table 1.** The number and ASIR of MDD in 1990 and 2019 by gender, SDI regions and GBD regions, and their percentage change

	Number × 10^6^ (95% UI)		Percentage change of number (%, 95% UI)	ASIR per 1000 population (95% UI)		Percentage change of ASIR (%, 95% UI)
Characteristics	In 1990	In 2019	During 1990−2019	In 1990	In 2019	During 1990−2019
**Global**	172.72 (197.44, 150.25)	274.80 (312.77, 241.28)	59.10 (63.65, 54.38)	3.49 (3.08, 3.95)	3.40 (2.98, 3.87)	−2.78 (−4.01, −1.65)
**Gender**						
Men	63.18 (72.3, 54.92)	103.72 (118.04, 90.68)	64.16 (68.65, 59.50)	2.59 (2.93, 2.27)	2.59 (2.94, 2.27)	0.48 (1.45, −0.63)
Women	109.54 (125.05, 95.72)	171.09 (195.23, 150.21)	56.19 (61.14, 51.38)	4.40 (4.98, 3.87)	4.19 (4.78, 3.67)	−4.62 (−3.22, −6.12)
**SDI regions**						
High SDI	31.06 (34.72, 27.68)	42.84 (48.28, 37.99)	37.92 (41.41, 33.99)	3.47 (3.09, 3.88)	3.84 (3.37, 4.38)	10.72 (7.81, 13.60)
High-middle SDI	38.45 (43.47, 33.87)	50.60 (57.32, 44.56)	31.40 (36.37, 26.27)	3.30 (2.91, 3.71)	3.00 (2.65, 3.40)	−9.23 (−11.05, −7.41)
Middle SDI	46.04 (53.12, 39.66)	75.72 (86.27, 66.30)	61.18 (69.53, 52.33)	2.97 (2.59, 3.37)	2.95 (2.58, 3.35)	−2.63 (−4.39, −0.96)
Low-middle SDI	38.99 (45.29, 33.41)	66.75 (76.41, 57.86)	69.70 (75.31, 63.93)	4.33 (3.76, 4.96)	3.99 (3.48, 4.54)	−8.69 (−10.87, −6.79)
Low SDI	18.08 (21.16, 15.42)	38.72 (45.21, 32.96)	107.13 (110.00, 104.23)	4.83 (4.18, 5.56)	4.56 (3.95, 5.25)	−8.81 (−10.00, −7.52)
**GBD regions**						
Central Asia	1.94 (2.24, 1.67)	2.81 (3.29, 2.42)	44.74 (51.25, 38.25)	3.32 (2.87, 3.80)	3.15 (2.71, 3.64)	−5.03 (−8.59, −1.17)
Central Europe	3.46 (3.94, 3.03)	3.33 (3.79, 2.90)	−3.98 (0.69, −8.51)	2.58 (2.26, 2.94)	2.26 (1.96, 2.60)	−12.64 (−15.78, −9.55)
Eastern Europe	9.47 (10.85, 8.11)	8.72 (10.00, 7.50)	−7.93 (−5.53, −10.15)	3.76 (3.22, 4.32)	3.36 (2.89, 3.89)	−10.41 (−12.03, −8.64)
Australasia	1.04 (1.19, 0.92)	1.49 (1.72, 1.28)	42.40 (53.86, 31.55)	4.86 (4.26, 5.53)	4.91 (4.20, 5.76)	1.05 (−6.37, 9.11)
High-income Asia Pacific	4.01 (4.52, 3.55)	4.96 (5.51, 4.42)	23.68 (30.05, 17.56)	2.10 (1.86, 2.36)	2.21 (1.96, 2.49)	5.27 (2.94, 7.84)
High-income North America	10.72 (12.14, 9.41)	17.72 (19.93, 15.70)	65.35 (69.76, 61.48)	3.59 (3.16, 4.09)	4.69 (4.11, 5.34)	30.80 (27.76, 33.90)
Southern Latin America	1.71 (1.94, 1.50)	2.28 (2.58, 2.01)	33.36 (39.83, 27.31)	3.52 (3.11, 3.99)	3.20 (2.81, 3.62)	−9.28 (−13.57, −5.03)
Western Europe	19.00 (21.05, 17.09)	21.51 (24.19, 19.08)	13.18 (17.61, 8.46)	4.36 (3.92, 4.84)	4.18 (3.68, 4.74)	−4.31 (−7.79, −0.49)
Andean Latin America	0.92 (1.09, 0.78)	1.71 (1.99, 1.47)	86.54 (98.02, 74.82)	2.91 (2.51, 3.40)	2.73 (2.35, 3.16)	−6.10 (−10.29, −2.11)
Caribbean	1.55 (1.80, 1.32)	2.08 (2.41, 1.78)	34.41 (42.68, 25.90)	4.73 (4.07, 5.47)	4.18 (3.59, 4.84)	−11.51 (−15.84, −7.44)
Central Latin America	4.24 (4.91, 3.63)	9.03 (10.33, 7.85)	112.82 (122.38, 103.57)	3.20 (2.80, 3.67)	3.53 (3.08, 4.04)	10.13 (8.01, 12.18)
Tropical Latin America	6.46 (7.42, 5.59)	10.56 (11.76, 9.39)	63.37 (71.72, 54.62)	4.72 (4.12, 5.35)	4.41 (3.93, 4.91)	−6.62 (−10.43, −2.19)
North Africa and Middle East	14.06 (16.56, 11.94)	29.92 (35.26, 25.28)	112.76 (123.60, 102.47)	4.89 (4.21, 5.66)	4.92 (4.20, 5.77)	0.61 (−1.59, 2.83)
South Asia	38.63 (44.77, 33.22)	68.51 (78.16, 59.56)	77.35 (83.63, 70.53)	4.47 (3.88, 5.12)	3.99 (3.47, 4.54)	−10.72 (−13.16, −8.51)
East Asia	29.72 (34.17, 25.52)	38.63 (43.62, 34.01)	29.97 (43.20, 17.62)	2.43 (2.12, 2.76)	2.09 (1.85, 2.36)	−13.81 (−17.65, −9.63)
Oceania	0.15 (0.18, 0.12)	0.30 (0.37, 0.25)	106.66 (115.46, 96.78)	2.61 (2.19, 3.09)	2.51 (2.12, 2.99)	−3.79 (−7.41, −0.31)
Southeast Asia	8.12 (9.55, 6.88)	12.99 (14.98, 11.13)	60.07 (68.40, 52.04)	1.95 (1.68, 2.24)	1.86 (1.60, 2.14)	−4.66 (−6.48, −2.41)
Central sub-Saharan Africa	2.70 (3.26, 2.23)	6.48 (7.84, 5.37)	139.57 (151.04, 128.75)	6.68 (5.68, 7.86)	6.44 (5.46, 7.63)	−3.68 (−7.83, 0.38)
Eastern sub-Saharan Africa	7.02 (8.18, 5.99)	15.20 (17.75, 12.92)	116.64 (120.71, 112.43)	5.59 (4.87, 6.42)	5.24 (4.56, 5.99)	−6.33 (−8.06, −4.64)
Southern sub-Saharan Africa	1.79 (2.05, 1.54)	3.18 (3.62, 2.75)	77.30 (83.50, 70.73)	4.29 (3.75, 4.87)	4.34 (3.80, 4.90)	1.12 (−1.00, 3.48)
Western sub-Saharan Africa	6.00 (6.96, 5.13)	13.41 (15.60, 11.44)	123.43 (126.38, 120.39)	4.48 (3.88, 5.15)	4.20 (3.64, 4.82)	−6.36 (−7.63, −4.99)

ASIR, age-standardized incidence rate; MDD, major depressive disorder; SDI, socio-demographic index; GBD, Global Burden of Disease Study; UI, uncertainty interval.

**Table 2.** The global annual per cent changes and average annual per cent changes of ASIR, ASPR and ASDR of MDD during 1990–2019

	ASIR			ASPR			ASDR		
Characteristics	Period	APC (95% CI)	AAPC (95% CI)	Period	APC (95% CI)	AAPC (95% CI)	Period	APC (95% CI)	AAPC (95% CI)
**Segment**									
Trend 1	1990−1994	1.13 (1.04, 1.21)*	–	1990−1994	1.12 (1.04, 1.21)*	–	1990−1994	1.15 (1.06, 1.24)*	–
Trend 2	1994−2001	−0.14 (−0.18, −0.09)*	–	1994−2001	−0.15 (−0.20, −0.11)*	–	1994−2001	−0.15 (−0.19, −0.10)*	–
Trend 3	2001−2005	0.13 (−0.00, 0.26)	–	2001−2005	0.12 (−0.01, 0.25)	–	2001−2005	0.13 (−0.00, 0.27)	–
Trend 4	2005−2010	−1.70 (−1.78, −1.62)*	–	2005−2010	−1.71 (−1.79, −1.63)*	–	2005−2010	−1.70 (−1.79, −1.62)*	–
Trend 5	2010−2019	0.16 (0.14, 0.19)*	–	2010−2019	0.16 (0.13, 0.18)*	–	2010−2019	0.16 (0.13, 0.18)*	–
**AAPC**	1990−2019	–	−0.11 (−0.13, −0.08)*	1990−2019	–	−0.12 (−0.14, −0.09)*	1990 −2019	–	−0.11 (−0.14, −0.08)*

ASIR, age-standardized incidence rate; ASPR, age-standardized prevalence rate; ASDR, age-standardized DALYs rate; DALYs, disability-adjusted life-years; MDD, major depressive disorder; APC, annual percentage change; AAPC, average annual percentage change; CI, confidence interval;

* *P* < 0.05.

At regional level of SDI, middle-SDI region had the most MDD incident cases, prevalent cases and DALYs in 2019, and only high-SDI region had increasing trend in ASRs (Supplemental Table S5 and Figure S1). But the highest ASRs occurred in low-SDI region (Table 1, and Supplemental Tables S6 and S7). At regional level of GBD in 2019, South Asia had the most MDD cases and DALYs, while Central sub-Saharan Africa had the highest ASRs (Table 1, and Supplemental Tables S6 and S7). High-income North America had the maximum increase trend in ASRs during 1990–2019 (Supplemental Table S5 and Figure S1).

**Figure 1.**
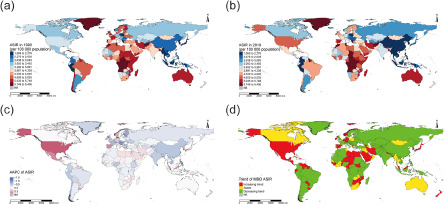
The ASIR of MDD across 204 countries and territories in 1990 (a) and in 2019 (b), the AAPC of ASIR during 1990–2019 (c), and the trend in ASIR during 1990–2019 (d). ASIR, age-standardized incidence rate; MDD, major depressive disorder; AAPC, average annual percentage change.

At national level, India ranked the first in the number of MDD cases and DALYs, followed by China and the United States of America in 2019 (Supplemental Tables S8–S10). Uganda, Palestine and Greenland ranked the top three in ASRs in 2019 (Fig. 1b, and Supplemental Figure S3). However, the United States of America had the largest increase in ASRs, followed by Mexico and Spain (Fig. 1c, and Supplemental Figure S3). Moreover, Malaysia, Uruguay, Republic of Korea and Germany kept a low level of ASRs but with a large upward trend (Fig. 1b, d and Supplemental Figure S3).

Regarding demographics, the ASIR, ASPR and ASDR of MDD in women were 1.62, 1.62 and 1.60 times as that in men in 2019, respectively (Table 1, and Supplemental Tables S6 and S7). In 2019, the highest age-specific incidence, prevalence and DALYs rate occurred at the age of 60–64 in women, and at the age of 75–84 in men (Supplemental Figure S2). Both in men and in women, the number of DALYs peaked at the age of 20–24, and the maximum increasing trends in age-specific incidence, prevalence and DALYs rates occurred at the age of 5–9 (Supplemental Figure S2).

### Effects of age, period and birth cohort on MDD burden

**Figure 2.**
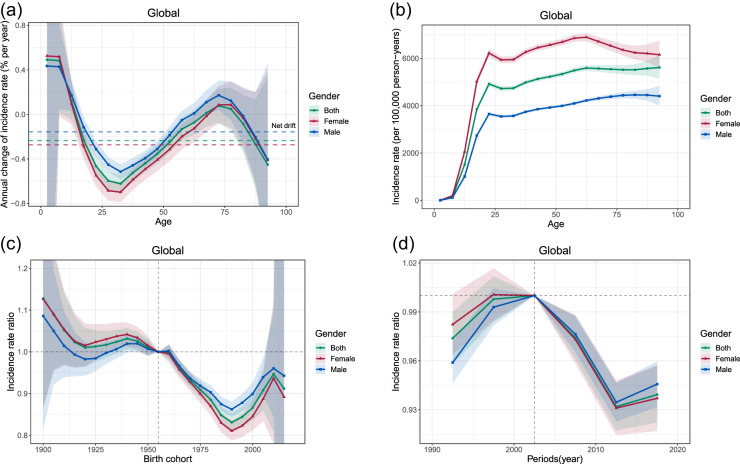
The parameters estimated by age–period–cohort model for the incidence rates of MDD by gender. Net drifts (horizontal lines) and local drifts (curves) (a), longitudinal age curves adjusted for period effects (b), cohort rate ratios relative to the reference cohort of 1955–1959 (c), and period rate ratios relative to the reference period of 2000–2005 (d).

Globally, a net drift of MDD incidence was −0.23% per year for the whole population, −0.27% for women and −0.16% for men (Supplemental Table S4). The local drifts were above zero for the ages of 0–14 and 65–79 (Fig. 2a). The incidence rates increased rapidly before the age of 20–24, and then maintained at high level (Fig. 2b). The period RR increased from 1990–1994 to a peak at 2000–2004, then decreased from 2005–2009 to 2010–2014 and increased again in 2015–2019 (Fig. 2d). Compared with the birth cohort of 1955–1959, population born before the birth cohort of 1955–1959 had higher risk of MDD (Fig. 2c).

### DALYs and ASDR of MDD attributed to IPV

**Figure 3.**
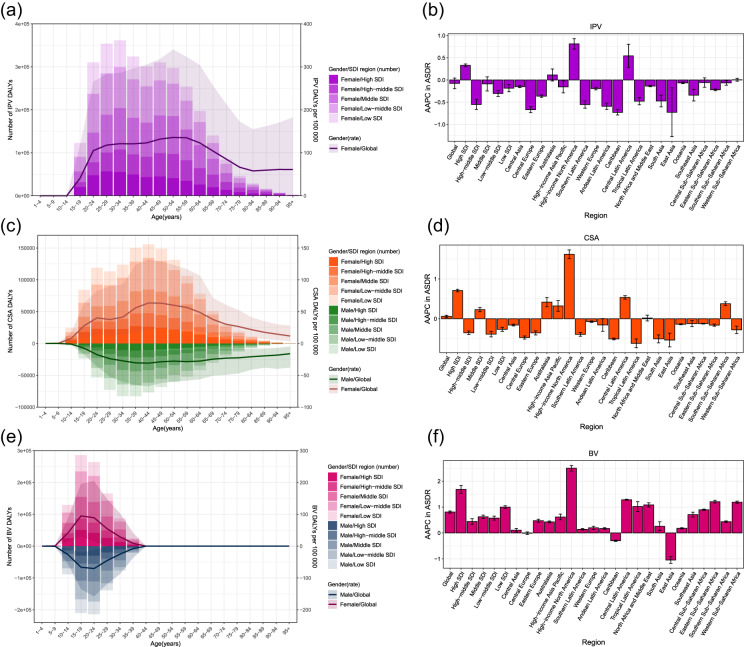
The age-specific numbers and DALYs rates of MDD attributed to IPV (a), CSA (c) and BV (e) in 2019. The AAPC of ASDR attributed to IPV (b), CSA (d) and BV (f) by SDI regions and GBD regions during 1990–2019. DALYs, disability-adjusted life years. MDD, major depressive disorder; IPV, intimate partner violence; CSA, childhood sexual abuse; BV, bullying victimization; SDI, socio-demographic index; GBD, Global Burden of Disease Study.

**Figure 4.**
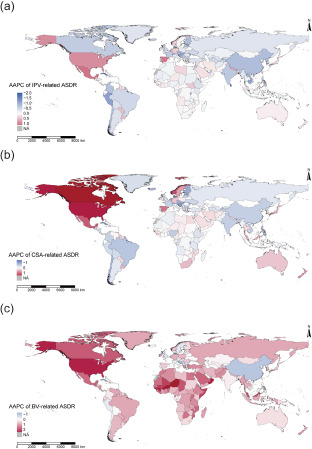
The AAPC of ASDR attributed to IPV (a), CSA (b) and BV (c) across 204 countries and territories during 1990–2019. AAPC, average annual percentage change. ASDR, age-standardized DALYs rate. IPV, intimate partner violence. CSA, childhood sexual abuse. BV, bullying victimization. DALYs, disability-adjusted life years.

At global level, the estimated number of IPV-related DALYs increased by 60.72% during 1990–2019, but IPV-related ASDR slightly decreased with a stable trend and AAPC of −0.07 (95% CI: −0.20, 0.04) (Supplemental Table S11, and Fig. 3b). An increasing trend in IPV-related DALYs rate occurred in population aged at 10–59 and 80–95+ in 2019 (Fig. 3a). At regional and national levels, low-SDI region, Central sub-Saharan Africa and Uganda had the highest IPV-related ASDR in 2019 (Supplemental Tables S11 and S15). Mexico had the maximum increasing trend in IPV-related ASDR, while Singapore had the maximum decreasing trend (Fig. 4a). Across the countries and territories of downward trend in ASDR, Guatemala, Bangladesh, Angola and American Samoa had an upward trend in IPV-related ASDR (Supplemental Tables S10 and S15).

### DALYs and ASDR of MDD attributed to CSA

At global level, the estimated number of CSA-related DALYs increased by 68.77% during 1990–2019, and the CSA-related ASDR had an elevated trend with AAPC of 0.06 (95% CI: 0.03, 0.09) (Supplemental Table S12, and Fig. 3d). An increasing trend in CSA-related DALYs rate occurred in women aged at 5–24 and 30–44, and men aged at 5–44 in 2019 (Fig. 3c). At regional and national levels, low-SDI region, Western sub-Saharan Africa and Gambia had the highest CSA-related ASDR in 2019 (Supplemental Tables S12 and S16). Canada had the maximum increasing trend in CSA-related ASDR, while Singapore had the maximum decreasing trend (Fig. 4b). Across the countries and territories of decreasing trend in ASDR, only Sweden had an increasing trend in CSA-related ASDR (Supplemental Tables S10 and S16).

### DALYs and ASDR of MDD attributed to BV

At global level, the estimated number of BV-related DALYs increased by 60.30% during 1990–2019, and the BV-related ASDR had an elevated trend with AAPC of 0.81 (95% CI: 0.77, 0.85) (Supplemental Table S13, and Fig. 3f). Both in men and in women, an increasing trend in BV-related DALYs rate occurred in population aged at 5–19 in 2019 (Fig. 3e). At regional and national levels, high-SDI region, High-income North America and Greenland had the highest ASDR in 2019 (Supplemental Tables S13 and S17). Niger had the maximum increasing trend in BV-related ASDR, while Cuba had the maximum decreasing trend (Fig. 4c). Across the countries and territories of decreasing trend in ASDR, Mali, Somalia, Equatorial Guinea, Burundi and Uganda had an increasing trend in BV-related ASDR (Supplemental Tables S10 and S17).

### PAFs of MDD attributed to IPV, CSA and BV

At the global level in 2019, the PAFs of IPV, CSA and BV were 8.43%, 5.46% and 4.86%, respectively (Supplemental Figure S6). At regional and national levels in 2019, low-SDI region, Southern sub-Saharan Africa and Liberia had the highest PAFs of IPV (Supplemental Table S15, and Figure S6). And low-SDI region, Western sub-Saharan Africa and New Zealand had the highest PAFs of CSA (Supplemental Table S16, and Figure S6). Moreover, high-SDI region, High-income North America and Greenland had the highest PAFs of BV (Supplemental Table S17, and Figure S6). At global level in 1990 and 2019, the PAFs of DALYs attributed to IPV, CSA and BV ranked the first, the second and the third, respectively (i.e., IPV > CSA > BV) (Supplemental Figure S6). At national level in 2019, the PAFs of DALYs ranked in order of IPV > BV >CSA for most countries and territories (Supplemental Figure S7).

## Discussion

The global ASIR, ASPR and ASDR of MDD decreased during 1990–2019, while the number of MDD cases and DALYs increased considerably. The burden of MDD in women was higher than that in men. Children had the maximum increasing trends in age-specific incidence, prevalence and DALYs rates of MDD, while these rates were the highest in elderly. Young adults had the largest number of MDD cases and DALYs. The global IPV-related ASDR kept a stable and high-level trend, while both CSA-related ASDR and BV-related ASDR increased over the past 30 years.

In the early 2000s, WHO initiated the World Mental Health Survey (WMHS), and it was a comprehensive initiative aimed at studying the prevalence and impact of mental disorders including MDD worldwide (Ustün, 1999). The WMHS has expanded its reach over time to provide the epidemiological parameters for MDD in many countries and has informed policy decisions to improve mental health services and reduce the burden of MDD worldwide (Ferrari *et al.*, 2013). We found that the global ASIR, ASPR and ASDR had the largest downward trend during 2005–2010. The contribution of the WMHS may partly explain the large decline in ASRs.

The estimated MDD burden varied across countries. The ASRs of MDD were the highest in Uganda, which has suffered from violent conflicts. A survey revealed that post-conflict northern Uganda still had high rates of MDD, and the relevant risk factors of MDD consisted of social-demographic factors, distal psycho-social vulnerability factors and the psycho-social stressors (Mugisha *et al.*, 2015). However, the United States of America had the maximum increasing trend in ASRs of MDD during 1990–2019. In a survey on adults in the United States, 12-month and lifetime prevalence of MDD were 10.4% and 20.6%, respectively, and MDD was associated with impaired functioning, low income and substance use disorders (Hasin *et al.*, 2018). In Malaysia, a survey revealed that the prevalence of mental health problems had an increasing trend among children at age of 5–15 (Sahril *et al.*, 2021). We also found that although the ASRs of MDD in Malaysia were lower than that in more than a half of countries and territories, the growth rate of ASRs was high. The situations of ASRs in Uruguay, the Republic of Korea and Germany were the same as that in Malaysia. At national level, the severity of MDD burden in this study differed from previous findings, that the highest ASIR was Lesotho rather than Uganda in GBD 2017 (Liu *et al.*, 2020). Future studies would be needed to further explore the possible reasons underlying these changes.

Previous studies revealed that prevalence of depression was approximately twice in women than that in men across different cultures (Bromet *et al.*, 2011). Coincidentally, our findings showed that the burden of MDD in women was higher than that in men for all age groups. The heritability of MDD was estimated at 30%–40%, and women had stronger genetic risk than that in men (Sullivan *et al.*, 2000). In addition, special life stages of women, such as pr-menstruation, pregnancy, postpartum and menopause, would be the biological factors of high vulnerability to MDD (Kuehner, 2017). Children and adolescents were more susceptible to MDD. Due to symptom variation from the adult criteria, MDD was often unrecognized and untreated in childhood and adolescence, and untreated MDD in children and adolescents may increase the risk of substance abuse and suicidal behaviours (Mullen, 2018). For young adults, the addiction of social media may be associated with the onset of MDD, since indulging in the internet could cause adverse outcomes including less health activities, sleep deprivation, social isolation and loneliness, and feelings of envy (Lin *et al.*, 2016). The risk of MDD varied over different life stages, and the high incidence rate in the elderly was associated with several physical diseases, disability and social determinants, including empty nest, widowhood, low education level, poverty and social exclusion (Abdoli *et al.*, 2022). Tailored strategies against MDD should be prioritized to vulnerable populations, particularly in women, children and the elderly.

Population having high risk of MDD were born in unrest and colonialism periods, when World War I and World War II outbroke. One meta-analysis estimated that around 354 million adult war survivors suffered from post-traumatic stress disorder and/or MDD (Hoppen and Morina, 2019). The influence of war on mental health was enduring. The National Vietnam Veterans Longitudinal Study indicated that approximately one-third of Vietnam veterans still suffer from MDD, 40 years or more late post the Vietnam war (Marmar *et al.*, 2015). Obviously, exposures to periods of social unrest increased the burden of mental disorders. Thus, future global mental health policies and intervention strategies should be tailored to these vulnerable populations.

Since social and environmental factors are often modifiable, targeting these modifiable factors should be prioritized to take public health actions against MDD. IPV is considered as gender-based violence. Although men are victim of MDD, IPV is more likely to affect women, due to the disparities in severity, frequency, type and lifetime impact of MDD (Berry and Monk, 2020). The World Conference on Women held in Beijing in 1995, marked a significant turning point for the global agenda of gender equality. Coincidentally, we found that the burden of MDD attributed to IPV began to decline substantially since 1995. Despite advances in international laws, standards and principles aimed at securing the equality of women has been achieved, violence against women happens widely across the world (Akhmedshina, 2020). This may lead to IPV-related MDD burden being continuously maintained at high level.

Despite the majority of MDD onset in adult life, most of relevant risk factors begin during childhood (Kessler *et al.*, 2010). In sub-Saharan Africa, most of childhood sexual violence was frequently perpetrated by similar age peers (Rumble *et al.*, 2015), and poverty and superstition may place children at heightened risk of CSA (McCrann *et al.*, 2006). According to the study in North American and Europe, over 10% of students worldwide reported experiencing bullying on campus at least 2–3 times per month (Lian *et al.*, 2018). The majority of bullied children suffered in silence, unwilling to share their experiences with parents or teachers out of fear of retaliation or shame (Wolke and Lereya, 2015). Unfortunately, BV has often been viewed as a natural part of the growing up process, rather than a major public health issue (Olweus, 2013). Therefore, future global mental health strategies should highlight these modifiable risk factors.

Identifying and eliminating IPV, CSA and BV should be prioritized to develop prevention measures against MDD. We found that Singapore had the largest downtrend of MDD burden during 1990–2019. The vulnerable populations were taken seriously in Singapore. For instance, during the COVID-19 lockdown, the Ministry of Social and Family Development of Singapore launched a 24 hours hotline against domestic violence towards vulnerable populations of IPV (O’Hara and Tan, 2022). Furthermore, in developing countries, most interventions for CSA focused on preschool and primary children to enhance their sexual safety knowledge and skills, but interventions should be expanded to communities in the future (Russell *et al.*, 2020). For the intervention of BV, whole-school programme integrating individual students, parents, class and schools to prevent bullying were successful in high-income countries (Menesini and Salmivalli, 2017). In a sense, future studies on mental disorders and modifiable risk factors should be strengthened to mitigate the burden.

There were several limitations in this study. First, in GBD 2019, due to the fact that the existing data sources on MDD were limited and incompletely available in some countries, the effect of health service interventions were unable to quantify, and the MDD burden in countries with little or none access to treatment for MDD may be underestimated (GBD 2019 Mental Disorders Collaborators, 2022). Second, since only three attributable risk factors (i.e., IPV, CSA and BV) of MDD could be obtained from GBD data sources, this study did not explore other risk factors of MDD. Third, the effects of age, period and birth cohort on MDD burden were explored. Yet this study was based on the estimated cross-sectional GBD data during 1990–2019, rather than a real-world cohort study.

## Conclusion

Over the past 30 years, the global ASIR, ASPR and ASDR of MDD all had decreased trends, while the burden of MDD was still serious. Women, elderly and populations living during 2000–2004 and in low-SDI region, suffered from more severe burden of MDD. Children were more susceptible to MDD. Up to 18.75% of the global MDD burden would be eliminated through early prevention against IPV, CSA and BV. These findings would facilitate policy makers and researchers to further understand the long-term spatio-temporal trends in global MDD burden, and the attributable impacts of modifiable risk factors (e.g., IPV, CSA and BV) on global MDD. Tailored strategies-and-measures in different regions and demographic groups based on findings in this study would be urgently needed to eliminate the impacts of modifiable risk factors on MDD, and then mitigate the burden of MDD.

## Supporting information

Mo et al. supplementary material Mo et al. supplementary material

## Data Availability

All data are openly available in the Global Health Data Exchange GBD Results Tool, http://ghdx.healthdata.org/gbd-results-tool.

## Supplementary material

The supplementary material for this article can be found at https://doi.org/10.1017/S2045796024000295.
