# Supplementary material for: The long-term spatio-temporal trends in burden and attributable risk factors of major depressive disorder at global, regional and national levels during 1990–2019: a systematic analysis for GBD 2019
Source: Epidemiol Psychiatr Sci. 2024 May 20;33:e28. doi: 10.1017/S2045796024000295 (PMC11362682; doi:10.1017/S2045796024000295)
Supplement: Mo et al. supplementary material [file S2045796024000295sup001.docx]

**Contents**

[Supplemental methods 2](#_Toc163396196)

[Abbreviation list 2](#_Toc163396197)

[Figure S1 3](#_Toc163396198)

[Figure S2 4](#_Toc163396199)

[Figure S3 5](#_Toc163396200)

[Figure S4 6](#_Toc163396201)

[Figure S5 7](#_Toc163396202)

[Figure S6 8](#_Toc163396203)

[Figure S7 9](#_Toc163396204)

[Figure S8 10](#_Toc163396205)

[Figure S9 11](#_Toc163396206)

[Figure S10 12](#_Toc163396207)

[Figure S11 13](#_Toc163396208)

[Figure S12 14](#_Toc163396209)

[Figure S13 15](#_Toc163396210)

[Table S1 16](#_Toc163396211)

[Table S2 24](#_Toc163396212)

[Table S3 32](#_Toc163396213)

[Table S4 42](#_Toc163396214)

[Table S5 43](#_Toc163396215)

[Table S6 45](#_Toc163396216)

[TableS7 48](#_Toc163396217)

[Table S8 51](#_Toc163396218)

[Table S9 68](#_Toc163396219)

[Table S10 85](#_Toc163396220)

[Table S11 102](#_Toc163396221)

[Table S12 106](#_Toc163396222)

[Table S13 110](#_Toc163396223)

[Table S14 114](#_Toc163396224)

[Table S15 130](#_Toc163396225)

[Table S16 154](#_Toc163396226)

[Table S17 178](#_Toc163396227)

Supplemental methods

**1 Measurements**

**1.1 Social**-**development index**

The social-development index (SDI) was developed by GBD researchers based on the total fertility rate under the age of 25, average education level for population aged at ≥15 years old, and lag distributed income per capital. SDI synthetically indicates the health development status. A region with an SDI of zero would represent a theoretical minimum level of development relevant to health, and a region with an SDI of one would represent a theoretical maximum level. High SDI regions are defined as SDI values between 0.805 and 1.000. High-middle SDI regions are defined as SDI values between 0.690 and 0.805. Middle SDI regions are defined as SDI values between 0.608 and 0.690. Low-middle SDI regions are defined as SDI values between 0.455 and 0.608. Low SDI regions are defined as SDI values between 0.000 and 0.455.

**1.2 Population attributable fractions**

Population attributable fractions (PAFs) are defined as when the exposure of a certain risk factor declined to the theoretical minimum exposure level in a certain population, the proportion of relevant diseases or deaths in the population would reduce.

Abbreviation list

| NO. | Abbreviation | Full term or phrase |
| --- | --- | --- |
| 1 | AAPC | average annual percentage change |
| 2 | APC | annual percentage change |
| 3 | ASDR | age-standardized DALYs rate |
| 4 | ASIR | age-standardized incidence rate |
| 5 | ASPR | age-standardized prevalence rate |
| 6 | ASR | age-standardized rate |
| 7 | BV | bullying victimization |
| 8 | CI | confidence interval |
| 9 | COVID-19 | coronavirus disease |
| 10 | CSA | childhood sexual abuse |
| 11 | DALY | disability-adjusted life year |
| 12 | GBD | global burden of disease study |
| 13 | IPV | intimate partner violence |
| 14 | MDD | major depressive disorder |
| 15 | PAF | population attributable fraction |
| 16 | RR | rate ratio |
| 17 | SDI | socio-demographic index |
| 18 | UI | uncertainty interval |
| 19 | WHO | World Health Organization |
| 20 | WMHS | World Mental Health Survey |


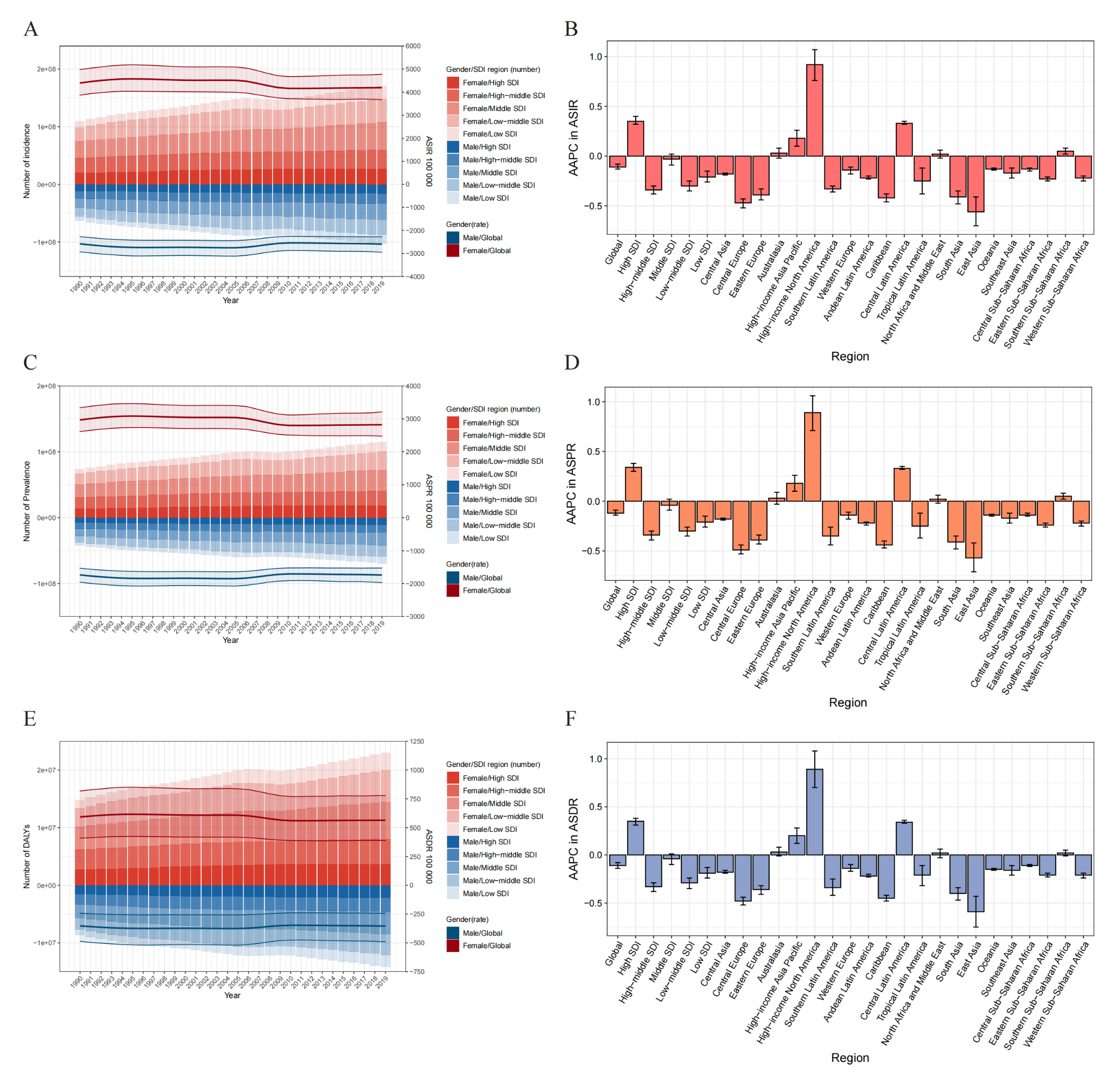


Figure S1 The number of MDD incident cases (A), prevalent cases (C), DALYs (E) and corresponding age-standardized rates during 1990-2019 by gender and SDI regions. The AAPC of ASIR (B), ASPR (D) and ASDR (F) by SDI regions and GBD regions (B).


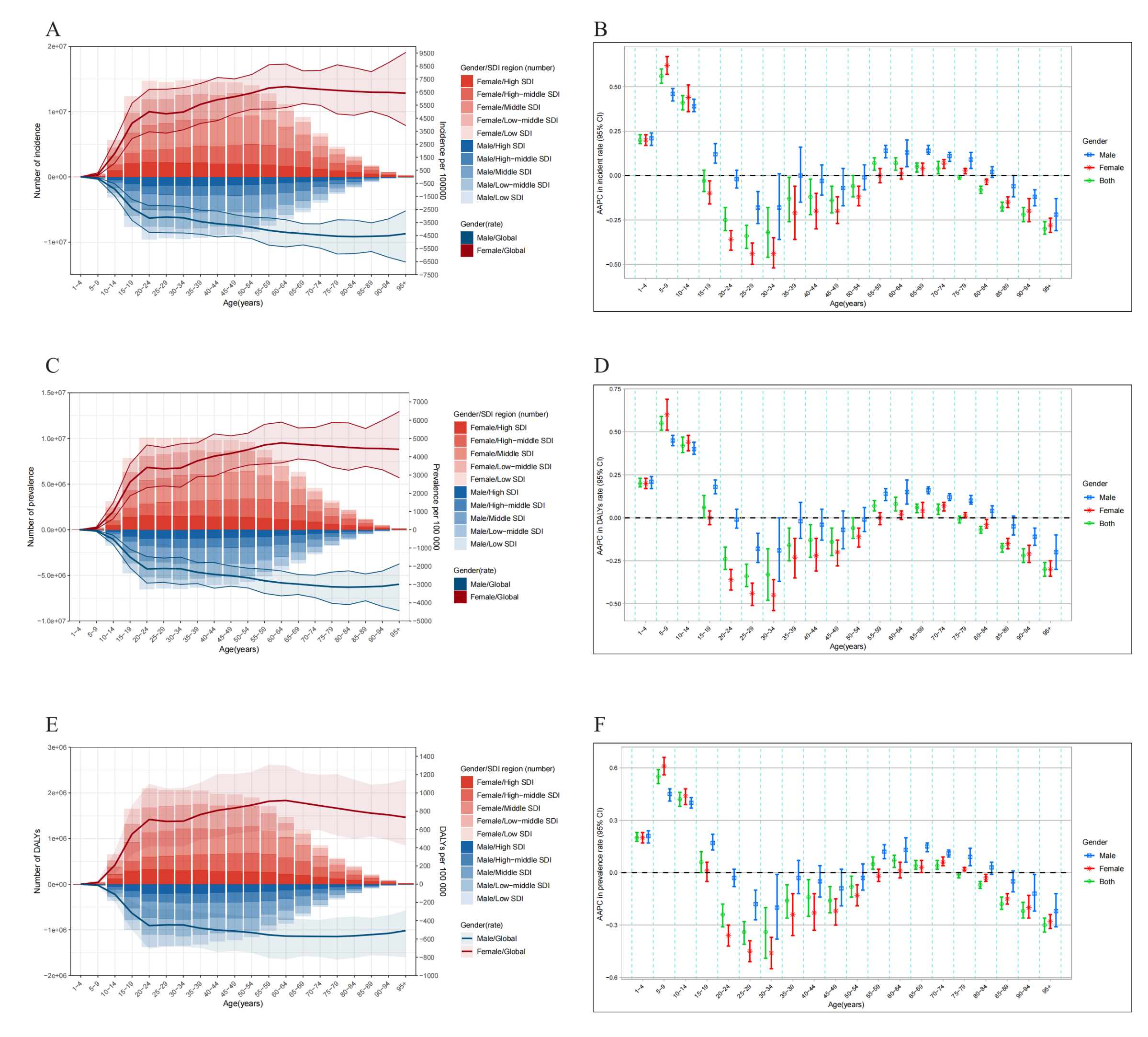


Figure S2 The age-specific number of MDD incident cases (A), prevalent cases (C), DALYs (E) and corresponding age-specific rates during 1990-2019 by gender and SDI regions. The age-specific AAPC of incidence rate (B), prevalence rate (D), and DALYs rate (F) by gender.


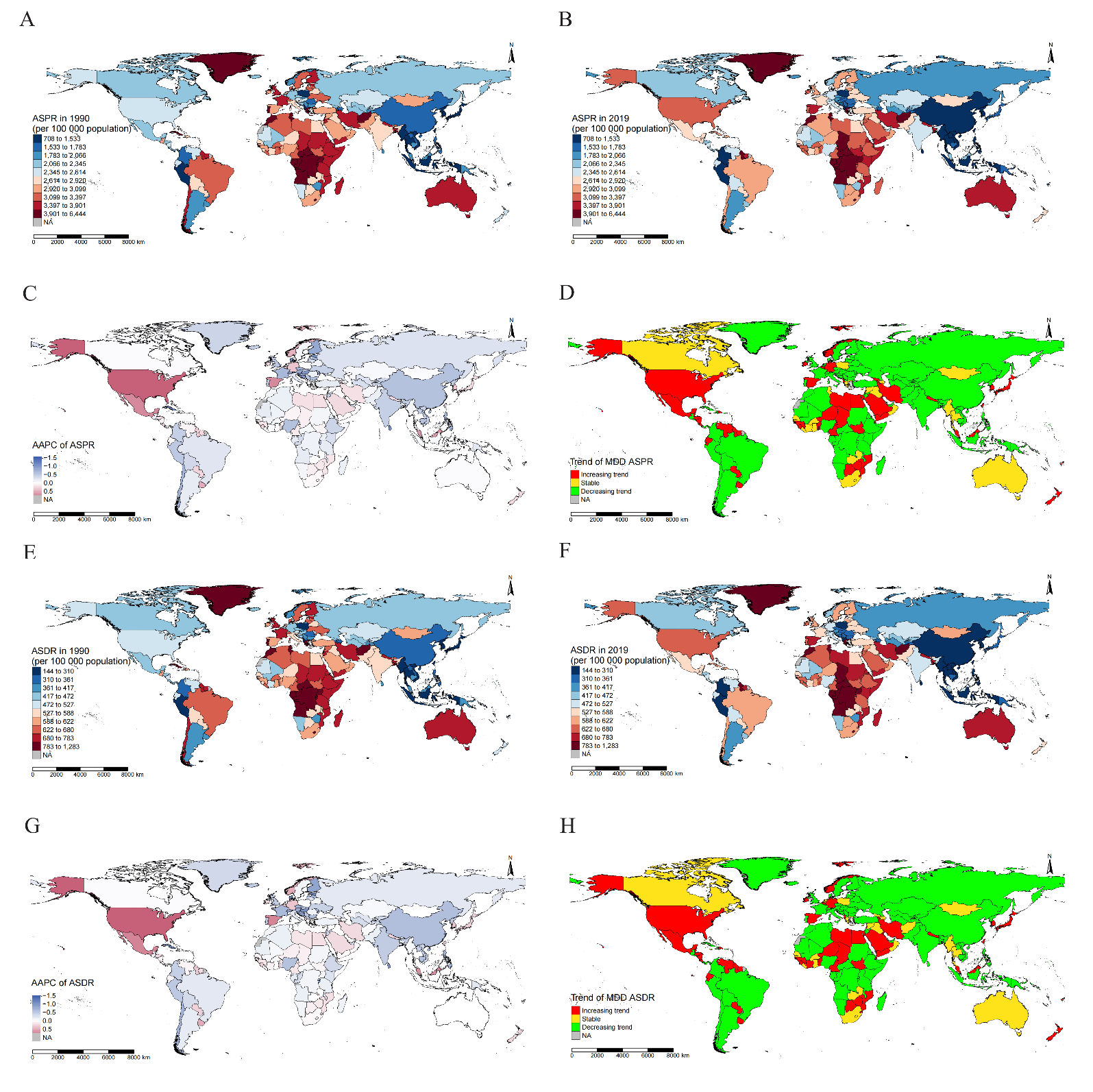


Figure S3 The ASPR of MDD across 204 countries and territories in 1990 (A) and 2019 (B). The AAPC of ASPR (C). The trend in ASPR (D). The ASDR of MDD across 204 countries and territories in 1990 (E) and 2019 (F). The AAPC of ASDR (G). The trend in ASDR (H).


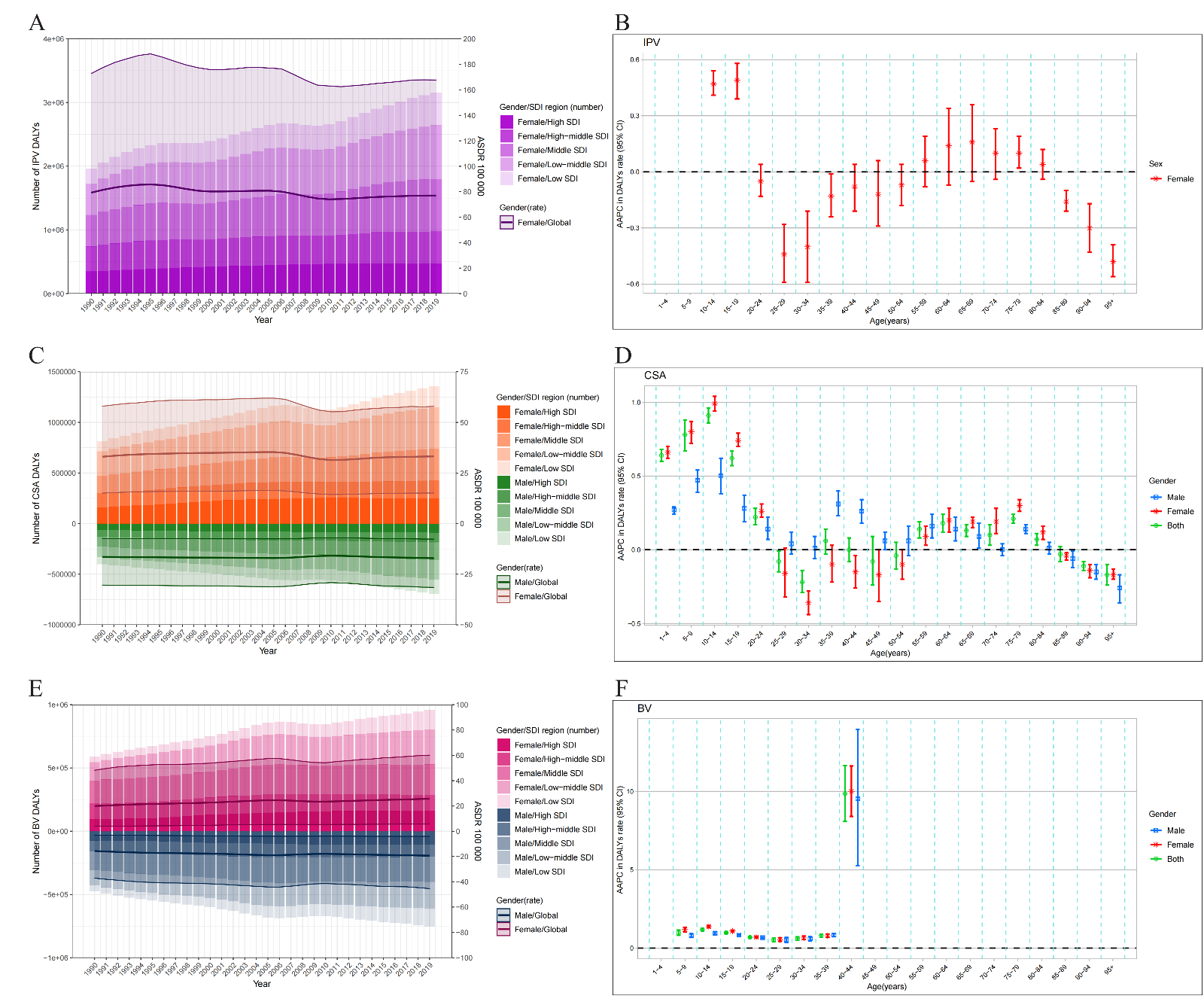


Figure S4 The number and ASDR of MDD attributed to IPV (A), CSA(C) and BV (E) during 1990-2019 by gender and SDI regions. The age-specific AAPC of DALYs rate attributed to IPV (B), CSA (D) and BV (F) by gender.


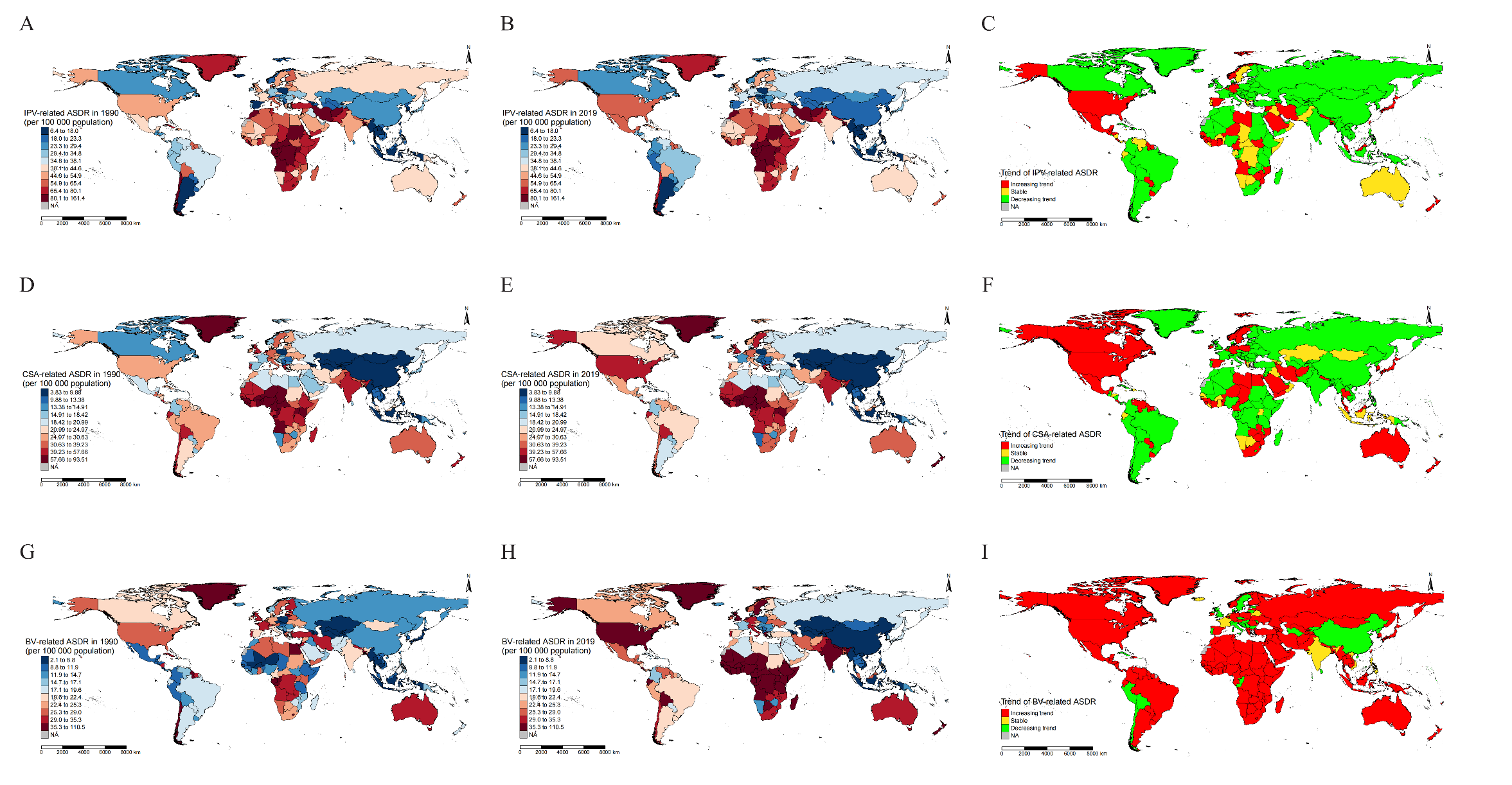


Figure S5 The ASDR of MDD across 204 countries and territories attributed to IPV in 1990 (A) and 2019 (B), CSA in 1990 (D) and 2019 (E), and BV in 1990 (G) and 2019 (H). The trend in ASDR attributed to IPV (C), CSA (F), and BV (I) during 1990-2019.


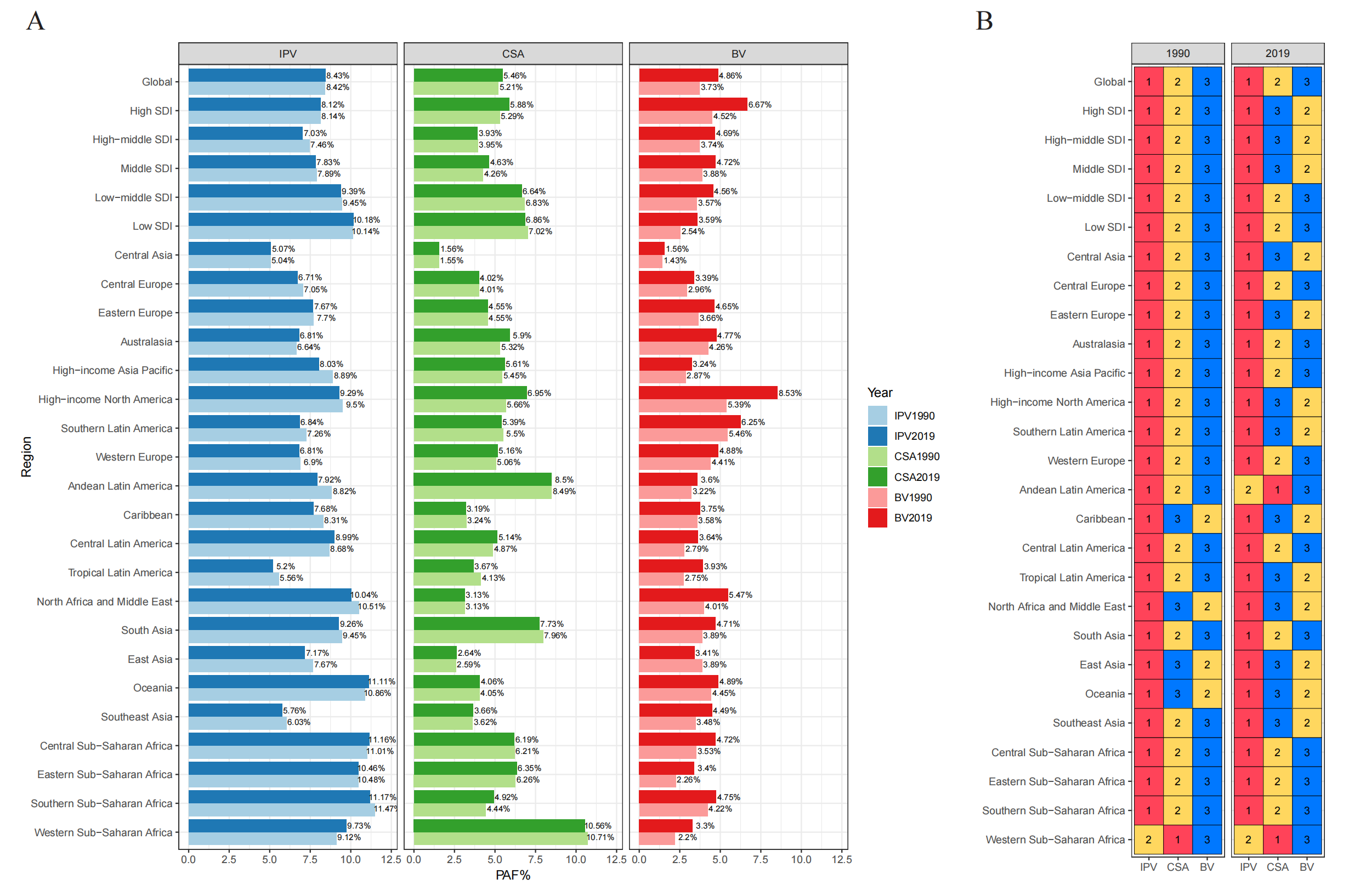


Figure S6 The PAFs of IPV, CSA and BV by SDI regions and GBD regions in 1990 and in 2019 (A), and the PAFs-ranks of IPV, CSA, and BV in 1990 and 2019 (B).


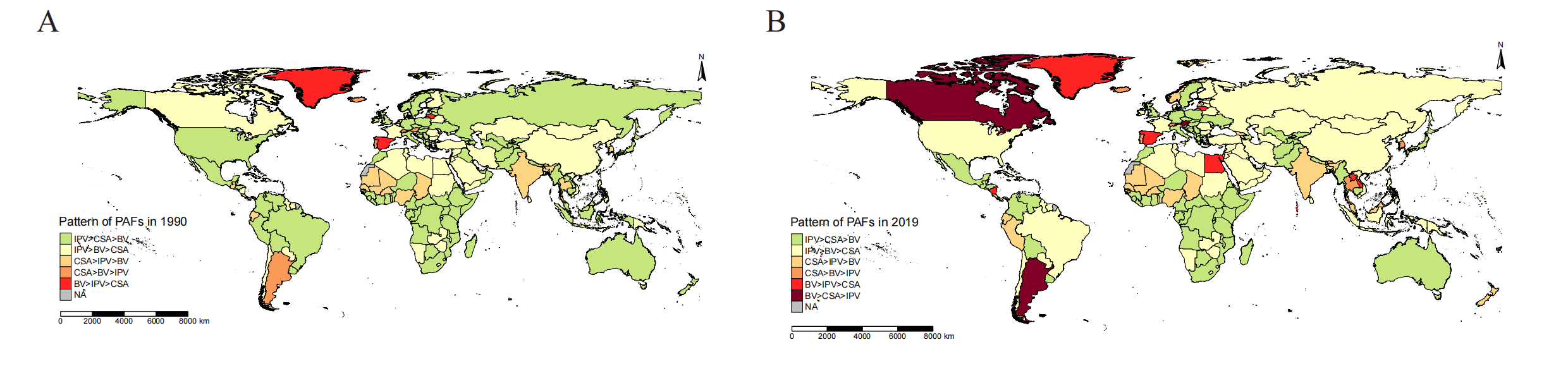


Figure S7 The spatial pattern of PAFs attributed to IPV, CSA and BV in 1990 (A) and in 2019 (B).


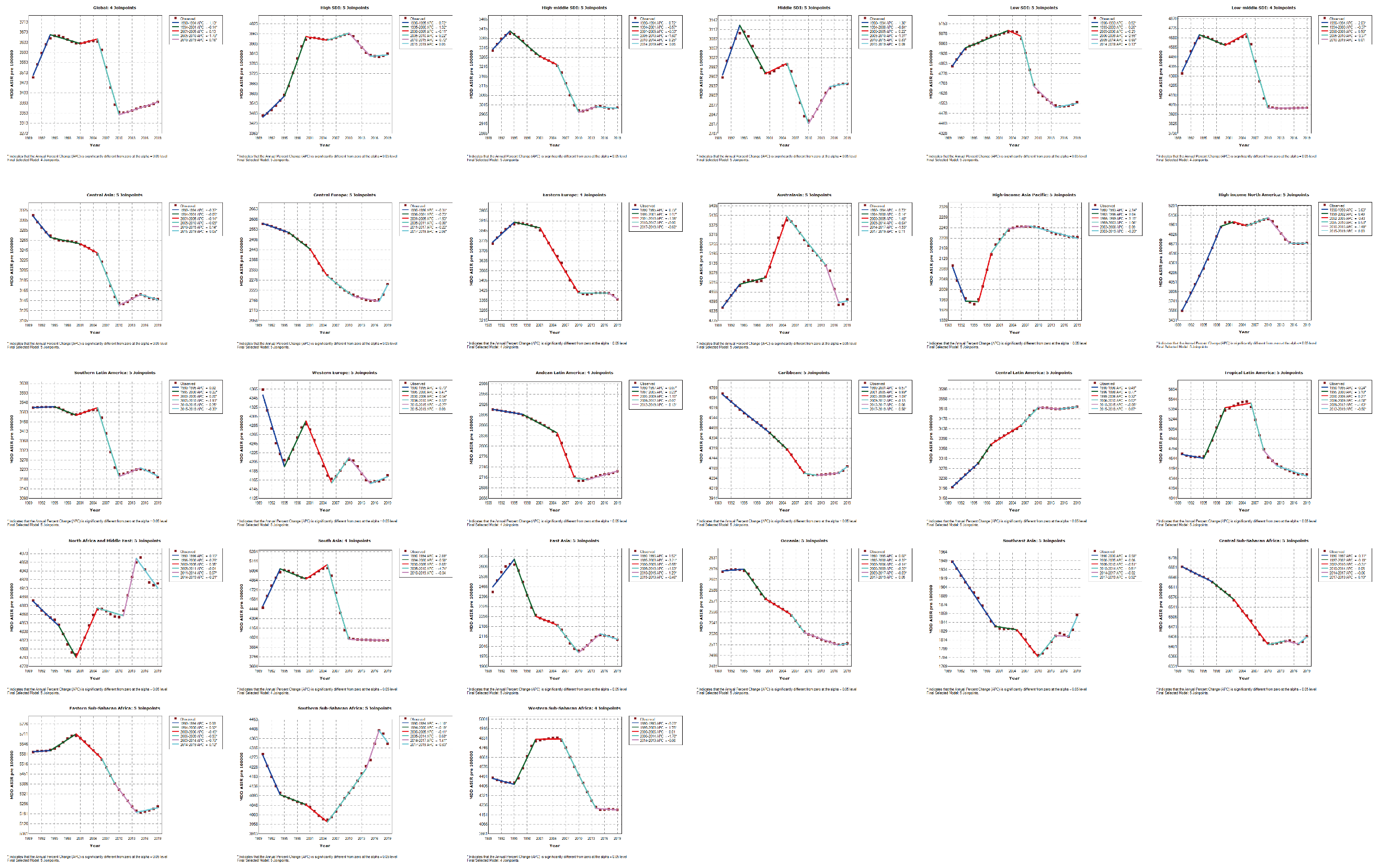


Figure S8 The trends in ASIR of MDD across globe, SDI regions and GBD regions during1990-2019.


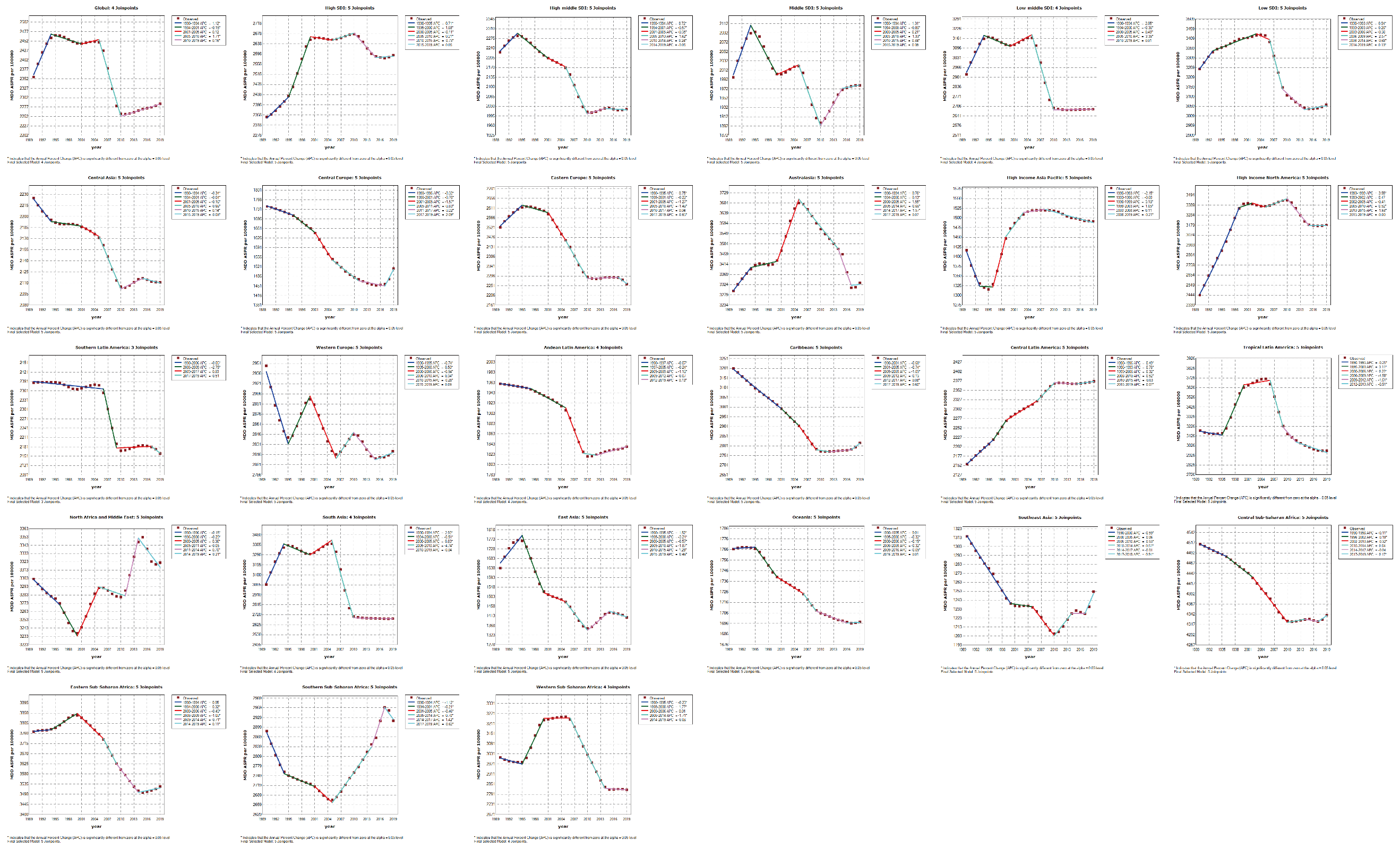


Figure S9 The trends in ASPR of MDD across globe, SDI regions and GBD regions during 1990-2019.


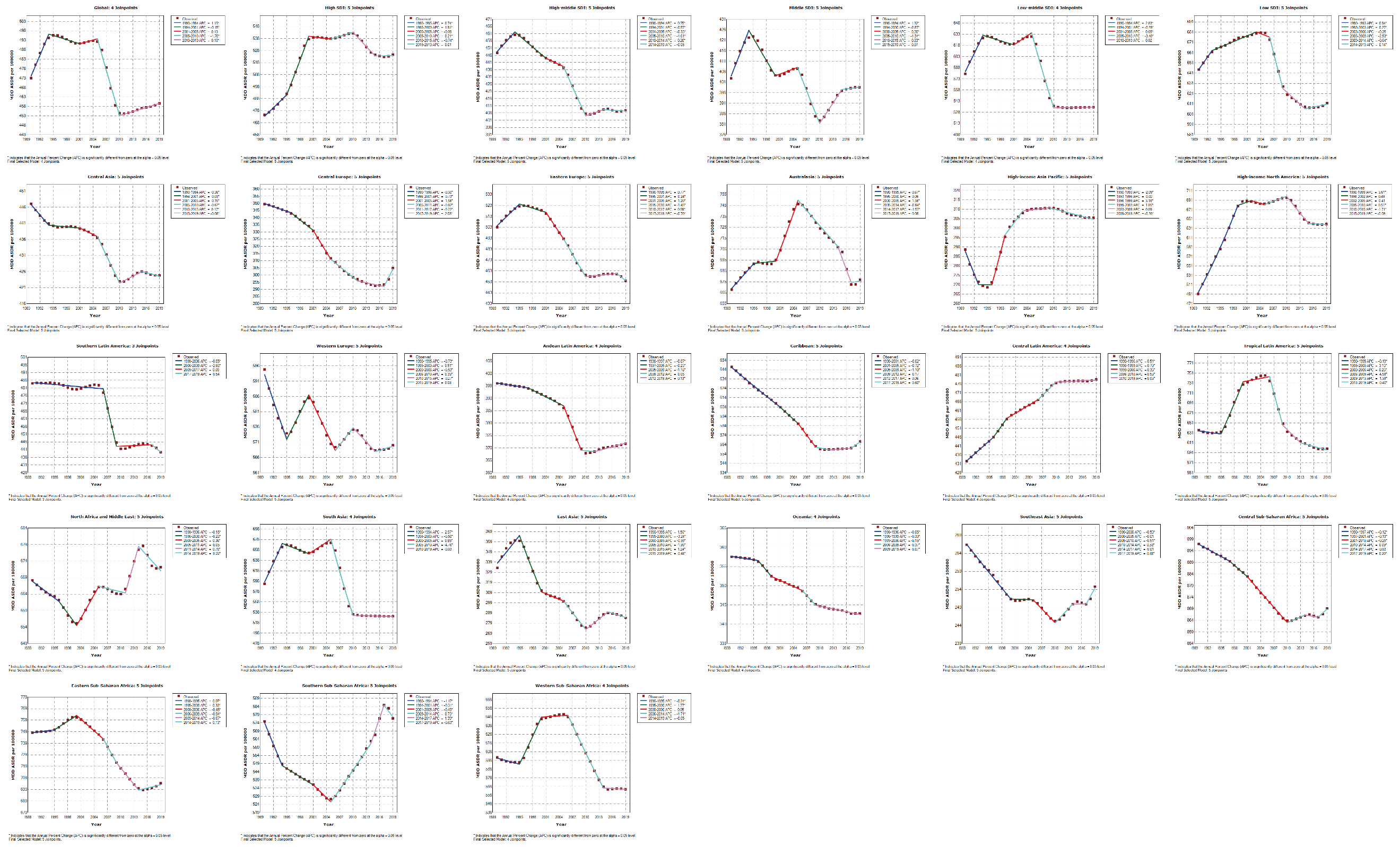


Figure S10 The trends in ASDR of MDD across globe, SDI regions and GBD regions during 1990-2019.


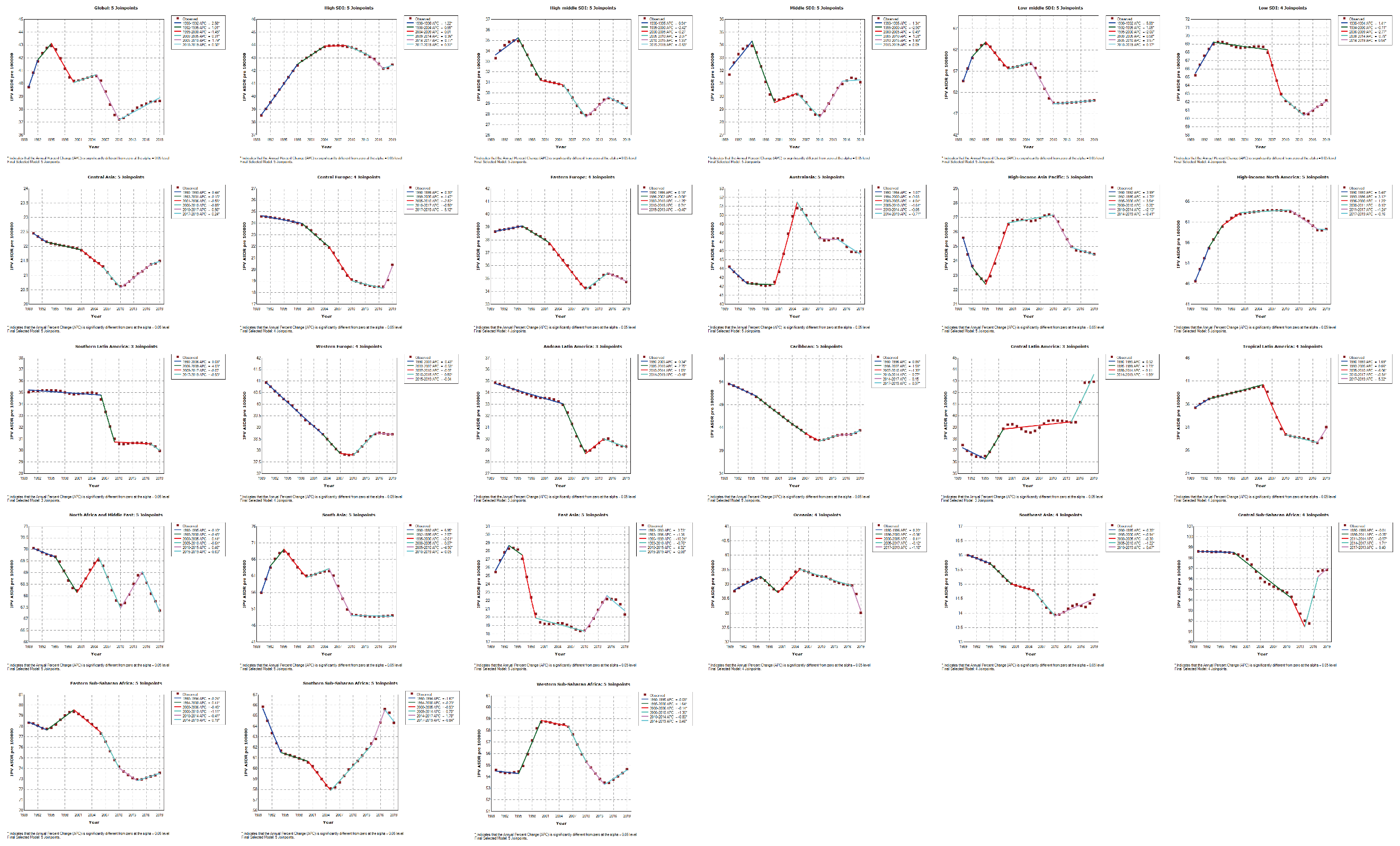


Figure S11 The trends in ASDR of MDD attributable to IPV across globe SDI regions and GBD regions during 1990-2019.


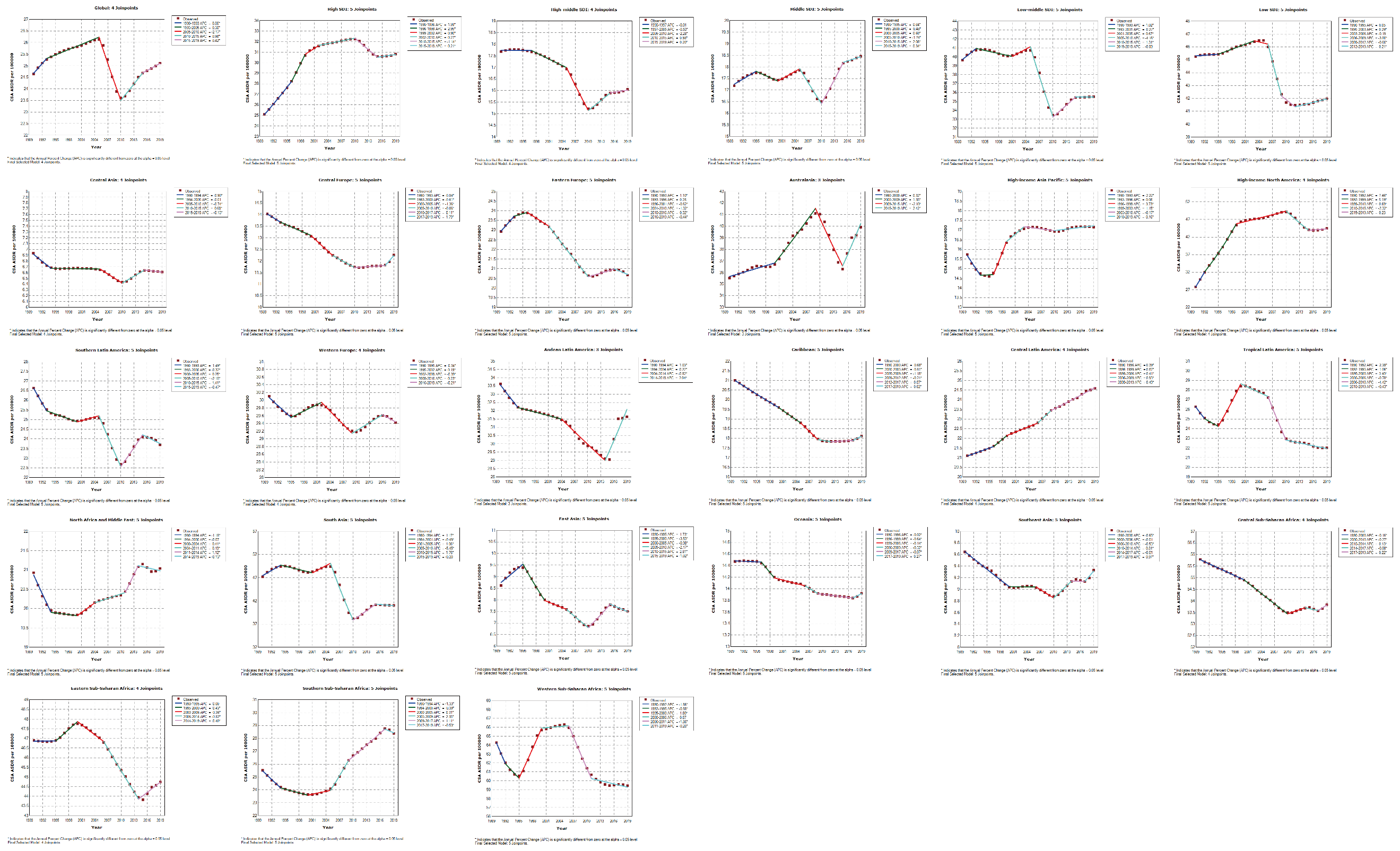


Figure S12 The trends in ASDR of MDD attributed to CSA across globe, SDI regions and GBD regions during 1990-2019.


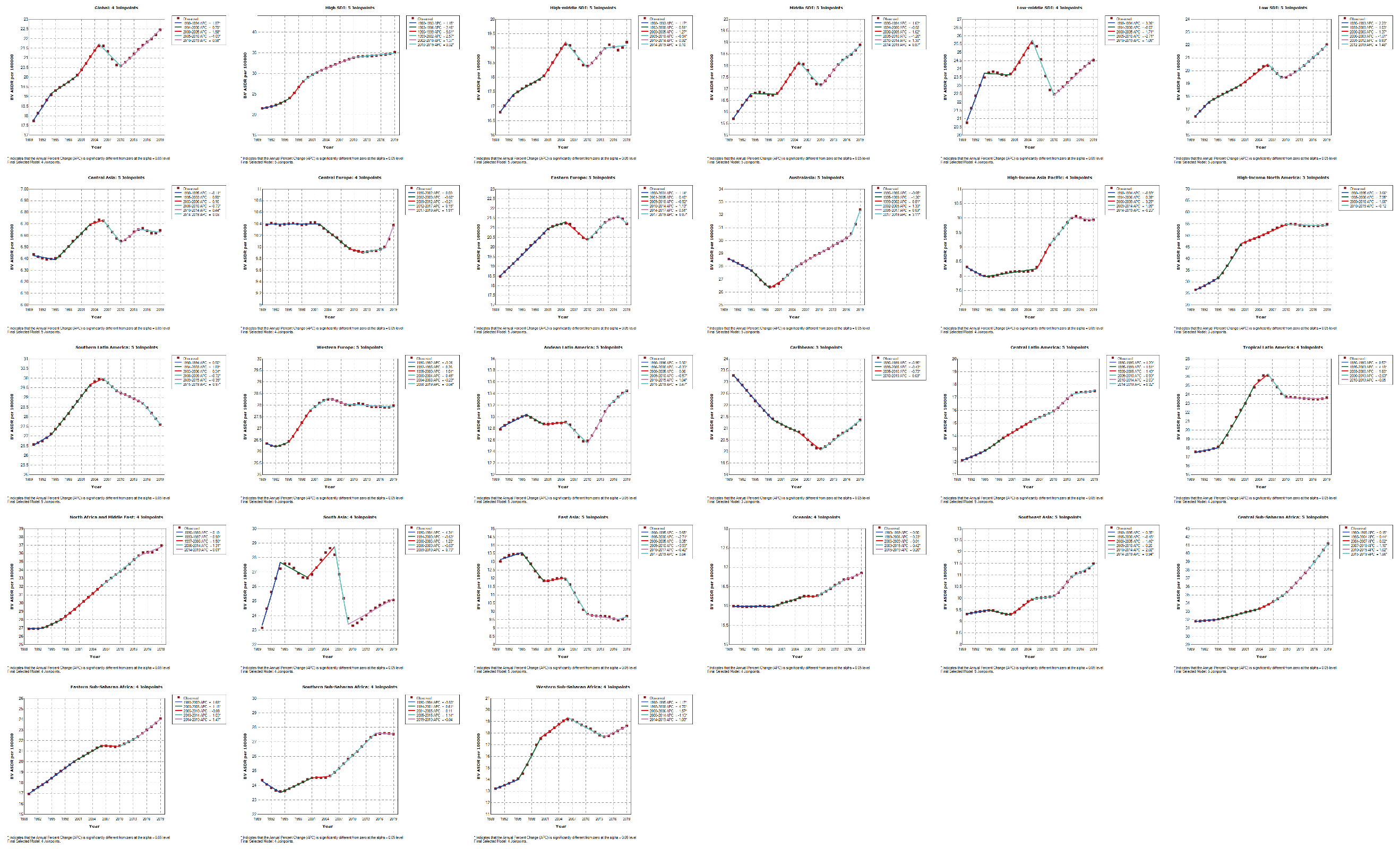


Figure S13 The trends in ASDR rate of MDD attributed to BV across globe, SDI regions and GBD regions during 1990-2019.

| Table S1 The SDI regions and the countries within each SDI regions in 2019. | | |
| --- | --- | --- |
| SDI regions | Country | SDI-value in 2019 |
| High SDI region |  |  |
|  | Switzerland | 0.929 |
|  | Norway | 0.913 |
|  | Monaco | 0.902 |
|  | Germany | 0.898 |
|  | Luxembourg | 0.895 |
|  | Andorra | 0.894 |
|  | Denmark | 0.890 |
|  | San Marino | 0.884 |
|  | Netherlands | 0.883 |
|  | United Arab Emirates | 0.880 |
|  | Republic of Korea | 0.878 |
|  | Canada | 0.873 |
|  | Sweden | 0.872 |
|  | Japan | 0.870 |
|  | Iceland | 0.869 |
|  | Taiwan (Province of China) | 0.868 |
|  | Ireland | 0.867 |
|  | Singapore | 0.861 |
|  | United States of America | 0.859 |
|  | Finland | 0.856 |
|  | Belgium | 0.851 |
|  | Kuwait | 0.851 |
|  | Austria | 0.849 |
|  | United Kingdom | 0.847 |
|  | Lithuania | 0.843 |
|  | Cyprus | 0.841 |
|  | New Zealand | 0.840 |
|  | Slovenia | 0.840 |
|  | Australia | 0.839 |
|  | Estonia | 0.835 |
|  | France | 0.834 |
|  | Qatar | 0.830 |
|  | Czechia | 0.828 |
|  | Brunei Darussalam | 0.823 |
|  | Latvia | 0.820 |
|  | Puerto Rico | 0.814 |
|  | Bermuda | 0.813 |
|  | Guam | 0.813 |
|  | Slovakia | 0.812 |
| High-middle SDI region |  |  |
|  | Russian Federation | 0.805 |
|  | Saudi Arabia | 0.805 |
|  | Israel | 0.803 |
|  | Poland | 0.802 |
|  | Italy | 0.801 |
|  | Malta | 0.801 |
|  | United States Virgin Islands | 0.799 |
|  | Bahamas | 0.796 |
|  | Croatia | 0.794 |
|  | Greece | 0.794 |
|  | Hungary | 0.791 |
|  | Montenegro | 0.791 |
|  | Oman | 0.783 |
|  | Northern Mariana Islands | 0.771 |
|  | Serbia | 0.767 |
|  | Spain | 0.767 |
|  | Bulgaria | 0.764 |
|  | Cook Islands | 0.764 |
|  | Greenland | 0.761 |
|  | Romania | 0.760 |
|  | Chile | 0.759 |
|  | Trinidad and Tobago | 0.757 |
|  | Bahrain | 0.751 |
|  | Turkey | 0.748 |
|  | Saint Kitts and Nevis | 0.746 |
|  | Belarus | 0.745 |
|  | North Macedonia | 0.744 |
|  | Antigua and Barbuda | 0.743 |
|  | Portugal | 0.743 |
|  | Barbados | 0.742 |
|  | Palau | 0.738 |
|  | Malaysia | 0.737 |
|  | Ukraine | 0.736 |
|  | Jordan | 0.731 |
|  | Dominica | 0.729 |
|  | Seychelles | 0.724 |
|  | Kazakhstan | 0.723 |
|  | Bosnia and Herzegovina | 0.718 |
|  | American Samoa | 0.712 |
|  | Niue | 0.711 |
|  | Libya | 0.709 |
|  | Argentina | 0.708 |
|  | Lebanon | 0.708 |
|  | Mauritius | 0.705 |
|  | Georgia | 0.702 |
|  | Uruguay | 0.697 |
|  | Republic of Moldova | 0.696 |
|  | Sri Lanka | 0.690 |
| Middle SDI region |  |  |
|  | Armenia | 0.689 |
|  | Thailand | 0.687 |
|  | China | 0.686 |
|  | Panama | 0.686 |
|  | Equatorial Guinea | 0.685 |
|  | Jamaica | 0.684 |
|  | Azerbaijan | 0.683 |
|  | Albania | 0.681 |
|  | Costa Rica | 0.680 |
|  | South Africa | 0.678 |
|  | Tunisia | 0.672 |
|  | Iraq | 0.671 |
|  | Iran (Islamic Republic of) | 0.670 |
|  | Saint Lucia | 0.670 |
|  | Turkmenistan | 0.670 |
|  | Grenada | 0.669 |
|  | Cuba | 0.668 |
|  | Fiji | 0.664 |
|  | Indonesia | 0.660 |
|  | Egypt | 0.658 |
|  | Gabon | 0.656 |
|  | Algeria | 0.652 |
|  | Mexico | 0.649 |
|  | Peru | 0.648 |
|  | Samoa | 0.641 |
|  | Brazil | 0.640 |
|  | Ecuador | 0.640 |
|  | Paraguay | 0.638 |
|  | Suriname | 0.636 |
|  | Tonga | 0.636 |
|  | Botswana | 0.634 |
|  | Colombia | 0.633 |
|  | Uzbekistan | 0.631 |
|  | Saint Vincent and the Grenadines | 0.627 |
|  | Tokelau | 0.626 |
|  | Philippines | 0.623 |
|  | Syrian Arab Republic | 0.619 |
|  | Guyana | 0.618 |
|  | Nauru | 0.618 |
|  | Viet Nam | 0.617 |
|  | Namibia | 0.612 |
| Low-middle SDI region |  |  |
|  | Venezuela (Bolivarian Republic of) | 0.607 |
|  | Mongolia | 0.606 |
|  | Belize | 0.603 |
|  | Kyrgyzstan | 0.596 |
|  | Dominican Republic | 0.592 |
|  | Tuvalu | 0.589 |
|  | Palestine | 0.588 |
|  | Micronesia (Federated States of) | 0.580 |
|  | Eswatini | 0.577 |
|  | El Salvador | 0.573 |
|  | Congo | 0.568 |
|  | Bolivia (Plurinational State of) | 0.566 |
|  | India | 0.566 |
|  | Maldives | 0.562 |
|  | Democratic People's Republic of Korea | 0.558 |
|  | Ghana | 0.557 |
|  | Morocco | 0.548 |
|  | Marshall Islands | 0.544 |
|  | Tajikistan | 0.539 |
|  | Kiribati | 0.527 |
|  | Guatemala | 0.526 |
|  | Cabo Verde | 0.525 |
|  | Myanmar | 0.521 |
|  | Nicaragua | 0.517 |
|  | Nigeria | 0.515 |
|  | Sudan | 0.515 |
|  | Timor-Leste | 0.514 |
|  | Kenya | 0.508 |
|  | Lesotho | 0.507 |
|  | Zambia | 0.505 |
|  | Sao Tome and Principe | 0.502 |
|  | Honduras | 0.496 |
|  | Mauritania | 0.496 |
|  | Cameroon | 0.490 |
|  | Lao People's Democratic Republic | 0.490 |
|  | Vanuatu | 0.485 |
|  | Bangladesh | 0.483 |
|  | Zimbabwe | 0.476 |
|  | Angola | 0.470 |
|  | Cambodia | 0.469 |
|  | Djibouti | 0.459 |
|  | Bhutan | 0.455 |
|  | Comoros | 0.455 |
| Low SDI region |  |  |
|  | Pakistan | 0.449 |
|  | Haiti | 0.432 |
|  | Rwanda | 0.429 |
|  | United Republic of Tanzania | 0.423 |
|  | Nepal | 0.422 |
|  | Togo | 0.417 |
|  | Yemen | 0.412 |
|  | Côte d'Ivoire | 0.408 |
|  | Solomon Islands | 0.407 |
|  | Uganda | 0.404 |
|  | Gambia | 0.399 |
|  | Eritrea | 0.396 |
|  | Madagascar | 0.396 |
|  | Papua New Guinea | 0.394 |
|  | Senegal | 0.389 |
|  | Malawi | 0.384 |
|  | Democratic Republic of the Congo | 0.382 |
|  | Liberia | 0.370 |
|  | South Sudan | 0.363 |
|  | Guinea-Bissau | 0.355 |
|  | Benin | 0.352 |
|  | Sierra Leone | 0.347 |
|  | Afghanistan | 0.343 |
|  | Ethiopia | 0.343 |
|  | Guinea | 0.325 |
|  | Mozambique | 0.307 |
|  | Burundi | 0.284 |
|  | Central African Republic | 0.274 |
|  | Mali | 0.263 |
|  | Burkina Faso | 0.257 |
|  | Chad | 0.238 |
|  | Niger | 0.162 |
|  | Somalia | 0.081 |

| Table S2 The GBD regions and the countries within each GBD regions. | |
| --- | --- |
| GBD Regions | Country |
| Central Asia |  |
|  | Armenia |
|  | Azerbaijan |
|  | Georgia |
|  | Kazakhstan |
|  | Kyrgyzstan |
|  | Mongolia |
|  | Tajikistan |
|  | Turkmenistan |
|  | Uzbekistan |
| Central Europe |  |
|  | Albania |
|  | Bosnia and Herzegovina |
|  | Bulgaria |
|  | Croatia |
|  | Czechia |
|  | Hungary |
|  | Montenegro |
|  | North Macedonia |
|  | Poland |
|  | Romania |
|  | Serbia |
|  | Slovakia |
|  | Slovenia |
| Eastern Europe |  |
|  | Belarus |
|  | Estonia |
|  | Latvia |
|  | Lithuania |
|  | Republic of Moldova |
|  | Russian Federation |
|  | Ukraine |
| Australasia |  |
|  | Australia |
|  | New Zealand |
| High-income Asia Pacific |  |
|  | Brunei Darussalam |
|  | Japan |
|  | Republic of Korea |
|  | Singapore |
| High-income North America |  |
|  | Canada |
|  | Greenland |
|  | United States of America |
| Southern Latin America |  |
|  | Argentina |
|  | Chile |
|  | Uruguay |
| Western Europe |  |
|  | Andorra |
|  | Austria |
|  | Belgium |
|  | Cyprus |
|  | Denmark |
|  | Finland |
|  | France |
|  | Germany |
|  | Greece |
|  | Iceland |
|  | Ireland |
|  | Israel |
|  | Italy |
|  | Luxembourg |
|  | Malta |
|  | Monaco |
|  | Netherlands |
|  | Norway |
|  | Portugal |
|  | San Marino |
|  | Spain |
|  | Sweden |
|  | Switzerland |
|  | United Kingdom |
| Andean Latin America |  |
|  | Bolivia (Plurinational State of) |
|  | Ecuador |
|  | Peru |
| Caribbean |  |
|  | Antigua and Barbuda |
|  | Bahamas |
|  | Barbados |
|  | Belize |
|  | Bermuda |
|  | Cuba |
|  | Dominica |
|  | Dominican Republic |
|  | Grenada |
|  | Guyana |
|  | Haiti |
|  | Jamaica |
|  | Puerto Rico |
|  | Saint Kitts and Nevis |
|  | Saint Lucia |
|  | Saint Vincent and the Grenadines |
|  | Suriname |
|  | Trinidad and Tobago |
|  | United States Virgin Islands |
| Central Latin America |  |
|  | Colombia |
|  | Costa Rica |
|  | El Salvador |
|  | Guatemala |
|  | Honduras |
|  | Mexico |
|  | Nicaragua |
|  | Panama |
|  | Venezuela (Bolivarian Republic of) |
| Tropical Latin America |  |
|  | Brazil |
|  | Paraguay |
| North Africa and Middle East |  |
|  | Afghanistan |
|  | Algeria |
|  | Bahrain |
|  | Egypt |
|  | Iran (Islamic Republic of) |
|  | Iraq |
|  | Jordan |
|  | Kuwait |
|  | Lebanon |
|  | Libya |
|  | Morocco |
|  | Oman |
|  | Palestine |
|  | Qatar |
|  | Saudi Arabia |
|  | Sudan |
|  | Syrian Arab Republic |
|  | Tunisia |
|  | Turkey |
|  | United Arab Emirates |
|  | Yemen |
| South Asia |  |
|  | Bangladesh |
|  | Bhutan |
|  | India |
|  | Nepal |
|  | Pakistan |
| East Asia |  |
|  | China |
|  | Democratic People's Republic of Korea |
|  | Taiwan (Province of China) |
| Oceania |  |
|  | American Samoa |
|  | Cook Islands |
|  | Fiji |
|  | Guam |
|  | Kiribati |
|  | Marshall Islands |
|  | Micronesia (Federated States of) |
|  | Nauru |
|  | Niue |
|  | Northern Mariana Islands |
|  | Palau |
|  | Papua New Guinea |
|  | Samoa |
|  | Solomon Islands |
|  | Tokelau |
|  | Tonga |
|  | Tuvalu |
|  | Vanuatu |
| Southeast Asia |  |
|  | Cambodia |
|  | Indonesia |
|  | Lao People's Democratic Republic |
|  | Malaysia |
|  | Maldives |
|  | Mauritius |
|  | Myanmar |
|  | Philippines |
|  | Seychelles |
|  | Sri Lanka |
|  | Thailand |
|  | Timor-Leste |
|  | Viet Nam |
| Central Sub-Saharan Africa |  |
|  | Angola |
|  | Central African Republic |
|  | Congo |
|  | Democratic Republic of the Congo |
|  | Equatorial Guinea |
|  | Gabon |
| Eastern Sub-Saharan Africa |  |
|  | Burundi |
|  | Comoros |
|  | Djibouti |
|  | Eritrea |
|  | Ethiopia |
|  | Kenya |
|  | Madagascar |
|  | Malawi |
|  | Mozambique |
|  | Rwanda |
|  | Somalia |
|  | South Sudan |
|  | Uganda |
|  | United Republic of Tanzania |
|  | Zambia |
| Southern Sub-Saharan Africa |  |
|  | Botswana |
|  | Eswatini |
|  | Lesotho |
|  | Namibia |
|  | South Africa |
|  | Zimbabwe |
| Western Sub-Saharan Africa |  |
|  | Benin |
|  | Burkina Faso |
|  | Cabo Verde |
|  | Cameroon |
|  | Chad |
|  | Côte d'Ivoire |
|  | Gambia |
|  | Ghana |
|  | Guinea |
|  | Guinea-Bissau |
|  | Liberia |
|  | Mali |
|  | Mauritania |
|  | Niger |
|  | Nigeria |
|  | Sao Tome and Principe |
|  | Senegal |
|  | Sierra Leone |
|  | Togo |

| Table S3 The APC of ASIR, ASPR, and ASDR across SDI regions and GBD regions. | | | | | | | | | | |
| --- | --- | --- | --- | --- | --- | --- | --- | --- | --- | --- |
|  | ASIR |  |  |  | ASPR |  |  |  | ASDR |  |
| Characteristics | Period | APC (95% CI) |  | Characteristics | Period | APC (95% CI) |  | Characteristics | Period | APC (95% CI) |
| **SDI regions** |  |  |  |  |  |  |  |  |  |  |
| High SDI | 1990-1995 | 0.72 (0.64, 0.80)* |  | High SDI | 1990-1995 | 0.71 (0.63, 0.78)* |  | High SDI | 1990-1995 | 0.74 (0.67, 0.81)* |
| High SDI | 1995-2000 | 1.92 (1.81, 2.04)* |  | High SDI | 1995-2000 | 1.88 (1.78, 1.98)* |  | High SDI | 1995-2000 | 1.91 (1.81, 2.01)* |
| High SDI | 2000-2005 | -0.11 (-0.22, -0.00)* |  | High SDI | 2000-2005 | -0.10 (-0.20, -0.01)* |  | High SDI | 2000-2005 | -0.09 (-0.19, 0.01) |
| High SDI | 2005-2010 | 0.22 (0.11, 0.33)* |  | High SDI | 2005-2010 | 0.21 (0.11, 0.31)* |  | High SDI | 2005-2010 | 0.21 (0.11, 0.31)* |
| High SDI | 2010-2015 | -0.72 (-0.83, -0.60)* |  | High SDI | 2010-2015 | -0.73 (-0.83, -0.63)* |  | High SDI | 2010-2015 | -0.74 (-0.84, -0.64)* |
| High SDI | 2015-2019 | 0.05 (-0.07, 0.18) |  | High SDI | 2015-2019 | 0.05 (-0.06, 0.16) |  | High SDI | 2015-2019 | 0.01 (-0.09, 0.12) |
| High-middle SDI | 1990-1994 | 0.72 (0.60, 0.83)* |  | High-middle SDI | 1990-1994 | 0.71 (0.60, 0.83)* |  | High-middle SDI | 1990-1994 | 0.74 (0.63, 0.86)* |
| High-middle SDI | 1994-2001 | -0.57 (-0.63, -0.51)* |  | High-middle SDI | 1994-2001 | -0.57 (-0.63, -0.51)* |  | High-middle SDI | 1994-2001 | -0.57 (-0.64, -0.51)* |
| High-middle SDI | 2001-2005 | -0.34 (-0.50, -0.17)* |  | High-middle SDI | 2001-2005 | -0.35 (-0.52, -0.19)* |  | High-middle SDI | 2001-2005 | -0.33 (-0.51, -0.14)* |
| High-middle SDI | 2005-2010 | -1.60 (-1.70, -1.50)* |  | High-middle SDI | 2005-2010 | -1.62 (-1.72, -1.51)* |  | High-middle SDI | 2005-2010 | -1.61 (-1.72, -1.49)* |
| High-middle SDI | 2010-2014 | 0.25 (0.08, 0.41)* |  | High-middle SDI | 2010-2014 | 0.24 (0.07, 0.41)* |  | High-middle SDI | 2010-2014 | 0.26 (0.08, 0.45)* |
| High-middle SDI | 2014-2019 | -0.05 (-0.14, 0.03) |  | High-middle SDI | 2014-2019 | -0.05 (-0.13, 0.03) |  | High-middle SDI | 2014-2019 | -0.05 (-0.13, 0.04) |
| Middle SDI | 1990-1994 | 1.30 (1.13, 1.47)* |  | Middle SDI | 1990-1994 | 1.31 (1.14, 1.48)* |  | Middle SDI | 1990-1994 | 1.32 (1.15, 1.50)* |
| Middle SDI | 1994-2000 | -0.84 (-0.95, -0.72)* |  | Middle SDI | 1994-2000 | -0.86 (-0.97, -0.74)* |  | Middle SDI | 1994-2000 | -0.87 (-0.98, -0.75)* |
| Middle SDI | 2000-2005 | 0.22 (0.06, 0.38)* |  | Middle SDI | 2000-2005 | 0.21 (0.05, 0.36)* |  | Middle SDI | 2000-2005 | 0.20 (0.03, 0.36)* |
| Middle SDI | 2005-2010 | -1.31 (-1.46, -1.16)* |  | Middle SDI | 2005-2010 | -1.33 (-1.48, -1.18)* |  | Middle SDI | 2005-2010 | -1.34 (-1.50, -1.18)* |
| Middle SDI | 2010-2015 | 0.83 (0.67, 0.98)* |  | Middle SDI | 2010-2015 | 0.84 (0.68, 0.99)* |  | Middle SDI | 2010-2015 | 0.83 (0.66, 1.00)* |
| Middle SDI | 2015-2019 | 0.09 (-0.08, 0.25) |  | Middle SDI | 2015-2019 | 0.09 (-0.08, 0.26) |  | Middle SDI | 2015-2019 | 0.07 (-0.10, 0.24) |
| Low-middle SDI | 1990-1994 | 2.03 (1.87, 2.19)* |  | Low-middle SDI | 1990-1994 | 2.05 (1.88, 2.22)* |  | Low-middle SDI | 1990-1994 | 2.03 (1.86, 2.19)* |
| Low-middle SDI | 1994-2000 | -0.37 (-0.48, -0.26)* |  | Low-middle SDI | 1994-2000 | -0.38 (-0.50, -0.27)* |  | Low-middle SDI | 1994-2001 | -0.29 (-0.38, -0.21)* |
| Low-middle SDI | 2000-2005 | 0.50 (0.35, 0.66)* |  | Low-middle SDI | 2000-2005 | 0.49 (0.34, 0.65)* |  | Low-middle SDI | 2001-2005 | 0.66 (0.40, 0.91)* |
| Low-middle SDI | 2005-2010 | -3.37 (-3.52, -3.22)* |  | Low-middle SDI | 2005-2010 | -3.38 (-3.53, -3.23)* |  | Low-middle SDI | 2005-2010 | -3.40 (-3.56, -3.25)* |
| Low-middle SDI | 2010-2019 | 0.01 (-0.03, 0.05) |  | Low-middle SDI | 2010-2019 | 0.01 (-0.04, 0.05) |  | Low-middle SDI | 2010-2019 | 0.02 (-0.03, 0.06) |
| Low SDI | 1990-1993 | 0.92 (0.74, 1.10)* |  | Low SDI | 1990-1993 | 0.94 (0.75, 1.13)* |  | Low SDI | 1990-1993 | 0.94 (0.76, 1.12)* |
| Low SDI | 1993-2003 | 0.26 (0.23, 0.29)* |  | Low SDI | 1993-2003 | 0.26 (0.23, 0.29)* |  | Low SDI | 1993-2003 | 0.27 (0.24, 0.30)* |
| Low SDI | 2003-2006 | -0.29 (-0.64, 0.05) |  | Low SDI | 2003-2006 | -0.30 (-0.65, 0.05) |  | Low SDI | 2003-2006 | -0.25 (-0.61, 0.10) |
| Low SDI | 2006-2009 | -2.55 (-2.88, -2.23)* |  | Low SDI | 2006-2009 | -2.57 (-2.91, -2.24)* |  | Low SDI | 2006-2009 | -2.53 (-2.88, -2.19)* |
| Low SDI | 2009-2014 | -0.68 (-0.78, -0.57)* |  | Low SDI | 2009-2014 | -0.68 (-0.79, -0.57)* |  | Low SDI | 2009-2014 | -0.64 (-0.75, -0.52)* |
| Low SDI | 2014-2019 | 0.13 (0.05, 0.21)* |  | Low SDI | 2014-2019 | 0.13 (0.04, 0.21)* |  | Low SDI | 2014-2019 | 0.14 (0.06, 0.22)* |
| **GBD regions** |  |  |  |  |  |  |  |  |  |  |
| Central Asia | 1990-1994 | -0.32 (-0.35, -0.29)* |  | Central Asia | 1990-1994 | -0.34 (-0.38, -0.31)* |  | Central Asia | 1990-1994 | -0.36 (-0.40, -0.33)* |
| Central Asia | 1994-2001 | -0.06 (-0.07, -0.04)* |  | Central Asia | 1994-2001 | -0.04 (-0.06, -0.02)* |  | Central Asia | 1994-2001 | -0.03 (-0.04, -0.01)* |
| Central Asia | 2001-2005 | -0.14 (-0.19, -0.09)* |  | Central Asia | 2001-2005 | -0.16 (-0.21, -0.11)* |  | Central Asia | 2001-2005 | -0.15 (-0.20, -0.10)* |
| Central Asia | 2005-2010 | -0.65 (-0.68, -0.62)* |  | Central Asia | 2005-2010 | -0.66 (-0.69, -0.63)* |  | Central Asia | 2005-2010 | -0.67 (-0.70, -0.64)* |
| Central Asia | 2010-2015 | 0.14 (0.11, 0.17)* |  | Central Asia | 2010-2015 | 0.14 (0.10, 0.17)* |  | Central Asia | 2010-2015 | 0.17 (0.14, 0.20)* |
| Central Asia | 2015-2019 | -0.08 (-0.11, -0.05)* |  | Central Asia | 2015-2019 | -0.08 (-0.11, -0.04)* |  | Central Asia | 2015-2019 | -0.08 (-0.11, -0.05)* |
| Central Europe | 1990-1996 | -0.31 (-0.37, -0.26)* |  | Central Europe | 1990-1996 | -0.32 (-0.38, -0.26)* |  | Central Europe | 1990-1996 | -0.30 (-0.36, -0.24)* |
| Central Europe | 1996-2001 | -0.73 (-0.83, -0.62)* |  | Central Europe | 1996-2001 | -0.76 (-0.87, -0.65)* |  | Central Europe | 1996-2001 | -0.73 (-0.84, -0.62)* |
| Central Europe | 2001-2005 | -1.53 (-1.70, -1.37)* |  | Central Europe | 2001-2005 | -1.57 (-1.74, -1.39)* |  | Central Europe | 2001-2005 | -1.56 (-1.73, -1.39)* |
| Central Europe | 2005-2011 | -0.80 (-0.88, -0.73)* |  | Central Europe | 2005-2011 | -0.83 (-0.91, -0.75)* |  | Central Europe | 2005-2011 | -0.82 (-0.90, -0.74)* |
| Central Europe | 2011-2017 | -0.22 (-0.29, -0.14)* |  | Central Europe | 2011-2017 | -0.22 (-0.30, -0.14)* |  | Central Europe | 2011-2017 | -0.22 (-0.30, -0.15)* |
| Central Europe | 2017-2019 | 2.06 (1.68, 2.44)* |  | Central Europe | 2017-2019 | 2.09 (1.70, 2.48)* |  | Central Europe | 2017-2019 | 2.08 (1.72, 2.45)* |
| Eastern Europe | 1990-1995 | 0.73 (0.60, 0.86)* |  | Eastern Europe | 1990-1995 | 0.75 (0.67, 0.83)* |  | Eastern Europe | 1990-1995 | 0.77 (0.69, 0.85)* |
| Eastern Europe | 1995-2001 | -0.17 (-0.30, -0.05)* |  | Eastern Europe | 1995-2001 | -0.22 (-0.30, -0.14)* |  | Eastern Europe | 1995-2001 | -0.24 (-0.32, -0.16)* |
| Eastern Europe | 2001-2010 | -1.39 (-1.45, -1.33)* |  | Eastern Europe | 2001-2005 | -1.27 (-1.45, -1.09)* |  | Eastern Europe | 2001-2005 | -1.20 (-1.38, -1.02)* |
| Eastern Europe | 2010-2017 | 0.00 (-0.09, 0.09) |  | Eastern Europe | 2005-2010 | -1.48 (-1.59, -1.37)* |  | Eastern Europe | 2005-2010 | -1.45 (-1.56, -1.33)* |
| Eastern Europe | 2017-2019 | -0.60 (-1.17, -0.04)* |  | Eastern Europe | 2010-2017 | 0.04 (-0.02, 0.10) |  | Eastern Europe | 2010-2017 | 0.09 (0.03, 0.15)* |
| Australasia | 1990-1994 | 0.73 (0.62, 0.84)* |  | Eastern Europe | 2017-2019 | -0.65 (-1.01, -0.29)* |  | Eastern Europe | 2017-2019 | -0.70 (-1.07, -0.33)* |
| Australasia | 1994-2000 | 0.14 (0.06, 0.21)* |  | Australasia | 1990-1994 | 0.76 (0.64, 0.88)* |  | Australasia | 1990-1995 | 0.67 (0.60, 0.74)* |
| Australasia | 2000-2005 | 1.48 (1.38, 1.58)* |  | Australasia | 1994-2000 | 0.13 (0.06, 0.21)* |  | Australasia | 1995-2000 | 0.06 (-0.04, 0.17) |
| Australasia | 2005-2014 | -0.64 (-0.68, -0.60)* |  | Australasia | 2000-2005 | 1.55 (1.45, 1.66)* |  | Australasia | 2000-2005 | 1.56 (1.46, 1.66)* |
| Australasia | 2014-2017 | -1.55 (-1.94, -1.17)* |  | Australasia | 2005-2014 | -0.68 (-0.71, -0.64)* |  | Australasia | 2005-2014 | -0.64 (-0.68, -0.61)* |
| Australasia | 2017-2019 | 0.11 (-0.33, 0.54) |  | Australasia | 2014-2017 | -1.56 (-1.98, -1.14)* |  | Australasia | 2014-2017 | -1.57 (-1.90, -1.24)* |
| High-income Asia Pacific | 1990-1993 | -2.14 (-2.37, -1.90)* |  | Australasia | 2017-2019 | 0.07 (-0.41, 0.56) |  | Australasia | 2017-2019 | 0.08 (-0.27, 0.43) |
| High-income Asia Pacific | 1993-1996 | -0.04 (-0.51, 0.44) |  | High-income Asia Pacific | 1990-1993 | -2.15 (-2.39, -1.90)* |  | High-income Asia Pacific | 1990-1993 | -2.09 (-2.33, -1.85)* |
| High-income Asia Pacific | 1996-1999 | 3.15 (2.67, 3.62)* |  | High-income Asia Pacific | 1993-1996 | -0.03 (-0.52, 0.45) |  | High-income Asia Pacific | 1993-1996 | -0.02 (-0.51, 0.47) |
| High-income Asia Pacific | 1999-2003 | 1.06 (0.83, 1.29)* |  | High-income Asia Pacific | 1996-1999 | 3.18 (2.70, 3.66)* |  | High-income Asia Pacific | 1996-1999 | 3.16 (2.67, 3.66)* |
| High-income Asia Pacific | 2003-2008 | 0.09 (-0.05, 0.23) |  | High-income Asia Pacific | 1999-2003 | 1.05 (0.82, 1.28)* |  | High-income Asia Pacific | 1999-2003 | 1.05 (0.81, 1.30)* |
| High-income Asia Pacific | 2008-2019 | -0.20 (-0.23, -0.17)* |  | High-income Asia Pacific | 2003-2008 | 0.10 (-0.03, 0.24) |  | High-income Asia Pacific | 2003-2009 | 0.09 (-0.02, 0.20) |
| High-income North America | 1990-1999 | 3.63 (3.54, 3.73)* |  | High-income Asia Pacific | 2008-2019 | -0.21 (-0.24, -0.17)* |  | High-income Asia Pacific | 2009-2019 | -0.20 (-0.24, -0.16)* |
| High-income North America | 1999-2002 | 0.49 (-0.47, 1.46) |  | High-income North America | 1990-1999 | 3.55 (3.44, 3.66)* |  | High-income North America | 1990-1999 | 3.61 (3.50, 3.72)* |
| High-income North America | 2002-2005 | -0.43 (-1.36, 0.51) |  | High-income North America | 1999-2002 | 0.47 (-0.64, 1.59) |  | High-income North America | 1999-2002 | 0.49 (-0.69, 1.69) |
| High-income North America | 2005-2010 | 0.53 (0.23, 0.82)* |  | High-income North America | 2002-2005 | -0.41 (-1.49, 0.67) |  | High-income North America | 2002-2005 | -0.41 (-1.58, 0.78) |
| High-income North America | 2010-2015 | -1.69 (-1.98, -1.40)* |  | High-income North America | 2005-2010 | 0.52 (0.19, 0.86)* |  | High-income North America | 2005-2010 | 0.51 (0.14, 0.89)* |
| High-income North America | 2015-2019 | 0.00 (-0.31, 0.31) |  | High-income North America | 2010-2015 | -1.68 (-2.02, -1.35)* |  | High-income North America | 2010-2015 | -1.72 (-2.08, -1.35)* |
| Southern Latin America | 1990-1995 | 0.02 (-0.03, 0.07) |  | High-income North America | 2015-2019 | -0.00 (-0.36, 0.36) |  | High-income North America | 2015-2019 | -0.09 (-0.46, 0.29) |
| Southern Latin America | 1995-2000 | -0.22 (-0.30, -0.15)* |  | Southern Latin America | 1990-2006 | -0.06 (-0.09, -0.04)* |  | Southern Latin America | 1990-2006 | -0.05 (-0.08, -0.03)* |
| Southern Latin America | 2000-2005 | 0.20 (0.12, 0.28)* |  | Southern Latin America | 2006-2009 | -2.75 (-3.46, -2.03)* |  | Southern Latin America | 2006-2009 | -2.66 (-3.32, -2.01)* |
| Southern Latin America | 2005-2010 | -1.91 (-1.99, -1.83)* |  | Southern Latin America | 2009-2017 | 0.03 (-0.06, 0.13) |  | Southern Latin America | 2009-2017 | 0.03 (-0.06, 0.12) |
| Southern Latin America | 2010-2015 | 0.25 (0.17, 0.33)* |  | Southern Latin America | 2017-2019 | -0.52 (-1.23, 0.20) |  | Southern Latin America | 2017-2019 | -0.54 (-1.21, 0.14) |
| Southern Latin America | 2015-2019 | -0.29 (-0.37, -0.22)* |  | Western Europe | 1990-1995 | -0.74 (-0.80, -0.67)* |  | Western Europe | 1990-1995 | -0.72 (-0.79, -0.66)* |
| Western Europe | 1990-1995 | -0.73 (-0.80, -0.66)* |  | Western Europe | 1995-2000 | 0.50 (0.40, 0.59)* |  | Western Europe | 1995-2000 | 0.50 (0.41, 0.60)* |
| Western Europe | 1995-2000 | 0.47 (0.38, 0.56)* |  | Western Europe | 2000-2006 | -0.54 (-0.61, -0.47)* |  | Western Europe | 2000-2006 | -0.53 (-0.59, -0.46)* |
| Western Europe | 2000-2006 | -0.54 (-0.60, -0.47)* |  | Western Europe | 2006-2010 | 0.34 (0.18, 0.50)* |  | Western Europe | 2006-2010 | 0.33 (0.18, 0.48)* |
| Western Europe | 2006-2010 | 0.33 (0.18, 0.48)* |  | Western Europe | 2010-2015 | -0.28 (-0.39, -0.18)* |  | Western Europe | 2010-2015 | -0.27 (-0.37, -0.18)* |
| Western Europe | 2010-2015 | -0.27 (-0.37, -0.18)* |  | Western Europe | 2015-2019 | 0.09 (-0.02, 0.21) |  | Western Europe | 2015-2019 | 0.08 (-0.02, 0.17) |
| Western Europe | 2015-2019 | 0.09 (-0.02, 0.20) |  | Andean Latin America | 1990-1997 | -0.07 (-0.09, -0.05)* |  | Andean Latin America | 1990-1997 | -0.07 (-0.09, -0.05)* |
| Andean Latin America | 1990-1997 | -0.07 (-0.09, -0.05)* |  | Andean Latin America | 1997-2005 | -0.24 (-0.26, -0.23)* |  | Andean Latin America | 1997-2005 | -0.23 (-0.25, -0.21)* |
| Andean Latin America | 1997-2005 | -0.23 (-0.25, -0.22)* |  | Andean Latin America | 2005-2009 | -1.18 (-1.24, -1.12)* |  | Andean Latin America | 2005-2009 | -1.18 (-1.24, -1.12)* |
| Andean Latin America | 2005-2009 | -1.15 (-1.21, -1.10)* |  | Andean Latin America | 2009-2012 | -0.07 (-0.18, 0.05) |  | Andean Latin America | 2009-2012 | -0.05 (-0.17, 0.07) |
| Andean Latin America | 2009-2012 | -0.07 (-0.19, 0.04) |  | Andean Latin America | 2012-2019 | 0.13 (0.12, 0.15)* |  | Andean Latin America | 2012-2019 | 0.13 (0.11, 0.15)* |
| Andean Latin America | 2012-2019 | 0.13 (0.12, 0.15)* |  | Caribbean | 1990-2001 | -0.60 (-0.61, -0.58)* |  | Caribbean | 1990-2001 | -0.62 (-0.63, -0.60)* |
| Caribbean | 1990-2001 | -0.57 (-0.59, -0.55)* |  | Caribbean | 2001-2005 | -0.74 (-0.85, -0.62)* |  | Caribbean | 2001-2005 | -0.72 (-0.83, -0.61)* |
| Caribbean | 2001-2005 | -0.69 (-0.83, -0.56)* |  | Caribbean | 2005-2009 | -1.09 (-1.20, -0.98)* |  | Caribbean | 2005-2009 | -1.10 (-1.21, -0.99)* |
| Caribbean | 2005-2009 | -1.06 (-1.19, -0.93)* |  | Caribbean | 2009-2012 | -0.13 (-0.36, 0.10) |  | Caribbean | 2009-2012 | -0.17 (-0.39, 0.05) |
| Caribbean | 2009-2012 | -0.12 (-0.40, 0.15) |  | Caribbean | 2012-2017 | 0.08 (0.00, 0.15)* |  | Caribbean | 2012-2017 | 0.06 (-0.01, 0.14) |
| Caribbean | 2012-2017 | 0.08 (-0.01, 0.17) |  | Caribbean | 2017-2019 | 0.60 (0.35, 0.85)* |  | Caribbean | 2017-2019 | 0.60 (0.37, 0.83)* |
| Caribbean | 2017-2019 | 0.58 (0.29, 0.88)* |  | Central Latin America | 1990-1996 | 0.49 (0.47, 0.50)* |  | Central Latin America | 1990-1996 | 0.51 (0.48, 0.53)* |
| Central Latin America | 1990-1996 | 0.49 (0.47, 0.51)* |  | Central Latin America | 1996-1999 | 0.78 (0.68, 0.89)* |  | Central Latin America | 1996-1999 | 0.79 (0.66, 0.91)* |
| Central Latin America | 1996-1999 | 0.77 (0.66, 0.87)* |  | Central Latin America | 1999-2006 | 0.32 (0.30, 0.34)* |  | Central Latin America | 1999-2006 | 0.33 (0.31, 0.35)* |
| Central Latin America | 1999-2006 | 0.32 (0.30, 0.34)* |  | Central Latin America | 2006-2010 | 0.52 (0.47, 0.57)* |  | Central Latin America | 2006-2010 | 0.52 (0.46, 0.59)* |
| Central Latin America | 2006-2010 | 0.52 (0.47, 0.57)* |  | Central Latin America | 2010-2015 | -0.03 (-0.06, 0.00) |  | Central Latin America | 2010-2019 | 0.03 (0.01, 0.04)* |
| Central Latin America | 2010-2015 | -0.05 (-0.08, -0.01)* |  | Central Latin America | 2015-2019 | 0.07 (0.04, 0.10)* |  | Tropical Latin America | 1990-1995 | -0.19 (-0.37, -0.02)* |
| Central Latin America | 2015-2019 | 0.07 (0.04, 0.10)* |  | Tropical Latin America | 1990-1995 | -0.25 (-0.47, -0.03)* |  | Tropical Latin America | 1995-2000 | 3.13 (2.88, 3.39)* |
| Tropical Latin America | 1990-1995 | -0.24 (-0.46, -0.02)* |  | Tropical Latin America | 1995-2000 | 3.12 (2.81, 3.42)* |  | Tropical Latin America | 2000-2006 | 0.23 (0.06, 0.41)* |
| Tropical Latin America | 1995-2000 | 3.10 (2.79, 3.41)* |  | Tropical Latin America | 2000-2006 | 0.22 (0.01, 0.42)* |  | Tropical Latin America | 2006-2009 | -4.59 (-5.30, -3.86)* |
| Tropical Latin America | 2000-2006 | 0.21 (0.00, 0.42)* |  | Tropical Latin America | 2006-2009 | -4.58 (-5.36, -3.81)* |  | Tropical Latin America | 2009-2013 | -1.39 (-1.76, -1.02)* |
| Tropical Latin America | 2006-2009 | -4.58 (-5.35, -3.80)* |  | Tropical Latin America | 2009-2012 | -1.61 (-2.31, -0.91)* |  | Tropical Latin America | 2013-2019 | -0.40 (-0.52, -0.27)* |
| Tropical Latin America | 2009-2012 | -1.63 (-2.37, -0.89)* |  | Tropical Latin America | 2012-2019 | -0.51 (-0.62, -0.40)* |  | North Africa and Middle East | 1990-1996 | -0.15 (-0.21, -0.10)* |
| Tropical Latin America | 2012-2019 | -0.50 (-0.61, -0.40)* |  | North Africa and Middle East | 1990-1996 | -0.15 (-0.21, -0.10)* |  | North Africa and Middle East | 1996-2000 | -0.28 (-0.44, -0.12)* |
| North Africa and Middle East | 1990-1996 | -0.15 (-0.20, -0.10)* |  | North Africa and Middle East | 1996-2000 | -0.29 (-0.42, -0.15)* |  | North Africa and Middle East | 2000-2005 | 0.36 (0.26, 0.46)* |
| North Africa and Middle East | 1996-2000 | -0.28 (-0.42, -0.14)* |  | North Africa and Middle East | 2000-2005 | 0.36 (0.28, 0.44)* |  | North Africa and Middle East | 2005-2011 | -0.05 (-0.12, 0.02) |
| North Africa and Middle East | 2000-2005 | 0.35 (0.27, 0.44)* |  | North Africa and Middle East | 2005-2011 | -0.06 (-0.12, 0.00) |  | North Africa and Middle East | 2011-2014 | 0.70 (0.38, 1.03)* |
| North Africa and Middle East | 2005-2011 | -0.04 (-0.10, 0.01) |  | North Africa and Middle East | 2011-2014 | 0.70 (0.41, 1.00)* |  | North Africa and Middle East | 2014-2019 | -0.22 (-0.29, -0.15)* |
| North Africa and Middle East | 2011-2014 | 0.67 (0.38, 0.96)* |  | North Africa and Middle East | 2014-2019 | -0.21 (-0.28, -0.14)* |  | South Asia | 1990-1994 | 2.97 (2.74, 3.20)* |
| North Africa and Middle East | 2014-2019 | -0.21 (-0.28, -0.14)* |  | South Asia | 1990-1994 | 2.92 (2.68, 3.15)* |  | South Asia | 1994-2000 | -0.50 (-0.66, -0.34)* |
| South Asia | 1990-1994 | 2.89 (2.66, 3.12)* |  | South Asia | 1994-2000 | -0.51 (-0.67, -0.35)* |  | South Asia | 2000-2005 | 0.85 (0.62, 1.08)* |
| South Asia | 1994-2000 | -0.50 (-0.66, -0.34)* |  | South Asia | 2000-2005 | 0.83 (0.60, 1.06)* |  | South Asia | 2005-2010 | -4.76 (-4.98, -4.55)* |
| South Asia | 2000-2005 | 0.85 (0.62, 1.07)* |  | South Asia | 2005-2010 | -4.74 (-4.94, -4.53)* |  | South Asia | 2010-2019 | -0.03 (-0.10, 0.03) |
| South Asia | 2005-2010 | -4.74 (-4.94, -4.53)* |  | South Asia | 2010-2019 | -0.04 (-0.10, 0.02) |  | East Asia | 1990-1995 | 1.56 (1.24, 1.88)* |
| South Asia | 2010-2019 | -0.04 (-0.10, 0.02) |  | East Asia | 1990-1995 | 1.52 (1.19, 1.84)* |  | East Asia | 1995-2000 | -3.24 (-3.67, -2.81)* |
| East Asia | 1990-1995 | 1.52 (1.20, 1.85)* |  | East Asia | 1995-2000 | -3.21 (-3.61, -2.81)* |  | East Asia | 2000-2005 | -0.59 (-1.03, -0.15)* |
| East Asia | 1995-2000 | -3.21 (-3.62, -2.81)* |  | East Asia | 2000-2005 | -0.57 (-0.96, -0.18)* |  | East Asia | 2005-2010 | -1.93 (-2.36, -1.49)* |
| East Asia | 2000-2005 | -0.55 (-0.95, -0.14)* |  | East Asia | 2005-2010 | -1.87 (-2.24, -1.50)* |  | East Asia | 2010-2015 | 1.24 (0.79, 1.69)* |
| East Asia | 2005-2010 | -1.83 (-2.22, -1.44)* |  | East Asia | 2010-2015 | 1.29 (0.88, 1.70)* |  | East Asia | 2015-2019 | -0.48 (-0.92, -0.04)* |
| East Asia | 2010-2015 | 1.29 (0.89, 1.70)* |  | East Asia | 2015-2019 | -0.46 (-0.90, -0.03)* |  | Oceania | 1990-1996 | -0.05 (-0.07, -0.04)* |
| East Asia | 2015-2019 | -0.46 (-0.88, -0.03)* |  | Oceania | 1990-1995 | 0.01 (-0.00, 0.03) |  | Oceania | 1996-1999 | -0.39 (-0.48, -0.30)* |
| Oceania | 1990-1995 | 0.02 (0.00, 0.04)* |  | Oceania | 1995-2000 | -0.32 (-0.34, -0.30)* |  | Oceania | 1999-2006 | -0.15 (-0.16, -0.13)* |
| Oceania | 1995-2000 | -0.32 (-0.34, -0.30)* |  | Oceania | 2000-2006 | -0.15 (-0.16, -0.14)* |  | Oceania | 2006-2009 | -0.37 (-0.46, -0.28)* |
| Oceania | 2000-2006 | -0.14 (-0.16, -0.13)* |  | Oceania | 2006-2009 | -0.32 (-0.39, -0.26)* |  | Oceania | 2009-2019 | -0.07 (-0.08, -0.06)* |
| Oceania | 2006-2009 | -0.32 (-0.39, -0.26)* |  | Oceania | 2009-2016 | -0.09 (-0.10, -0.08)* |  | Southeast Asia | 1990-2000 | -0.58 (-0.61, -0.56)* |
| Oceania | 2009-2017 | -0.09 (-0.10, -0.08)* |  | Oceania | 2016-2019 | 0.01 (-0.02, 0.04) |  | Southeast Asia | 2000-2005 | -0.01 (-0.12, 0.09) |
| Oceania | 2017-2019 | 0.06 (-0.01, 0.12) |  | Southeast Asia | 1990-2000 | -0.59 (-0.62, -0.57)* |  | Southeast Asia | 2005-2010 | -0.52 (-0.62, -0.41)* |
| Southeast Asia | 1990-2000 | -0.58 (-0.61, -0.56)* |  | Southeast Asia | 2000-2005 | -0.06 (-0.16, 0.04) |  | Southeast Asia | 2010-2014 | 0.53 (0.36, 0.69)* |
| Southeast Asia | 2000-2005 | -0.06 (-0.16, 0.04) |  | Southeast Asia | 2005-2010 | -0.53 (-0.63, -0.43)* |  | Southeast Asia | 2014-2017 | -0.00 (-0.34, 0.33) |
| Southeast Asia | 2005-2010 | -0.51 (-0.61, -0.42)* |  | Southeast Asia | 2010-2014 | 0.51 (0.36, 0.67)* |  | Southeast Asia | 2017-2019 | 0.88 (0.54, 1.22)* |
| Southeast Asia | 2010-2014 | 0.51 (0.35, 0.66)* |  | Southeast Asia | 2014-2017 | -0.01 (-0.33, 0.32) |  | Central Sub-Saharan Africa | 1990-1997 | -0.12 (-0.13, -0.11)* |
| Southeast Asia | 2014-2017 | -0.02 (-0.34, 0.30) |  | Southeast Asia | 2017-2019 | 0.91 (0.58, 1.25)* |  | Central Sub-Saharan Africa | 1997-2001 | -0.19 (-0.22, -0.16)* |
| Southeast Asia | 2017-2019 | 0.92 (0.59, 1.25)* |  | Central Sub-Saharan Africa | 1990-1996 | -0.11 (-0.13, -0.09)* |  | Central Sub-Saharan Africa | 2001-2010 | -0.25 (-0.26, -0.24)* |
| Central Sub-Saharan Africa | 1990-1997 | -0.11 (-0.13, -0.10)* |  | Central Sub-Saharan Africa | 1996-2002 | -0.19 (-0.21, -0.16)* |  | Central Sub-Saharan Africa | 2010-2014 | 0.09 (0.06, 0.13)* |
| Central Sub-Saharan Africa | 1997-2002 | -0.19 (-0.22, -0.15)* |  | Central Sub-Saharan Africa | 2002-2010 | -0.32 (-0.33, -0.30)* |  | Central Sub-Saharan Africa | 2014-2017 | -0.03 (-0.10, 0.03) |
| Central Sub-Saharan Africa | 2002-2010 | -0.31 (-0.32, -0.29)* |  | Central Sub-Saharan Africa | 2010-2014 | 0.04 (-0.01, 0.10) |  | Central Sub-Saharan Africa | 2017-2019 | 0.20 (0.14, 0.27)* |
| Central Sub-Saharan Africa | 2010-2014 | 0.05 (-0.00, 0.11) |  | Central Sub-Saharan Africa | 2014-2017 | -0.04 (-0.15, 0.06) |  | Eastern Sub-Saharan Africa | 1990-1995 | 0.07 (0.03, 0.11)* |
| Central Sub-Saharan Africa | 2014-2017 | -0.06 (-0.17, 0.05) |  | Central Sub-Saharan Africa | 2017-2019 | 0.17 (0.06, 0.27)* |  | Eastern Sub-Saharan Africa | 1995-2000 | 0.33 (0.28, 0.38)* |
| Central Sub-Saharan Africa | 2017-2019 | 0.19 (0.08, 0.30)* |  | Eastern Sub-Saharan Africa | 1990-1994 | 0.04 (-0.00, 0.10) |  | Eastern Sub-Saharan Africa | 2000-2006 | -0.45 (-0.48, -0.41)* |
| Eastern Sub-Saharan Africa | 1990-1994 | 0.03 (-0.01, 0.08) |  | Eastern Sub-Saharan Africa | 1994-2000 | 0.32 (0.28, 0.35)* |  | Eastern Sub-Saharan Africa | 2006-2009 | -0.94 (-1.09, -0.78)* |
| Eastern Sub-Saharan Africa | 1994-2000 | 0.32 (0.29, 0.35)* |  | Eastern Sub-Saharan Africa | 2000-2006 | -0.49 (-0.52, -0.46)* |  | Eastern Sub-Saharan Africa | 2009-2014 | -0.67 (-0.72, -0.62)* |
| Eastern Sub-Saharan Africa | 2000-2006 | -0.48 (-0.51, -0.45)* |  | Eastern Sub-Saharan Africa | 2006-2009 | -1.00 (-1.15, -0.86)* |  | Eastern Sub-Saharan Africa | 2014-2019 | 0.13 (0.10, 0.17)* |
| Eastern Sub-Saharan Africa | 2006-2009 | -0.98 (-1.12, -0.84)* |  | Eastern Sub-Saharan Africa | 2009-2014 | -0.71 (-0.76, -0.67)* |  | Southern Sub-Saharan Africa | 1990-1994 | -1.16 (-1.24, -1.09)* |
| Eastern Sub-Saharan Africa | 2009-2014 | -0.70 (-0.75, -0.66)* |  | Eastern Sub-Saharan Africa | 2014-2019 | 0.12 (0.08, 0.15)* |  | Southern Sub-Saharan Africa | 1994-2001 | -0.31 (-0.35, -0.27)* |
| Eastern Sub-Saharan Africa | 2014-2019 | 0.12 (0.08, 0.15)* |  | Southern Sub-Saharan Africa | 1990-1994 | -1.12 (-1.19, -1.05)* |  | Southern Sub-Saharan Africa | 2001-2005 | -0.49 (-0.60, -0.38)* |
| Southern Sub-Saharan Africa | 1990-1994 | -1.10 (-1.16, -1.04)* |  | Southern Sub-Saharan Africa | 1994-2001 | -0.21 (-0.24, -0.17)* |  | Southern Sub-Saharan Africa | 2005-2014 | 0.73 (0.71, 0.76)* |
| Southern Sub-Saharan Africa | 1994-2000 | -0.19 (-0.24, -0.15)* |  | Southern Sub-Saharan Africa | 2001-2005 | -0.46 (-0.56, -0.36)* |  | Southern Sub-Saharan Africa | 2014-2017 | 1.39 (1.16, 1.61)* |
| Southern Sub-Saharan Africa | 2000-2005 | -0.41 (-0.47, -0.34)* |  | Southern Sub-Saharan Africa | 2005-2014 | 0.70 (0.67, 0.72)* |  | Southern Sub-Saharan Africa | 2017-2019 | -0.62 (-0.84, -0.41)* |
| Southern Sub-Saharan Africa | 2005-2014 | 0.68 (0.66, 0.71)* |  | Southern Sub-Saharan Africa | 2014-2017 | 1.42 (1.21, 1.63)* |  | Western Sub-Saharan Africa | 1990-1995 | -0.23 (-0.30, -0.17)* |
| Southern Sub-Saharan Africa | 2014-2017 | 1.40 (1.20, 1.61)* |  | Southern Sub-Saharan Africa | 2017-2019 | -0.62 (-0.84, -0.41)* |  | Western Sub-Saharan Africa | 1995-2000 | 1.77 (1.68, 1.87)* |
| Southern Sub-Saharan Africa | 2017-2019 | -0.63 (-0.83, -0.44)* |  | Western Sub-Saharan Africa | 1990-1995 | -0.23 (-0.30, -0.17)* |  | Western Sub-Saharan Africa | 2000-2006 | 0.05 (-0.01, 0.12) |
| Western Sub-Saharan Africa | 1990-1995 | -0.23 (-0.30, -0.17)* |  | Western Sub-Saharan Africa | 1995-2000 | 1.76 (1.67, 1.86)* |  | Western Sub-Saharan Africa | 2006-2014 | -1.71 (-1.75, -1.68)* |
| Western Sub-Saharan Africa | 1995-2000 | 1.75 (1.65, 1.84)* |  | Western Sub-Saharan Africa | 2000-2006 | 0.01 (-0.06, 0.08) |  | Western Sub-Saharan Africa | 2014-2019 | -0.05 (-0.12, 0.02) |
| Western Sub-Saharan Africa | 2000-2006 | 0.01 (-0.05, 0.08) |  | Western Sub-Saharan Africa | 2006-2014 | -1.71 (-1.75, -1.67)* |  |  |  |  |
| Western Sub-Saharan Africa | 2006-2014 | -1.70 (-1.73, -1.66)* |  | Western Sub-Saharan Africa | 2014-2019 | -0.06 (-0.13, 0.01) |  |  |  |  |
| Western Sub-Saharan Africa | 2014-2019 | -0.06 (-0.13, 0.00) |  |  |  |  |  |  |  |  |

| Table S4 Statistical parameters for overall and age-specific annual percentage changes in age-period-cohort model. | | | | |
| --- | --- | --- | --- | --- |
| Gender | Net drift | *P* value | | |
|  | (% per year; 95% CI) | All local drifts = net drift | All cohort deviations = 0 | all period deviations = 0 |
| Both | -0.23 (-0.33, -0.14) | <0.01 | <0.01 | <0.01 |
| Male | -0.16 (-0.24, -0.08) | <0.01 | <0.01 | <0.01 |
| Female | -0.27 (-0.39, -0.16) | <0.01 | <0.01 | <0.01 |

| Table S5 The AAPC of ASIR, ASPR and ASDR during 1990-2019 by gender, SDI regions, and GBD regions. | | | |
| --- | --- | --- | --- |
| Characteristics | AAPC during 1990-2019 (95% CI) | | |
|  | ASIR | ASPR | ASDR |
| **Gender** |  |  |  |
| Male | 0.00 (-0.03, 0.04) | -0.01 (-0.04, 0.03) | -0.01 (-0.04, 0.03) |
| Female | -0.17 (-0.25, -0.09)* | -0.18 (-0.25, -0.11)* | -0.18 (-0.25, -0.11)* |
| **SDI regions** |  |  |  |
| High SDI | 0.35 (0.40, 0.32)* | 0.34 (0.38, 0.30)* | 0.34 (0.38, 0.30)* |
| High-middle SDI | -0.34 (-0.30, -0.38)* | -0.34 (-0.30, -0.39)* | -0.34 (-0.30, -0.39)* |
| Middle SDI | -0.03 (0.02, -0.09) | -0.04 (0.02, -0.09) | -0.04 (0.02, -0.09) |
| Low-middle SDI | -0.30 (-0.25, -0.35)* | -0.30 (-0.26, -0.35)* | -0.30 (-0.26, -0.35)* |
| Low SDI | -0.21 (-0.15, -0.26)* | -0.21 (-0.15, -0.26)* | -0.21 (-0.15, -0.26)* |
| **GBD regions** |  |  |  |
| Central Asia | -0.18 (-0.17, -0.19)* | -0.18 (-0.17, -0.19)* | -0.18 (-0.17, -0.19)* |
| Central Europe | -0.47 (-0.43, -0.52)* | -0.49 (-0.44, -0.53)* | -0.49 (-0.44, -0.53)* |
| Eastern Europe | -0.39 (-0.33, -0.44)* | -0.39 (-0.34, -0.43)* | -0.39 (-0.34, -0.43)* |
| Australasia | 0.03 (0.08, -0.02) | 0.03 (0.09, -0.03) | 0.03 (0.09, -0.03) |
| High-income Asia Pacific | 0.18 (0.26, 0.10)* | 0.18 (0.26, 0.10)* | 0.18 (0.26, 0.10)* |
| High-income North America | 0.92 (1.07, 0.76)* | 0.89 (1.06, 0.71)* | 0.89 (1.06, 0.71)* |
| Southern Latin America | -0.33 (-0.30, -0.36)* | -0.35 (-0.26, -0.44)* | -0.35 (-0.26, -0.44)* |
| Western Europe | -0.14 (-0.11, -0.18)* | -0.14 (-0.11, -0.18)* | -0.14 (-0.11, -0.18)* |
| Andean Latin America | -0.22 (-0.20, -0.23)* | -0.22 (-0.21, -0.24)* | -0.22 (-0.21, -0.24)* |
| Caribbean | -0.42 (-0.38, -0.46)* | -0.44 (-0.40, -0.47)* | -0.44 (-0.40, -0.47)* |
| Central Latin America | 0.33 (0.35, 0.32)* | 0.33 (0.35, 0.32)* | 0.33 (0.35, 0.32)* |
| Tropical Latin America | -0.25 (-0.12, -0.38)* | -0.25 (-0.12, -0.37)* | -0.25 (-0.12, -0.37)* |
| North Africa and Middle East | 0.02 (0.06, -0.02) | 0.02 (0.06, -0.02) | 0.02 (0.06, -0.02) |
| South Asia | -0.41 (-0.35, -0.48)* | -0.41 (-0.35, -0.48)* | -0.41 (-0.35, -0.48)* |
| East Asia | -0.56 (-0.41, -0.70)* | -0.57 (-0.42, -0.71)* | -0.57 (-0.42, -0.71)* |
| Oceania | -0.13 (-0.12, -0.14)* | -0.14 (-0.13, -0.15)* | -0.14 (-0.13, -0.15)* |
| Southeast Asia | -0.17 (-0.12, -0.22)* | -0.17 (-0.12, -0.22)* | -0.17 (-0.12, -0.22)* |
| Central Sub-Saharan Africa | -0.13 (-0.12, -0.15)* | -0.14 (-0.12, -0.15)* | -0.14 (-0.12, -0.15)* |
| Eastern Sub-Saharan Africa | -0.23 (-0.21, -0.25)* | -0.24 (-0.22, -0.26)* | -0.24 (-0.22, -0.26)* |
| Southern Sub-Saharan Africa | 0.05 (0.08, 0.02)* | 0.05 (0.08, 0.02)* | 0.05 (0.08, 0.02)* |
| Western Sub-Saharan Africa | -0.22 (-0.20, -0.25)* | -0.22 (-0.20, -0.25)* | -0.22 (-0.20, -0.25)* |

| Table S6 The number and ASPR of MDD in 1990 and 2019 by gender, SDI regions and GBD regions, and their percentage change. | | | | | | |
| --- | --- | --- | --- | --- | --- | --- |
| Characteristics | Number × 106 (95% UI) | | Percentage change of number (%, 95% UI) | ASPR per 1000 population  (95% UI) | | Percentage change of |
|  |  |  |  |  |  | ASPR (%, 95% UI) |
|  | 1990 year | 2019 year | during 1990-2019 | 1990 year | 2019 year | during 1990-2019 |
| **Global** | 116.03 (131.81, 101.84) | 185.15 (210.15, 162.59) | 59.57 (64.02, 54.64) | 2.36 (2.08, 2.65) | 2.29 (2.01, 2.59) | -3.02 (-1.86, -4.23) |
| **Gender** |  |  |  |  |  |  |
| Male | 42.36 (48.2, 37.04) | 69.76 (79.11, 61.17) | 64.66 (68.94, 59.81) | 1.74 (1.96, 1.53) | 1.74 (1.97, 1.53) | 0.26 (1.32, -0.82) |
| Female | 73.67 (83.55, 64.67) | 115.4 (131.28, 101.37) | 56.64 (61.47, 51.61) | 2.97 (3.34, 2.61) | 2.82 (3.21, 2.47) | -4.88 (-3.46, -6.37) |
| **SDI regions** |  |  |  |  |  |  |
| High SDI | 21.14 (23.46, 18.96) | 29.18 (32.72, 25.94) | 38.00 (41.65, 34.05) | 2.35 (2.10, 2.62) | 2.59 (2.28, 2.94) | 10.34 (13.17, 7.25) |
| High-middle SDI | 25.93 (29.08, 23.02) | 34.27 (38.47, 30.42) | 32.19 (37.10, 27.05) | 2.22 (1.98, 2.49) | 2.02 (1.79, 2.28) | -9.22 (-7.48, -11.04) |
| Middle SDI | 30.81 (35.31, 26.60) | 51.01 (57.93, 44.67) | 65.54 (74.52, 56.58) | 2.00 (1.75, 2.27) | 1.98 (1.74, 2.25) | -0.86 (0.85, -2.68) |
| Low-middle SDI | 26.06 (30.11, 22.37) | 44.84 (51.22, 38.99) | 72.04 (77.59, 66.31) | 2.92 (2.55, 3.34) | 2.69 (2.36, 3.06) | -8.00 (-6.04, -10.20) |
| Low SDI | 12.02 (13.99, 10.31) | 25.75 (29.95, 22.01) | 114.21 (117.34, 111.15) | 3.25 (2.83, 3.73) | 3.07 (2.67, 3.52) | -5.68 (-4.31, -6.93) |
| **GBD regions** |  |  |  |  |  |  |
| Central Asia | 1.30 (1.50, 1.11) | 1.88 (2.21, 1.62) | 45.12 (51.62, 38.67) | 2.23 (1.92, 2.56) | 2.11 (1.83, 2.45) | -5.13 (-1.42, -8.91) |
| Central Europe | 2.34 (2.66, 2.06) | 2.25 (2.56, 1.97) | -3.90 (0.85, -8.28) | 1.74 (1.53, 1.97) | 1.51 (1.32, 1.74) | -13.02 (-9.79, -16.17) |
| Eastern Europe | 6.38 (7.31, 5.54) | 5.90 (6.74, 5.14) | -7.60 (-5.22, -9.83) | 2.52 (2.19, 2.89) | 2.26 (1.96, 2.60) | -10.42 (-8.66, -12.00) |
| Australasia | 0.71 (0.81, 0.63) | 1.02 (1.18, 0.87) | 43.12 (55.14, 31.90) | 3.30 (2.90, 3.74) | 3.33 (2.84, 3.90) | 1.07 (8.98, -6.23) |
| High-income Asia Pacific | 2.71 (3.04, 2.41) | 3.38 (3.74, 3.03) | 24.45 (30.66, 18.64) | 1.42 (1.26, 1.59) | 1.49 (1.33, 1.67) | 5.25 (7.70, 3.04) |
| High-income North America | 7.35 (8.32, 6.51) | 12.11 (13.57, 10.80) | 64.77 (69.37, 60.80) | 2.44 (2.16, 2.77) | 3.18 (2.81, 3.62) | 30.18 (33.31, 26.81) |
| Southern Latin America | 1.16 (1.31, 1.02) | 1.55 (1.75, 1.36) | 33.76 (40.07, 27.77) | 2.39 (2.11, 2.69) | 2.16 (1.90, 2.45) | -9.54 (-5.34, -13.71) |
| Western Europe | 12.92 (14.19, 11.69) | 14.66 (16.52, 13.06) | 13.48 (18.25, 8.70) | 2.95 (2.66, 3.26) | 2.82 (2.49, 3.20) | -4.30 (-0.55, -7.94) |
| Andean Latin America | 0.61 (0.72, 0.52) | 1.15 (1.34, 0.99) | 87.77 (99.03, 75.85) | 1.96 (1.70, 2.29) | 1.84 (1.58, 2.14) | -6.25 (-2.24, -10.31) |
| Caribbean | 1.04 (1.21, 0.89) | 1.40 (1.63, 1.21) | 34.43 (42.81, 26.24) | 3.20 (2.76, 3.69) | 2.82 (2.43, 3.25) | -12.00 (-7.89, -16.30) |
| Central Latin America | 2.83 (3.27, 2.44) | 6.08 (6.95, 5.30) | 115.01 (124.78, 105.31) | 2.16 (1.89, 2.47) | 2.38 (2.08, 2.71) | 10.22 (12.22, 8.13) |
| Tropical Latin America | 4.33 (4.94, 3.76) | 7.15 (7.94, 6.40) | 65.14 (73.67, 56.24) | 3.18 (2.79, 3.60) | 2.97 (2.66, 3.30) | -6.57 (-2.29, -10.61) |
| North Africa and Middle East | 9.38 (11.04, 8.02) | 20.16 (23.84, 16.99) | 114.85 (125.47, 104.41) | 3.30 (2.86, 3.82) | 3.32 (2.84, 3.90) | 0.60 (2.90, -1.53) |
| South Asia | 25.78 (29.67, 22.21) | 45.94 (52.21, 40.08) | 78.19 (84.36, 71.46) | 3.01 (2.64, 3.44) | 2.68 (2.36, 3.03) | -10.77 (-8.57, -13.23) |
| East Asia | 19.99 (22.92, 17.22) | 26.09 (29.57, 23.05) | 30.48 (43.03, 18.48) | 1.64 (1.44, 1.85) | 1.41 (1.25, 1.59) | -14.12 (-10.08, -18.01) |
| Oceania | 0.10 (0.12, 0.08) | 0.20 (0.25, 0.17) | 107.45 (116.46, 97.73) | 1.77 (1.48, 2.08) | 1.70 (1.43, 2.02) | -3.90 (-0.43, -7.41) |
| Southeast Asia | 5.44 (6.37, 4.64) | 8.77 (10.12, 7.59) | 61.31 (69.68, 53.23) | 1.31 (1.14, 1.51) | 1.25 (1.09, 1.44) | -4.70 (-2.51, -6.55) |
| Central Sub-Saharan Africa | 1.80 (2.17, 1.49) | 4.31 (5.20, 3.54) | 139.26 (151.09, 128.82) | 4.51 (3.81, 5.33) | 4.34 (3.67, 5.08) | -3.85 (0.40, -7.96) |
| Eastern Sub-Saharan Africa | 4.66 (5.40, 3.99) | 10.10 (11.73, 8.62) | 116.62 (120.63, 112.24) | 3.77 (3.30, 4.31) | 3.52 (3.09, 4.04) | -6.46 (-4.75, -8.17) |
| Southern Sub-Saharan Africa | 1.19 (1.37, 1.03) | 2.13 (2.42, 1.85) | 78.58 (84.28, 72.40) | 2.89 (2.54, 3.27) | 2.92 (2.57, 3.28) | 1.12 (3.33, -1.00) |
| Western Sub-Saharan Africa | 3.99 (4.65, 3.43) | 8.91 (10.39, 7.63) | 123.30 (126.28, 120.30) | 3.01 (2.62, 3.44) | 2.82 (2.46, 3.22) | -6.40 (-5.01, -7.70) |

| TableS7 The number and ASDR of MDD in 1990 and 2019 by gender, SDI regions and GBD regions, and their percentage change. | | | | | | |
| --- | --- | --- | --- | --- | --- | --- |
|  | Number × 106 (95% UI) | | Percentage change of  number (%, 95% UI) | ASDR per 1000 population  (95% UI) | | Percentage change of  ASDR (%, 95% UI) |
| Characteristics |  |  |  |  |  |  |
|  | 1990 year | 2019 year | during 1990-2019 | 1990 year | 2019 year | during 1990-2019 |
| **Global** | 23.46 (32.50, 16.03) | 37.20 (51.22, 25.65) | 58.57 (62.96, 53.61) | 0.47 (0.33, 0.65) | 0.46 (0.32, 0.63) | -2.83 (-1.63, -4.06) |
| **Gender** |  |  |  |  |  |  |
| Male | 8.67 (12.03，5.9) | 14.18 (19.62，9.74) | 63.65 (68.04, 58.90) | 0.35 (0.49，0.24) | 0.35 (0.49，0.24) | 0.48 (1.62, -0.73) |
| Female | 14.79 (20.49，10.1) | 23.02 (31.73，15.81) | 55.60 (60.58, 50.58) | 0.59 (0.82，0.41) | 0.56 (0.79，0.39) | -4.75 (-3.17, -6.24) |
| **SDI regions** |  |  |  |  |  |  |
| High SDI | 4.25 (5.81, 2.96) | 5.82 (8.01, 4.02) | 36.92 (40.71, 32.86) | 0.47 (0.33, 0.65) | 0.52 (0.36, 0.72) | 10.57 (13.52, 7.48) |
| High-middle SDI | 5.24 (7.19, 3.60) | 6.86 (9.50, 4.73) | 31.07 (35.95, 25.60) | 0.45 (0.31, 0.61) | 0.41 (0.28, 0.56) | -8.91 (-7.02, -10.81) |
| Middle SDI | 6.30 (8.80, 4.25) | 10.28 (14.15, 7.09) | 63.24 (72.06, 54.47) | 0.40 (0.28, 0.56) | 0.40 (0.27, 0.55) | -1.08 (0.71, -2.92) |
| Low-middle SDI | 5.25 (7.32, 3.58) | 9.00 (12.49, 6.18) | 71.56 (77.22, 65.95) | 0.58 (0.40, 0.80) | 0.54 (0.37, 0.74) | -7.72 (-5.70, -9.93) |
| Low SDI | 2.42 (3.38, 1.63) | 5.22 (7.31, 3.50) | 115.78 (119.14, 112.39) | 0.64 (0.44, 0.90) | 0.61 (0.42, 0.85) | -5.07 (-3.67, -6.43) |
| **GBD regions** |  |  |  |  |  |  |
| Central Asia | 0.26 (0.37, 0.18) | 0.38 (0.54, 0.26) | 45.05 (51.46, 38.10) | 0.45 (0.30, 0.63) | 0.42 (0.29, 0.60) | -4.99 (-1.33, -8.65) |
| Central Europe | 0.47 (0.65, 0.32) | 0.44 (0.61, 0.31) | -4.93 (-0.09, -9.60) | 0.35 (0.24, 0.49) | 0.30 (0.21, 0.42) | -12.77 (-9.36, -16.06) |
| Eastern Europe | 1.27 (1.78, 0.87) | 1.17 (1.63, 0.80) | -7.74 (-5.31, -10.05) | 0.50 (0.34, 0.70) | 0.45 (0.31, 0.63) | -9.76 (-7.77, -11.48) |
| Australasia | 0.14 (0.20, 0.10) | 0.20 (0.29, 0.14) | 42.38 (54.35, 31.16) | 0.67 (0.46, 0.92) | 0.68 (0.46, 0.96) | 1.33 (9.47, -6.57) |
| High-income Asia Pacific | 0.55 (0.76, 0.38) | 0.68 (0.92, 0.47) | 22.50 (28.69, 16.44) | 0.29 (0.20, 0.40) | 0.31 (0.21, 0.42) | 5.82 (8.61, 3.22) |
| High-income North America | 1.47 (2.01, 1.02) | 2.41 (3.28, 1.66) | 63.41 (68.09, 59.45) | 0.49 (0.34, 0.68) | 0.64 (0.44, 0.87) | 30.14 (33.47, 26.71) |
| Southern Latin America | 0.23 (0.33, 0.16) | 0.31 (0.43, 0.21) | 33.38 (40.59, 27.06) | 0.48 (0.33, 0.67) | 0.44 (0.30, 0.61) | -9.26 (-4.44, -13.50) |
| Western Europe | 2.59 (3.51, 1.80) | 2.91 (3.98, 2.02) | 12.60 (17.19, 8.00) | 0.59 (0.41, 0.81) | 0.57 (0.39, 0.79) | -4.18 (-0.42, -7.86) |
| Andean Latin America | 0.13 (0.18, 0.08) | 0.23 (0.32, 0.16) | 86.42 (98.90, 73.73) | 0.40 (0.27, 0.55) | 0.37 (0.25, 0.51) | -6.15 (-1.60, -10.61) |
| Caribbean | 0.21 (0.30, 0.14) | 0.28 (0.39, 0.19) | 32.84 (41.60, 24.31) | 0.65 (0.44, 0.91) | 0.57 (0.38, 0.79) | -12.33 (-8.00, -16.82) |
| Central Latin America | 0.58 (0.81, 0.39) | 1.23 (1.71, 0.83) | 112.98 (122.94, 103.36) | 0.43 (0.30, 0.60) | 0.48 (0.33, 0.66) | 10.64 (12.91, 8.51) |
| Tropical Latin America | 0.88 (1.21, 0.60) | 1.44 (1.95, 1.00) | 64.33 (72.85, 55.55) | 0.64 (0.44, 0.88) | 0.60 (0.41, 0.82) | -5.83 (-1.29, -9.97) |
| North Africa and Middle East | 1.92 (2.72, 1.29) | 4.10 (5.75, 2.76) | 113.65 (124.28, 102.99) | 0.67 (0.46, 0.93) | 0.67 (0.46, 0.93) | 0.59 (2.87, -1.76) |
| South Asia | 5.16 (7.19, 3.52) | 9.16 (12.69, 6.29) | 77.60 (83.79, 70.91) | 0.59 (0.41, 0.82) | 0.53 (0.36, 0.73) | -10.52 (-8.27, -13.07) |
| East Asia | 4.11 (5.74, 2.81) | 5.24 (7.20, 3.62) | 27.42 (39.66, 15.70) | 0.33 (0.23, 0.46) | 0.28 (0.20, 0.39) | -14.74 (-10.65, -18.54) |
| Oceania | 0.02 (0.03, 0.01) | 0.04 (0.06, 0.03) | 106.65 (116.79, 95.94) | 0.36 (0.24, 0.50) | 0.34 (0.23, 0.49) | -4.12 (0.01, -8.59) |
| Southeast Asia | 1.12 (1.58, 0.75) | 1.79 (2.50, 1.21) | 60.36 (69.13, 52.26) | 0.27 (0.18, 0.37) | 0.25 (0.17, 0.36) | -4.36 (-2.02, -6.44) |
| Central Sub-Saharan Africa | 0.36 (0.52, 0.24) | 0.88 (1.26, 0.58) | 141.34 (153.62, 130.00) | 0.90 (0.61, 1.27) | 0.87 (0.58, 1.22) | -3.10 (1.38, -7.27) |
| Eastern Sub-Saharan Africa | 0.94 (1.32, 0.63) | 2.06 (2.89, 1.38) | 118.31 (123.01, 113.73) | 0.75 (0.51, 1.04) | 0.70 (0.48, 0.97) | -5.86 (-4.07, -7.74) |
| Southern Sub-Saharan Africa | 0.24 (0.34, 0.16) | 0.43 (0.59, 0.29) | 76.29 (82.29, 70.24) | 0.57 (0.39, 0.79) | 0.58 (0.40, 0.79) | 0.33 (2.78, -1.95) |
| Western Sub-Saharan Africa | 0.81 (1.13, 0.54) | 1.81 (2.54, 1.22) | 124.63 (128.02, 121.13) | 0.60 (0.41, 0.83) | 0.56 (0.38, 0.77) | -6.12 (-4.74, -7.47) |

| Table S8 The number and ASIR of MDD in 1990 and 2019, and the corresponding percentage change and AAPC of ASIR during 1990-2019 across 204 countries and territories. | | | | | | | |
| --- | --- | --- | --- | --- | --- | --- | --- |
|  |  |  | Percentage change of |  |  | Percentage change of | AAPC (95% CI) |
| Countries or territories | Number ×10^3^ (95% UI) | | number (%) (95% UI) | ASIR per 100 000 population (95% UI) | | ASIR (%, 95% UI) | of ASIR |
|  | 1990 year | 2019 year | during 1990-2019 | 1990 year | 2019 year | during 1990-2019 | during 1990-2019 |
| Afghanistan | 527.92 (443.35, 632.95) | 1732.44 (1428.85, 2081.45) | 228.16 (204.21, 254.43) | 5867.43 (4951.53, 6936.03) | 5808.71 (4892.33, 6847.55) | -1.00 (-6.71, 5.80) | -0.04 (-0.06, -0.02)* |
| Albania | 56.95 (47.86, 67.99) | 66.54 (56.75, 78.03) | 16.84 (4.57, 30.15) | 1928.22 (1654.65, 2270.67) | 2042.83 (1744.05, 2386.90) | 5.94 (-0.97, 13.66) | 0.20 (0.15, 0.25)* |
| Algeria | 983.48 (815.31, 1192.22) | 1932.05 (1612.39, 2309.67) | 96.45 (79.21, 115.02) | 4766.18 (4039.92, 5656.49) | 4586.78 (3869.57, 5434.81) | -3.76 (-9.30, 1.98) | -0.14 (-0.17, -0.10)* |
| American Samoa | 0.84 (0.69, 1.03) | 1.06 (0.88, 1.27) | 25.87 (15.94, 35.73) | 1974.09 (1669.57, 2331.17) | 1923.41 (1621.69, 2280.83) | -2.57 (-8.22, 3.64) | -0.09 (-0.13, -0.06)* |
| Andorra | 2.36 (1.99, 2.79) | 3.81 (3.20, 4.49) | 61.65 (48.18, 75.54) | 3887.68 (3303.45, 4605.79) | 3792.17 (3229.89, 4500.74) | -2.46 (-7.47, 3.15) | -0.09 (-0.12, -0.05)* |
| Angola | 538.07 (445.23, 653.63) | 1478.60 (1201.24, 1785.30) | 174.80 (156.06, 193.07) | 7072.38 (5988.55, 8414.30) | 6741.34 (5642.47, 7891.01) | -4.68 (-11.15, 1.24) | -0.16 (-0.17, -0.15)* |
| Antigua and Barbuda | 1.85 (1.54, 2.19) | 3.21 (2.68, 3.82) | 73.91 (61.62, 87.74) | 3208.12 (2683.90, 3789.86) | 3210.26 (2691.01, 3812.05) | 0.07 (-5.11, 5.23) | 0.00 (-0.09, 0.09) |
| Argentina | 916.98 (780.27, 1068.73) | 1282.08 (1140.21, 1432.27) | 39.82 (28.20, 52.83) | 2833.86 (2406.42, 3300.14) | 2695.62 (2392.70, 3025.66) | -4.88 (-13.10, 4.10) | -0.18 (-0.20, -0.15)* |
| Armenia | 83.03 (69.70, 97.80) | 100.95 (85.57, 118.21) | 21.58 (11.17, 33.25) | 2621.92 (2228.75, 3063.63) | 2845.59 (2422.04, 3325.51) | 8.53 (1.95, 15.90) | 0.29 (0.22, 0.36)* |
| Australia | 908.58 (792.22, 1040.47) | 1311.03 (1117.55, 1534.24) | 44.29 (31.83, 57.63) | 5073.88 (4432.60, 5830.87) | 5064.86 (4295.37, 5977.16) | -0.18 (-8.50, 8.80) | -0.02 (-0.06, 0.03) |
| Austria | 336.07 (287.46, 389.83) | 324.44 (280.48, 377.11) | -3.46 (-11.35, 5.31) | 3823.95 (3264.16, 4442.09) | 3068.16 (2628.51, 3556.12) | -19.76 (-26.01, -13.09) | -0.76 (-0.81, -0.72)* |
| Azerbaijan | 160.70 (134.09, 190.16) | 269.04 (226.24, 321.53) | 67.42 (54.73, 81.56) | 2514.43 (2140.08, 2961.75) | 2497.23 (2134.53, 2944.04) | -0.68 (-6.51, 5.45) | -0.04 (-0.14, 0.06) |
| Bahamas | 7.83 (6.53, 9.30) | 13.51 (11.36, 15.98) | 72.59 (59.11, 86.80) | 3313.15 (2819.84, 3923.67) | 3259.95 (2760.48, 3827.62) | -1.61 (-6.72, 4.31) | -0.06 (-0.13, 0.01) |
| Bahrain | 29.05 (23.90, 35.04) | 88.11 (72.59, 105.74) | 203.32 (165.50, 244.78) | 6000.38 (5109.89, 7020.27) | 5245.00 (4464.36, 6158.13) | -12.59 (-18.96, -6.19) | -0.47 (-0.49, -0.44)* |
| Bangladesh | 4392.92 (3660.77, 5207.70) | 8166.47 (6855.80, 9684.24) | 85.90 (68.91, 105.36) | 5452.83 (4609.77, 6445.36) | 5233.04 (4432.84, 6172.01) | -4.03 (-10.49, 3.69) | -0.14 (-0.15, -0.13)* |
| Barbados | 8.91 (7.63, 10.49) | 12.30 (10.42, 14.54) | 38.09 (26.88, 49.35) | 3383.42 (2899.24, 4002.12) | 3436.05 (2916.64, 4040.03) | 1.56 (-3.54, 7.01) | 0.05 (0.01, 0.10)* |
| Belarus | 496.90 (424.30, 578.13) | 496.67 (421.31, 574.81) | -0.05 (-9.38, 9.05) | 4267.09 (3636.62, 4953.83) | 4127.86 (3537.71, 4774.80) | -3.26 (-11.72, 4.26) | -0.10 (-0.18, -0.02)* |
| Belgium | 393.80 (359.84, 429.59) | 500.37 (433.98, 580.27) | 27.06 (14.63, 42.29) | 3473.67 (3166.49, 3804.74) | 3821.47 (3258.99, 4446.76) | 10.01 (-1.31, 22.67) | 0.33 (0.29, 0.36)* |
| Belize | 5.08 (4.23, 6.00) | 14.34 (12.14, 17.03) | 182.39 (159.76, 203.61) | 3484.91 (2974.11, 4051.15) | 3634.79 (3113.66, 4270.67) | 4.30 (-2.52, 10.46) | 0.14 (0.11, 0.17)* |
| Benin | 143.52 (119.82, 170.22) | 407.21 (338.83, 483.10) | 183.72 (167.76, 201.32) | 4587.93 (3923.76, 5393.07) | 4750.28 (4035.39, 5516.46) | 3.54 (-2.62, 10.28) | 0.12 (0.10, 0.14)* |
| Bermuda | 2.63 (2.24, 3.09) | 2.80 (2.39, 3.29) | 6.54 (-4.54, 18.70) | 4045.43 (3450.02, 4701.09) | 3439.39 (2936.36, 4036.50) | -14.98 (-21.50, -8.11) | -0.56 (-0.58, -0.54)* |
| Bhutan | 20.17 (16.89, 24.26) | 31.70 (26.57, 37.75) | 57.15 (45.22, 70.50) | 4711.29 (4032.27, 5586.76) | 4349.75 (3677.34, 5137.80) | -7.67 (-13.81, -1.19) | -0.28 (-0.32, -0.24)* |
| Bolivia (Plurinational State of) | 204.70 (171.74, 243.00) | 408.29 (343.11, 477.14) | 99.46 (86.31, 114.82) | 4148.31 (3520.18, 4885.70) | 3740.03 (3170.40, 4357.33) | -9.84 (-15.23, -3.18) | -0.36 (-0.38, -0.34)* |
| Bosnia and Herzegovina | 152.91 (129.98, 178.87) | 106.90 (90.27, 127.30) | -30.09 (-38.78, -20.77) | 3259.76 (2783.36, 3780.86) | 2495.20 (2123.71, 2947.96) | -23.45 (-30.72, -15.28) | -0.92 (-1.01, -0.82)* |
| Botswana | 40.36 (33.71, 48.01) | 94.68 (78.20, 114.41) | 134.59 (115.56, 155.61) | 4297.09 (3639.10, 5022.33) | 4446.78 (3789.25, 5235.78) | 3.48 (-3.11, 10.32) | 0.12 (0.10, 0.14)* |
| Brazil | 6329.69 (5471.17, 7263.69) | 10268.35 (9135.54, 11427.15) | 62.23 (53.35, 70.69) | 4740.16 (4144.19, 5372.99) | 4412.69 (3930.51, 4911.27) | -6.91 (-10.88, -2.37) | -0.25 (-0.36, -0.15)* |
| Brunei Darussalam | 3.36 (2.77, 4.10) | 6.64 (5.54, 8.02) | 97.46 (81.93, 113.63) | 1460.08 (1246.83, 1732.30) | 1463.64 (1236.03, 1746.07) | 0.24 (-5.70, 6.80) | 0.00 (-0.03, 0.03) |
| Bulgaria | 290.75 (249.80, 336.67) | 230.09 (194.81, 266.13) | -20.86 (-28.93, -11.25) | 2845.32 (2454.22, 3289.81) | 2396.63 (2032.65, 2806.67) | -15.77 (-23.28, -7.35) | -0.59 (-0.62, -0.56)* |
| Burkina Faso | 297.98 (251.82, 350.88) | 710.52 (591.53, 839.29) | 138.45 (124.41, 155.27) | 4753.64 (4076.47, 5525.51) | 4635.22 (3933.88, 5392.53) | -2.49 (-8.33, 4.37) | -0.05 (-0.14, 0.04) |
| Burundi | 232.89 (194.13, 276.31) | 409.79 (342.19, 489.61) | 75.96 (64.15, 87.81) | 6274.75 (5315.13, 7393.01) | 5016.45 (4285.94, 5938.93) | -20.05 (-25.82, -14.29) | -0.77 (-0.79, -0.76)* |
| Cabo Verde | 12.99 (10.99, 15.29) | 28.79 (24.34, 33.89) | 121.54 (103.92, 140.39) | 4948.40 (4200.74, 5785.15) | 5359.75 (4581.31, 6261.25) | 8.31 (1.48, 15.30) | 0.28 (0.25, 0.32)* |
| Cambodia | 225.34 (183.47, 273.98) | 375.41 (316.09, 454.23) | 66.60 (53.39, 81.72) | 2748.29 (2309.13, 3270.69) | 2329.05 (1977.58, 2778.43) | -15.25 (-20.98, -8.82) | -0.57 (-0.60, -0.55)* |
| Cameroon | 340.66 (284.27, 405.58) | 1056.31 (881.15, 1246.23) | 210.08 (191.38, 232.46) | 4810.51 (4096.03, 5636.18) | 4995.38 (4298.20, 5815.84) | 3.84 (-2.58, 10.28) | 0.13 (0.12, 0.15)* |
| Canada | 936.54 (820.88, 1065.76) | 1183.39 (1028.23, 1357.61) | 26.36 (18.29, 35.79) | 3186.34 (2796.34, 3632.88) | 3179.94 (2708.98, 3696.57) | -0.20 (-7.19, 7.86) | -0.02 (-0.12, 0.08) |
| Central African Republic | 148.10 (120.47, 179.14) | 291.65 (239.85, 351.24) | 96.93 (85.92, 108.82) | 7140.62 (5962.41, 8398.24) | 7021.03 (5909.85, 8262.08) | -1.67 (-7.04, 4.29) | -0.06 (-0.07, -0.05)* |
| Chad | 226.70 (190.69, 267.72) | 587.52 (489.88, 699.47) | 159.17 (142.64, 178.20) | 5628.06 (4826.34, 6571.79) | 5793.53 (4938.83, 6772.33) | 2.94 (-3.54, 10.21) | 0.10 (0.07, 0.14)* |
| Chile | 701.48 (632.27, 774.75) | 876.31 (747.76, 1018.28) | 24.92 (12.67, 40.33) | 5454.35 (4916.49, 6005.59) | 4393.06 (3752.80, 5110.37) | -19.46 (-27.72, -10.16) | -0.72 (-0.82, -0.62)* |
| China | 28952.25 (24864.44, 33299.11) | 37511.59 (33047.66, 42360.85) | 29.56 (17.18, 42.93) | 2445.16 (2135.04, 2774.70) | 2102.12 (1854.73, 2368.37) | -14.03 (-17.99, -9.78) | -0.57 (-0.71, -0.42)* |
| Colombia | 676.10 (564.88, 802.57) | 1089.69 (949.17, 1230.09) | 61.17 (43.20, 78.83) | 2416.43 (2067.51, 2863.64) | 2124.09 (1854.83, 2402.83) | -12.10 (-19.80, -4.89) | -0.44 (-0.52, -0.37)* |
| Comoros | 14.75 (12.44, 17.58) | 27.24 (22.94, 32.43) | 84.68 (72.33, 99.59) | 4566.39 (3894.19, 5363.38) | 4342.19 (3681.23, 5145.22) | -4.91 (-11.24, 2.34) | -0.17 (-0.19, -0.16)* |
| Congo | 128.94 (106.76, 155.68) | 286.78 (237.51, 346.80) | 122.41 (106.05, 139.48) | 7065.74 (5961.18, 8326.58) | 6426.16 (5413.50, 7589.29) | -9.05 (-14.36, -3.43) | -0.33 (-0.35, -0.31)* |
| Cook Islands | 0.46 (0.36, 0.58) | 0.51 (0.41, 0.63) | 9.91 (1.17, 20.55) | 2636.09 (2110.39, 3293.12) | 2662.33 (2105.06, 3345.46) | 1.00 (-3.68, 5.15) | 0.03 (0.02, 0.04)* |
| Costa Rica | 86.46 (73.22, 102.46) | 181.43 (154.57, 212.93) | 109.83 (93.18, 126.16) | 3357.13 (2889.22, 3927.39) | 3546.07 (3033.08, 4162.25) | 5.63 (-1.28, 11.83) | 0.19 (0.17, 0.22)* |
| Croatia | 200.84 (173.07, 233.58) | 165.73 (142.01, 193.33) | -17.48 (-25.49, -6.86) | 3548.36 (3062.84, 4109.85) | 2864.47 (2449.13, 3312.53) | -19.27 (-25.78, -11.36) | -0.74 (-0.78, -0.69)* |
| Cuba | 684.34 (578.84, 805.25) | 629.05 (536.57, 730.98) | -8.08 (-18.91, 3.62) | 5994.81 (5099.19, 7062.54) | 4382.68 (3754.13, 5105.22) | -26.89 (-33.31, -20.09) | -1.08 (-1.11, -1.06)* |
| Cyprus | 24.46 (20.70, 28.95) | 45.25 (38.27, 53.67) | 84.99 (71.82, 99.46) | 3044.67 (2568.09, 3606.65) | 3042.09 (2575.17, 3618.65) | -0.08 (-5.42, 5.53) | -0.01 (-0.08, 0.07) |
| Czechia | 384.22 (331.25, 441.72) | 368.43 (315.86, 426.27) | -4.11 (-13.27, 5.39) | 3272.69 (2817.19, 3792.62) | 2675.01 (2301.81, 3084.56) | -18.26 (-25.03, -10.74) | -0.70 (-0.72, -0.68)* |
| Côte d’Ivoire | 319.57 (262.25, 386.39) | 765.86 (644.47, 902.97) | 139.65 (124.68, 156.33) | 3986.80 (3394.50, 4664.71) | 3996.64 (3409.15, 4655.96) | 0.25 (-5.25, 6.67) | 0.00 (-0.01, 0.01) |
| Democratic People's Republic of Korea | 438.36 (369.52, 520.39) | 583.91 (496.26, 687.14) | 33.20 (23.62, 45.37) | 2227.89 (1895.37, 2623.00) | 1935.46 (1649.05, 2274.38) | -13.13 (-18.78, -6.77) | -0.49 (-0.51, -0.46)* |
| Democratic Republic of the Congo | 1814.04 (1489.87, 2206.52) | 4243.85 (3501.93, 5191.56) | 133.95 (120.06, 150.37) | 6509.24 (5493.68, 7689.05) | 6299.32 (5329.98, 7485.75) | -3.22 (-8.56, 2.69) | -0.12 (-0.13, -0.10)* |
| Denmark | 296.54 (255.88, 345.94) | 248.39 (215.38, 286.64) | -16.23 (-23.44, -7.65) | 4966.91 (4281.76, 5796.44) | 3602.62 (3094.02, 4179.42) | -27.47 (-33.35, -20.40) | -1.12 (-1.20, -1.04)* |
| Djibouti | 14.77 (12.13, 17.75) | 46.43 (38.32, 55.63) | 214.40 (188.19, 241.97) | 4642.12 (3960.76, 5470.16) | 4667.74 (3953.60, 5513.75) | 0.55 (-5.39, 7.22) | 0.02 (0.01, 0.03)* |
| Dominica | 2.25 (1.91, 2.64) | 2.53 (2.16, 2.99) | 12.24 (4.86, 20.33) | 3273.48 (2784.69, 3846.19) | 3300.58 (2814.94, 3920.81) | 0.83 (-4.67, 6.06) | 0.03 (-0.01, 0.06) |
| Dominican Republic | 259.20 (215.95, 308.03) | 471.77 (400.09, 551.87) | 82.01 (67.45, 98.39) | 4326.20 (3662.21, 5096.14) | 4410.58 (3759.41, 5176.00) | 1.95 (-4.35, 8.99) | 0.06 (0.01, 0.11)* |
| Ecuador | 303.55 (256.86, 364.57) | 628.81 (534.63, 741.58) | 107.15 (88.76, 125.85) | 3559.88 (3067.77, 4168.10) | 3631.67 (3108.41, 4260.83) | 2.02 (-5.23, 10.46) | 0.07 (0.06, 0.08)* |
| Egypt | 1926.49 (1597.23, 2306.38) | 4002.42 (3335.90, 4782.11) | 107.76 (95.18, 121.84) | 4077.37 (3445.18, 4830.59) | 4312.12 (3645.65, 5110.75) | 5.76 (-0.38, 12.11) | 0.17 (0.11, 0.23)* |
| El Salvador | 191.08 (161.63, 228.94) | 241.67 (205.55, 287.03) | 26.47 (16.50, 36.50) | 4285.16 (3665.76, 5087.23) | 3828.17 (3269.75, 4530.20) | -10.66 (-16.85, -4.58) | -0.39 (-0.41, -0.37)* |
| Equatorial Guinea | 22.29 (18.40, 26.76) | 76.05 (61.86, 93.53) | 241.14 (217.32, 267.03) | 7222.14 (6057.16, 8558.90) | 6733.89 (5665.05, 7969.49) | -6.76 (-12.41, -0.80) | -0.25 (-0.26, -0.23)* |
| Eritrea | 106.94 (88.12, 128.45) | 259.62 (216.60, 310.72) | 142.76 (126.97, 159.96) | 5508.55 (4668.48, 6487.48) | 5215.60 (4458.31, 6109.83) | -5.32 (-11.13, 1.61) | -0.19 (-0.20, -0.18)* |
| Estonia | 96.16 (82.72, 111.17) | 69.80 (59.22, 81.80) | -27.41 (-34.63, -19.16) | 5413.56 (4657.48, 6256.27) | 3935.03 (3381.08, 4578.01) | -27.31 (-33.78, -20.35) | -1.10 (-1.16, -1.04)* |
| Eswatini | 22.94 (18.95, 27.68) | 44.19 (37.26, 52.78) | 92.59 (79.43, 107.12) | 4266.74 (3614.77, 5038.50) | 4690.31 (3994.12, 5507.21) | 9.93 (3.35, 17.71) | 0.33 (0.29, 0.37)* |
| Ethiopia | 1882.14 (1587.80, 2211.84) | 3718.00 (3132.01, 4391.07) | 97.54 (93.22, 102.51) | 5584.95 (4786.39, 6450.81) | 4973.71 (4256.12, 5746.76) | -10.94 (-13.07, -9.03) | -0.39 (-0.42, -0.37)* |
| Fiji | 15.71 (12.92, 18.99) | 20.89 (17.57, 24.84) | 32.92 (23.23, 42.81) | 2294.98 (1932.87, 2699.98) | 2314.72 (1959.92, 2730.02) | 0.86 (-5.53, 6.93) | 0.02 (0.02, 0.03)* |
| Finland | 303.80 (262.22, 354.44) | 269.89 (237.55, 308.58) | -11.16 (-19.55, -1.96) | 5559.33 (4791.14, 6534.63) | 4383.42 (3785.42, 5096.13) | -21.15 (-28.05, -13.85) | -0.83 (-0.95, -0.71)* |
| France | 3239.72 (2954.20, 3525.88) | 3161.92 (2738.66, 3633.15) | -2.40 (-12.91, 8.26) | 5038.07 (4581.82, 5488.05) | 4129.70 (3520.30, 4812.31) | -18.03 (-26.89, -8.03) | -0.67 (-0.75, -0.58)* |
| Gabon | 52.95 (43.90, 63.63) | 101.95 (84.56, 122.48) | 92.54 (80.10, 108.23) | 6758.06 (5725.18, 7959.08) | 6444.78 (5486.24, 7580.57) | -4.64 (-10.21, 1.79) | -0.16 (-0.18, -0.15)* |
| Gambia | 41.86 (34.43, 50.60) | 104.09 (86.22, 124.74) | 148.67 (133.36, 164.38) | 6422.42 (5510.35, 7506.44) | 6373.19 (5463.42, 7480.93) | -0.77 (-6.87, 5.64) | -0.04 (-0.08, 0.01) |
| Georgia | 196.39 (167.97, 229.55) | 154.15 (132.31, 179.71) | -21.51 (-27.24, -15.23) | 3317.42 (2842.06, 3855.71) | 3354.37 (2862.54, 3906.77) | 1.11 (-5.30, 7.50) | 0.04 (0.00, 0.08)* |
| Germany | 3031.70 (2708.74, 3381.56) | 3642.41 (3167.51, 4179.81) | 20.14 (9.46, 32.05) | 3257.30 (2914.66, 3645.90) | 3561.88 (3047.01, 4138.94) | 9.35 (-0.44, 20.06) | 0.33 (0.20, 0.46)* |
| Ghana | 485.11 (406.56, 579.96) | 1176.75 (985.11, 1393.35) | 142.57 (129.34, 156.96) | 4611.57 (3916.86, 5390.93) | 4614.33 (3934.33, 5395.81) | 0.06 (-5.34, 5.41) | 0.00 (-0.01, 0.01) |
| Greece | 693.86 (585.81, 810.62) | 752.66 (643.74, 881.48) | 8.47 (0.82, 18.94) | 5861.93 (4952.53, 6866.01) | 5932.12 (5004.98, 7005.85) | 1.20 (-4.83, 9.04) | 0.02 (-0.11, 0.15) |
| Greenland | 4.92 (4.08, 5.91) | 4.09 (3.42, 4.78) | -16.75 (-23.57, -9.65) | 7883.15 (6668.35, 9320.06) | 7035.86 (5894.95, 8283.97) | -10.75 (-17.05, -4.39) | -0.38 (-0.45, -0.32)* |
| Grenada | 2.63 (2.24, 3.09) | 3.97 (3.37, 4.67) | 50.85 (40.63, 61.69) | 3520.84 (3007.18, 4133.32) | 3525.82 (3003.01, 4134.69) | 0.14 (-5.17, 6.03) | 0.00 (-0.04, 0.05) |
| Guam | 3.70 (3.07, 4.50) | 4.77 (4.06, 5.60) | 28.76 (18.00, 41.30) | 2708.29 (2279.96, 3203.79) | 2738.41 (2314.61, 3227.66) | 1.11 (-5.25, 8.78) | 0.04 (0.03, 0.05)* |
| Guatemala | 252.15 (211.53, 299.32) | 661.90 (550.55, 781.93) | 162.50 (146.28, 181.22) | 4403.73 (3728.13, 5181.48) | 4223.20 (3548.41, 4948.90) | -4.10 (-10.29, 3.05) | -0.12 (-0.22, -0.03)* |
| Guinea | 196.23 (165.70, 230.75) | 408.42 (343.06, 483.22) | 108.14 (95.54, 120.88) | 4429.09 (3759.74, 5171.79) | 4634.53 (3923.63, 5401.36) | 4.64 (-2.35, 11.54) | 0.16 (0.15, 0.17)* |
| Guinea-Bissau | 31.10 (25.81, 37.10) | 65.54 (53.98, 78.19) | 110.73 (98.32, 124.16) | 4609.90 (3915.48, 5447.12) | 4815.91 (4102.40, 5597.99) | 4.47 (-2.09, 11.43) | 0.15 (0.13, 0.17)* |
| Guyana | 37.40 (31.35, 45.01) | 47.25 (39.72, 55.93) | 26.35 (15.88, 37.98) | 5567.53 (4722.78, 6592.91) | 6054.12 (5128.84, 7110.28) | 8.74 (0.20, 16.92) | 0.30 (0.26, 0.34)* |
| Haiti | 217.97 (181.93, 261.69) | 458.09 (378.11, 552.87) | 110.17 (98.64, 122.62) | 4254.86 (3613.97, 5004.02) | 4117.85 (3471.87, 4855.94) | -3.22 (-8.55, 1.96) | -0.11 (-0.13, -0.10)* |
| Honduras | 107.93 (90.63, 128.09) | 292.16 (246.27, 348.45) | 170.69 (153.52, 190.15) | 3293.65 (2821.96, 3841.12) | 3470.08 (2975.66, 4082.66) | 5.36 (-0.63, 11.32) | 0.18 (0.16, 0.21)* |
| Hungary | 419.39 (363.20, 485.24) | 340.93 (293.97, 399.15) | -18.71 (-26.62, -8.81) | 3471.73 (3003.89, 4008.67) | 2591.21 (2243.36, 2999.65) | -25.36 (-31.96, -17.72) | -1.00 (-1.04, -0.95)* |
| Iceland | 8.43 (7.24, 9.84) | 10.65 (9.17, 12.33) | 26.40 (15.96, 36.67) | 3193.07 (2760.30, 3727.76) | 2783.36 (2371.70, 3269.13) | -12.83 (-19.13, -6.23) | -0.49 (-0.54, -0.43)* |
| India | 29992.25 (25782.60, 34879.06) | 51433.40 (44729.24, 58814.14) | 71.49 (64.52, 77.99) | 4333.01 (3759.88, 4961.15) | 3783.42 (3317.91, 4305.44) | -12.68 (-15.31, -10.24) | -0.48 (-0.58, -0.39)* |
| Indonesia | 2667.24 (2228.21, 3172.89) | 4287.23 (3604.35, 5066.60) | 60.74 (53.39, 68.41) | 1604.50 (1367.22, 1877.39) | 1586.97 (1347.83, 1855.30) | -1.09 (-2.56, 0.40) | -0.04 (-0.07, -0.01)* |
| Iran (Islamic Republic of) | 2571.17 (2102.46, 3106.65) | 5182.69 (4265.07, 6226.21) | 101.57 (83.98, 119.29) | 5419.34 (4489.55, 6446.93) | 5714.26 (4751.58, 6775.21) | 5.44 (3.72, 7.18) | 0.20 (0.14, 0.26)* |
| Iraq | 586.22 (481.25, 704.94) | 1713.31 (1415.22, 2030.24) | 192.26 (172.04, 213.32) | 4272.28 (3599.26, 5062.34) | 4288.86 (3637.18, 5046.80) | 0.39 (-5.74, 7.09) | 0.04 (-0.03, 0.10) |
| Ireland | 160.22 (138.14, 185.35) | 248.43 (219.29, 279.74) | 55.06 (42.58, 69.31) | 4383.45 (3777.43, 5075.42) | 4648.12 (4070.89, 5326.54) | 6.04 (-2.71, 15.87) | 0.27 (0.12, 0.43)* |
| Israel | 237.45 (204.37, 278.67) | 432.29 (371.82, 508.21) | 82.06 (69.02, 97.35) | 4925.24 (4233.14, 5776.20) | 4551.76 (3898.53, 5331.55) | -7.58 (-14.20, -0.22) | -0.30 (-0.36, -0.24)* |
| Italy | 2709.81 (2334.24, 3112.86) | 2848.28 (2464.77, 3247.83) | 5.11 (0.93, 9.25) | 4045.85 (3456.10, 4681.72) | 3785.86 (3251.38, 4405.44) | -6.43 (-7.80, -4.95) | -0.25 (-0.32, -0.17)* |
| Jamaica | 67.86 (56.66, 81.19) | 99.00 (83.48, 117.52) | 45.90 (35.20, 57.05) | 3217.32 (2699.65, 3824.13) | 3267.67 (2759.84, 3877.94) | 1.57 (-3.93, 7.41) | 0.05 (0.01, 0.09)* |
| Japan | 3027.63 (2693.79, 3385.19) | 3474.03 (3109.76, 3855.59) | 14.74 (9.63, 20.21) | 2136.78 (1897.09, 2406.86) | 2288.92 (2021.12, 2590.72) | 7.12 (5.16, 9.23) | 0.25 (0.18, 0.31)* |
| Jordan | 154.22 (125.90, 187.22) | 516.45 (427.94, 618.05) | 234.87 (208.39, 261.23) | 5239.94 (4399.36, 6180.81) | 4645.62 (3906.67, 5514.36) | -11.34 (-17.82, -4.70) | -0.42 (-0.46, -0.39)* |
| Kazakhstan | 542.30 (462.38, 631.21) | 648.74 (556.06, 762.42) | 19.63 (9.69, 30.13) | 3618.10 (3108.09, 4202.52) | 3529.51 (3038.57, 4099.10) | -2.45 (-9.98, 6.04) | -0.08 (-0.11, -0.04)* |
| Kenya | 796.46 (685.62, 917.06) | 1929.39 (1672.94, 2214.55) | 142.24 (137.25, 147.28) | 5512.31 (4842.54, 6245.70) | 5078.45 (4481.45, 5740.19) | -7.87 (-9.08, -6.66) | -0.28 (-0.31, -0.25)* |
| Kiribati | 1.71 (1.38, 2.10) | 2.58 (2.14, 3.12) | 51.58 (41.17, 63.11) | 2612.74 (2170.15, 3124.20) | 2362.52 (1997.48, 2804.76) | -9.58 (-15.89, -3.26) | -0.35 (-0.36, -0.34)* |
| Kuwait | 72.73 (59.22, 88.53) | 224.76 (183.70, 272.13) | 209.05 (181.79, 242.91) | 4321.62 (3630.41, 5122.80) | 4503.22 (3775.08, 5284.96) | 4.20 (-1.65, 10.98) | 0.14 (0.11, 0.17)* |
| Kyrgyzstan | 142.28 (121.09, 167.38) | 202.86 (170.08, 241.27) | 42.57 (30.58, 55.33) | 3846.76 (3306.77, 4494.86) | 3441.98 (2922.95, 4041.11) | -10.52 (-17.89, -2.37) | -0.39 (-0.41, -0.36)* |
| Lao People's Democratic Republic | 79.87 (65.90, 97.20) | 142.87 (118.66, 171.00) | 78.88 (66.40, 94.61) | 2330.51 (1954.04, 2772.74) | 2045.51 (1733.00, 2416.20) | -12.23 (-18.30, -5.26) | -0.46 (-0.51, -0.40)* |
| Latvia | 151.90 (130.17, 178.50) | 103.46 (89.68, 117.81) | -31.89 (-38.55, -24.12) | 4936.61 (4243.19, 5769.88) | 4009.37 (3510.14, 4575.10) | -18.78 (-25.45, -10.38) | -0.72 (-0.76, -0.68)* |
| Lebanon | 144.46 (121.27, 170.64) | 283.42 (237.01, 334.17) | 96.20 (78.03, 114.93) | 5039.35 (4268.49, 5955.79) | 5359.18 (4485.80, 6330.33) | 6.35 (-2.74, 15.26) | 0.22 (0.16, 0.28)* |
| Lesotho | 86.47 (72.64, 102.92) | 117.68 (99.23, 139.18) | 36.10 (23.68, 47.84) | 6226.08 (5281.37, 7331.59) | 6339.67 (5466.89, 7420.85) | 1.82 (-6.19, 10.20) | 0.05 (-0.01, 0.11) |
| Liberia | 71.01 (59.76, 83.70) | 181.31 (150.24, 217.35) | 155.34 (137.81, 176.19) | 4959.80 (4237.49, 5773.48) | 4939.92 (4227.12, 5777.52) | -0.40 (-7.37, 7.35) | 0.02 (-0.05, 0.09) |
| Libya | 161.66 (133.41, 196.47) | 368.70 (306.74, 440.96) | 128.07 (108.17, 152.50) | 4753.83 (4040.44, 5678.94) | 5007.28 (4247.48, 5965.82) | 5.33 (-0.02, 11.25) | 0.18 (0.16, 0.19)* |
| Lithuania | 194.14 (166.00, 225.84) | 161.87 (138.64, 188.72) | -16.62 (-24.38, -8.20) | 4774.49 (4098.45, 5548.29) | 4372.46 (3744.94, 5086.06) | -8.42 (-15.01, -0.09) | -0.29 (-0.33, -0.26)* |
| Luxembourg | 17.90 (15.32, 20.80) | 23.01 (20.14, 26.26) | 28.55 (16.72, 40.14) | 4129.22 (3532.84, 4813.04) | 3205.69 (2808.74, 3677.27) | -22.37 (-28.77, -15.73) | -0.88 (-0.92, -0.84)* |
| Madagascar | 428.80 (356.76, 514.95) | 990.41 (827.61, 1179.73) | 130.97 (115.74, 146.34) | 5258.98 (4435.82, 6170.98) | 5059.17 (4302.38, 5891.82) | -3.80 (-10.04, 2.64) | -0.13 (-0.14, -0.12)* |
| Malawi | 280.68 (233.14, 334.46) | 531.78 (442.88, 633.78) | 89.46 (77.40, 102.24) | 4345.35 (3708.43, 5098.35) | 4077.76 (3478.41, 4780.28) | -6.16 (-12.06, 0.30) | -0.22 (-0.25, -0.19)* |
| Malaysia | 429.97 (357.04, 512.63) | 1104.21 (942.42, 1293.93) | 156.81 (132.00, 185.93) | 2851.87 (2406.14, 3354.04) | 3421.09 (2938.54, 3991.65) | 19.96 (8.54, 33.39) | 0.60 (0.51, 0.70)* |
| Maldives | 5.63 (4.64, 6.76) | 12.11 (10.12, 14.40) | 115.11 (92.49, 137.53) | 3273.33 (2781.24, 3820.04) | 2380.88 (2023.33, 2786.84) | -27.26 (-32.13, -22.26) | -1.10 (-1.14, -1.06)* |
| Mali | 207.53 (174.21, 246.30) | 496.00 (410.22, 597.11) | 139.00 (121.54, 155.75) | 3479.34 (2970.84, 4081.95) | 3384.12 (2872.45, 3994.38) | -2.74 (-9.47, 3.98) | -0.10 (-0.15, -0.06)* |
| Malta | 12.38 (10.60, 14.50) | 15.84 (13.48, 18.51) | 27.96 (19.07, 38.43) | 3153.58 (2689.57, 3713.57) | 3074.44 (2598.46, 3623.75) | -2.51 (-8.10, 2.82) | -0.10 (-0.12, -0.07)* |
| Marshall Islands | 0.85 (0.69, 1.05) | 1.24 (1.02, 1.48) | 46.08 (35.16, 58.01) | 2399.89 (2003.41, 2877.13) | 2276.14 (1911.00, 2708.48) | -5.16 (-10.19, 0.47) | -0.19 (-0.20, -0.17)* |
| Mauritania | 54.05 (45.25, 63.99) | 109.07 (91.71, 130.57) | 101.79 (88.51, 115.95) | 3711.29 (3128.51, 4358.27) | 3551.63 (3019.63, 4187.82) | -4.30 (-10.79, 2.66) | -0.15 (-0.18, -0.13)* |
| Mauritius | 45.72 (38.48, 54.96) | 52.65 (44.70, 61.45) | 15.16 (4.17, 27.91) | 4196.47 (3590.83, 4951.01) | 3614.35 (3049.03, 4248.25) | -13.87 (-20.30, -7.59) | -0.52 (-0.55, -0.49)* |
| Mexico | 2221.28 (1921.37, 2532.65) | 5160.63 (4498.21, 5891.41) | 132.33 (122.07, 143.08) | 3266.10 (2871.44, 3690.19) | 4006.79 (3505.57, 4567.70) | 22.68 (19.75, 25.94) | 0.71 (0.68, 0.74)* |
| Micronesia (Federated States of) | 2.08 (1.71, 2.55) | 2.30 (1.90, 2.79) | 10.32 (2.08, 20.18) | 2471.28 (2099.01, 2960.67) | 2299.10 (1921.88, 2751.05) | -6.97 (-12.81, -1.14) | -0.25 (-0.26, -0.24)* |
| Monaco | 1.67 (1.36, 2.04) | 2.03 (1.65, 2.47) | 21.80 (15.15, 28.60) | 4474.51 (3601.12, 5524.11) | 4460.76 (3600.58, 5523.46) | -0.31 (-4.54, 3.63) | -0.01 (-0.02, -0.01)* |
| Mongolia | 72.61 (60.60, 86.74) | 138.05 (115.78, 163.11) | 90.12 (72.22, 109.39) | 4328.39 (3683.60, 5068.45) | 4308.98 (3656.52, 5024.83) | -0.45 (-7.02, 6.29) | -0.01 (-0.04, 0.01) |
| Montenegro | 17.05 (14.47, 20.04) | 19.63 (16.62, 22.86) | 15.13 (5.07, 25.85) | 2648.72 (2257.00, 3097.36) | 2582.29 (2200.55, 3014.52) | -2.51 (-9.32, 4.40) | -0.08 (-0.10, -0.06)* |
| Morocco | 1330.16 (1101.97, 1591.11) | 2236.87 (1883.53, 2678.75) | 68.17 (54.31, 83.49) | 6164.33 (5194.14, 7273.35) | 6012.99 (5100.42, 7147.18) | -2.46 (-9.39, 5.07) | -0.09 (-0.10, -0.08)* |
| Mozambique | 470.25 (393.83, 556.48) | 1059.74 (880.98, 1259.87) | 125.36 (110.01, 140.69) | 5179.26 (4415.10, 6027.34) | 5419.07 (4607.34, 6365.86) | 4.63 (-2.92, 12.46) | 0.15 (0.14, 0.16)* |
| Myanmar | 438.07 (361.08, 530.69) | 659.84 (544.29, 785.79) | 50.62 (40.52, 62.11) | 1215.31 (1025.03, 1433.49) | 1192.47 (993.14, 1409.63) | -1.88 (-7.21, 3.91) | -0.09 (-0.24, 0.06) |
| Namibia | 37.60 (31.64, 44.91) | 71.10 (60.08, 84.59) | 89.12 (75.92, 103.34) | 3534.18 (3013.23, 4137.21) | 3473.53 (2974.87, 4043.05) | -1.72 (-7.68, 4.98) | -0.08 (-0.15, -0.01)* |
| Nauru | 0.22 (0.17, 0.28) | 0.25 (0.19, 0.32) | 14.28 (8.06, 20.06) | 2618.30 (2086.45, 3276.54) | 2645.75 (2107.53, 3313.75) | 1.05 (-4.02, 5.60) | 0.03 (0.02, 0.04)* |
| Nepal | 747.19 (628.40, 882.11) | 1559.44 (1342.15, 1818.04) | 108.71 (93.17, 125.96) | 5289.49 (4477.06, 6167.09) | 5644.49 (4855.93, 6568.15) | 6.71 (-0.63, 14.65) | 0.22 (0.16, 0.29)* |
| Netherlands | 662.11 (604.11, 722.08) | 749.62 (644.43, 876.95) | 13.22 (2.36, 26.37) | 3911.45 (3570.26, 4269.55) | 3750.68 (3228.62, 4388.72) | -4.11 (-13.67, 7.63) | -0.12 (-0.17, -0.07)* |
| New Zealand | 136.13 (111.59, 163.03) | 176.63 (151.94, 203.39) | 29.75 (20.42, 40.66) | 3774.05 (3100.16, 4529.67) | 4008.58 (3428.80, 4667.92) | 6.21 (-0.55, 14.35) | 0.20 (0.19, 0.22)* |
| Nicaragua | 102.46 (85.02, 122.25) | 222.34 (187.29, 264.65) | 116.99 (99.92, 135.77) | 3556.41 (3011.18, 4167.92) | 3609.20 (3083.96, 4230.74) | 1.48 (-4.22, 9.00) | 0.05 (0.04, 0.07)* |
| Niger | 218.36 (181.48, 261.27) | 623.82 (520.68, 742.20) | 185.69 (167.50, 204.68) | 4315.29 (3696.32, 5043.31) | 4432.19 (3778.57, 5178.61) | 2.71 (-4.11, 9.17) | 0.09 (0.08, 0.10)* |
| Nigeria | 2919.85 (2480.97, 3413.03) | 5636.04 (4788.95, 6583.59) | 93.02 (90.09, 96.74) | 4501.55 (3849.77, 5212.33) | 3809.90 (3266.40, 4379.57) | -15.36 (-16.70, -13.94) | -0.57 (-0.64, -0.50)* |
| Niue | 0.06 (0.05, 0.07) | 0.05 (0.04, 0.06) | -17.19 (-22.23, -11.74) | 2650.24 (2114.90, 3308.44) | 2644.39 (2103.86, 3331.45) | -0.22 (-4.26, 4.15) | -0.01 (-0.02, -0.00)* |
| North Macedonia | 49.15 (41.48, 57.96) | 58.48 (49.28, 68.29) | 18.99 (8.64, 31.42) | 2424.48 (2058.38, 2838.05) | 2216.79 (1883.65, 2583.12) | -8.57 (-15.21, -0.65) | -0.31 (-0.36, -0.26)* |
| Northern Mariana Islands | 0.92 (0.76, 1.13) | 1.02 (0.86, 1.21) | 11.21 (-2.40, 26.23) | 1990.90 (1693.71, 2346.59) | 2126.17 (1798.53, 2513.46) | 6.79 (0.60, 13.68) | 0.23 (0.16, 0.29)* |
| Norway | 131.18 (113.07, 151.98) | 190.73 (163.47, 220.78) | 45.39 (41.69, 49.11) | 2818.55 (2411.23, 3270.29) | 3242.60 (2746.25, 3781.84) | 15.04 (12.89, 17.01) | 0.47 (0.30, 0.64)* |
| Oman | 69.21 (56.83, 83.96) | 209.63 (169.28, 257.45) | 202.90 (177.81, 231.10) | 4374.69 (3702.37, 5166.70) | 4416.82 (3736.98, 5204.86) | 0.96 (-5.03, 7.08) | 0.03 (-0.04, 0.10) |
| Pakistan | 3477.15 (2927.42, 4080.87) | 7317.52 (6147.59, 8636.69) | 110.45 (104.51, 116.42) | 4396.35 (3713.88, 5122.39) | 4206.27 (3550.18, 4888.20) | -4.32 (-6.93, -1.76) | -0.15 (-0.19, -0.12)* |
| Palau | 0.40 (0.31, 0.51) | 0.52 (0.42, 0.65) | 32.21 (18.25, 48.51) | 2631.11 (2097.74, 3316.46) | 2610.78 (2078.30, 3273.55) | -0.77 (-5.08, 4.13) | -0.03 (-0.06, -0.01)* |
| Palestine | 118.73 (99.33, 143.21) | 334.92 (279.33, 402.01) | 182.09 (166.55, 200.40) | 7822.59 (6625.25, 9221.69) | 7687.68 (6546.14, 9023.85) | -1.72 (-6.81, 3.46) | -0.06 (-0.14, 0.01) |
| Panama | 66.39 (55.58, 78.33) | 132.44 (112.49, 154.41) | 99.49 (86.13, 115.52) | 3197.46 (2714.04, 3739.12) | 3145.01 (2672.81, 3665.83) | -1.64 (-7.14, 4.17) | -0.06 (-0.06, -0.05)* |
| Papua New Guinea | 96.74 (79.29, 118.65) | 228.46 (187.68, 279.57) | 136.15 (122.28, 149.96) | 2718.02 (2281.61, 3250.68) | 2558.42 (2147.08, 3054.47) | -5.87 (-10.81, -1.16) | -0.21 (-0.22, -0.20)* |
| Paraguay | 133.71 (111.06, 159.89) | 290.66 (244.11, 342.67) | 117.38 (101.02, 134.31) | 3891.13 (3307.21, 4575.39) | 4199.46 (3588.56, 4938.69) | 7.92 (0.83, 14.72) | 0.26 (0.24, 0.28)* |
| Peru | 409.68 (344.57, 488.69) | 675.21 (574.45, 793.71) | 64.81 (48.73, 81.21) | 2274.11 (1956.88, 2671.00) | 1967.59 (1676.66, 2302.62) | -13.48 (-20.81, -6.66) | -0.51 (-0.54, -0.48)* |
| Philippines | 1372.74 (1140.74, 1626.23) | 2265.78 (1907.44, 2676.94) | 65.06 (60.52, 70.05) | 2616.95 (2224.22, 3046.97) | 2137.81 (1826.35, 2483.59) | -18.31 (-19.22, -17.37) | -0.71 (-0.76, -0.66)* |
| Poland | 715.65 (610.12, 835.25) | 845.54 (722.17, 977.21) | 18.15 (13.21, 23.15) | 1770.51 (1509.48, 2068.05) | 1772.37 (1515.98, 2068.39) | 0.10 (-1.43, 1.72) | -0.01 (-0.05, 0.02) |
| Portugal | 650.10 (563.28, 759.86) | 679.73 (581.14, 792.84) | 4.56 (-5.65, 15.49) | 5754.65 (4953.55, 6748.93) | 4969.86 (4236.77, 5805.27) | -13.64 (-20.91, -6.56) | -0.47 (-0.55, -0.39)* |
| Puerto Rico | 114.38 (98.08, 131.83) | 129.95 (110.29, 152.11) | 13.62 (4.94, 24.33) | 3134.38 (2689.88, 3618.72) | 2988.71 (2548.56, 3503.54) | -4.65 (-10.81, 2.78) | -0.17 (-0.22, -0.12)* |
| Qatar | 21.74 (17.61, 26.39) | 149.37 (121.01, 183.76) | 587.10 (530.99, 651.52) | 4796.34 (4074.98, 5655.93) | 4454.52 (3776.05, 5260.66) | -7.13 (-13.55, -0.44) | -0.26 (-0.29, -0.22)* |
| Republic of Korea | 873.67 (755.15, 1006.24) | 1346.94 (1191.29, 1510.55) | 54.17 (38.74, 71.82) | 1923.90 (1674.88, 2202.08) | 2122.29 (1877.56, 2388.11) | 10.31 (2.75, 18.26) | 0.34 (0.29, 0.39)* |
| Republic of Moldova | 177.26 (150.99, 207.29) | 151.85 (128.88, 180.99) | -14.34 (-22.92, -5.06) | 3925.80 (3351.50, 4586.22) | 3218.75 (2734.31, 3812.79) | -18.01 (-25.50, -10.42) | -0.67 (-0.72, -0.62)* |
| Romania | 620.95 (525.74, 722.56) | 581.47 (493.72, 676.59) | -6.36 (-15.07, 3.91) | 2427.81 (2052.75, 2815.29) | 2327.84 (1981.23, 2690.92) | -4.12 (-11.87, 4.90) | -0.14 (-0.15, -0.13)* |
| Russian Federation | 5398.54 (4598.32, 6254.59) | 5295.09 (4515.04, 6104.85) | -1.92 (-4.18, 0.32) | 3248.99 (2780.49, 3762.29) | 3000.32 (2572.89, 3483.53) | -7.65 (-8.98, -6.20) | -0.26 (-0.32, -0.21)* |
| Rwanda | 313.85 (261.63, 372.96) | 549.60 (461.88, 652.53) | 75.11 (63.68, 88.74) | 6617.54 (5646.21, 7733.87) | 5593.22 (4772.21, 6549.83) | -15.48 (-20.94, -9.32) | -0.59 (-0.62, -0.55)* |
| Saint Kitts and Nevis | 1.72 (1.39, 2.14) | 3.08 (2.46, 3.84) | 78.89 (63.52, 96.48) | 4607.98 (3737.86, 5706.85) | 4570.72 (3689.28, 5676.06) | -0.81 (-4.74, 3.59) | -0.03 (-0.05, -0.01)* |
| Saint Lucia | 4.20 (3.52, 5.01) | 7.28 (6.18, 8.52) | 73.17 (59.06, 89.85) | 3589.88 (3054.52, 4215.47) | 3644.63 (3117.49, 4262.39) | 1.53 (-4.01, 7.99) | 0.05 (0.01, 0.09)* |
| Saint Vincent and the Grenadines | 3.35 (2.83, 3.95) | 4.62 (3.96, 5.44) | 37.69 (25.50, 50.14) | 3561.25 (3035.20, 4190.87) | 3722.10 (3196.37, 4376.76) | 4.52 (-1.50, 10.97) | 0.15 (0.10, 0.20)* |
| Samoa | 3.47 (2.83, 4.21) | 4.26 (3.57, 5.13) | 22.68 (14.53, 31.75) | 2404.84 (2012.47, 2863.77) | 2152.03 (1821.86, 2572.61) | -10.51 (-15.62, -4.84) | -0.39 (-0.42, -0.36)* |
| San Marino | 1.17 (0.95, 1.44) | 1.74 (1.41, 2.13) | 49.01 (40.76, 58.43) | 4452.85 (3602.24, 5508.13) | 4509.00 (3634.35, 5634.18) | 1.26 (-3.13, 5.84) | 0.04 (0.04, 0.04)* |
| Sao Tome and Principe | 3.15 (2.59, 3.75) | 6.23 (5.16, 7.47) | 98.07 (85.38, 110.66) | 3604.05 (3056.73, 4270.51) | 3642.16 (3092.46, 4319.51) | 1.06 (-4.61, 6.84) | 0.03 (0.01, 0.06)* |
| Saudi Arabia | 585.55 (480.16, 710.69) | 1851.76 (1531.17, 2240.81) | 216.24 (180.65, 251.66) | 4378.98 (3706.85, 5198.68) | 4670.15 (3951.91, 5511.25) | 6.65 (-0.31, 14.36) | 0.22 (0.20, 0.24)* |
| Senegal | 205.56 (172.91, 242.59) | 467.05 (391.11, 551.83) | 127.20 (113.69, 143.03) | 4076.72 (3471.20, 4741.74) | 4121.74 (3515.16, 4792.70) | 1.10 (-5.09, 8.26) | 0.03 (-0.03, 0.09) |
| Serbia | 313.43 (265.15, 367.05) | 290.88 (249.33, 338.69) | -7.20 (-16.61, 1.57) | 3000.68 (2553.50, 3479.74) | 2579.62 (2208.31, 3016.30) | -14.03 (-21.04, -7.01) | -0.52 (-0.55, -0.49)* |
| Seychelles | 1.48 (1.24, 1.77) | 2.12 (1.80, 2.51) | 43.59 (31.32, 55.95) | 2144.06 (1817.04, 2526.71) | 1923.06 (1622.97, 2278.25) | -10.31 (-15.38, -4.45) | -0.38 (-0.45, -0.31)* |
| Sierra Leone | 116.71 (97.76, 138.61) | 288.24 (241.56, 344.72) | 146.97 (130.43, 166.13) | 4347.20 (3702.10, 5130.45) | 4677.53 (3973.33, 5484.80) | 7.60 (0.92, 15.71) | 0.25 (0.24, 0.26)* |
| Singapore | 109.26 (95.46, 125.52) | 136.64 (118.29, 156.69) | 25.06 (12.57, 38.07) | 3359.09 (2956.27, 3820.50) | 2161.31 (1872.85, 2508.01) | -35.66 (-41.41, -29.28) | -1.53 (-1.63, -1.43)* |
| Slovakia | 153.58 (132.56, 179.68) | 166.78 (142.00, 194.23) | 8.59 (-0.15, 19.97) | 2739.10 (2367.75, 3212.76) | 2398.42 (2050.85, 2789.71) | -12.44 (-18.81, -4.56) | -0.46 (-0.48, -0.45)* |
| Slovenia | 89.19 (77.07, 103.46) | 84.64 (72.38, 97.85) | -5.11 (-14.71, 5.41) | 4011.12 (3474.33, 4639.28) | 3026.94 (2618.00, 3513.66) | -24.54 (-31.03, -17.60) | -0.95 (-1.02, -0.89)* |
| Solomon Islands | 7.03 (5.70, 8.59) | 13.97 (11.48, 17.04) | 98.76 (85.09, 112.36) | 2583.09 (2175.20, 3081.88) | 2447.06 (2047.22, 2929.81) | -5.27 (-10.80, -0.20) | -0.19 (-0.20, -0.18)* |
| Somalia | 244.83 (201.44, 294.81) | 688.05 (570.05, 821.25) | 181.04 (163.06, 199.98) | 5270.04 (4458.20, 6201.36) | 5230.55 (4473.87, 6157.81) | -0.75 (-7.40, 5.79) | -0.03 (-0.03, -0.02)* |
| South Africa | 1400.00 (1213.80, 1600.07) | 2501.49 (2182.53, 2832.70) | 78.68 (71.71, 85.76) | 4514.14 (3961.09, 5085.68) | 4555.32 (4014.76, 5112.72) | 0.91 (-1.61, 3.59) | 0.04 (-0.00, 0.09) |
| South Sudan | 200.26 (166.28, 239.82) | 330.12 (275.73, 393.65) | 64.85 (53.66, 75.94) | 4926.50 (4170.32, 5787.97) | 5066.49 (4308.71, 5949.34) | 2.84 (-4.22, 9.40) | 0.10 (0.08, 0.10)* |
| Spain | 1904.38 (1743.93, 2071.92) | 2991.78 (2682.20, 3293.54) | 57.10 (45.86, 68.73) | 4422.23 (4045.64, 4812.02) | 5396.21 (4772.08, 6015.79) | 22.02 (12.54, 31.60) | 0.70 (0.65, 0.74)* |
| Sri Lanka | 503.37 (425.12, 598.53) | 509.64 (438.21, 596.37) | 1.25 (-6.50, 10.98) | 2983.05 (2554.66, 3491.91) | 2211.56 (1891.07, 2599.17) | -25.86 (-31.02, -20.40) | -1.02 (-1.12, -0.92)* |
| Sudan | 831.40 (680.76, 1010.79) | 1798.54 (1489.11, 2177.45) | 116.33 (101.99, 131.97) | 5142.12 (4326.47, 6142.99) | 5024.55 (4237.68, 6003.54) | -2.29 (-8.39, 4.41) | -0.08 (-0.09, -0.07)* |
| Suriname | 19.11 (16.08, 22.68) | 34.27 (29.19, 39.81) | 79.37 (65.56, 95.40) | 5373.76 (4560.28, 6306.86) | 5698.25 (4870.92, 6650.50) | 6.04 (-0.79, 13.51) | 0.21 (0.18, 0.23)* |
| Sweden | 449.21 (398.15, 508.82) | 515.46 (457.81, 579.37) | 14.75 (7.88, 21.49) | 4608.39 (4048.13, 5263.79) | 4467.65 (3930.06, 5098.32) | -3.05 (-8.34, 2.55) | -0.11 (-0.14, -0.08)* |
| Switzerland | 382.09 (331.19, 446.67) | 396.20 (351.00, 443.97) | 3.69 (-4.61, 13.57) | 4876.21 (4201.30, 5676.03) | 3797.86 (3333.23, 4287.87) | -22.11 (-28.02, -15.35) | -0.86 (-0.98, -0.75)* |
| Syrian Arab Republic | 450.65 (367.43, 546.41) | 675.46 (563.31, 804.11) | 49.89 (37.41, 64.38) | 4560.78 (3850.89, 5382.74) | 4568.49 (3848.56, 5417.41) | 0.17 (-4.61, 5.85) | 0.00 (-0.04, 0.05) |
| Taiwan (Province of China) | 328.71 (279.44, 384.26) | 530.13 (449.47, 622.78) | 61.28 (44.98, 78.37) | 1638.22 (1401.17, 1899.11) | 1762.36 (1513.51, 2052.34) | 7.58 (-0.07, 15.31) | 0.25 (0.23, 0.27)* |
| Tajikistan | 114.30 (96.85, 136.56) | 206.80 (175.12, 246.46) | 80.92 (68.52, 96.37) | 2879.39 (2442.41, 3388.45) | 2600.09 (2237.28, 3022.24) | -9.70 (-16.28, -2.09) | -0.35 (-0.37, -0.33)* |
| Thailand | 1229.98 (1027.42, 1461.40) | 1850.61 (1571.17, 2178.25) | 50.46 (32.32, 72.95) | 2191.45 (1856.10, 2551.30) | 2193.56 (1872.17, 2571.64) | 0.10 (-6.98, 9.19) | 0.01 (-0.01, 0.02) |
| Timor-Leste | 16.48 (13.64, 19.84) | 25.49 (21.12, 30.97) | 54.72 (44.16, 64.95) | 2545.66 (2173.82, 2979.10) | 2141.99 (1810.72, 2530.90) | -15.86 (-21.08, -10.82) | -0.59 (-0.61, -0.57)* |
| Togo | 108.90 (90.36, 130.75) | 289.20 (241.14, 343.66) | 165.58 (148.14, 184.60) | 4637.97 (3910.95, 5479.95) | 4824.30 (4091.34, 5677.41) | 4.02 (-2.81, 10.85) | 0.14 (0.13, 0.14)* |
| Tokelau | 0.04 (0.03, 0.05) | 0.03 (0.03, 0.04) | -6.84 (-11.64, -1.62) | 2663.75 (2135.30, 3358.11) | 2626.86 (2092.07, 3294.00) | -1.39 (-5.75, 3.38) | -0.06 (-0.09, -0.02)* |
| Tonga | 1.68 (1.39, 2.05) | 1.87 (1.57, 2.24) | 11.37 (4.21, 19.07) | 2000.42 (1684.64, 2375.21) | 1968.63 (1666.92, 2336.60) | -1.59 (-7.07, 4.18) | -0.06 (-0.07, -0.04)* |
| Trinidad and Tobago | 52.27 (43.62, 63.14) | 68.86 (58.51, 80.96) | 31.74 (17.32, 45.59) | 4687.70 (3964.43, 5584.09) | 4408.06 (3738.17, 5157.57) | -5.97 (-13.48, 0.95) | -0.21 (-0.23, -0.19)* |
| Tunisia | 434.73 (362.17, 520.21) | 743.63 (625.04, 880.52) | 71.06 (56.03, 88.43) | 5977.79 (5067.48, 7052.65) | 5961.32 (5020.73, 7027.06) | -0.28 (-5.91, 6.21) | -0.01 (-0.04, 0.01) |
| Turkey | 2423.40 (2143.54, 2736.70) | 3874.52 (3284.47, 4575.29) | 59.88 (43.98, 78.37) | 4465.05 (3995.49, 4975.89) | 4294.86 (3644.71, 5047.20) | -3.81 (-12.54, 6.89) | -0.17 (-0.32, -0.02)* |
| Turkmenistan | 91.88 (77.75, 109.36) | 140.16 (118.98, 167.04) | 52.55 (39.53, 67.06) | 3234.05 (2765.29, 3791.89) | 2950.75 (2503.16, 3473.17) | -8.76 (-15.98, -0.97) | -0.32 (-0.33, -0.30)* |
| Tuvalu | 0.23 (0.18, 0.29) | 0.31 (0.24, 0.39) | 36.62 (30.72, 43.11) | 2667.33 (2127.82, 3337.18) | 2632.09 (2093.94, 3313.64) | -1.32 (-5.64, 3.10) | -0.05 (-0.06, -0.04)* |
| Uganda | 901.54 (746.32, 1082.97) | 2109.97 (1744.98, 2535.51) | 134.04 (116.22, 150.87) | 8139.10 (6959.61, 9636.41) | 7836.23 (6713.75, 9181.11) | -3.72 (-10.38, 3.07) | -0.13 (-0.17, -0.09)* |
| Ukraine | 2957.09 (2523.79, 3404.94) | 2442.38 (2081.74, 2814.27) | -17.41 (-21.41, -12.65) | 4837.82 (4130.20, 5588.76) | 4266.32 (3642.78, 4950.99) | -11.81 (-15.51, -7.50) | -0.45 (-0.50, -0.39)* |
| United Arab Emirates | 75.95 (61.76, 91.98) | 440.87 (350.07, 552.19) | 480.44 (403.58, 557.69) | 4216.40 (3584.70, 4978.78) | 3866.26 (3252.48, 4604.73) | -8.30 (-13.83, -2.37) | -0.31 (-0.34, -0.28)* |
| United Kingdom | 3335.25 (2876.77, 3839.69) | 3432.45 (2965.53, 3928.40) | 2.91 (0.58, 5.15) | 5208.95 (4472.36, 6022.91) | 4488.90 (3858.64, 5167.66) | -13.82 (-15.16, -12.57) | -0.51 (-0.62, -0.40)* |
| United Republic of Tanzania | 900.55 (753.87, 1075.69) | 1988.34 (1649.73, 2361.42) | 120.79 (107.72, 135.47) | 5172.12 (4388.52, 6082.02) | 4914.24 (4171.57, 5708.93) | -4.99 (-10.81, 1.80) | -0.18 (-0.19, -0.16)* |
| United States Virgin Islands | 3.68 (3.10, 4.36) | 4.55 (3.88, 5.35) | 23.66 (13.13, 36.42) | 3561.04 (3025.01, 4188.37) | 3699.81 (3146.12, 4355.68) | 3.90 (-2.09, 11.30) | 0.13 (0.09, 0.17)* |
| United States of America | 9775.42 (8597.47, 11081.70) | 16533.46 (14637.78, 18548.00) | 69.13 (64.86, 74.00) | 3628.32 (3191.02, 4135.59) | 4854.33 (4256.66, 5513.76) | 33.79 (30.66, 37.15) | 0.99 (0.83, 1.15)* |
| Uruguay | 91.16 (78.04, 106.19) | 121.58 (104.27, 141.26) | 33.37 (23.66, 44.20) | 2807.22 (2377.80, 3293.61) | 3206.75 (2731.23, 3752.78) | 14.23 (5.57, 23.31) | 0.47 (0.44, 0.51)* |
| Uzbekistan | 538.34 (454.01, 630.22) | 949.94 (801.16, 1127.72) | 76.46 (61.80, 92.23) | 3352.60 (2851.18, 3892.90) | 3114.47 (2666.16, 3639.84) | -7.10 (-14.76, 1.25) | -0.26 (-0.27, -0.24)* |
| Vanuatu | 3.22 (2.65, 3.94) | 6.38 (5.25, 7.72) | 98.00 (86.15, 110.37) | 2585.25 (2167.04, 3095.66) | 2405.27 (2025.02, 2875.08) | -6.96 (-11.98, -1.68) | -0.25 (-0.27, -0.24)* |
| Venezuela (Bolivarian Republic of) | 539.21 (449.76, 644.04) | 1047.84 (892.97, 1230.49) | 94.33 (78.08, 111.08) | 3498.78 (2986.08, 4101.80) | 3555.52 (3042.92, 4167.88) | 1.62 (-4.83, 7.56) | 0.06 (0.05, 0.06)* |
| Viet Nam | 1088.50 (907.77, 1313.86) | 1684.64 (1406.32, 1988.73) | 54.77 (41.53, 71.56) | 1835.91 (1559.39, 2176.10) | 1632.86 (1378.54, 1916.34) | -11.06 (-16.74, -4.28) | -0.40 (-0.42, -0.38)* |
| Yemen | 551.84 (451.17, 674.37) | 1524.77 (1246.40, 1842.49) | 176.31 (159.34, 194.77) | 5677.34 (4727.49, 6815.39) | 5734.02 (4821.11, 6807.63) | 1.00 (-5.13, 7.34) | 0.03 (0.02, 0.04)* |
| Zambia | 221.40 (182.52, 266.34) | 547.49 (449.64, 660.74) | 147.28 (131.04, 165.59) | 4268.65 (3629.05, 5031.63) | 4272.86 (3623.16, 5022.45) | 0.10 (-6.65, 7.14) | 0.00 (-0.02, 0.02) |
| Zimbabwe | 203.59 (168.47, 242.83) | 346.20 (288.83, 412.69) | 70.05 (60.48, 80.36) | 3021.81 (2564.84, 3534.32) | 3140.75 (2699.91, 3692.40) | 3.94 (-2.11, 10.15) | 0.14 (0.11, 0.17)* |

| Table S9 The number and ASPR of MDD in 1990 and 2019, and the corresponding percentage change and AAPC of ASPR during 1990-2019 across 204 countries and territories. | | | | | | | |
| --- | --- | --- | --- | --- | --- | --- | --- |
|  |  |  | Percentage change of |  |  | Percentage change of | AAPC (95% CI) |
| Countries or territories | Number ×10^3^, (95% UI) | | number (%) (95% UI) | ASPR per 100 000 population (95% UI) | | ASPR (%, 95% UI) | of ASPR |
|  | 1990 year | 2019 year | during 1990-2019 | 1990 year | 2019 year | during 1990-2019 | during 1990-2019 |
| Afghanistan | 353.57 (296.59, 423.25) | 1157.57 (955.04, 1399.74) | 227.39 (203.19, 253.89) | 3982.63 (3348.08, 4758.48) | 3942.16 (3316.67, 4665.78) | -1.02 (-6.67, 5.48) | -0.04 (-0.05, -0.02)* |
| Albania | 37.89 (31.63, 45.42) | 44.61 (38.26, 51.86) | 17.72 (4.99, 30.95) | 1286.34 (1095.97, 1512.63) | 1363.65 (1167.78, 1593.64) | 6.01 (-0.67, 13.22) | 0.20 (0.16, 0.25)* |
| Algeria | 654.97 (546.59, 790.74) | 1302.38 (1088.81, 1553.24) | 98.84 (81.30, 117.04) | 3214.48 (2713.70, 3804.93) | 3089.76 (2607.28, 3672.53) | -3.88 (-9.42, 2.01) | -0.14 (-0.17, -0.11)* |
| American Samoa | 0.56 (0.46, 0.68) | 0.71 (0.59, 0.84) | 25.75 (16.22, 35.71) | 1324.97 (1120.48, 1557.24) | 1289.55 (1087.85, 1525.86) | -2.67 (-8.37, 3.71) | -0.10 (-0.14, -0.06)* |
| Andorra | 1.60 (1.35, 1.90) | 2.59 (2.19, 3.09) | 61.96 (48.76, 75.66) | 2619.81 (2246.59, 3120.32) | 2552.79 (2174.04, 3010.50) | -2.56 (-7.73, 3.13) | -0.09 (-0.13, -0.06)* |
| Angola | 359.35 (293.76, 436.23) | 985.06 (802.02, 1184.10) | 174.13 (155.33, 193.17) | 4783.74 (4026.97, 5662.99) | 4552.44 (3799.60, 5360.01) | -4.84 (-11.14, 1.38) | -0.17 (-0.19, -0.15)* |
| Antigua and Barbuda | 1.23 (1.04, 1.47) | 2.15 (1.81, 2.58) | 74.66 (62.65, 88.25) | 2145.07 (1810.74, 2552.56) | 2146.08 (1805.34, 2558.09) | 0.05 (-5.07, 5.07) | 0.00 (-0.09, 0.09) |
| Argentina | 615.08 (525.96, 720.61) | 862.75 (770.14, 963.07) | 40.27 (28.50, 53.05) | 1904.48 (1624.43, 2226.19) | 1808.49 (1614.33, 2022.75) | -5.04 (-12.93, 3.89) | -0.18 (-0.20, -0.16)* |
| Armenia | 55.35 (46.89, 65.17) | 67.75 (57.72, 79.52) | 22.39 (12.04, 33.55) | 1748.04 (1491.06, 2056.37) | 1900.18 (1622.36, 2232.66) | 8.70 (2.23, 15.77) | 0.30 (0.23, 0.37)* |
| Australia | 619.60 (544.03, 703.56) | 898.40 (764.17, 1052.64) | 45.00 (32.52, 58.65) | 3445.99 (3027.83, 3906.14) | 3439.65 (2912.03, 4065.23) | -0.18 (-8.47, 8.85) | -0.02 (-0.07, 0.03) |
| Austria | 227.85 (196.36, 263.16) | 218.72 (189.15, 252.43) | -4.01 (-12.14, 4.50) | 2574.22 (2201.90, 2989.17) | 2051.94 (1758.77, 2370.07) | -20.29 (-26.85, -13.98) | -0.79 (-0.83, -0.74)* |
| Azerbaijan | 106.87 (89.71, 126.96) | 179.62 (151.93, 214.64) | 68.08 (55.16, 81.67) | 1674.53 (1420.06, 1979.42) | 1663.56 (1419.92, 1964.05) | -0.65 (-6.18, 5.45) | -0.04 (-0.14, 0.06) |
| Bahamas | 5.22 (4.38, 6.24) | 9.06 (7.63, 10.78) | 73.34 (59.56, 87.49) | 2217.49 (1890.09, 2619.95) | 2180.24 (1852.71, 2581.05) | -1.68 (-6.91, 4.02) | -0.06 (-0.13, 0.01) |
| Bahrain | 19.69 (16.19, 23.73) | 60.03 (49.17, 71.56) | 204.86 (165.72, 245.51) | 4077.54 (3444.16, 4758.25) | 3550.44 (3018.39, 4168.30) | -12.93 (-19.14, -6.69) | -0.48 (-0.51, -0.46)* |
| Bangladesh | 2919.58 (2458.05, 3464.40) | 5466.49 (4597.96, 6455.85) | 87.24 (70.06, 106.83) | 3666.36 (3114.87, 4330.33) | 3511.53 (2978.50, 4144.24) | -4.22 (-10.89, 3.57) | -0.15 (-0.16, -0.14)* |
| Barbados | 5.97 (5.12, 7.06) | 8.28 (7.01, 9.81) | 38.69 (27.27, 50.48) | 2263.68 (1933.38, 2679.70) | 2300.35 (1949.34, 2718.79) | 1.62 (-3.61, 7.29) | 0.05 (0.01, 0.10)* |
| Belarus | 335.70 (287.52, 392.31) | 336.23 (284.83, 390.02) | 0.16 (-8.62, 9.15) | 2872.96 (2463.48, 3340.58) | 2774.62 (2357.59, 3218.24) | -3.42 (-11.44, 4.14) | -0.11 (-0.15, -0.07)* |
| Belgium | 265.53 (244.78, 287.91) | 339.67 (294.18, 391.71) | 27.92 (15.12, 42.72) | 2327.41 (2137.38, 2533.25) | 2573.08 (2201.59, 2998.00) | 10.56 (-0.61, 23.51) | 0.34 (0.30, 0.38)* |
| Belize | 3.36 (2.83, 4.01) | 9.58 (8.11, 11.36) | 184.91 (161.70, 205.41) | 2334.36 (1991.27, 2704.65) | 2438.27 (2093.46, 2863.36) | 4.45 (-2.38, 10.49) | 0.14 (0.11, 0.18)* |
| Benin | 95.34 (80.08, 113.35) | 270.67 (227.13, 321.08) | 183.91 (167.78, 201.90) | 3082.60 (2629.77, 3621.76) | 3195.24 (2710.75, 3738.49) | 3.65 (-2.39, 10.57) | 0.12 (0.11, 0.14)* |
| Bermuda | 1.78 (1.51, 2.09) | 1.89 (1.61, 2.22) | 6.12 (-4.74, 17.98) | 2721.31 (2320.32, 3182.31) | 2301.68 (1967.85, 2691.01) | -15.42 (-22.16, -8.75) | -0.58 (-0.60, -0.56)* |
| Bhutan | 13.31 (11.13, 15.92) | 21.08 (17.72, 25.07) | 58.37 (46.32, 71.39) | 3150.56 (2687.46, 3747.96) | 2901.86 (2446.42, 3431.53) | -7.89 (-14.06, -1.41) | -0.29 (-0.33, -0.25)* |
| Bolivia (Plurinational State of) | 137.63 (115.26, 163.58) | 276.08 (233.73, 326.16) | 100.59 (87.92, 116.00) | 2821.04 (2391.08, 3311.15) | 2536.20 (2156.66, 2980.77) | -10.10 (-15.57, -3.69) | -0.37 (-0.40, -0.34)* |
| Bosnia and Herzegovina | 103.66 (88.54, 121.38) | 72.17 (60.66, 86.30) | -30.38 (-39.13, -21.04) | 2206.73 (1897.85, 2564.49) | 1674.57 (1424.91, 1990.72) | -24.12 (-31.51, -15.83) | -0.95 (-1.04, -0.85)* |
| Botswana | 26.79 (22.42, 31.99) | 63.55 (52.57, 76.06) | 137.22 (118.56, 158.18) | 2890.01 (2447.51, 3385.73) | 2994.15 (2538.18, 3529.52) | 3.60 (-3.00, 10.82) | 0.12 (0.10, 0.14)* |
| Brazil | 4238.56 (3679.57, 4842.27) | 6950.79 (6236.65, 7704.55) | 63.99 (54.85, 72.47) | 3195.87 (2799.42, 3612.80) | 2976.69 (2671.48, 3304.09) | -6.86 (-11.03, -2.40) | -0.25 (-0.35, -0.15)* |
| Brunei Darussalam | 2.24 (1.84, 2.71) | 4.44 (3.68, 5.30) | 98.47 (82.89, 114.95) | 972.04 (834.13, 1153.37) | 974.17 (826.04, 1147.42) | 0.22 (-5.66, 6.56) | 0.00 (-0.03, 0.03) |
| Bulgaria | 196.29 (169.33, 227.29) | 155.07 (131.12, 180.81) | -21.00 (-28.97, -11.53) | 1915.64 (1654.76, 2212.96) | 1603.96 (1365.55, 1877.73) | -16.27 (-23.85, -7.74) | -0.61 (-0.64, -0.59)* |
| Burkina Faso | 197.85 (167.24, 232.67) | 471.83 (397.01, 554.48) | 138.48 (124.75, 154.76) | 3197.04 (2735.80, 3734.05) | 3113.80 (2664.14, 3623.29) | -2.60 (-8.37, 4.12) | -0.05 (-0.15, 0.04) |
| Burundi | 155.59 (129.63, 184.91) | 271.87 (227.63, 323.40) | 74.73 (62.94, 86.77) | 4240.88 (3587.89, 5000.87) | 3367.91 (2880.08, 3987.37) | -20.58 (-26.09, -14.94) | -0.80 (-0.82, -0.78)* |
| Cabo Verde | 8.66 (7.32, 10.14) | 19.42 (16.43, 22.93) | 124.16 (105.78, 141.97) | 3331.60 (2827.44, 3921.07) | 3620.48 (3102.50, 4244.78) | 8.67 (1.57, 15.99) | 0.30 (0.27, 0.32)* |
| Cambodia | 151.19 (123.78, 183.90) | 253.48 (213.45, 305.82) | 67.66 (54.32, 82.89) | 1865.48 (1561.34, 2204.83) | 1573.67 (1336.23, 1879.68) | -15.64 (-21.70, -9.35) | -0.59 (-0.61, -0.56)* |
| Cameroon | 226.75 (189.32, 270.68) | 703.80 (590.57, 831.11) | 210.39 (192.44, 232.38) | 3237.92 (2753.24, 3798.39) | 3364.63 (2897.22, 3890.90) | 3.91 (-2.48, 10.31) | 0.13 (0.12, 0.15)* |
| Canada | 635.40 (562.74, 722.74) | 804.69 (702.07, 916.27) | 26.64 (18.55, 35.70) | 2148.59 (1892.81, 2441.63) | 2143.73 (1840.98, 2482.08) | -0.23 (-7.81, 7.44) | -0.02 (-0.12, 0.08) |
| Central African Republic | 99.09 (80.48, 119.07) | 194.78 (159.87, 233.80) | 96.57 (85.54, 108.63) | 4833.15 (4030.27, 5678.38) | 4747.32 (4010.17, 5594.53) | -1.78 (-7.14, 4.12) | -0.06 (-0.07, -0.05)* |
| Chad | 151.50 (127.36, 180.05) | 391.15 (325.78, 464.92) | 158.18 (141.59, 175.97) | 3807.37 (3248.75, 4460.83) | 3922.70 (3344.92, 4583.80) | 3.03 (-3.58, 10.21) | 0.11 (0.08, 0.14)* |
| Chile | 479.66 (433.70, 527.53) | 601.23 (512.45, 700.32) | 25.34 (12.60, 40.33) | 3742.47 (3383.70, 4094.41) | 2993.27 (2550.91, 3494.28) | -20.02 (-28.13, -10.84) | -0.74 (-0.84, -0.64)* |
| China | 19478.75 (16786.07, 22341.42) | 25335.78 (22408.49, 28702.55) | 30.07 (17.90, 42.76) | 1649.27 (1445.90, 1862.14) | 1412.69 (1256.12, 1592.99) | -14.34 (-18.33, -10.22) | -0.58 (-0.73, -0.43)* |
| Colombia | 449.15 (375.33, 534.94) | 725.93 (639.10, 819.97) | 61.62 (44.00, 79.92) | 1613.05 (1381.50, 1889.76) | 1412.10 (1244.86, 1593.42) | -12.46 (-20.25, -5.17) | -0.46 (-0.53, -0.38)* |
| Comoros | 9.76 (8.28, 11.57) | 18.11 (15.20, 21.69) | 85.51 (72.72, 100.96) | 3057.97 (2613.72, 3595.86) | 2899.68 (2459.54, 3445.36) | -5.18 (-11.37, 2.22) | -0.18 (-0.21, -0.16)* |
| Congo | 85.95 (70.72, 103.85) | 192.00 (159.60, 231.76) | 123.39 (106.84, 140.71) | 4779.76 (4019.75, 5629.98) | 4331.60 (3646.56, 5099.58) | -9.38 (-14.95, -3.78) | -0.34 (-0.36, -0.32)* |
| Cook Islands | 0.31 (0.24, 0.40) | 0.34 (0.28, 0.43) | 10.98 (1.79, 21.55) | 1782.75 (1424.03, 2235.75) | 1801.07 (1419.02, 2259.87) | 1.03 (-3.65, 5.21) | 0.03 (0.02, 0.04)* |
| Costa Rica | 57.92 (48.94, 68.60) | 122.84 (104.45, 144.58) | 112.07 (95.68, 128.12) | 2262.98 (1930.99, 2665.67) | 2394.16 (2044.36, 2820.19) | 5.80 (-0.93, 12.11) | 0.20 (0.18, 0.21)* |
| Croatia | 136.84 (118.55, 158.34) | 112.50 (96.47, 133.13) | -17.79 (-25.48, -7.96) | 2407.75 (2097.82, 2775.21) | 1929.54 (1648.38, 2235.00) | -19.86 (-26.50, -11.81) | -0.76 (-0.80, -0.72)* |
| Cuba | 466.33 (396.35, 548.22) | 426.93 (363.02, 497.32) | -8.45 (-19.50, 3.07) | 4082.67 (3494.51, 4758.09) | 2953.17 (2526.56, 3420.91) | -27.67 (-34.37, -20.98) | -1.12 (-1.15, -1.09)* |
| Cyprus | 16.40 (13.95, 19.38) | 30.51 (25.85, 36.09) | 85.97 (72.60, 99.79) | 2038.05 (1729.28, 2407.78) | 2036.17 (1725.36, 2416.16) | -0.09 (-5.26, 5.38) | -0.01 (-0.08, 0.06) |
| Czechia | 260.33 (224.35, 302.37) | 249.46 (215.31, 289.13) | -4.17 (-13.10, 4.80) | 2212.59 (1909.00, 2563.51) | 1798.06 (1550.71, 2080.51) | -18.74 (-25.33, -11.66) | -0.71 (-0.73, -0.70)* |
| Côte d’Ivoire | 211.41 (173.29, 254.23) | 508.36 (424.07, 603.53) | 140.46 (125.39, 156.03) | 2665.51 (2257.10, 3115.50) | 2673.97 (2282.19, 3130.70) | 0.32 (-5.26, 6.75) | 0.00 (-0.01, 0.02) |
| Democratic People's Republic of Korea | 294.93 (247.84, 350.59) | 393.41 (333.04, 468.79) | 33.39 (23.60, 44.86) | 1500.16 (1276.75, 1763.98) | 1298.66 (1105.77, 1537.78) | -13.43 (-19.07, -7.56) | -0.50 (-0.52, -0.48)* |
| Democratic Republic of the Congo | 1208.20 (995.75, 1458.61) | 2822.74 (2308.40, 3424.89) | 133.63 (119.56, 149.17) | 4392.02 (3698.19, 5193.63) | 4242.75 (3594.11, 5018.10) | -3.40 (-8.79, 2.45) | -0.12 (-0.13, -0.11)* |
| Denmark | 202.74 (175.32, 236.04) | 168.44 (145.18, 194.72) | -16.92 (-24.40, -8.28) | 3371.83 (2896.67, 3916.55) | 2421.92 (2079.25, 2811.21) | -28.17 (-34.13, -21.11) | -1.16 (-1.24, -1.07)* |
| Djibouti | 9.77 (8.05, 11.76) | 30.97 (25.60, 37.17) | 217.09 (190.25, 243.03) | 3109.23 (2642.29, 3650.28) | 3126.70 (2640.28, 3711.89) | 0.56 (-5.57, 6.70) | 0.02 (0.01, 0.03)* |
| Dominica | 1.50 (1.27, 1.77) | 1.70 (1.45, 1.99) | 12.98 (5.87, 20.87) | 2189.83 (1864.04, 2587.48) | 2209.42 (1889.64, 2613.40) | 0.89 (-4.68, 5.99) | 0.03 (-0.01, 0.07) |
| Dominican Republic | 173.23 (144.08, 208.06) | 317.95 (270.28, 375.33) | 83.54 (69.56, 99.64) | 2914.34 (2484.31, 3441.71) | 2973.72 (2539.58, 3493.87) | 2.04 (-4.00, 8.92) | 0.06 (0.02, 0.11)* |
| Ecuador | 203.62 (172.14, 243.01) | 425.36 (362.74, 499.32) | 108.90 (90.11, 127.89) | 2409.22 (2073.05, 2819.57) | 2459.66 (2108.58, 2871.78) | 2.09 (-4.95, 9.95) | 0.07 (0.06, 0.08)* |
| Egypt | 1282.95 (1067.07, 1544.59) | 2679.30 (2245.32, 3212.84) | 108.84 (96.28, 123.08) | 2738.58 (2323.65, 3253.48) | 2899.60 (2467.29, 3443.91) | 5.88 (-0.27, 12.09) | 0.18 (0.03, 0.32)* |
| El Salvador | 128.21 (107.89, 153.25) | 163.46 (139.32, 193.12) | 27.50 (17.43, 37.86) | 2908.79 (2484.26, 3444.67) | 2590.93 (2210.10, 3069.54) | -10.93 (-17.20, -5.09) | -0.40 (-0.43, -0.38)* |
| Equatorial Guinea | 14.90 (12.35, 17.81) | 50.62 (41.11, 61.80) | 239.78 (215.92, 266.07) | 4887.32 (4125.14, 5774.40) | 4549.81 (3816.72, 5417.94) | -6.91 (-12.38, -0.96) | -0.25 (-0.27, -0.24)* |
| Eritrea | 70.97 (58.64, 84.86) | 172.66 (142.63, 205.31) | 143.28 (127.57, 160.58) | 3708.35 (3160.32, 4348.76) | 3505.44 (3001.30, 4135.40) | -5.47 (-11.28, 1.20) | -0.19 (-0.20, -0.18)* |
| Estonia | 65.45 (56.57, 75.58) | 47.24 (40.24, 55.18) | -27.82 (-34.98, -19.92) | 3669.90 (3186.65, 4237.76) | 2641.92 (2280.89, 3076.11) | -28.01 (-34.32, -20.95) | -1.14 (-1.21, -1.07)* |
| Eswatini | 15.19 (12.65, 18.30) | 29.56 (24.74, 35.14) | 94.61 (81.78, 108.93) | 2867.98 (2419.39, 3396.88) | 3162.31 (2678.73, 3714.61) | 10.26 (3.39, 18.24) | 0.34 (0.30, 0.38)* |
| Ethiopia | 1249.93 (1059.18, 1464.04) | 2468.76 (2088.76, 2899.31) | 97.51 (93.27, 102.30) | 3757.75 (3252.79, 4325.04) | 3343.36 (2910.46, 3852.49) | -11.03 (-13.04, -9.14) | -0.40 (-0.43, -0.38)* |
| Fiji | 10.51 (8.74, 12.62) | 14.07 (11.81, 16.76) | 33.86 (23.72, 44.26) | 1546.43 (1296.06, 1813.83) | 1560.27 (1322.77, 1841.47) | 0.90 (-5.37, 7.04) | 0.03 (0.02, 0.03)* |
| Finland | 208.46 (180.06, 243.62) | 183.86 (161.71, 208.16) | -11.80 (-19.96, -3.28) | 3786.35 (3265.73, 4429.98) | 2961.68 (2564.45, 3411.23) | -21.78 (-28.47, -14.40) | -0.86 (-0.98, -0.73)* |
| France | 2208.24 (2029.19, 2368.97) | 2150.00 (1848.57, 2481.05) | -2.64 (-13.70, 8.88) | 3414.94 (3138.16, 3674.05) | 2786.65 (2384.45, 3246.67) | -18.40 (-27.90, -8.63) | -0.68 (-0.78, -0.59)* |
| Gabon | 35.40 (29.60, 42.34) | 68.35 (56.71, 81.49) | 93.10 (80.68, 107.89) | 4563.28 (3844.30, 5356.79) | 4343.59 (3678.74, 5100.07) | -4.81 (-10.25, 1.72) | -0.17 (-0.19, -0.15)* |
| Gambia | 28.08 (23.19, 33.88) | 69.87 (58.42, 83.83) | 148.87 (133.23, 164.33) | 4372.67 (3758.80, 5069.64) | 4334.63 (3705.40, 5111.16) | -0.87 (-7.08, 5.62) | -0.04 (-0.09, 0.00) |
| Georgia | 132.09 (112.43, 153.92) | 104.20 (89.34, 121.08) | -21.12 (-26.84, -15.11) | 2227.31 (1910.11, 2599.15) | 2253.25 (1924.07, 2627.41) | 1.16 (-5.24, 7.72) | 0.04 (0.00, 0.09)* |
| Germany | 2042.95 (1845.38, 2264.91) | 2469.06 (2147.72, 2845.08) | 20.86 (9.72, 32.80) | 2178.43 (1959.96, 2419.82) | 2392.28 (2055.81, 2792.87) | 9.82 (-0.16, 20.11) | 0.35 (0.21, 0.48)* |
| Ghana | 322.70 (271.07, 384.39) | 785.51 (656.05, 930.42) | 143.42 (130.02, 157.59) | 3099.50 (2654.16, 3630.09) | 3098.99 (2639.01, 3636.74) | -0.02 (-5.32, 5.32) | -0.00 (-0.01, 0.00) |
| Greece | 475.92 (407.23, 555.86) | 519.39 (444.34, 608.85) | 9.13 (1.05, 19.37) | 3996.67 (3401.05, 4710.80) | 4047.31 (3432.76, 4801.60) | 1.27 (-4.70, 9.23) | 0.02 (-0.11, 0.15) |
| Greenland | 3.43 (2.88, 4.09) | 2.84 (2.40, 3.32) | -17.09 (-23.82, -9.90) | 5434.02 (4614.61, 6362.23) | 4836.36 (4080.88, 5675.92) | -11.00 (-17.16, -4.89) | -0.39 (-0.44, -0.35)* |
| Grenada | 1.75 (1.49, 2.06) | 2.67 (2.28, 3.13) | 52.30 (42.04, 63.46) | 2359.03 (2016.03, 2775.21) | 2363.16 (2030.48, 2762.93) | 0.18 (-5.04, 5.84) | 0.01 (-0.04, 0.05) |
| Guam | 2.51 (2.09, 3.03) | 3.23 (2.76, 3.81) | 28.74 (17.82, 41.37) | 1832.85 (1546.92, 2166.16) | 1853.91 (1572.40, 2183.26) | 1.15 (-4.95, 8.80) | 0.04 (0.03, 0.05)* |
| Guatemala | 169.13 (142.05, 199.07) | 446.49 (369.10, 526.63) | 163.99 (148.25, 183.70) | 2993.11 (2548.72, 3521.19) | 2866.84 (2412.61, 3369.32) | -4.22 (-10.35, 2.94) | -0.13 (-0.22, -0.04)* |
| Guinea | 130.66 (110.22, 153.89) | 271.35 (229.29, 321.42) | 107.68 (95.09, 120.71) | 2973.31 (2527.78, 3488.04) | 3113.12 (2644.72, 3657.87) | 4.70 (-2.12, 11.56) | 0.16 (0.15, 0.17)* |
| Guinea-Bissau | 20.65 (17.18, 24.63) | 43.64 (36.19, 52.18) | 111.35 (99.21, 123.36) | 3099.20 (2637.42, 3663.64) | 3241.87 (2766.64, 3805.23) | 4.60 (-1.73, 11.44) | 0.15 (0.14, 0.17)* |
| Guyana | 25.15 (21.20, 30.24) | 32.15 (26.89, 37.77) | 27.84 (16.89, 39.27) | 3778.76 (3212.85, 4437.03) | 4122.00 (3497.38, 4790.86) | 9.08 (0.97, 17.08) | 0.31 (0.27, 0.35)* |
| Haiti | 145.49 (121.57, 174.56) | 306.82 (253.00, 370.74) | 110.89 (99.40, 123.17) | 2866.55 (2435.34, 3371.01) | 2770.94 (2343.67, 3314.10) | -3.34 (-8.66, 1.93) | -0.12 (-0.13, -0.10)* |
| Honduras | 71.65 (60.00, 84.34) | 195.61 (165.82, 231.58) | 173.00 (155.78, 192.01) | 2216.50 (1903.27, 2581.50) | 2337.69 (1994.76, 2736.30) | 5.47 (-0.67, 11.53) | 0.19 (0.17, 0.21)* |
| Hungary | 284.63 (246.64, 327.90) | 230.36 (197.81, 269.68) | -19.07 (-26.75, -9.25) | 2349.02 (2037.34, 2714.48) | 1737.43 (1508.97, 2019.35) | -26.04 (-32.37, -18.46) | -1.02 (-1.08, -0.97)* |
| Iceland | 5.65 (4.88, 6.55) | 7.15 (6.17, 8.30) | 26.50 (16.09, 37.15) | 2136.82 (1839.00, 2475.35) | 1858.01 (1590.33, 2175.73) | -13.05 (-19.48, -6.26) | -0.48 (-0.52, -0.45)* |
| India | 20041.93 (17262.52, 23043.12) | 34539.45 (30318.12, 39153.37) | 72.34 (65.05, 78.85) | 2919.48 (2555.97, 3332.83) | 2547.78 (2254.11, 2880.01) | -12.73 (-15.45, -10.21) | -0.48 (-0.58, -0.39)* |
| Indonesia | 1787.46 (1507.58, 2123.67) | 2896.37 (2452.89, 3379.46) | 62.04 (54.93, 69.91) | 1083.78 (922.30, 1257.06) | 1071.86 (912.06, 1244.28) | -1.10 (-2.54, 0.35) | -0.04 (-0.07, -0.01)* |
| Iran (Islamic Republic of) | 1714.67 (1409.85, 2088.33) | 3517.47 (2911.50, 4227.86) | 105.14 (87.56, 122.49) | 3665.83 (3054.67, 4368.67) | 3863.95 (3221.08, 4592.02) | 5.40 (3.72, 7.08) | 0.20 (0.14, 0.26)* |
| Iraq | 388.61 (320.86, 466.70) | 1146.80 (949.52, 1358.52) | 195.10 (173.42, 216.05) | 2873.63 (2412.17, 3403.14) | 2885.58 (2434.82, 3382.16) | 0.42 (-5.96, 7.06) | 0.04 (-0.03, 0.10) |
| Ireland | 108.27 (94.14, 123.97) | 169.12 (150.24, 189.26) | 56.20 (43.78, 70.17) | 2966.25 (2573.47, 3410.08) | 3149.37 (2775.00, 3593.67) | 6.17 (-2.63, 15.71) | 0.27 (0.13, 0.41)* |
| Israel | 160.54 (137.77, 187.52) | 292.95 (253.99, 340.96) | 82.48 (69.74, 97.03) | 3338.96 (2868.99, 3895.48) | 3079.58 (2652.71, 3597.53) | -7.77 (-14.25, -0.86) | -0.31 (-0.36, -0.25)* |
| Italy | 1844.08 (1602.14, 2109.21) | 1946.78 (1694.84, 2208.88) | 5.57 (1.43, 9.63) | 2736.14 (2360.51, 3148.64) | 2562.71 (2210.39, 2948.50) | -6.34 (-7.73, -4.90) | -0.24 (-0.32, -0.16)* |
| Jamaica | 45.15 (37.92, 54.23) | 66.38 (56.20, 78.76) | 47.03 (36.42, 58.25) | 2150.84 (1819.84, 2565.39) | 2185.44 (1852.25, 2589.49) | 1.61 (-3.72, 7.42) | 0.05 (0.01, 0.09)* |
| Japan | 2050.44 (1831.50, 2285.79) | 2368.99 (2134.11, 2621.19) | 15.54 (10.62, 20.60) | 1442.35 (1282.31, 1617.36) | 1547.20 (1375.85, 1738.74) | 7.27 (5.28, 9.46) | 0.25 (0.19, 0.32)* |
| Jordan | 102.69 (84.10, 123.87) | 346.29 (288.48, 420.76) | 237.22 (210.37, 263.45) | 3546.66 (2988.80, 4187.52) | 3131.06 (2647.06, 3747.99) | -11.72 (-18.38, -4.82) | -0.44 (-0.48, -0.40)* |
| Kazakhstan | 363.69 (311.37, 424.30) | 436.71 (373.56, 508.73) | 20.08 (10.38, 30.25) | 2430.01 (2089.09, 2818.09) | 2370.43 (2049.01, 2759.66) | -2.45 (-9.54, 5.56) | -0.07 (-0.10, -0.04)* |
| Kenya | 527.14 (455.94, 604.10) | 1283.13 (1117.73, 1470.50) | 143.41 (138.37, 148.40) | 3704.09 (3274.17, 4189.72) | 3411.80 (3023.29, 3863.07) | -7.89 (-9.11, -6.75) | -0.28 (-0.32, -0.25)* |
| Kiribati | 1.15 (0.92, 1.41) | 1.73 (1.45, 2.10) | 51.38 (40.88, 62.91) | 1766.01 (1471.96, 2095.68) | 1592.66 (1341.48, 1890.48) | -9.82 (-16.06, -3.22) | -0.36 (-0.37, -0.34)* |
| Kuwait | 48.80 (39.57, 59.63) | 152.10 (123.09, 183.92) | 211.68 (184.86, 244.43) | 2907.54 (2449.78, 3437.75) | 3031.62 (2548.72, 3608.07) | 4.27 (-1.44, 11.07) | 0.14 (0.11, 0.18)* |
| Kyrgyzstan | 95.51 (81.15, 111.79) | 136.13 (114.40, 160.12) | 42.53 (31.02, 55.50) | 2593.60 (2233.27, 3040.19) | 2313.01 (1970.28, 2711.52) | -10.82 (-17.61, -3.15) | -0.40 (-0.42, -0.38)* |
| Lao People's Democratic Republic | 53.35 (43.84, 64.89) | 96.16 (79.80, 115.52) | 80.24 (67.33, 95.35) | 1575.28 (1318.67, 1869.63) | 1378.27 (1161.66, 1621.54) | -12.51 (-18.61, -5.64) | -0.46 (-0.52, -0.41)* |
| Latvia | 103.17 (87.50, 120.54) | 70.09 (61.19, 79.67) | -32.06 (-38.89, -23.84) | 3337.82 (2846.27, 3876.33) | 2694.12 (2367.97, 3066.79) | -19.29 (-25.93, -11.11) | -0.74 (-0.78, -0.70)* |
| Lebanon | 97.01 (81.33, 115.05) | 192.17 (160.29, 227.24) | 98.09 (78.81, 116.71) | 3404.66 (2876.70, 4006.87) | 3625.45 (3037.81, 4288.61) | 6.49 (-3.22, 15.28) | 0.23 (0.17, 0.29)* |
| Lesotho | 58.17 (48.64, 69.81) | 79.64 (67.13, 94.23) | 36.92 (24.29, 48.63) | 4239.75 (3585.55, 4987.52) | 4318.07 (3688.70, 5069.34) | 1.85 (-6.12, 10.13) | 0.05 (-0.01, 0.11) |
| Liberia | 47.36 (39.85, 55.92) | 121.02 (100.22, 143.90) | 155.53 (138.36, 176.03) | 3344.99 (2837.95, 3922.03) | 3330.32 (2836.79, 3911.51) | -0.44 (-7.54, 7.08) | 0.02 (-0.04, 0.09) |
| Libya | 107.48 (88.90, 130.00) | 249.34 (208.44, 300.88) | 131.98 (112.17, 156.05) | 3208.03 (2719.34, 3819.38) | 3381.68 (2866.94, 4058.66) | 5.41 (0.21, 11.45) | 0.18 (0.16, 0.20)* |
| Lithuania | 131.56 (113.34, 152.13) | 110.01 (94.32, 127.70) | -16.38 (-24.01, -7.85) | 3224.95 (2775.12, 3710.45) | 2946.68 (2537.07, 3416.06) | -8.63 (-15.39, -0.78) | -0.31 (-0.34, -0.28)* |
| Luxembourg | 12.17 (10.50, 14.16) | 15.52 (13.64, 17.68) | 27.51 (16.02, 38.69) | 2786.41 (2398.57, 3241.72) | 2146.91 (1884.28, 2458.97) | -22.95 (-29.74, -16.51) | -0.90 (-0.94, -0.86)* |
| Madagascar | 284.85 (236.15, 341.01) | 657.72 (547.79, 778.92) | 130.90 (115.73, 146.30) | 3534.68 (2999.50, 4170.06) | 3394.64 (2896.69, 3965.64) | -3.96 (-10.02, 2.44) | -0.14 (-0.15, -0.13)* |
| Malawi | 185.53 (153.89, 222.37) | 350.10 (290.55, 418.62) | 88.71 (76.87, 101.82) | 2902.78 (2470.82, 3422.69) | 2717.28 (2302.13, 3201.95) | -6.39 (-12.18, 0.13) | -0.23 (-0.26, -0.20)* |
| Malaysia | 290.01 (241.22, 344.38) | 756.45 (649.28, 884.41) | 160.83 (136.01, 192.64) | 1939.11 (1643.61, 2270.57) | 2335.49 (2012.69, 2708.26) | 20.44 (8.97, 34.40) | 0.61 (0.52, 0.71)* |
| Maldives | 3.79 (3.14, 4.52) | 8.25 (6.88, 9.82) | 117.84 (96.58, 139.70) | 2233.32 (1889.04, 2589.81) | 1609.25 (1370.64, 1876.82) | -27.94 (-32.66, -22.79) | -1.13 (-1.17, -1.09)* |
| Mali | 137.05 (115.16, 163.13) | 326.27 (271.51, 392.38) | 138.07 (120.72, 155.05) | 2318.45 (1976.38, 2722.69) | 2251.26 (1914.37, 2659.21) | -2.90 (-9.52, 3.73) | -0.11 (-0.15, -0.07)* |
| Malta | 8.31 (7.12, 9.76) | 10.69 (9.17, 12.59) | 28.62 (19.81, 38.82) | 2112.74 (1810.37, 2492.57) | 2057.93 (1748.86, 2426.01) | -2.59 (-8.15, 2.69) | -0.10 (-0.13, -0.07)* |
| Marshall Islands | 0.56 (0.45, 0.69) | 0.83 (0.68, 1.00) | 47.91 (36.90, 59.97) | 1617.84 (1348.56, 1929.13) | 1533.01 (1281.46, 1821.46) | -5.24 (-10.11, 0.31) | -0.19 (-0.20, -0.17)* |
| Mauritania | 35.77 (30.15, 42.14) | 72.06 (60.49, 86.37) | 101.45 (87.87, 115.79) | 2477.88 (2096.20, 2897.91) | 2365.99 (2014.50, 2792.26) | -4.52 (-11.01, 2.37) | -0.17 (-0.21, -0.13)* |
| Mauritius | 31.34 (26.39, 37.48) | 36.34 (30.76, 42.60) | 15.96 (5.21, 28.19) | 2884.60 (2457.32, 3390.22) | 2476.14 (2083.91, 2904.48) | -14.16 (-20.03, -7.94) | -0.53 (-0.56, -0.50)* |
| Mexico | 1480.38 (1287.82, 1689.38) | 3483.05 (3031.44, 3954.16) | 135.28 (125.30, 146.04) | 2198.94 (1938.35, 2477.72) | 2703.54 (2367.50, 3061.78) | 22.95 (20.13, 26.11) | 0.72 (0.68, 0.75)* |
| Micronesia (Federated States of) | 1.38 (1.13, 1.69) | 1.54 (1.27, 1.86) | 11.18 (2.74, 20.87) | 1668.71 (1409.53, 1987.57) | 1548.96 (1302.86, 1845.15) | -7.18 (-12.99, -1.27) | -0.26 (-0.27, -0.25)* |
| Monaco | 1.14 (0.93, 1.38) | 1.39 (1.13, 1.69) | 21.69 (15.33, 28.53) | 3025.87 (2441.40, 3753.54) | 3015.65 (2421.05, 3730.22) | -0.34 (-4.54, 3.51) | -0.01 (-0.02, -0.01)* |
| Mongolia | 48.68 (40.55, 58.27) | 93.58 (78.35, 109.69) | 92.23 (75.20, 111.01) | 2931.48 (2508.47, 3436.02) | 2916.72 (2471.10, 3394.67) | -0.50 (-6.91, 5.94) | -0.02 (-0.04, 0.01) |
| Montenegro | 11.47 (9.79, 13.41) | 13.25 (11.24, 15.45) | 15.51 (5.27, 26.50) | 1780.31 (1521.03, 2073.85) | 1734.18 (1479.18, 2011.99) | -2.59 (-9.27, 4.37) | -0.08 (-0.10, -0.06)* |
| Morocco | 894.91 (739.67, 1072.49) | 1518.27 (1275.98, 1808.37) | 69.66 (55.71, 84.51) | 4191.20 (3526.89, 4953.60) | 4082.09 (3463.69, 4843.56) | -2.60 (-9.21, 4.43) | -0.09 (-0.10, -0.08)* |
| Mozambique | 312.07 (261.48, 369.36) | 703.24 (584.47, 833.78) | 125.35 (110.31, 140.80) | 3478.56 (2950.26, 4054.84) | 3645.96 (3116.37, 4290.52) | 4.81 (-2.80, 12.42) | 0.16 (0.14, 0.17)* |
| Myanmar | 289.64 (238.79, 348.63) | 438.45 (363.83, 522.96) | 51.38 (40.85, 62.58) | 807.64 (682.15, 956.89) | 792.31 (664.96, 937.02) | -1.90 (-7.09, 3.85) | -0.09 (-0.26, 0.07) |
| Namibia | 24.87 (20.89, 29.52) | 47.26 (39.72, 55.99) | 89.99 (76.67, 103.70) | 2361.61 (2011.07, 2765.75) | 2318.76 (1992.50, 2707.36) | -1.81 (-7.87, 5.07) | -0.08 (-0.14, -0.02)* |
| Nauru | 0.15 (0.11, 0.19) | 0.17 (0.13, 0.22) | 14.40 (8.00, 20.26) | 1770.06 (1400.49, 2221.57) | 1788.54 (1424.95, 2254.60) | 1.04 (-3.94, 5.70) | 0.03 (0.02, 0.04)* |
| Nepal | 496.48 (416.77, 587.84) | 1043.32 (899.16, 1211.86) | 110.14 (94.40, 127.30) | 3550.56 (3018.75, 4166.40) | 3795.90 (3265.67, 4408.13) | 6.91 (-0.08, 14.79) | 0.23 (0.17, 0.29)* |
| Netherlands | 448.39 (413.22, 482.94) | 508.82 (440.57, 590.35) | 13.48 (2.35, 26.60) | 2634.91 (2416.68, 2843.97) | 2524.50 (2173.93, 2931.56) | -4.19 (-14.15, 7.51) | -0.12 (-0.17, -0.06)* |
| New Zealand | 92.27 (76.36, 110.37) | 120.46 (104.95, 138.55) | 30.55 (21.02, 41.45) | 2552.69 (2113.68, 3058.18) | 2716.81 (2336.77, 3178.29) | 6.43 (-0.54, 14.76) | 0.21 (0.20, 0.22)* |
| Nicaragua | 68.15 (56.68, 81.01) | 149.76 (126.67, 177.09) | 119.77 (102.98, 138.67) | 2400.88 (2044.77, 2809.75) | 2437.25 (2091.60, 2854.61) | 1.52 (-4.19, 8.64) | 0.06 (0.03, 0.08)* |
| Niger | 144.58 (120.49, 172.06) | 412.03 (343.92, 489.18) | 184.98 (166.79, 203.88) | 2894.93 (2470.28, 3386.79) | 2974.65 (2525.04, 3490.33) | 2.75 (-3.94, 9.08) | 0.09 (0.08, 0.10)* |
| Nigeria | 1944.02 (1640.21, 2257.62) | 3744.74 (3185.28, 4360.69) | 92.63 (89.55, 96.21) | 3027.07 (2615.35, 3482.92) | 2559.15 (2215.93, 2917.97) | -15.46 (-16.78, -13.98) | -0.57 (-0.64, -0.51)* |
| Niue | 0.04 (0.03, 0.05) | 0.03 (0.03, 0.04) | -16.61 (-21.78, -11.14) | 1792.48 (1419.61, 2248.08) | 1788.61 (1415.55, 2246.74) | -0.22 (-4.21, 4.13) | -0.01 (-0.02, -0.00)* |
| North Macedonia | 32.93 (27.86, 38.36) | 39.29 (33.19, 45.79) | 19.30 (9.23, 30.98) | 1624.57 (1380.16, 1890.93) | 1481.49 (1259.73, 1730.03) | -8.81 (-15.51, -0.99) | -0.32 (-0.37, -0.27)* |
| Northern Mariana Islands | 0.62 (0.51, 0.75) | 0.69 (0.58, 0.82) | 10.77 (-3.16, 25.39) | 1337.36 (1143.74, 1579.21) | 1429.49 (1213.71, 1685.40) | 6.89 (0.85, 13.41) | 0.23 (0.17, 0.29)* |
| Norway | 89.60 (77.28, 102.68) | 130.42 (112.22, 150.53) | 45.55 (42.04, 49.17) | 1912.22 (1637.77, 2209.10) | 2199.63 (1870.09, 2563.98) | 15.03 (12.84, 17.09) | 0.47 (0.30, 0.64)* |
| Oman | 46.09 (37.98, 55.56) | 141.58 (113.70, 174.46) | 207.20 (181.57, 234.58) | 2945.96 (2485.52, 3485.13) | 2973.58 (2517.74, 3514.19) | 0.94 (-5.08, 7.16) | 0.03 (-0.04, 0.10) |
| Pakistan | 2312.76 (1962.10, 2720.58) | 4874.51 (4108.90, 5753.52) | 110.77 (104.72, 116.80) | 2956.17 (2535.60, 3441.95) | 2830.09 (2433.75, 3296.50) | -4.26 (-6.75, -1.74) | -0.15 (-0.19, -0.11)* |
| Palau | 0.27 (0.21, 0.34) | 0.36 (0.28, 0.44) | 33.43 (19.25, 49.11) | 1778.69 (1406.03, 2225.65) | 1764.41 (1403.82, 2207.81) | -0.80 (-5.14, 4.07) | -0.03 (-0.06, -0.01)* |
| Palestine | 79.80 (66.69, 95.83) | 226.67 (191.04, 270.70) | 184.04 (167.99, 202.30) | 5354.27 (4534.38, 6309.11) | 5259.45 (4493.51, 6188.36) | -1.77 (-7.00, 3.30) | -0.06 (-0.14, 0.01) |
| Panama | 44.39 (37.36, 52.36) | 89.09 (75.48, 104.28) | 100.72 (87.75, 117.25) | 2150.44 (1834.06, 2528.56) | 2115.24 (1790.63, 2476.80) | -1.64 (-7.02, 3.75) | -0.06 (-0.06, -0.05)* |
| Papua New Guinea | 64.82 (52.71, 79.30) | 153.58 (125.55, 186.91) | 136.92 (123.10, 150.98) | 1840.14 (1536.43, 2195.15) | 1729.16 (1448.86, 2064.07) | -6.03 (-10.78, -1.35) | -0.22 (-0.23, -0.21)* |
| Paraguay | 89.11 (73.90, 106.33) | 195.74 (165.52, 230.55) | 119.66 (103.46, 136.82) | 2616.80 (2224.60, 3086.79) | 2829.56 (2412.29, 3327.63) | 8.13 (0.96, 14.95) | 0.27 (0.25, 0.29)* |
| Peru | 271.63 (227.79, 322.75) | 449.34 (383.56, 526.18) | 65.42 (48.67, 81.28) | 1518.63 (1302.32, 1769.25) | 1308.56 (1121.07, 1530.26) | -13.83 (-21.00, -7.28) | -0.52 (-0.55, -0.49)* |
| Philippines | 917.40 (768.12, 1086.33) | 1525.27 (1286.49, 1785.04) | 66.26 (61.72, 71.18) | 1766.48 (1508.20, 2046.44) | 1443.30 (1232.06, 1672.23) | -18.29 (-19.23, -17.39) | -0.71 (-0.76, -0.66)* |
| Poland | 482.86 (414.44, 559.79) | 573.62 (495.52, 661.02) | 18.80 (13.82, 23.65) | 1191.85 (1018.06, 1385.25) | 1192.06 (1022.79, 1385.40) | 0.02 (-1.56, 1.63) | -0.02 (-0.06, 0.02) |
| Portugal | 444.87 (382.25, 522.43) | 466.29 (398.83, 541.30) | 4.82 (-6.00, 15.40) | 3923.34 (3372.33, 4605.77) | 3371.13 (2879.33, 3938.92) | -14.08 (-21.14, -7.05) | -0.49 (-0.57, -0.40)* |
| Puerto Rico | 76.29 (65.29, 87.98) | 87.13 (74.51, 102.05) | 14.22 (5.54, 24.61) | 2092.01 (1791.65, 2408.29) | 1992.90 (1692.16, 2326.18) | -4.74 (-10.83, 2.36) | -0.18 (-0.24, -0.11)* |
| Qatar | 14.67 (12.03, 17.85) | 101.40 (81.26, 124.41) | 591.19 (537.01, 653.98) | 3241.16 (2745.95, 3826.92) | 3002.21 (2538.19, 3579.96) | -7.37 (-13.38, -0.43) | -0.26 (-0.30, -0.23)* |
| Republic of Korea | 584.85 (506.45, 674.94) | 908.89 (807.73, 1015.32) | 55.41 (39.91, 72.15) | 1287.63 (1129.91, 1474.15) | 1420.90 (1258.66, 1594.52) | 10.35 (2.84, 18.28) | 0.34 (0.29, 0.39)* |
| Republic of Moldova | 119.02 (101.50, 139.23) | 101.86 (86.24, 120.95) | -14.42 (-22.93, -5.56) | 2634.35 (2261.43, 3068.80) | 2146.74 (1821.38, 2523.87) | -18.51 (-25.88, -11.15) | -0.69 (-0.75, -0.63)* |
| Romania | 416.67 (356.05, 485.38) | 391.73 (333.94, 455.41) | -5.98 (-14.70, 3.84) | 1627.45 (1390.59, 1893.82) | 1558.56 (1319.24, 1806.63) | -4.23 (-11.78, 4.30) | -0.15 (-0.16, -0.14)* |
| Russian Federation | 3634.06 (3134.22, 4210.78) | 3579.97 (3099.74, 4127.42) | -1.49 (-3.72, 0.75) | 2179.64 (1876.61, 2517.22) | 2014.13 (1728.78, 2333.77) | -7.59 (-8.89, -6.10) | -0.26 (-0.32, -0.21)* |
| Rwanda | 209.78 (173.26, 250.02) | 366.68 (306.85, 434.25) | 74.79 (63.19, 88.20) | 4482.32 (3791.09, 5280.95) | 3766.43 (3225.75, 4416.13) | -15.97 (-21.73, -9.90) | -0.61 (-0.64, -0.57)* |
| Saint Kitts and Nevis | 1.16 (0.94, 1.43) | 2.09 (1.67, 2.58) | 80.22 (65.71, 98.24) | 3109.48 (2510.48, 3831.90) | 3083.83 (2508.05, 3805.53) | -0.82 (-4.47, 3.63) | -0.03 (-0.05, -0.01)* |
| Saint Lucia | 2.80 (2.37, 3.32) | 4.90 (4.19, 5.74) | 75.30 (60.76, 92.87) | 2405.98 (2050.72, 2835.09) | 2444.88 (2086.01, 2848.92) | 1.62 (-3.99, 8.10) | 0.05 (0.02, 0.09)* |
| Saint Vincent and the Grenadines | 2.23 (1.89, 2.64) | 3.11 (2.66, 3.65) | 39.44 (26.84, 51.86) | 2386.63 (2044.58, 2812.33) | 2499.60 (2139.88, 2935.34) | 4.73 (-1.45, 11.29) | 0.16 (0.11, 0.20)* |
| Samoa | 2.30 (1.90, 2.81) | 2.83 (2.37, 3.42) | 22.94 (14.90, 32.35) | 1622.10 (1363.66, 1933.14) | 1446.78 (1223.68, 1723.80) | -10.81 (-15.88, -5.15) | -0.40 (-0.44, -0.36)* |
| San Marino | 0.79 (0.64, 0.97) | 1.19 (0.96, 1.45) | 49.45 (41.00, 58.72) | 3009.92 (2422.56, 3702.76) | 3048.57 (2460.10, 3781.64) | 1.28 (-3.10, 5.84) | 0.04 (0.04, 0.04)* |
| Sao Tome and Principe | 2.07 (1.73, 2.47) | 4.13 (3.42, 4.99) | 99.22 (86.94, 112.23) | 2405.76 (2032.24, 2863.53) | 2430.90 (2073.98, 2889.47) | 1.05 (-4.57, 6.68) | 0.03 (0.01, 0.06)* |
| Saudi Arabia | 389.98 (323.18, 473.51) | 1251.76 (1025.27, 1509.98) | 220.98 (185.85, 255.64) | 2949.01 (2498.96, 3506.54) | 3148.25 (2664.66, 3700.60) | 6.76 (-0.08, 14.20) | 0.23 (0.20, 0.25)* |
| Senegal | 135.96 (114.02, 160.04) | 309.84 (259.96, 366.15) | 127.89 (114.69, 143.74) | 2727.77 (2317.18, 3186.21) | 2758.39 (2345.87, 3227.06) | 1.12 (-4.96, 7.97) | 0.04 (-0.04, 0.10) |
| Serbia | 211.84 (180.09, 247.32) | 196.41 (167.04, 230.99) | -7.28 (-16.25, 1.18) | 2023.58 (1734.14, 2346.77) | 1731.57 (1474.20, 2013.99) | -14.43 (-20.97, -7.44) | -0.53 (-0.56, -0.51)* |
| Seychelles | 0.99 (0.84, 1.19) | 1.43 (1.21, 1.67) | 44.25 (32.07, 56.30) | 1445.45 (1229.31, 1696.77) | 1292.68 (1093.57, 1514.74) | -10.57 (-15.83, -4.82) | -0.40 (-0.46, -0.33)* |
| Sierra Leone | 77.68 (64.93, 92.39) | 191.94 (159.91, 227.81) | 147.10 (130.84, 165.53) | 2915.29 (2471.32, 3457.81) | 3145.11 (2671.27, 3705.18) | 7.88 (1.46, 15.76) | 0.26 (0.25, 0.27)* |
| Singapore | 74.55 (65.57, 84.60) | 92.78 (80.89, 105.94) | 24.45 (12.34, 37.15) | 2285.75 (2015.57, 2575.65) | 1452.17 (1265.45, 1683.42) | -36.47 (-42.32, -30.07) | -1.57 (-1.68, -1.46)* |
| Slovakia | 103.46 (88.96, 120.75) | 112.50 (95.72, 130.44) | 8.73 (0.31, 19.67) | 1843.21 (1585.75, 2153.01) | 1606.88 (1378.48, 1857.82) | -12.82 (-19.13, -5.19) | -0.47 (-0.49, -0.46)* |
| Slovenia | 60.96 (53.14, 70.18) | 57.68 (49.15, 67.23) | -5.38 (-14.85, 5.71) | 2731.01 (2385.88, 3147.04) | 2044.87 (1758.62, 2379.90) | -25.12 (-31.96, -18.06) | -0.98 (-1.04, -0.92)* |
| Solomon Islands | 4.67 (3.80, 5.70) | 9.34 (7.57, 11.35) | 99.94 (85.62, 113.50) | 1746.34 (1462.74, 2074.74) | 1650.82 (1383.18, 1975.40) | -5.47 (-10.96, -0.31) | -0.20 (-0.21, -0.18)* |
| Somalia | 162.09 (134.65, 196.27) | 455.78 (377.24, 547.44) | 181.18 (163.07, 200.90) | 3544.29 (3003.69, 4202.24) | 3515.91 (3016.43, 4162.02) | -0.80 (-7.34, 5.76) | -0.03 (-0.04, -0.02)* |
| South Africa | 935.54 (810.16, 1068.96) | 1684.27 (1474.47, 1911.02) | 80.03 (73.22, 86.73) | 3040.84 (2684.41, 3435.28) | 3066.90 (2715.09, 3448.98) | 0.86 (-1.55, 3.42) | 0.04 (0.00, 0.08)* |
| South Sudan | 132.78 (110.49, 158.13) | 218.53 (183.00, 259.66) | 64.58 (53.02, 75.78) | 3306.10 (2815.12, 3889.46) | 3402.09 (2887.14, 3986.40) | 2.90 (-4.23, 9.31) | 0.10 (0.08, 0.11)* |
| Spain | 1293.28 (1195.89, 1392.74) | 2053.92 (1851.20, 2239.84) | 58.81 (47.31, 69.21) | 2988.47 (2761.50, 3229.15) | 3667.60 (3239.25, 4075.48) | 22.73 (13.57, 32.39) | 0.72 (0.67, 0.77)* |
| Sri Lanka | 340.60 (288.34, 404.20) | 343.92 (296.05, 399.63) | 0.97 (-7.23, 10.34) | 2025.45 (1748.44, 2356.90) | 1489.97 (1275.36, 1737.64) | -26.44 (-31.56, -21.15) | -1.05 (-1.16, -0.94)* |
| Sudan | 554.75 (457.41, 668.06) | 1202.20 (997.89, 1454.90) | 116.71 (102.35, 132.24) | 3476.96 (2919.89, 4174.48) | 3393.12 (2853.32, 4007.70) | -2.41 (-8.78, 4.36) | -0.08 (-0.10, -0.07)* |
| Suriname | 12.90 (10.96, 15.14) | 23.32 (19.91, 27.04) | 80.82 (66.95, 96.84) | 3643.85 (3124.51, 4253.96) | 3872.72 (3299.73, 4487.36) | 6.28 (-0.20, 13.76) | 0.21 (0.20, 0.23)* |
| Sweden | 305.74 (270.37, 346.08) | 352.09 (314.09, 394.19) | 15.16 (8.36, 21.71) | 3113.42 (2743.92, 3542.87) | 3024.28 (2658.08, 3419.10) | -2.86 (-8.04, 2.55) | -0.10 (-0.13, -0.06)* |
| Switzerland | 261.28 (228.11, 302.94) | 269.33 (238.63, 303.47) | 3.08 (-5.33, 12.23) | 3306.44 (2892.33, 3845.31) | 2557.43 (2265.53, 2892.97) | -22.65 (-28.56, -16.22) | -0.89 (-1.01, -0.77)* |
| Syrian Arab Republic | 298.79 (244.95, 361.89) | 452.98 (378.39, 541.76) | 51.61 (39.43, 66.10) | 3073.38 (2594.06, 3646.40) | 3077.57 (2584.35, 3664.92) | 0.14 (-4.53, 5.74) | 0.00 (-0.04, 0.04) |
| Taiwan (Province of China) | 219.52 (185.87, 257.83) | 357.50 (305.71, 421.20) | 62.86 (46.40, 79.31) | 1094.18 (937.70, 1275.74) | 1180.16 (1010.47, 1366.17) | 7.86 (0.20, 14.98) | 0.26 (0.24, 0.28)* |
| Tajikistan | 75.91 (64.25, 90.04) | 137.36 (115.57, 163.92) | 80.95 (68.77, 95.92) | 1926.23 (1644.81, 2265.09) | 1732.60 (1488.70, 2033.16) | -10.05 (-16.40, -2.60) | -0.37 (-0.39, -0.35)* |
| Thailand | 827.99 (690.10, 987.19) | 1256.37 (1078.93, 1480.34) | 51.74 (33.19, 74.13) | 1478.36 (1248.87, 1726.30) | 1478.81 (1267.30, 1742.48) | 0.03 (-6.75, 8.98) | 0.01 (-0.01, 0.03) |
| Timor-Leste | 11.09 (9.12, 13.32) | 17.02 (14.08, 20.43) | 53.46 (42.88, 64.41) | 1724.72 (1460.79, 2019.70) | 1443.76 (1218.87, 1704.66) | -16.29 (-21.30, -11.22) | -0.61 (-0.63, -0.59)* |
| Togo | 72.22 (59.67, 86.73) | 192.95 (161.35, 229.59) | 167.18 (150.08, 186.33) | 3117.99 (2639.63, 3667.66) | 3245.88 (2757.71, 3822.54) | 4.10 (-2.78, 10.87) | 0.14 (0.13, 0.15)* |
| Tokelau | 0.03 (0.02, 0.03) | 0.02 (0.02, 0.03) | -6.34 (-11.36, -1.18) | 1801.17 (1439.18, 2270.59) | 1776.33 (1409.60, 2234.62) | -1.38 (-5.76, 3.33) | -0.06 (-0.09, -0.03)* |
| Tonga | 1.11 (0.92, 1.36) | 1.25 (1.05, 1.49) | 12.06 (4.93, 19.95) | 1342.63 (1131.94, 1600.56) | 1320.46 (1122.44, 1571.86) | -1.65 (-7.25, 4.12) | -0.06 (-0.08, -0.04)* |
| Trinidad and Tobago | 35.19 (29.83, 42.20) | 46.67 (39.76, 54.66) | 32.62 (18.78, 45.46) | 3167.72 (2695.79, 3743.90) | 2973.03 (2534.28, 3463.15) | -6.15 (-13.63, 0.74) | -0.22 (-0.24, -0.20)* |
| Tunisia | 292.34 (245.00, 348.74) | 506.28 (428.29, 602.00) | 73.18 (57.29, 90.87) | 4055.63 (3451.05, 4762.16) | 4044.92 (3427.81, 4791.00) | -0.26 (-6.00, 6.11) | -0.01 (-0.04, 0.01) |
| Turkey | 1617.49 (1445.20, 1813.92) | 2617.42 (2214.88, 3090.46) | 61.82 (45.37, 80.82) | 3002.56 (2699.75, 3348.40) | 2888.12 (2444.51, 3403.82) | -3.81 (-12.70, 6.88) | -0.17 (-0.31, -0.03)* |
| Turkmenistan | 61.26 (51.88, 72.55) | 93.75 (79.11, 111.12) | 53.03 (40.15, 66.96) | 2170.35 (1851.79, 2549.90) | 1974.61 (1675.30, 2304.60) | -9.02 (-16.29, -1.16) | -0.32 (-0.34, -0.30)* |
| Tuvalu | 0.15 (0.12, 0.19) | 0.21 (0.17, 0.27) | 36.49 (30.60, 42.79) | 1803.59 (1431.84, 2243.50) | 1779.43 (1414.85, 2238.54) | -1.34 (-5.61, 3.06) | -0.05 (-0.06, -0.04)* |
| Uganda | 604.88 (503.97, 724.58) | 1411.81 (1176.81, 1684.20) | 133.40 (116.72, 149.35) | 5550.73 (4762.96, 6539.85) | 5337.56 (4567.53, 6257.47) | -3.84 (-10.42, 3.03) | -0.14 (-0.18, -0.10)* |
| Ukraine | 1994.99 (1722.76, 2293.15) | 1653.30 (1422.23, 1905.58) | -17.13 (-20.84, -12.43) | 3251.01 (2800.99, 3745.13) | 2866.83 (2477.52, 3301.81) | -11.82 (-15.31, -7.66) | -0.44 (-0.51, -0.36)* |
| United Arab Emirates | 51.06 (41.48, 62.16) | 297.68 (235.29, 372.40) | 482.96 (409.50, 560.90) | 2841.48 (2419.68, 3357.10) | 2596.70 (2203.74, 3099.01) | -8.61 (-14.26, -2.88) | -0.32 (-0.35, -0.29)* |
| United Kingdom | 2272.43 (1984.38, 2591.44) | 2338.13 (2048.43, 2662.18) | 2.89 (0.48, 5.06) | 3524.28 (3043.02, 4051.75) | 3033.85 (2610.22, 3499.23) | -13.92 (-15.26, -12.68) | -0.51 (-0.62, -0.40)* |
| United Republic of Tanzania | 597.45 (498.28, 708.47) | 1319.30 (1093.90, 1570.53) | 120.82 (107.70, 135.26) | 3475.16 (2950.42, 4107.92) | 3295.73 (2782.61, 3873.07) | -5.16 (-11.02, 1.48) | -0.18 (-0.20, -0.17)* |
| United States Virgin Islands | 2.46 (2.07, 2.92) | 3.07 (2.64, 3.63) | 24.85 (13.75, 37.27) | 2385.50 (2026.77, 2817.00) | 2482.93 (2119.62, 2940.31) | 4.08 (-1.64, 11.18) | 0.14 (0.10, 0.17)* |
| United States of America | 6711.44 (5921.11, 7585.73) | 11303.86 (10062.64, 12641.46) | 68.43 (64.15, 73.47) | 2475.02 (2186.31, 2806.55) | 3294.06 (2911.31, 3737.79) | 33.09 (29.63, 36.38) | 0.96 (0.78, 1.15)* |
| Uruguay | 61.32 (52.40, 70.87) | 82.34 (70.80, 95.35) | 34.27 (24.32, 44.48) | 1886.23 (1607.01, 2188.96) | 2160.93 (1849.43, 2519.04) | 14.56 (6.25, 23.59) | 0.48 (0.45, 0.52)* |
| Uzbekistan | 359.52 (302.15, 421.01) | 635.81 (537.27, 751.21) | 76.85 (61.64, 92.67) | 2253.15 (1910.45, 2610.87) | 2087.99 (1793.01, 2450.32) | -7.33 (-15.07, 0.59) | -0.26 (-0.28, -0.25)* |
| Vanuatu | 2.15 (1.76, 2.62) | 4.28 (3.49, 5.19) | 98.59 (86.77, 110.64) | 1746.83 (1469.23, 2081.39) | 1622.51 (1359.38, 1922.81) | -7.12 (-11.96, -1.80) | -0.26 (-0.28, -0.24)* |
| Venezuela (Bolivarian Republic of) | 360.69 (301.87, 429.84) | 707.74 (602.13, 838.97) | 96.22 (79.76, 113.12) | 2359.77 (2013.57, 2779.21) | 2398.62 (2049.19, 2832.81) | 1.65 (-4.57, 7.64) | 0.06 (0.05, 0.06)* |
| Viet Nam | 726.51 (607.92, 872.31) | 1131.85 (954.31, 1336.21) | 55.79 (42.57, 72.66) | 1233.59 (1046.78, 1459.67) | 1093.13 (930.44, 1289.87) | -11.39 (-16.81, -4.71) | -0.41 (-0.43, -0.39)* |
| Yemen | 367.05 (299.83, 446.95) | 1020.77 (836.58, 1229.56) | 178.10 (161.46, 196.72) | 3849.39 (3215.31, 4632.37) | 3888.32 (3248.87, 4619.86) | 1.01 (-5.05, 7.29) | 0.03 (0.03, 0.04)* |
| Zambia | 145.72 (120.52, 175.07) | 361.39 (297.08, 436.02) | 148.00 (132.17, 166.41) | 2847.36 (2424.68, 3334.87) | 2850.01 (2414.36, 3367.79) | 0.09 (-6.49, 7.46) | 0.00 (-0.02, 0.03) |
| Zimbabwe | 133.47 (111.44, 160.18) | 228.08 (190.54, 272.17) | 70.89 (61.31, 81.06) | 2005.47 (1708.05, 2340.51) | 2085.99 (1789.05, 2446.04) | 4.02 (-1.75, 9.97) | 0.14 (0.11, 0.17)* |

| Table S10 The number and ASDR of MDD in 1990 and 2019, and the corresponding percentage change and AAPC of ASDR during 1990-2019 across 204 countries and territories. | | | | | | | |
| --- | --- | --- | --- | --- | --- | --- | --- |
|  |  |  | Percentage change of |  |  | Percentage change of | AAPC (95% CI) |
| Countries or territories | Number ×10^3^ (95% UI) | | number (%, 95% UI) | ASDR per 100 000 population (95% UI) | | ASDR (%, 95% UI) | of ASDR |
|  | 1990 year | 2019 year | during 1990-2019 | 1990 year | 2019 year | during 1990-2019 | during 1990-2019 |
| Afghanistan | 70.44 (46.97, 101.21) | 234.63 (155.84, 339.42) | 233.11 (207.13, 263.00) | 787.13 (527.43, 1111.71) | 783.06 (529.99, 1103.49) | -0.52 (-6.52, 6.66) | -0.02 (-0.04, 0.00) |
| Albania | 7.73 (5.09, 10.85) | 8.96 (6.06, 12.44) | 15.80 (3.26, 29.75) | 259.68 (174.88, 363.46) | 276.36 (187.66, 385.56) | 6.43 (-0.90, 14.92) | 0.22 (0.18, 0.25)* |
| Algeria | 134.89 (88.55, 193.82) | 265.34 (176.47, 375.59) | 96.71 (79.10, 115.98) | 653.41 (434.24, 927.72) | 627.14 (420.50, 883.91) | -4.02 (-9.98, 2.42) | -0.14 (-0.18, -0.11)* |
| American Samoa | 0.12 (0.08, 0.17) | 0.14 (0.10, 0.20) | 23.45 (12.93, 34.19) | 269.07 (181.19, 375.53) | 259.90 (173.44, 366.70) | -3.41 (-10.12, 3.61) | -0.12 (-0.16, -0.08)* |
| Andorra | 0.32 (0.22, 0.46) | 0.52 (0.35, 0.72) | 59.58 (45.99, 74.22) | 531.91 (358.82, 741.80) | 517.28 (346.48, 727.19) | -2.75 (-8.48, 3.50) | -0.09 (-0.14, -0.05)* |
| Angola | 73.10 (47.91, 105.58) | 201.12 (133.41, 287.75) | 175.15 (156.50, 195.93) | 958.93 (645.46, 1373.55) | 914.77 (618.90, 1291.55) | -4.61 (-10.81, 2.36) | -0.16 (-0.19, -0.14)* |
| Antigua and Barbuda | 0.25 (0.16, 0.36) | 0.43 (0.29, 0.60) | 73.44 (59.67, 87.13) | 434.25 (288.16, 612.79) | 433.73 (286.87, 608.12) | -0.12 (-5.96, 5.71) | -0.00 (-0.09, 0.08) |
| Argentina | 124.88 (84.97, 175.73) | 175.29 (120.21, 240.60) | 40.37 (27.54, 55.66) | 386.37 (262.28, 542.58) | 368.55 (251.80, 508.25) | -4.61 (-13.29, 5.70) | -0.17 (-0.21, -0.13)* |
| Armenia | 11.19 (7.38, 15.62) | 13.53 (9.04, 18.68) | 20.87 (10.54, 33.29) | 350.77 (233.50, 484.30) | 382.35 (254.64, 534.98) | 9.00 (1.56, 17.25) | 0.28 (0.19, 0.37)* |
| Australia | 125.30 (86.52, 171.67) | 180.62 (122.98, 255.37) | 44.15 (31.31, 57.56) | 698.28 (479.44, 956.97) | 698.28 (475.27, 998.29) | 0.00 (-9.06, 9.16) | -0.01 (-0.05, 0.03) |
| Austria | 45.65 (31.23, 63.63) | 43.46 (29.73, 60.78) | -4.80 (-13.24, 3.98) | 519.96 (353.95, 722.97) | 414.56 (283.37, 581.61) | -20.27 (-27.04, -13.39) | -0.79 (-0.84, -0.74)* |
| Azerbaijan | 21.74 (14.39, 31.03) | 36.35 (24.22, 50.93) | 67.21 (52.57, 82.44) | 337.18 (225.56, 471.42) | 334.88 (224.83, 463.27) | -0.68 (-6.80, 6.14) | -0.04 (-0.15, 0.07) |
| Bahamas | 1.07 (0.71, 1.52) | 1.83 (1.21, 2.57) | 71.23 (56.39, 86.97) | 449.34 (298.11, 627.50) | 440.91 (293.72, 615.93) | -1.87 (-8.02, 4.72) | -0.07 (-0.15, 0.01) |
| Bahrain | 4.06 (2.71, 5.76) | 12.23 (8.07, 17.37) | 201.29 (161.42, 242.30) | 827.37 (560.40, 1160.57) | 718.75 (483.71, 1007.71) | -13.13 (-19.09, -6.52) | -0.49 (-0.52, -0.46)* |
| Bangladesh | 593.27 (395.61, 842.50) | 1101.29 (739.49, 1556.17) | 85.63 (67.15, 105.55) | 733.60 (492.17, 1024.50) | 703.40 (474.26, 988.64) | -4.12 (-10.99, 3.78) | -0.15 (-0.16, -0.13)* |
| Barbados | 1.21 (0.82, 1.70) | 1.66 (1.12, 2.32) | 36.92 (24.98, 49.06) | 460.08 (309.79, 644.23) | 466.16 (313.52, 658.85) | 1.32 (-4.50, 7.46) | 0.04 (-0.01, 0.09) |
| Belarus | 67.04 (45.31, 93.99) | 66.76 (44.63, 92.45) | -0.41 (-9.49, 8.96) | 576.36 (389.55, 814.44) | 558.00 (371.49, 780.09) | -3.19 (-11.71, 4.84) | -0.09 (-0.18, -0.01)* |
| Belgium | 53.18 (37.31, 72.27) | 67.54 (45.63, 94.23) | 27.00 (13.98, 41.93) | 470.29 (328.45, 640.94) | 519.75 (351.41, 726.46) | 10.52 (-1.77, 23.50) | 0.34 (0.30, 0.39)* |
| Belize | 0.69 (0.46, 1.00) | 1.96 (1.32, 2.76) | 182.64 (160.75, 206.28) | 474.92 (317.23, 667.67) | 493.62 (330.90, 692.88) | 3.94 (-3.08, 10.72) | 0.13 (0.10, 0.16)* |
| Benin | 19.28 (12.72, 27.38) | 55.26 (36.61, 79.04) | 186.55 (168.99, 206.29) | 613.67 (415.86, 851.02) | 640.38 (434.40, 897.00) | 4.35 (-2.00, 11.68) | 0.15 (0.14, 0.16)* |
| Bermuda | 0.36 (0.24, 0.51) | 0.38 (0.26, 0.53) | 4.03 (-7.15, 15.88) | 554.42 (373.23, 776.07) | 467.59 (317.21, 658.17) | -15.66 (-22.56, -8.44) | -0.59 (-0.62, -0.56)* |
| Bhutan | 2.70 (1.81, 3.81) | 4.25 (2.82, 6.02) | 57.36 (44.83, 71.30) | 627.27 (421.17, 879.34) | 579.47 (389.74, 812.93) | -7.62 (-13.69, -0.48) | -0.28 (-0.31, -0.24)* |
| Bolivia (Plurinational State of) | 27.93 (18.74, 39.43) | 55.89 (37.83, 78.39) | 100.12 (86.05, 116.82) | 565.43 (383.50, 786.33) | 509.98 (347.57, 711.46) | -9.81 (-15.58, -3.06) | -0.35 (-0.38, -0.33)* |
| Bosnia and Herzegovina | 21.02 (14.07, 29.65) | 14.24 (9.50, 19.84) | -32.25 (-40.61, -23.20) | 445.10 (302.02, 623.91) | 335.74 (224.34, 470.45) | -24.57 (-32.22, -16.42) | -0.97 (-1.05, -0.89)* |
| Botswana | 5.44 (3.58, 7.73) | 12.74 (8.44, 18.12) | 134.18 (113.92, 154.97) | 576.60 (389.11, 802.38) | 591.52 (394.82, 828.42) | 2.59 (-4.53, 9.83) | 0.08 (0.07, 0.10)* |
| Brazil | 857.03 (586.44, 1186.99) | 1398.47 (971.93, 1901.06) | 63.18 (54.18, 71.91) | 638.99 (440.14, 885.17) | 599.97 (414.53, 816.81) | -6.11 (-10.41, -1.46) | -0.23 (-0.34, -0.12)* |
| Brunei Darussalam | 0.46 (0.30, 0.66) | 0.91 (0.61, 1.29) | 96.91 (79.05, 116.62) | 196.74 (132.03, 271.69) | 197.75 (132.85, 280.06) | 0.51 (-6.97, 8.28) | 0.01 (-0.03, 0.05) |
| Bulgaria | 39.13 (26.47, 54.50) | 30.51 (20.61, 42.09) | -22.04 (-30.05, -12.39) | 384.72 (262.36, 539.67) | 322.11 (218.64, 450.88) | -16.27 (-23.85, -7.68) | -0.61 (-0.64, -0.58)* |
| Burkina Faso | 39.84 (26.84, 56.45) | 96.37 (63.64, 137.50) | 141.89 (125.53, 160.24) | 634.38 (428.87, 886.60) | 624.64 (419.09, 879.96) | -1.54 (-8.01, 5.64) | -0.02 (-0.12, 0.08) |
| Burundi | 31.50 (20.71, 44.66) | 55.09 (36.60, 77.80) | 74.88 (62.26, 87.69) | 845.71 (570.12, 1185.08) | 670.70 (451.14, 943.35) | -20.69 (-26.29, -14.60) | -0.80 (-0.83, -0.78)* |
| Cabo Verde | 1.76 (1.18, 2.50) | 3.95 (2.65, 5.54) | 123.79 (104.73, 143.11) | 673.61 (452.35, 937.46) | 729.93 (493.41, 1021.85) | 8.36 (1.19, 16.15) | 0.29 (0.24, 0.34)* |
| Cambodia | 30.96 (20.17, 44.46) | 51.93 (34.60, 73.90) | 67.75 (52.54, 83.78) | 376.61 (252.99, 531.75) | 319.64 (215.80, 451.14) | -15.13 (-21.48, -7.93) | -0.57 (-0.59, -0.54)* |
| Cameroon | 45.78 (30.69, 64.76) | 143.35 (94.70, 203.34) | 213.14 (191.73, 236.82) | 642.87 (435.62, 898.91) | 672.03 (451.42, 930.49) | 4.54 (-2.36, 11.50) | 0.15 (0.13, 0.17)* |
| Canada | 129.64 (87.57, 177.19) | 162.78 (111.65, 223.69) | 25.57 (16.66, 35.10) | 439.47 (295.90, 604.42) | 438.74 (297.32, 614.30) | -0.16 (-8.09, 8.36) | -0.02 (-0.13, 0.09) |
| Central African Republic | 19.96 (13.05, 28.58) | 39.50 (26.31, 57.07) | 97.83 (86.09, 111.20) | 959.57 (639.86, 1347.70) | 947.68 (642.40, 1342.22) | -1.24 (-6.83, 4.78) | -0.05 (-0.07, -0.02)* |
| Chad | 30.67 (20.40, 43.50) | 79.71 (53.08, 114.00) | 159.93 (140.66, 179.18) | 760.30 (513.98, 1054.64) | 783.78 (529.02, 1098.63) | 3.09 (-4.22, 10.42) | 0.10 (0.07, 0.13)* |
| Chile | 97.54 (67.12, 132.75) | 121.37 (81.55, 170.44) | 24.43 (11.47, 39.73) | 756.46 (526.34, 1026.24) | 607.65 (410.05, 856.03) | -19.67 (-28.24, -10.26) | -0.73 (-0.86, -0.59)* |
| China | 4006.83 (2740.21, 5593.26) | 5088.00 (3514.40, 6995.49) | 26.98 (15.17, 39.43) | 335.72 (232.08, 464.14) | 285.39 (196.84, 392.86) | -14.99 (-18.94, -10.80) | -0.60 (-0.76, -0.44)* |
| Colombia | 91.91 (61.61, 131.08) | 146.69 (100.62, 201.57) | 59.61 (40.65, 79.34) | 325.55 (221.11, 458.14) | 285.78 (195.55, 393.54) | -12.22 (-20.57, -3.83) | -0.46 (-0.54, -0.38)* |
| Comoros | 1.98 (1.31, 2.81) | 3.67 (2.46, 5.17) | 85.46 (71.36, 100.81) | 611.57 (410.72, 860.13) | 582.46 (392.63, 820.36) | -4.76 (-11.69, 2.70) | -0.17 (-0.19, -0.15)* |
| Congo | 17.45 (11.42, 25.04) | 38.98 (26.01, 55.60) | 123.33 (106.44, 140.86) | 956.52 (644.64, 1341.73) | 868.58 (585.39, 1220.74) | -9.19 (-15.01, -3.28) | -0.33 (-0.36, -0.31)* |
| Cook Islands | 0.06 (0.04, 0.10) | 0.07 (0.05, 0.10) | 8.31 (-1.36, 20.12) | 363.26 (237.17, 534.07) | 364.87 (238.43, 534.92) | 0.44 (-5.10, 6.08) | 0.01 (-0.01, 0.04) |
| Costa Rica | 11.84 (7.95, 16.69) | 24.82 (16.53, 34.73) | 109.53 (91.92, 127.24) | 457.43 (309.94, 640.49) | 484.11 (323.13, 674.94) | 5.83 (-1.57, 13.13) | 0.20 (0.18, 0.21)* |
| Croatia | 27.21 (18.65, 38.01) | 22.02 (15.13, 30.47) | -19.06 (-27.09, -8.82) | 481.27 (329.43, 669.62) | 386.13 (263.86, 532.97) | -19.77 (-26.38, -11.19) | -0.76 (-0.80, -0.72)* |
| Cuba | 94.68 (62.72, 134.98) | 84.93 (57.49, 117.83) | -10.30 (-21.62, 1.45) | 826.17 (551.11, 1176.99) | 594.85 (405.14, 825.05) | -28.00 (-34.97, -21.17) | -1.14 (-1.16, -1.11)* |
| Cyprus | 3.32 (2.22, 4.64) | 6.13 (4.11, 8.54) | 84.56 (70.33, 99.70) | 412.63 (275.94, 576.46) | 412.43 (276.00, 583.73) | -0.05 (-6.13, 6.32) | -0.01 (-0.08, 0.06) |
| Czechia | 51.75 (35.16, 72.29) | 48.90 (33.55, 67.97) | -5.50 (-14.21, 3.86) | 442.92 (304.36, 619.28) | 359.99 (243.57, 503.75) | -18.72 (-25.55, -11.38) | -0.72 (-0.74, -0.69)* |
| Côte d’Ivoire | 42.72 (28.26, 60.95) | 103.49 (68.85, 147.27) | 142.25 (124.25, 160.15) | 526.22 (356.75, 737.38) | 533.86 (364.53, 740.72) | 1.45 (-4.62, 8.60) | 0.04 (0.03, 0.06)* |
| Democratic People's Republic of Korea | 60.26 (40.24, 84.75) | 79.55 (53.63, 111.58) | 32.01 (21.40, 43.97) | 304.02 (204.42, 424.59) | 263.28 (178.01, 369.47) | -13.40 (-19.71, -6.47) | -0.50 (-0.53, -0.48)* |
| Democratic Republic of the Congo | 242.94 (160.34, 349.74) | 573.74 (375.79, 825.08) | 136.17 (120.49, 153.85) | 869.92 (585.08, 1234.27) | 848.84 (569.93, 1190.66) | -2.42 (-8.33, 4.20) | -0.09 (-0.10, -0.07)* |
| Denmark | 40.46 (27.52, 56.19) | 33.47 (22.93, 46.02) | -17.29 (-24.81, -8.87) | 678.46 (460.44, 945.68) | 488.40 (332.07, 680.06) | -28.01 (-34.25, -20.77) | -1.15 (-1.22, -1.07)* |
| Djibouti | 2.01 (1.31, 2.86) | 6.32 (4.14, 9.00) | 214.83 (187.24, 241.93) | 624.50 (420.44, 873.47) | 629.02 (421.31, 889.39) | 0.72 (-5.88, 7.48) | 0.02 (0.01, 0.03)* |
| Dominica | 0.30 (0.20, 0.42) | 0.34 (0.23, 0.47) | 11.89 (3.98, 20.25) | 443.07 (296.43, 621.11) | 445.87 (300.96, 618.77) | 0.63 (-5.64, 6.75) | 0.02 (-0.02, 0.06) |
| Dominican Republic | 35.54 (23.32, 50.64) | 64.51 (42.59, 91.29) | 81.55 (66.05, 98.85) | 590.99 (394.83, 826.26) | 600.98 (398.48, 846.47) | 1.69 (-4.83, 9.24) | 0.05 (0.01, 0.10)* |
| Ecuador | 41.80 (27.68, 59.21) | 86.54 (57.83, 121.92) | 107.01 (87.82, 127.05) | 488.81 (326.15, 681.35) | 498.18 (335.12, 695.36) | 1.92 (-5.92, 10.52) | 0.06 (0.05, 0.08)* |
| Egypt | 262.95 (173.78, 372.18) | 547.68 (359.95, 767.33) | 108.28 (94.24, 124.02) | 555.56 (372.92, 772.28) | 587.67 (395.54, 815.46) | 5.78 (-0.80, 13.17) | 0.18 (0.02, 0.34)* |
| El Salvador | 26.12 (17.60, 37.70) | 33.04 (22.25, 46.80) | 26.48 (15.38, 37.13) | 586.42 (395.90, 834.46) | 522.38 (352.77, 742.93) | -10.92 (-17.59, -4.45) | -0.40 (-0.42, -0.38)* |
| Equatorial Guinea | 2.99 (1.98, 4.24) | 10.35 (6.73, 15.03) | 245.84 (221.22, 273.14) | 969.11 (646.52, 1361.22) | 911.04 (615.66, 1279.20) | -5.99 (-11.90, 0.25) | -0.22 (-0.24, -0.19)* |
| Eritrea | 14.30 (9.44, 20.31) | 35.13 (23.28, 49.80) | 145.75 (128.06, 165.33) | 732.97 (496.26, 1025.42) | 698.64 (469.72, 976.83) | -4.68 (-10.78, 2.48) | -0.16 (-0.17, -0.15)* |
| Estonia | 13.02 (8.98, 18.26) | 9.30 (6.28, 12.78) | -28.59 (-35.52, -21.12) | 734.73 (505.31, 1037.57) | 530.05 (355.39, 737.32) | -27.86 (-34.41, -20.64) | -1.13 (-1.20, -1.07)* |
| Eswatini | 3.10 (2.05, 4.43) | 5.92 (3.95, 8.39) | 90.89 (77.59, 106.43) | 573.74 (387.59, 806.31) | 622.49 (415.21, 878.78) | 8.50 (1.51, 16.45) | 0.28 (0.24, 0.32)* |
| Ethiopia | 253.29 (170.07, 355.60) | 504.61 (336.51, 712.13) | 99.22 (94.23, 104.41) | 747.79 (508.14, 1049.16) | 670.49 (455.95, 941.02) | -10.34 (-12.54, -8.17) | -0.38 (-0.40, -0.35)* |
| Fiji | 2.16 (1.43, 3.08) | 2.85 (1.89, 4.04) | 32.04 (21.86, 43.19) | 313.20 (211.52, 438.92) | 314.64 (210.06, 444.82) | 0.46 (-6.12, 7.61) | 0.01 (0.00, 0.03)* |
| Finland | 42.08 (28.69, 58.67) | 36.62 (25.37, 51.19) | -12.97 (-21.28, -4.49) | 769.39 (524.51, 1073.98) | 600.62 (412.35, 843.91) | -21.94 (-28.85, -14.87) | -0.86 (-0.99, -0.73)* |
| France | 442.94 (310.86, 596.08) | 428.85 (293.34, 589.02) | -3.18 (-14.64, 7.95) | 689.73 (482.89, 929.94) | 564.30 (380.21, 789.16) | -18.19 (-27.81, -7.87) | -0.67 (-0.74, -0.60)* |
| Gabon | 7.17 (4.81, 10.12) | 13.85 (9.23, 19.54) | 93.07 (79.96, 109.10) | 914.98 (619.02, 1281.71) | 870.37 (582.12, 1214.43) | -4.88 (-10.71, 2.07) | -0.17 (-0.19, -0.15)* |
| Gambia | 5.70 (3.77, 8.13) | 14.15 (9.35, 19.99) | 148.23 (131.96, 165.59) | 871.57 (585.87, 1218.00) | 862.58 (581.91, 1210.53) | -1.03 (-7.29, 5.90) | -0.04 (-0.06, -0.02)* |
| Georgia | 26.57 (17.92, 37.33) | 20.57 (13.83, 28.67) | -22.59 (-28.36, -16.21) | 448.39 (302.05, 629.19) | 451.36 (302.53, 631.18) | 0.66 (-6.16, 7.62) | 0.03 (-0.02, 0.08) |
| Germany | 407.97 (283.62, 552.58) | 488.40 (329.98, 676.17) | 19.71 (8.28, 31.63) | 439.55 (303.31, 599.76) | 482.84 (325.20, 677.92) | 9.85 (-0.23, 21.22) | 0.35 (0.21, 0.48)* |
| Ghana | 65.65 (43.24, 93.98) | 159.98 (105.79, 223.59) | 143.70 (129.15, 159.46) | 620.01 (417.57, 857.49) | 621.43 (421.62, 868.92) | 0.23 (-5.51, 6.02) | 0.01 (-0.00, 0.02) |
| Greece | 95.56 (63.95, 134.63) | 103.08 (70.09, 143.88) | 7.88 (-0.07, 17.95) | 808.19 (540.54, 1132.90) | 818.62 (544.97, 1153.50) | 1.29 (-5.00, 10.12) | 0.03 (-0.11, 0.16) |
| Greenland | 0.70 (0.47, 1.00) | 0.58 (0.39, 0.81) | -17.48 (-24.38, -10.51) | 1101.52 (748.37, 1556.88) | 983.91 (659.47, 1403.66) | -10.68 (-16.85, -4.31) | -0.38 (-0.44, -0.33)* |
| Grenada | 0.36 (0.24, 0.50) | 0.54 (0.36, 0.75) | 51.37 (40.47, 63.02) | 477.77 (324.10, 669.65) | 477.40 (318.47, 659.50) | -0.08 (-5.82, 5.88) | -0.00 (-0.05, 0.04) |
| Guam | 0.52 (0.34, 0.75) | 0.66 (0.44, 0.93) | 26.68 (15.71, 40.10) | 375.91 (251.75, 531.79) | 379.37 (254.91, 536.15) | 0.92 (-5.74, 8.64) | 0.03 (0.01, 0.05)* |
| Guatemala | 34.21 (22.79, 48.98) | 90.52 (59.99, 128.93) | 164.61 (147.11, 185.54) | 597.21 (406.46, 847.69) | 573.90 (390.02, 814.49) | -3.90 (-10.38, 3.32) | -0.12 (-0.22, -0.02)* |
| Guinea | 26.40 (17.78, 37.28) | 55.21 (36.54, 77.95) | 109.09 (95.51, 123.46) | 594.25 (403.59, 825.54) | 623.32 (417.18, 863.81) | 4.89 (-2.31, 12.40) | 0.16 (0.15, 0.17)* |
| Guinea-Bissau | 4.19 (2.76, 5.94) | 8.89 (5.88, 12.79) | 112.20 (98.96, 126.40) | 617.96 (414.66, 868.39) | 647.55 (437.23, 902.41) | 4.79 (-2.08, 12.30) | 0.16 (0.15, 0.17)* |
| Guyana | 5.11 (3.41, 7.21) | 6.48 (4.31, 9.10) | 26.97 (15.67, 38.37) | 756.46 (509.46, 1067.60) | 826.18 (557.15, 1159.91) | 9.22 (1.14, 17.59) | 0.31 (0.27, 0.36)* |
| Haiti | 29.50 (19.60, 42.17) | 62.10 (41.35, 87.88) | 110.53 (96.40, 124.30) | 574.85 (383.38, 816.03) | 554.63 (374.96, 775.20) | -3.52 (-9.30, 2.64) | -0.12 (-0.14, -0.11)* |
| Honduras | 14.60 (9.64, 20.52) | 39.66 (26.41, 55.69) | 171.64 (153.04, 192.70) | 444.49 (297.18, 616.84) | 467.87 (312.64, 651.86) | 5.26 (-1.71, 12.22) | 0.18 (0.17, 0.19)* |
| Hungary | 56.54 (38.42, 79.33) | 45.31 (30.70, 63.28) | -19.86 (-27.85, -9.87) | 470.29 (319.58, 657.18) | 348.24 (235.45, 488.04) | -25.95 (-32.58, -18.35) | -1.03 (-1.08, -0.98)* |
| Iceland | 1.14 (0.78, 1.60) | 1.43 (0.99, 2.00) | 25.36 (14.04, 37.11) | 433.61 (294.32, 606.42) | 376.96 (256.76, 529.18) | -13.06 (-19.95, -5.58) | -0.49 (-0.52, -0.45)* |
| India | 3998.21 (2719.74, 5554.86) | 6864.23 (4699.88, 9435.64) | 71.68 (64.65, 78.18) | 573.73 (391.83, 790.82) | 502.27 (344.19, 688.57) | -12.46 (-15.23, -10.04) | -0.47 (-0.57, -0.37)* |
| Indonesia | 366.75 (243.21, 519.85) | 591.81 (399.63, 830.47) | 61.36 (53.76, 69.73) | 219.11 (147.48, 307.62) | 217.76 (146.41, 305.26) | -0.62 (-2.57, 1.44) | -0.03 (-0.05, -0.00)* |
| Iran (Islamic Republic of) | 350.86 (229.77, 499.84) | 712.91 (474.95, 1026.87) | 103.19 (85.88, 120.42) | 740.52 (496.34, 1055.05) | 781.54 (527.08, 1117.97) | 5.54 (3.68, 7.38) | 0.21 (0.14, 0.27)* |
| Iraq | 79.27 (52.79, 111.80) | 234.00 (156.27, 332.57) | 195.20 (174.17, 217.90) | 578.26 (391.61, 812.05) | 582.44 (394.58, 818.16) | 0.72 (-5.90, 8.25) | 0.01 (-0.06, 0.07) |
| Ireland | 21.93 (14.88, 30.67) | 33.93 (23.46, 46.99) | 54.72 (42.28, 69.47) | 601.48 (406.90, 836.84) | 637.40 (437.07, 892.83) | 5.97 (-2.90, 15.53) | 0.21 (0.11, 0.32)* |
| Israel | 32.56 (21.81, 45.26) | 59.07 (40.33, 81.55) | 81.38 (68.01, 97.61) | 676.50 (452.62, 940.70) | 624.00 (423.10, 868.65) | -7.76 (-14.45, -0.03) | -0.30 (-0.35, -0.25)* |
| Italy | 366.54 (249.09, 510.56) | 384.08 (265.57, 532.09) | 4.78 (0.62, 8.94) | 548.85 (371.33, 763.42) | 517.12 (348.83, 721.22) | -5.78 (-7.33, -4.25) | -0.23 (-0.31, -0.14)* |
| Jamaica | 9.23 (6.08, 12.96) | 13.42 (8.89, 18.76) | 45.49 (34.31, 57.93) | 436.64 (287.16, 613.73) | 441.96 (293.43, 619.86) | 1.22 (-4.75, 7.54) | 0.04 (-0.01, 0.08) |
| Japan | 415.94 (287.07, 569.41) | 472.88 (328.54, 641.77) | 13.69 (9.02, 18.81) | 294.19 (202.74, 403.24) | 317.24 (218.02, 436.11) | 7.83 (5.70, 10.22) | 0.27 (0.21, 0.33)* |
| Jordan | 21.15 (13.88, 30.52) | 70.98 (46.62, 101.54) | 235.67 (208.10, 265.19) | 717.73 (486.04, 1000.71) | 635.45 (424.28, 883.08) | -11.46 (-18.05, -4.33) | -0.43 (-0.47, -0.39)* |
| Kazakhstan | 73.41 (49.71, 102.66) | 88.02 (58.74, 123.32) | 19.90 (10.36, 30.38) | 486.64 (329.84, 679.17) | 476.58 (323.17, 665.55) | -2.07 (-9.67, 6.13) | -0.06 (-0.09, -0.03)* |
| Kenya | 107.12 (72.85, 149.07) | 261.04 (177.16, 362.29) | 143.70 (138.46, 148.93) | 737.31 (507.08, 1015.23) | 681.12 (472.88, 939.82) | -7.62 (-8.90, -6.31) | -0.27 (-0.31, -0.24)* |
| Kiribati | 0.23 (0.15, 0.34) | 0.35 (0.23, 0.51) | 51.39 (39.99, 64.67) | 356.12 (237.85, 503.76) | 321.75 (217.03, 451.64) | -9.65 (-16.10, -2.23) | -0.35 (-0.36, -0.34)* |
| Kuwait | 10.08 (6.58, 14.37) | 31.10 (20.56, 44.06) | 208.59 (181.13, 242.74) | 592.74 (395.93, 836.35) | 616.13 (410.69, 865.20) | 3.95 (-2.21, 11.17) | 0.13 (0.10, 0.16)* |
| Kyrgyzstan | 19.35 (13.08, 27.24) | 27.75 (18.48, 39.75) | 43.44 (30.87, 56.42) | 520.78 (350.92, 727.77) | 467.33 (315.01, 659.30) | -10.26 (-17.51, -1.97) | -0.38 (-0.40, -0.35)* |
| Lao People's Democratic Republic | 10.98 (7.17, 15.66) | 19.88 (13.18, 28.18) | 80.94 (66.46, 99.87) | 320.50 (215.36, 451.98) | 281.99 (189.69, 398.58) | -12.02 (-18.65, -4.29) | -0.45 (-0.50, -0.40)* |
| Latvia | 20.42 (13.93, 28.59) | 13.79 (9.49, 19.07) | -32.45 (-39.20, -24.37) | 665.34 (455.34, 927.39) | 540.90 (366.65, 745.76) | -18.70 (-25.66, -9.96) | -0.71 (-0.76, -0.67)* |
| Lebanon | 19.70 (13.12, 27.81) | 38.79 (26.20, 54.65) | 96.84 (78.52, 115.19) | 687.10 (459.45, 963.62) | 732.01 (492.18, 1025.94) | 6.54 (-3.12, 15.76) | 0.22 (0.17, 0.28)* |
| Lesotho | 11.77 (7.94, 16.81) | 15.90 (10.75, 22.29) | 35.01 (22.05, 46.78) | 846.92 (571.73, 1191.43) | 849.45 (577.23, 1176.72) | 0.30 (-8.05, 8.53) | -0.00 (-0.06, 0.05) |
| Liberia | 9.38 (6.36, 13.21) | 24.33 (16.02, 34.43) | 159.21 (139.08, 182.06) | 656.03 (449.70, 915.39) | 656.75 (446.43, 918.15) | 0.11 (-7.18, 8.55) | 0.04 (-0.01, 0.08) |
| Libya | 22.13 (14.48, 31.63) | 50.65 (33.75, 71.11) | 128.84 (108.31, 153.57) | 651.90 (438.42, 913.00) | 683.12 (456.45, 951.87) | 4.79 (-0.64, 11.53) | 0.16 (0.14, 0.17)* |
| Lithuania | 26.20 (17.67, 36.74) | 21.70 (14.78, 30.69) | -17.16 (-25.07, -8.75) | 644.95 (434.14, 901.02) | 592.37 (402.09, 834.35) | -8.15 (-15.49, 0.24) | -0.29 (-0.32, -0.26)* |
| Luxembourg | 2.44 (1.64, 3.41) | 3.10 (2.15, 4.23) | 26.80 (15.05, 38.54) | 563.03 (381.85, 785.63) | 433.52 (295.79, 592.66) | -23.00 (-29.76, -16.11) | -0.91 (-0.95, -0.86)* |
| Madagascar | 57.69 (38.25, 81.88) | 134.40 (88.43, 190.32) | 132.99 (116.50, 149.48) | 704.77 (476.32, 983.74) | 680.97 (456.33, 951.31) | -3.38 (-9.68, 3.62) | -0.12 (-0.13, -0.10)* |
| Malawi | 37.45 (24.82, 53.10) | 71.27 (46.57, 101.76) | 90.31 (76.50, 105.95) | 575.95 (388.08, 807.63) | 542.27 (364.67, 757.23) | -5.85 (-11.98, 1.44) | -0.21 (-0.22, -0.19)* |
| Malaysia | 59.45 (39.66, 83.82) | 155.23 (104.63, 219.71) | 161.08 (134.39, 192.75) | 391.83 (263.71, 552.04) | 477.06 (322.56, 673.01) | 21.75 (9.47, 35.51) | 0.67 (0.57, 0.77)* |
| Maldives | 0.78 (0.52, 1.13) | 1.70 (1.13, 2.40) | 118.05 (94.80, 142.73) | 453.79 (310.48, 638.45) | 328.63 (220.51, 462.47) | -27.58 (-32.70, -21.71) | -1.11 (-1.15, -1.08)* |
| Mali | 27.65 (18.43, 38.73) | 66.61 (43.60, 94.24) | 140.88 (122.10, 159.86) | 461.20 (311.20, 644.43) | 451.31 (301.64, 633.91) | -2.15 (-9.71, 4.83) | -0.08 (-0.13, -0.04)* |
| Malta | 1.68 (1.12, 2.32) | 2.13 (1.44, 2.95) | 26.58 (17.18, 37.44) | 428.06 (285.95, 597.93) | 416.79 (281.46, 582.46) | -2.63 (-8.65, 3.71) | -0.10 (-0.14, -0.06)* |
| Marshall Islands | 0.12 (0.07, 0.17) | 0.17 (0.11, 0.24) | 46.07 (33.78, 60.64) | 328.60 (219.81, 465.31) | 309.56 (205.28, 435.77) | -5.79 (-11.39, 0.85) | -0.21 (-0.23, -0.18)* |
| Mauritania | 7.29 (4.83, 10.31) | 14.71 (9.57, 20.93) | 101.77 (86.79, 117.82) | 498.28 (336.43, 696.16) | 476.37 (313.64, 661.42) | -4.40 (-11.30, 3.08) | -0.16 (-0.20, -0.12)* |
| Mauritius | 6.43 (4.28, 9.17) | 7.31 (4.98, 10.30) | 13.77 (2.38, 26.50) | 586.05 (395.03, 836.95) | 501.93 (338.10, 715.97) | -14.35 (-20.70, -7.89) | -0.54 (-0.57, -0.51)* |
| Mexico | 301.34 (204.70, 416.99) | 702.13 (480.47, 970.65) | 133.00 (122.46, 143.91) | 439.77 (303.11, 603.24) | 543.54 (373.60, 749.99) | 23.60 (20.60, 26.87) | 0.73 (0.70, 0.76)* |
| Micronesia (Federated States of) | 0.29 (0.19, 0.41) | 0.32 (0.21, 0.45) | 10.43 (0.75, 21.25) | 339.03 (226.18, 479.16) | 313.91 (209.43, 443.55) | -7.41 (-14.13, -0.53) | -0.27 (-0.29, -0.25)* |
| Monaco | 0.23 (0.15, 0.33) | 0.27 (0.19, 0.40) | 20.82 (14.07, 28.16) | 614.01 (405.76, 883.16) | 610.19 (397.72, 875.65) | -0.62 (-5.39, 4.44) | -0.02 (-0.04, -0.01)* |
| Mongolia | 9.98 (6.59, 14.30) | 19.08 (12.55, 27.01) | 91.20 (73.57, 110.31) | 592.41 (393.49, 842.00) | 590.15 (393.96, 831.82) | -0.38 (-6.81, 6.35) | -0.01 (-0.04, 0.01) |
| Montenegro | 2.32 (1.55, 3.27) | 2.63 (1.77, 3.65) | 13.73 (3.33, 25.07) | 359.02 (241.10, 504.34) | 348.73 (233.38, 486.60) | -2.87 (-10.34, 4.85) | -0.10 (-0.11, -0.08)* |
| Morocco | 183.02 (122.28, 262.77) | 307.87 (204.40, 432.06) | 68.22 (53.99, 84.20) | 848.31 (571.82, 1199.11) | 825.20 (553.01, 1154.00) | -2.72 (-9.31, 4.51) | -0.10 (-0.11, -0.08)* |
| Mozambique | 62.68 (42.17, 88.78) | 142.23 (93.37, 201.89) | 126.92 (109.51, 143.52) | 688.21 (465.17, 960.58) | 722.80 (488.53, 1007.10) | 5.03 (-2.92, 13.18) | 0.17 (0.14, 0.19)* |
| Myanmar | 59.38 (38.82, 84.70) | 89.58 (60.04, 125.69) | 50.86 (38.49, 64.65) | 163.59 (108.14, 229.60) | 161.10 (108.18, 224.84) | -1.52 (-8.22, 6.15) | -0.08 (-0.25, 0.08) |
| Namibia | 5.04 (3.38, 7.19) | 9.53 (6.39, 13.32) | 89.14 (73.69, 104.55) | 471.36 (315.66, 663.37) | 461.37 (309.62, 640.80) | -2.12 (-8.71, 5.00) | -0.09 (-0.16, -0.03)* |
| Nauru | 0.03 (0.02, 0.05) | 0.03 (0.02, 0.05) | 13.91 (6.65, 21.99) | 360.55 (232.58, 529.05) | 362.34 (237.38, 532.81) | 0.50 (-5.45, 6.87) | 0.01 (-0.01, 0.03) |
| Nepal | 99.69 (66.51, 139.29) | 209.22 (141.60, 289.90) | 109.88 (93.86, 127.73) | 702.28 (474.76, 973.43) | 754.62 (510.96, 1045.80) | 7.45 (-0.32, 16.09) | 0.25 (0.18, 0.32)* |
| Netherlands | 90.51 (63.48, 122.22) | 101.76 (69.33, 142.25) | 12.43 (0.39, 25.63) | 534.18 (373.87, 724.98) | 512.62 (349.61, 711.50) | -4.04 (-14.64, 7.84) | -0.12 (-0.25, 0.01) |
| New Zealand | 18.64 (12.41, 26.35) | 24.34 (16.66, 33.57) | 30.53 (20.52, 41.85) | 516.36 (344.21, 730.38) | 553.78 (373.60, 771.56) | 7.25 (-0.50, 15.90) | 0.24 (0.22, 0.25)* |
| Nicaragua | 13.94 (9.28, 19.89) | 30.55 (20.49, 42.66) | 119.08 (100.78, 140.33) | 483.57 (327.33, 673.41) | 492.36 (332.39, 682.00) | 1.82 (-3.88, 9.72) | 0.07 (0.04, 0.09)* |
| Niger | 29.46 (19.53, 42.48) | 84.44 (55.42, 119.75) | 186.65 (167.34, 208.84) | 579.35 (392.55, 814.74) | 597.66 (399.74, 833.13) | 3.16 (-4.13, 9.98) | 0.11 (0.10, 0.12)* |
| Nigeria | 392.97 (263.48, 551.17) | 760.54 (512.93, 1069.44) | 93.54 (90.11, 97.36) | 602.77 (410.52, 843.37) | 509.66 (348.25, 710.60) | -15.45 (-16.82, -13.94) | -0.57 (-0.64, -0.51)* |
| Niue | 0.01 (0.01, 0.01) | 0.01 (0.00, 0.01) | -17.72 (-23.74, -11.29) | 364.11 (238.96, 528.77) | 361.32 (235.01, 531.03) | -0.77 (-6.19, 5.67) | -0.03 (-0.04, -0.01)* |
| North Macedonia | 6.64 (4.43, 9.30) | 7.82 (5.27, 10.94) | 17.75 (7.10, 30.31) | 326.23 (220.15, 456.06) | 297.41 (201.11, 414.44) | -8.83 (-15.94, -0.86) | -0.32 (-0.37, -0.27)* |
| Northern Mariana Islands | 0.13 (0.08, 0.19) | 0.14 (0.09, 0.20) | 8.07 (-5.05, 23.11) | 273.76 (181.78, 383.23) | 291.09 (195.34, 405.70) | 6.33 (-0.91, 14.44) | 0.21 (0.15, 0.27)* |
| Norway | 17.99 (12.20, 25.05) | 26.14 (17.72, 36.44) | 45.33 (41.38, 49.13) | 387.40 (260.32, 539.36) | 446.41 (302.04, 626.20) | 15.23 (12.78, 17.44) | 0.48 (0.31, 0.64)* |
| Oman | 9.52 (6.30, 13.42) | 29.21 (19.50, 42.35) | 206.82 (180.04, 237.05) | 598.60 (401.96, 834.05) | 604.13 (404.91, 852.85) | 0.92 (-5.87, 7.63) | 0.03 (-0.03, 0.09) |
| Pakistan | 465.72 (315.29, 652.57) | 984.34 (666.12, 1396.45) | 111.36 (103.74, 118.82) | 587.82 (400.92, 824.47) | 562.60 (386.40, 791.82) | -4.29 (-7.29, -1.30) | -0.15 (-0.20, -0.11)* |
| Palau | 0.05 (0.03, 0.08) | 0.07 (0.05, 0.10) | 30.76 (16.38, 47.52) | 361.45 (237.83, 528.40) | 357.05 (230.00, 516.13) | -1.22 (-6.51, 4.88) | -0.05 (-0.07, -0.02)* |
| Palestine | 16.32 (10.80, 23.48) | 46.33 (30.86, 66.19) | 183.87 (166.48, 202.97) | 1079.95 (728.50, 1531.15) | 1060.22 (725.32, 1494.32) | -1.83 (-7.47, 3.34) | -0.06 (-0.13, 0.01) |
| Panama | 9.05 (6.02, 12.65) | 17.98 (12.04, 25.15) | 98.64 (84.19, 115.07) | 434.01 (290.76, 603.59) | 426.73 (285.60, 595.56) | -1.68 (-7.67, 4.53) | -0.06 (-0.07, -0.05)* |
| Papua New Guinea | 13.29 (8.61, 19.12) | 31.43 (20.60, 44.65) | 136.55 (120.88, 152.30) | 371.80 (243.89, 525.11) | 348.95 (230.76, 496.79) | -6.14 (-11.69, -0.49) | -0.22 (-0.23, -0.21)* |
| Paraguay | 18.21 (12.00, 25.87) | 39.84 (26.70, 56.25) | 118.79 (101.24, 135.74) | 529.28 (352.78, 739.69) | 573.00 (383.42, 806.95) | 8.26 (0.61, 15.36) | 0.27 (0.24, 0.30)* |
| Peru | 55.52 (36.81, 78.88) | 91.06 (60.11, 127.35) | 64.01 (47.17, 82.25) | 306.91 (203.89, 434.45) | 264.73 (175.43, 369.39) | -13.74 (-21.94, -5.77) | -0.52 (-0.56, -0.48)* |
| Philippines | 187.70 (124.48, 265.95) | 311.63 (208.61, 438.99) | 66.02 (61.31, 71.22) | 356.02 (239.94, 499.52) | 292.19 (195.97, 410.48) | -17.93 (-19.11, -16.76) | -0.69 (-0.75, -0.64)* |
| Poland | 96.74 (66.12, 135.79) | 114.39 (78.24, 159.73) | 18.24 (13.16, 23.65) | 239.47 (162.85, 334.94) | 241.26 (162.30, 339.77) | 0.75 (-1.36, 2.89) | 0.01 (-0.03, 0.04) |
| Portugal | 88.85 (59.82, 123.71) | 91.92 (62.22, 127.24) | 3.45 (-7.16, 14.51) | 788.08 (530.66, 1098.27) | 678.15 (454.32, 938.52) | -13.95 (-21.40, -6.56) | -0.49 (-0.58, -0.40)* |
| Puerto Rico | 15.48 (10.40, 21.36) | 17.33 (11.69, 23.87) | 11.96 (3.25, 23.52) | 424.06 (284.53, 583.80) | 403.27 (271.53, 563.85) | -4.90 (-11.50, 2.93) | -0.18 (-0.25, -0.12)* |
| Qatar | 3.03 (1.98, 4.33) | 20.90 (13.71, 30.02) | 589.58 (527.78, 655.74) | 658.80 (440.65, 914.04) | 609.02 (409.73, 862.25) | -7.56 (-13.98, -0.41) | -0.27 (-0.31, -0.23)* |
| Republic of Korea | 119.94 (80.45, 168.32) | 183.10 (127.20, 250.32) | 52.66 (36.60, 70.80) | 261.33 (177.67, 362.34) | 289.88 (198.35, 400.17) | 10.93 (2.26, 19.91) | 0.35 (0.28, 0.42)* |
| Republic of Moldova | 23.80 (15.99, 33.12) | 20.24 (13.63, 28.74) | -14.93 (-23.57, -5.76) | 526.12 (353.58, 731.06) | 430.86 (288.93, 608.96) | -18.11 (-25.75, -10.59) | -0.68 (-0.74, -0.61)* |
| Romania | 83.46 (55.86, 115.34) | 77.68 (52.55, 107.39) | -6.93 (-15.73, 3.02) | 326.96 (221.08, 456.74) | 314.57 (212.89, 439.68) | -3.79 (-12.01, 5.03) | -0.13 (-0.14, -0.11)* |
| Russian Federation | 722.40 (489.53, 1017.63) | 710.81 (485.25, 994.09) | -1.60 (-4.04, 0.80) | 434.72 (294.22, 609.76) | 405.10 (274.08, 567.51) | -6.82 (-8.35, -5.27) | -0.24 (-0.30, -0.18)* |
| Rwanda | 42.44 (28.33, 60.18) | 74.43 (48.56, 104.73) | 75.36 (63.32, 89.91) | 892.20 (604.50, 1240.85) | 751.92 (505.33, 1052.15) | -15.72 (-21.81, -9.53) | -0.60 (-0.65, -0.55)* |
| Saint Kitts and Nevis | 0.23 (0.15, 0.34) | 0.42 (0.27, 0.61) | 79.28 (64.63, 97.03) | 626.85 (411.88, 902.93) | 621.37 (406.57, 904.88) | -0.88 (-5.14, 3.93) | -0.03 (-0.05, -0.01)* |
| Saint Lucia | 0.57 (0.38, 0.81) | 0.98 (0.67, 1.37) | 72.68 (57.16, 90.73) | 485.10 (325.18, 675.02) | 492.60 (333.34, 687.18) | 1.55 (-4.82, 8.08) | 0.05 (0.01, 0.09)* |
| Saint Vincent and the Grenadines | 0.46 (0.30, 0.64) | 0.63 (0.42, 0.87) | 37.33 (24.79, 50.35) | 482.99 (323.23, 675.48) | 504.82 (337.94, 708.48) | 4.52 (-2.24, 11.23) | 0.15 (0.11, 0.19)* |
| Samoa | 0.48 (0.32, 0.68) | 0.58 (0.38, 0.83) | 21.94 (12.49, 32.62) | 330.79 (222.67, 463.76) | 293.90 (195.88, 412.68) | -11.15 (-17.17, -4.44) | -0.41 (-0.45, -0.38)* |
| San Marino | 0.16 (0.10, 0.23) | 0.24 (0.16, 0.34) | 47.90 (38.74, 57.56) | 610.59 (398.43, 885.04) | 617.30 (405.83, 889.55) | 1.10 (-3.79, 5.94) | 0.04 (0.03, 0.04)* |
| Sao Tome and Principe | 0.42 (0.27, 0.60) | 0.84 (0.55, 1.20) | 99.61 (85.60, 115.36) | 484.48 (320.10, 682.74) | 489.05 (320.11, 683.11) | 0.94 (-4.86, 7.37) | 0.03 (-0.00, 0.06) |
| Saudi Arabia | 80.48 (52.58, 116.40) | 256.18 (167.32, 362.90) | 218.32 (182.41, 255.12) | 598.16 (403.05, 843.60) | 636.82 (430.77, 886.31) | 6.46 (-1.13, 14.18) | 0.22 (0.19, 0.25)* |
| Senegal | 27.59 (18.33, 39.21) | 62.93 (42.26, 88.88) | 128.09 (113.08, 144.34) | 544.43 (364.35, 760.18) | 551.43 (378.07, 772.17) | 1.28 (-5.28, 8.85) | 0.04 (-0.02, 0.10) |
| Serbia | 42.48 (28.86, 59.34) | 38.73 (26.30, 53.68) | -8.84 (-17.82, 0.47) | 407.00 (276.48, 570.72) | 347.10 (234.62, 477.68) | -14.72 (-22.00, -6.96) | -0.54 (-0.57, -0.52)* |
| Seychelles | 0.20 (0.14, 0.29) | 0.29 (0.19, 0.41) | 42.58 (29.47, 56.79) | 295.37 (199.11, 415.16) | 263.20 (177.43, 370.06) | -10.89 (-17.10, -3.76) | -0.40 (-0.43, -0.37)* |
| Sierra Leone | 15.67 (10.25, 22.20) | 39.07 (25.81, 55.19) | 149.40 (131.25, 169.16) | 581.00 (389.74, 817.47) | 628.88 (424.51, 883.03) | 8.24 (0.87, 16.62) | 0.28 (0.26, 0.29)* |
| Singapore | 15.33 (10.36, 21.27) | 18.94 (12.85, 26.08) | 23.51 (11.05, 36.86) | 465.82 (317.49, 643.94) | 298.62 (201.50, 420.47) | -35.89 (-42.31, -29.09) | -1.54 (-1.65, -1.43)* |
| Slovakia | 20.71 (14.00, 29.39) | 22.30 (15.26, 31.03) | 7.68 (-1.70, 19.04) | 369.72 (250.22, 524.46) | 322.70 (220.04, 449.79) | -12.72 (-19.86, -4.80) | -0.47 (-0.50, -0.44)* |
| Slovenia | 12.17 (8.26, 16.76) | 11.33 (7.80, 15.61) | -6.91 (-16.92, 4.51) | 547.38 (374.05, 757.47) | 410.46 (280.88, 565.63) | -25.01 (-32.23, -17.39) | -0.98 (-1.06, -0.90)* |
| Solomon Islands | 0.96 (0.63, 1.40) | 1.92 (1.26, 2.74) | 98.90 (82.87, 116.27) | 355.31 (237.45, 500.71) | 334.49 (221.83, 471.53) | -5.86 (-11.79, 0.53) | -0.21 (-0.23, -0.19)* |
| Somalia | 32.82 (21.99, 46.87) | 92.76 (61.43, 131.80) | 182.66 (162.96, 202.99) | 705.26 (477.91, 995.53) | 700.89 (476.63, 980.03) | -0.62 (-7.72, 6.25) | -0.02 (-0.03, -0.01)* |
| South Africa | 188.78 (130.13, 260.92) | 335.20 (231.29, 460.73) | 77.56 (70.65, 84.49) | 605.49 (417.62, 832.77) | 606.18 (420.19, 830.48) | 0.11 (-2.55, 2.97) | 0.02 (-0.02, 0.06) |
| South Sudan | 26.71 (17.69, 37.81) | 43.97 (28.99, 61.98) | 64.65 (52.41, 76.59) | 651.25 (437.18, 909.72) | 671.25 (448.39, 933.83) | 3.07 (-4.29, 9.98) | 0.10 (0.09, 0.11)* |
| Spain | 260.45 (182.82, 352.24) | 408.44 (285.89, 556.27) | 56.82 (44.60, 68.03) | 605.75 (424.16, 821.40) | 742.49 (516.73, 1014.02) | 22.57 (12.01, 32.50) | 0.71 (0.67, 0.76)* |
| Sri Lanka | 70.16 (47.30, 100.93) | 70.06 (46.89, 98.54) | -0.14 (-8.96, 10.28) | 413.76 (282.32, 587.45) | 304.49 (204.11, 427.52) | -26.41 (-32.31, -20.10) | -1.07 (-1.15, -0.99)* |
| Sudan | 113.90 (74.72, 161.62) | 247.10 (162.63, 352.42) | 116.94 (101.18, 132.78) | 705.96 (473.71, 975.41) | 688.63 (463.01, 966.65) | -2.46 (-9.45, 4.42) | -0.08 (-0.10, -0.07)* |
| Suriname | 2.63 (1.76, 3.76) | 4.70 (3.17, 6.61) | 78.44 (64.02, 94.79) | 737.39 (496.37, 1048.44) | 780.29 (527.54, 1100.41) | 5.82 (-1.02, 13.90) | 0.20 (0.18, 0.22)* |
| Sweden | 61.22 (41.91, 84.35) | 70.30 (48.05, 96.07) | 14.83 (7.75, 21.59) | 630.33 (430.94, 871.54) | 613.29 (418.83, 848.44) | -2.70 (-8.09, 2.92) | -0.09 (-0.12, -0.06)* |
| Switzerland | 52.23 (35.60, 73.11) | 53.69 (37.33, 73.21) | 2.79 (-5.22, 12.12) | 666.11 (458.66, 935.61) | 517.33 (358.07, 717.67) | -22.33 (-28.27, -15.73) | -0.88 (-1.00, -0.76)* |
| Syrian Arab Republic | 61.57 (40.19, 87.96) | 91.85 (61.47, 129.82) | 49.19 (36.59, 64.10) | 624.44 (418.16, 877.65) | 621.20 (415.87, 880.50) | -0.52 (-5.91, 5.42) | -0.02 (-0.06, 0.02) |
| Taiwan (Province of China) | 44.97 (30.00, 62.28) | 72.08 (48.31, 101.07) | 60.31 (43.69, 78.48) | 222.24 (149.62, 309.17) | 240.62 (162.19, 334.38) | 8.27 (-0.21, 16.85) | 0.27 (0.25, 0.30)* |
| Tajikistan | 15.52 (10.20, 22.07) | 28.11 (18.61, 39.66) | 81.08 (66.98, 96.49) | 388.72 (260.81, 547.95) | 349.08 (234.10, 482.65) | -10.20 (-16.90, -2.98) | -0.38 (-0.40, -0.35)* |
| Thailand | 170.43 (113.67, 244.04) | 254.00 (170.88, 354.19) | 49.03 (29.76, 71.74) | 300.79 (205.58, 425.07) | 301.38 (200.83, 424.03) | 0.20 (-8.01, 9.45) | 0.01 (-0.01, 0.03) |
| Timor-Leste | 2.28 (1.49, 3.26) | 3.50 (2.32, 5.07) | 53.39 (41.17, 66.68) | 349.28 (233.40, 490.24) | 292.95 (196.72, 414.41) | -16.13 (-21.99, -10.09) | -0.60 (-0.68, -0.54)* |
| Togo | 14.71 (9.67, 20.87) | 39.30 (25.98, 55.95) | 167.11 (148.18, 187.40) | 623.20 (417.89, 870.86) | 650.43 (437.99, 907.55) | 4.37 (-2.54, 10.90) | 0.15 (0.14, 0.16)* |
| Tokelau | 0.01 (0.00, 0.01) | 0.00 (0.00, 0.01) | -6.91 (-12.93, -0.56) | 366.85 (238.91, 536.62) | 360.55 (236.06, 524.84) | -1.72 (-6.85, 4.26) | -0.07 (-0.10, -0.04)* |
| Tonga | 0.23 (0.15, 0.33) | 0.26 (0.17, 0.36) | 11.31 (2.77, 20.42) | 272.96 (182.91, 385.30) | 268.31 (179.11, 377.26) | -1.71 (-8.52, 5.20) | -0.06 (-0.08, -0.05)* |
| Trinidad and Tobago | 7.19 (4.84, 10.22) | 9.38 (6.38, 13.11) | 30.48 (16.57, 44.29) | 642.28 (437.86, 907.10) | 601.65 (407.21, 846.96) | -6.33 (-14.05, 1.20) | -0.23 (-0.26, -0.19)* |
| Tunisia | 60.05 (39.92, 85.02) | 102.50 (68.90, 143.69) | 70.70 (54.78, 88.08) | 824.24 (555.57, 1147.52) | 820.27 (556.63, 1150.99) | -0.48 (-6.50, 6.26) | -0.02 (-0.04, -0.00)* |
| Turkey | 330.84 (228.61, 453.14) | 530.79 (359.67, 742.23) | 60.44 (43.90, 79.42) | 608.13 (423.96, 836.96) | 586.28 (398.91, 815.41) | -3.59 (-13.02, 7.08) | -0.12 (-0.24, -0.00)* |
| Turkmenistan | 12.54 (8.26, 17.95) | 19.08 (12.65, 26.60) | 52.15 (38.66, 67.73) | 437.78 (295.72, 608.48) | 398.87 (265.38, 551.10) | -8.89 (-16.34, -0.86) | -0.32 (-0.34, -0.30)* |
| Tuvalu | 0.03 (0.02, 0.05) | 0.04 (0.03, 0.06) | 35.97 (28.41, 43.98) | 366.42 (238.90, 536.50) | 360.82 (235.37, 526.63) | -1.53 (-6.96, 3.91) | -0.06 (-0.08, -0.04)* |
| Uganda | 121.57 (80.59, 175.48) | 288.32 (190.36, 412.35) | 137.16 (119.82, 154.19) | 1096.24 (738.63, 1539.96) | 1067.85 (712.97, 1499.41) | -2.59 (-9.28, 4.72) | -0.10 (-0.14, -0.05)* |
| Ukraine | 395.26 (271.98, 552.46) | 327.37 (223.60, 457.41) | -17.18 (-21.37, -12.56) | 648.60 (442.68, 910.85) | 575.17 (389.19, 805.62) | -11.32 (-15.26, -6.65) | -0.42 (-0.48, -0.36)* |
| United Arab Emirates | 10.56 (6.94, 15.12) | 61.02 (39.63, 87.42) | 477.84 (403.38, 561.86) | 578.04 (385.75, 815.19) | 527.39 (352.23, 736.31) | -8.76 (-14.99, -2.19) | -0.32 (-0.35, -0.29)* |
| United Kingdom | 454.21 (309.45, 631.18) | 464.42 (319.73, 643.26) | 2.25 (-0.11, 4.44) | 710.13 (482.24, 985.78) | 610.76 (412.21, 848.78) | -13.99 (-15.38, -12.73) | -0.51 (-0.61, -0.42)* |
| United Republic of Tanzania | 120.22 (79.47, 170.47) | 268.21 (178.58, 379.58) | 123.10 (108.85, 139.90) | 688.60 (460.73, 960.06) | 659.25 (443.75, 922.80) | -4.26 (-10.74, 2.82) | -0.15 (-0.17, -0.13)* |
| United States Virgin Islands | 0.50 (0.33, 0.71) | 0.61 (0.41, 0.85) | 21.91 (10.84, 34.80) | 485.19 (322.21, 676.36) | 502.53 (337.52, 704.33) | 3.57 (-2.99, 10.65) | 0.12 (0.08, 0.16)* |
| United States of America | 1341.83 (930.71, 1837.66) | 2242.30 (1552.73, 3056.52) | 67.11 (62.83, 72.26) | 497.26 (342.25, 683.81) | 661.72 (455.80, 902.24) | 33.07 (29.58, 36.62) | 0.96 (0.79, 1.12)* |
| Uruguay | 12.41 (8.40, 17.48) | 16.57 (11.37, 23.09) | 33.46 (22.73, 44.24) | 383.25 (259.00, 539.68) | 438.62 (299.37, 616.58) | 14.45 (5.44, 23.64) | 0.48 (0.44, 0.52)* |
| Uzbekistan | 73.22 (48.80, 103.90) | 129.74 (86.11, 183.21) | 77.19 (60.98, 93.98) | 453.10 (303.82, 641.20) | 420.83 (281.16, 589.83) | -7.12 (-15.12, 1.48) | -0.26 (-0.27, -0.24)* |
| Vanuatu | 0.44 (0.29, 0.64) | 0.88 (0.57, 1.26) | 97.92 (84.47, 112.42) | 354.34 (237.28, 500.10) | 328.77 (219.85, 466.39) | -7.22 (-13.21, -1.07) | -0.26 (-0.29, -0.24)* |
| Venezuela (Bolivarian Republic of) | 73.66 (48.62, 104.87) | 142.81 (95.41, 202.85) | 93.87 (76.56, 111.61) | 475.08 (322.98, 665.54) | 484.10 (323.88, 687.27) | 1.90 (-5.07, 8.56) | 0.06 (0.05, 0.08)* |
| Viet Nam | 149.77 (97.55, 213.79) | 231.53 (155.16, 323.45) | 54.60 (39.91, 73.38) | 251.56 (168.10, 352.34) | 223.17 (150.55, 312.30) | -11.28 (-17.73, -3.61) | -0.41 (-0.43, -0.38)* |
| Yemen | 74.85 (49.08, 105.93) | 207.93 (137.09, 295.47) | 177.79 (158.34, 197.85) | 774.41 (513.60, 1086.16) | 782.23 (526.14, 1087.55) | 1.01 (-5.55, 7.59) | 0.03 (0.02, 0.04)* |
| Zambia | 29.54 (19.31, 42.00) | 73.37 (48.56, 104.88) | 148.38 (130.55, 168.04) | 566.70 (380.08, 796.42) | 567.48 (380.75, 792.46) | 0.14 (-6.99, 7.98) | 0.00 (-0.02, 0.03) |
| Zimbabwe | 27.12 (18.10, 38.53) | 46.02 (30.91, 65.06) | 69.71 (58.62, 81.76) | 399.07 (268.58, 558.64) | 412.53 (279.42, 570.89) | 3.37 (-2.83, 10.26) | 0.12 (0.08, 0.16)* |

| Table S11 The number, PAFs and ASDR of MDD attributed to IPV in 1990 and 2019, and the corresponding percentage change and AAPC of ASDR during 1990-2019 by gender, SDI regions and GBD regions. | | | | | | | | | | |
| --- | --- | --- | --- | --- | --- | --- | --- | --- | --- | --- |
|  |  |  | Percentage change of |  |  | Percentage change of |  |  | Percentage change of | AAPC (95% CI) |
| Characteristics | IPV-related DALYs  number ×10^3^ (95% UI) | | number (%, 95% UI) | PAFs (%, 95% UI) | | PAFs (%, 95% UI) | IPV-related ASDR per 100 000 (95% UI) | | ASDR (%, 95% UI) | of IPV-related ASDR |
|  | 1990 year | 2019 year | during 1990-2019 | 1990 year | 2019 year | during 1990-2019 | 1990 year | 2019 year | during 1990-2019 | during 1990-2019 |
| **Global** | 1965.20 (8.58, 4338.62) | 3158.37 (13.87, 6921.79) | 60.72 (50.90, 72.48) | 8.42 (0.04, 18.28) | 8.43 (0.04, 18.20) | 0.07 (-2.36, 3.50) | 39.74 (0.17, 86.83) | 38.65 (0.17, 84.35) | -2.75 (-5.64, 1.06) | -0.07 (-0.20, 0.04) |
| **Gender** |  |  |  |  |  |  |  |  |  |  |
| Male | —— | —— | —— | —— | —— | —— | —— | —— | —— | —— |
| Female | 1965.20 (8.58, 4338.62) | 3158.37 (13.87, 6921.79) | 60.72 (50.90, 72.48) | 13.40 (0.07, 29.04) | 13.66 (0.07, 29.51) | 1.91 (-0.51, 29.51) | 79.25 (0.34, 29.51) | 76.94 (0.34, 29.51) | -2.91 (0.85, -5.83) | -0.08 (-0.20, 0.03) |
| **SDI regions** |  |  |  |  |  |  |  |  |  |  |
| High SDI | 349.61 (1.48, 776.84) | 473.49 (2.03, 1038.18) | 35.44 (30.29, 40.81) | 8.14 (0.04, 17.76) | 8.12 (0.04, 17.57) | -0.24 (-3.50, 3.44) | 38.53 (0.16, 85.94) | 42.48 (0.19, 94.60) | 10.25 (5.51, 15.12) | 0.33 (0.30, 0.36)* |
| High-middle SDI | 392.61 (1.62, 887.49) | 499.74 (2.02, 1142.89) | 27.29 (16.10, 38.81) | 7.46 (0.04, 16.61) | 7.03 (0.03, 15.67) | -5.80 (-8.96, -2.49) | 33.31 (0.14, 75.58) | 28.59 (0.12, 64.72) | -14.16 (-17.80, -10.59) | -0.55 (-0.66, -0.43)* |
| Middle SDI | 492.37 (2.13, 1128.59) | 824.08 (3.57, 1829.29) | 67.37 (47.99, 90.47) | 7.89 (0.04, 17.42) | 7.83 (0.04, 17.23) | -0.80 (-5.02, 4.45) | 31.70 (0.13, 70.23) | 31.11 (0.13, 69.60) | -1.86 (-6.52, 3.61) | -0.09 (-0.25, 0.07) |
| Low-middle SDI | 490.45 (2.23, 1075.27) | 846.07 (3.88, 1828.95) | 72.51 (61.50, 85.39) | 9.45 (0.05, 20.20) | 9.39 (0.05, 20.11) | -0.64 (-4.18, 3.73) | 54.69 (0.25, 118.74) | 50.16 (0.23, 108.97) | -8.29 (-12.47, -3.72) | -0.30 (-0.37, -0.24)* |
| Low SDI | 239.01 (1.12, 521.17) | 513.14 (2.36, 1114.64) | 114.69 (107.04, 123.53) | 10.14 (0.05, 21.27) | 10.18 (0.05, 21.46) | 0.41 (-2.17, 3.65) | 65.24 (0.30, 141.20) | 62.19 (0.28, 135.21) | -4.67 (-7.72, -1.14) | -0.18 (-0.26, -0.10)* |
| **GBD regions** |  |  |  |  |  |  |  |  |  |  |
| Central Asia | 12.96 (0.05, 30.90) | 19.90 (0.08, 47.21) | 53.55 (42.54, 65.81) | 5.04 (0.02, 11.92) | 5.07 (0.02, 12.02) | 0.70 (-2.27, 4.02) | 22.45 (0.09, 52.59) | 21.50 (0.08, 50.96) | -4.27 (-9.62, 1.57) | -0.15 (-0.17, -0.13)* |
| Central Europe | 33.23 (0.13, 75.30) | 30.62 (0.12, 71.50) | -7.86 (-16.51, 0.10) | 7.05 (0.03, 15.89) | 6.71 (0.03, 15.21) | -4.80 (-7.76, -1.87) | 24.58 (0.10, 55.50) | 20.42 (0.08, 46.15) | -16.95 (-21.95, -12.09) | -0.67 (-0.73, -0.60)* |
| Eastern Europe | 99.22 (0.40, 224.39) | 92.23 (0.37, 207.98) | -7.04 (-11.03, -3.54) | 7.70 (0.04, 17.22) | 7.67 (0.04, 17.00) | -0.39 (-3.05, 2.40) | 38.65 (0.16, 88.10) | 34.75 (0.14, 79.08) | -10.10 (-13.16, -6.93) | -0.36 (-0.39, -0.34)* |
| Australasia | 9.67 (0.04, 22.06) | 13.96 (0.06, 31.98) | 44.32 (27.08, 64.11) | 6.64 (0.03, 14.80) | 6.81 (0.03, 15.23) | 2.55 (-5.84, 11.34) | 44.22 (0.17, 100.84) | 45.93 (0.21, 108.28) | 3.88 (-8.98, 18.10) | 0.11 (-0.02, 0.25) |
| High-income Asia Pacific | 49.83 (0.21, 109.93) | 56.24 (0.24, 123.03) | 12.86 (4.40, 22.12) | 8.89 (0.05, 19.10) | 8.03 (0.04, 17.27) | -9.69 (-12.89, -6.79) | 25.60 (0.11, 56.16) | 24.46 (0.11, 54.39) | -4.43 (-8.32, -0.30) | -0.15 (-0.29, -0.02)* |
| High-income North America | 142.34 (0.64, 314.34) | 224.02 (0.99, 488.01) | 57.39 (49.32, 66.50) | 9.50 (0.05, 20.16) | 9.29 (0.05, 20.03) | -2.26 (-6.49, 2.30) | 46.61 (0.21, 103.02) | 59.27 (0.27, 132.37) | 27.14 (20.75, 33.28) | 0.81 (0.69, 0.93)* |
| Southern Latin America | 16.90 (0.08, 37.97) | 21.60 (0.10, 48.65) | 27.86 (16.67, 39.26) | 7.26 (0.04, 16.04) | 6.84 (0.04, 15.35) | -5.83 (-12.17, 0.14) | 35.04 (0.16, 78.47) | 29.98 (0.14, 67.68) | -14.46 (-22.01, -6.96) | -0.55 (-0.64, -0.47)* |
| Western Europe | 178.24 (0.72, 398.84) | 191.87 (0.78, 432.64) | 7.65 (1.09, 13.94) | 6.90 (0.03, 15.49) | 6.81 (0.03, 15.25) | -1.30 (-3.90, 1.55) | 40.93 (0.17, 92.55) | 38.70 (0.16, 87.49) | -5.44 (-10.22, -0.48) | -0.19 (-0.22, -0.16)* |
| Andean Latin America | 10.75 (0.05, 24.60) | 18.51 (0.08, 41.83) | 72.24 (51.50, 94.23) | 8.82 (0.04, 19.36) | 7.92 (0.04, 17.62) | -10.25 (-17.13, -4.33) | 34.86 (0.15, 77.56) | 29.34 (0.13, 66.08) | -15.84 (-23.50, -8.63) | -0.59 (-0.66, -0.52)* |
| Caribbean | 17.83 (0.07, 40.32) | 21.68 (0.09, 48.55) | 21.56 (8.56, 35.39) | 8.31 (0.04, 18.54) | 7.68 (0.04, 17.24) | -7.54 (-11.47, -3.83) | 53.55 (0.22, 119.46) | 43.44 (0.18, 97.62) | -18.89 (-25.25, -12.82) | -0.73 (-0.79, -0.67)* |
| Central Latin America | 49.58 (0.22, 111.10) | 111.37 (0.48, 248.81) | 124.64 (106.68, 142.76) | 8.68 (0.04, 18.78) | 8.99 (0.04, 19.44) | 3.60 (0.92, 6.31) | 37.47 (0.16, 83.78) | 42.94 (0.18, 96.50) | 14.60 (10.65, 18.57) | 0.54 (0.28, 0.80)* |
| Tropical Latin America | 47.75 (0.19, 114.58) | 77.44 (0.31, 179.94) | 62.19 (43.71, 80.53) | 5.56 (0.03, 13.11) | 5.20 (0.02, 12.31) | -6.46 (-9.67, -3.33) | 35.26 (0.15, 82.48) | 31.05 (0.12, 72.36) | -11.96 (-16.19, -7.53) | -0.48 (-0.56, -0.40)* |
| North Africa and Middle East | 191.96 (0.92, 421.22) | 412.12 (1.99, 899.75) | 114.69 (98.87, 130.53) | 10.51 (0.06, 21.96) | 10.04 (0.06, 21.13) | -4.44 (-7.34, -1.28) | 70.06 (0.33, 153.85) | 67.36 (0.32, 148.12) | -3.86 (-7.73, 0.15) | -0.14 (-0.15, -0.12)* |
| South Asia | 488.41 (2.23, 1072.17) | 851.75 (3.87, 1841.07) | 74.39 (61.27, 89.29) | 9.45 (0.05, 20.22) | 9.26 (0.05, 19.80) | -2.05 (-6.61, 3.12) | 55.93 (0.25, 122.12) | 49.03 (0.22, 107.01) | -12.34 (-17.02, -7.39) | -0.47 (-0.60, -0.34)* |
| East Asia | 316.87 (1.27, 733.92) | 390.96 (1.52, 903.06) | 23.38 (-1.82, 53.03) | 7.67 (0.04, 17.30) | 7.17 (0.03, 16.30) | -6.45 (-15.21, 3.24) | 25.48 (0.10, 57.58) | 20.33 (0.08, 46.52) | -20.20 (-28.30, -10.79) | -0.73 (-1.27, -0.18)* |
| Oceania | 2.13 (0.01, 4.48) | 4.59 (0.03, 9.49) | 116.12 (99.27, 135.35) | 10.86 (0.07, 22.10) | 11.11 (0.07, 22.15) | 2.31 (-3.55, 8.43) | 38.76 (0.21, 82.84) | 38.01 (0.21, 79.25) | -1.93 (-8.82, 5.20) | -0.06 (-0.08, -0.04)* |
| Southeast Asia | 65.69 (0.27, 156.70) | 104.97 (0.43, 241.44) | 59.81 (44.64, 76.38) | 6.03 (0.03, 13.61) | 5.76 (0.03, 13.09) | -4.41 (-6.57, -2.28) | 16.00 (0.07, 36.76) | 14.63 (0.06, 33.60) | -8.55 (-11.48, -5.38) | -0.34 (-0.47, -0.21)* |
| Central Sub-Saharan Africa | 39.61 (0.19, 87.94) | 96.98 (0.45, 209.20) | 144.85 (123.17, 172.43) | 11.01 (0.06, 22.83) | 11.16 (0.06, 22.98) | 1.34 (-5.22, 8.78) | 98.63 (0.47, 215.43) | 96.87 (0.44, 206.94) | -1.78 (-9.60, 7.26) | -0.06 (-0.16, 0.05) |
| Eastern Sub-Saharan Africa | 94.66 (0.44, 207.24) | 202.71 (0.94, 439.35) | 114.14 (104.08, 123.57) | 10.48 (0.06, 21.86) | 10.46 (0.06, 21.85) | -0.26 (-3.24, 3.23) | 78.33 (0.36, 169.68) | 73.57 (0.34, 158.83) | -6.09 (-9.88, -2.13) | -0.22 (-0.24, -0.20)* |
| Southern Sub-Saharan Africa | 26.84 (0.13, 59.44) | 47.74 (0.22, 102.90) | 77.89 (65.36, 91.73) | 11.47 (0.06, 24.03) | 11.17 (0.06, 23.61) | -2.67 (-6.83, 1.91) | 65.86 (0.30, 140.95) | 64.32 (0.29, 137.45) | -2.33 (-7.45, 2.64) | -0.06 (-0.12, -0.01)* |
| Western Sub-Saharan Africa | 70.76 (0.32, 157.53) | 167.10 (0.76, 371.56) | 136.13 (128.95, 144.96) | 9.12 (0.05, 19.48) | 9.73 (0.05, 20.59) | 6.68 (3.81, 10.22) | 54.57 (0.24, 119.40) | 54.66 (0.25, 119.93) | 0.16 (-2.74, 3.77) | 0.01 (-0.03, 0.04) |

| Table S12 The number, PAFs and ASDR of MDD attributed to CSA in 1990 and 2019, and the corresponding percentage change and AAPC of ASDR during 1990-2019 by gender, SDI regions and GBD regions. | | | | | | | | | | |
| --- | --- | --- | --- | --- | --- | --- | --- | --- | --- | --- |
|  |  |  | Percentage change of |  |  | Percentage change of |  |  | Percentage change of | AAPC (95% CI) |
| Characteristics | CSA-related DALYs  number ×10^3^ (95% UI) | | number (%, 95% UI) | PAFs (%, 95% UI) | | PAFs (%, 95% UI) | CSA-related ASDR per 100 000 (95% UI) | | ASDR (%, 95% UI) | of CSA-related ASDR |
|  | 1990 year | 2019 year | during 1990-2019 | 1990 year | 2019 year | during 1990-2019 | 1990 year | 2019 year | during1990-2019 | during 1990-2019 |
| **Global** | 1213.45 (563.47, 2107.94) | 2047.95 (960.75, 3562.75) | 68.77 (61.44, 77.24) | 5.21 (2.84, 8.23) | 5.46 (2.98, 8.57) | 4.86 (2.44, 7.39) | 24.65 (11.53, 42.98) | 25.11 (11.72, 43.38) | 1.89 (-0.77, 4.58) | 0.06 (0.03, 0.09)* |
| **Gender** |  |  |  |  |  |  |  |  |  |  |
| Male | 399.42 (176.03, 731.42) | 693.44 (308.93, 1277.24) | 73.61 (64.04, 83.36) | 4.67 (2.36, 7.88) | 4.83 (2.44, 8.16) | 3.53 (1.87, 8.16) | 16.44 (7.31, 30.34) | 17.10 (7.64, 30.34) | 4.03 (2.09, 6.03) | 0.13 (0.10, 0.16)* |
| Female | 814.03 (366.30, 1423.35) | 1354.51 (618.22, 2367.27) | 66.40 (57.93, 77.23) | 5.57 (2.97, 9.09) | 5.88 (3.17, 9.41) | 5.56 (1.96, 9.60) | 33.02 (15.03, 57.97) | 33.20 (15.07, 57.93) | 0.55 (-3.05, 4.60) | 0.02 (-0.02, 0.05) |
| **SDI regions** |  |  |  |  |  |  |  |  |  |  |
| High SDI | 225.76 (103.81, 390.66) | 339.72 (158.57, 592.11) | 50.48 (44.12, 58.46) | 5.29 (2.91, 8.36) | 5.88 (3.21, 9.19) | 11.07 (7.16, 16.77) | 25.10 (11.60, 43.74) | 30.82 (14.42, 53.47) | 22.79 (17.67, 29.48) | 0.71 (0.68, 0.74)* |
| High-middle SDI | 208.29 (94.97, 362.76) | 272.33 (124.34, 478.95) | 30.75 (23.06, 39.07) | 3.95 (2.13, 6.27) | 3.93 (2.14, 6.22) | -0.32 (-2.64, 2.12) | 17.68 (8.10, 30.73) | 16.05 (7.32, 27.88) | -9.20 (-11.63, -6.60) | -0.35 (-0.39, -0.31)* |
| Middle SDI | 263.97 (121.73, 460.79) | 487.86 (225.58, 853.59) | 84.82 (69.28, 103.01) | 4.26 (2.31, 6.74) | 4.63 (2.52, 7.27) | 8.59 (5.59, 11.81) | 17.19 (7.99, 30.01) | 18.46 (8.60, 31.99) | 7.41 (4.03, 11.03) | 0.23 (0.17, 0.28)* |
| Low-middle SDI | 348.48 (161.17, 610.85) | 596.65 (281.47, 1037.05) | 71.21 (62.20, 81.68) | 6.83 (3.74, 10.65) | 6.64 (3.67, 10.34) | -2.87 (-6.67, 0.55) | 39.63 (18.65, 69.13) | 35.52 (16.75, 61.64) | -10.37 (-14.46, -6.27) | -0.38 (-0.45, -0.32)* |
| Low SDI | 166.42 (77.29, 289.58) | 350.54 (162.01, 609.85) | 110.63 (105.52, 115.43) | 7.02 (3.86, 10.99) | 6.86 (3.80, 10.72) | -2.29 (-3.98, -0.71) | 45.25 (21.10, 78.21) | 41.97 (19.47, 72.89) | -7.24 (-9.45, -5.09) | -0.26 (-0.32, -0.21)* |
| **GBD regions** |  |  |  |  |  |  |  |  |  |  |
| Central Asia | 4.14 (1.86, 7.38) | 6.17 (2.75, 11.23) | 49.05 (38.64, 58.99) | 1.55 (0.82, 2.54) | 1.56 (0.82, 2.54) | 0.30 (-1.47, 2.27) | 6.93 (3.16, 12.39) | 6.61 (2.94, 11.73) | -4.68 (-8.72, -0.36) | -0.16 (-0.18, -0.14)* |
| Central Europe | 18.61 (8.48, 32.81) | 16.91 (7.53, 29.81) | -9.13 (-15.06, -2.23) | 4.01 (2.16, 6.35) | 4.02 (2.17, 6.38) | 0.16 (-2.22, 2.40) | 14.03 (6.39, 24.93) | 12.26 (5.51, 21.58) | -12.62 (-16.68, -8.57) | -0.47 (-0.51, -0.43)* |
| Eastern Europe | 57.04 (25.44, 101.41) | 51.38 (23.06, 92.00) | -9.92 (-14.25, -5.97) | 4.55 (2.45, 7.29) | 4.55 (2.43, 7.28) | -0.11 (-4.14, 3.18) | 22.93 (10.22, 39.98) | 20.67 (9.10, 36.41) | -9.86 (-14.08, -6.55) | -0.35 (-0.41, -0.30)* |
| Australasia | 7.72 (3.65, 13.14) | 12.10 (5.72, 20.91) | 56.59 (36.50, 76.50) | 5.32 (2.89, 8.17) | 5.90 (3.23, 9.18) | 10.90 (-1.20, 23.53) | 35.52 (16.74, 60.35) | 39.91 (18.77, 69.09) | 12.37 (-2.74, 27.99) | 0.42 (0.30, 0.53)* |
| High-income Asia Pacific | 30.73 (13.93, 54.05) | 37.43 (17.09, 65.80) | 21.79 (13.95, 30.48) | 5.45 (2.96, 8.71) | 5.61 (3.04, 8.97) | 2.98 (-0.10, 6.27) | 15.74 (7.16, 27.39) | 17.15 (7.76, 29.96) | 8.99 (4.78, 13.67) | 0.32 (0.19, 0.46)* |
| High-income North America | 84.66 (38.88, 148.21) | 168.60 (78.13, 293.20) | 99.15 (84.25, 117.55) | 5.66 (3.09, 9.10) | 6.95 (3.81, 10.85) | 22.84 (15.54, 32.10) | 27.83 (12.78, 49.35) | 44.49 (20.89, 77.22) | 59.85 (50.23, 72.68) | 1.62 (1.51, 1.73)* |
| Southern Latin America | 12.82 (5.91, 22.51) | 16.99 (7.70, 29.82) | 32.49 (24.57, 41.08) | 5.50 (3.01, 8.82) | 5.39 (2.97, 8.61) | -1.90 (-5.23, 1.32) | 26.63 (12.17, 46.64) | 23.69 (10.90, 41.55) | -11.02 (-16.80, -4.89) | -0.39 (-0.44, -0.34)* |
| Western Europe | 128.33 (59.16, 221.90) | 143.80 (66.73, 250.17) | 12.06 (5.28, 18.92) | 5.06 (2.77, 7.95) | 5.16 (2.84, 8.08) | 2.00 (-0.72, 4.70) | 30.11 (13.83, 51.71) | 29.42 (13.49, 51.17) | -2.27 (-6.54, 2.10) | -0.07 (-0.09, -0.06)* |
| Andean Latin America | 10.75 (5.06, 18.12) | 20.13 (9.24, 34.36) | 87.35 (68.75, 107.85) | 8.49 (4.75, 13.22) | 8.50 (4.73, 13.21) | 0.22 (-6.99, 7.68) | 33.63 (15.69, 57.59) | 31.64 (14.57, 53.95) | -5.90 (-13.66, 2.49) | -0.15 (-0.31, 0.01) |
| Caribbean | 6.94 (3.10, 12.47) | 9.00 (3.98, 15.96) | 29.70 (17.89, 42.41) | 3.24 (1.72, 5.29) | 3.19 (1.70, 5.15) | -1.59 (-4.20, 0.93) | 21.00 (9.28, 37.49) | 18.12 (7.95, 32.00) | -13.72 (-18.77, -8.61) | -0.51 (-0.53, -0.48)* |
| Central Latin America | 28.00 (12.50, 48.46) | 63.73 (28.63, 112.11) | 127.65 (111.94, 144.80) | 4.87 (2.62, 7.80) | 5.14 (2.77, 8.19) | 5.35 (2.04, 9.20) | 21.10 (9.57, 36.92) | 24.60 (11.09, 43.02) | 16.55 (11.91, 21.78) | 0.54 (0.49, 0.58)* |
| Tropical Latin America | 36.57 (15.82, 65.78) | 54.26 (23.86, 97.00) | 48.36 (33.87, 65.15) | 4.13 (2.15, 6.77) | 3.67 (1.95, 6.03) | -11.01 (-18.58, -3.58) | 26.28 (11.69, 47.18) | 22.02 (9.78, 39.23) | -16.20 (-24.15, -7.97) | -0.62 (-0.73, -0.51)* |
| North Africa and Middle East | 57.49 (25.85, 100.37) | 131.27 (58.36, 232.59) | 128.35 (112.36, 144.83) | 3.13 (1.64, 5.09) | 3.13 (1.64, 5.13) | -0.07 (-2.33, 2.14) | 20.93 (9.46, 36.33) | 21.04 (9.47, 37.11) | 0.53 (-2.90, 3.76) | 0.02 (-0.06, 0.09) |
| South Asia | 403.39 (187.65, 702.77) | 705.20 (330.35, 1229.12) | 74.82 (63.33, 88.00) | 7.96 (4.40, 12.41) | 7.73 (4.26, 11.92) | -2.86 (-7.30, 1.87) | 47.21 (22.42, 82.03) | 41.04 (19.32, 71.46) | -13.07 (-17.81, -8.17) | -0.50 (-0.60, -0.41)* |
| East Asia | 108.28 (48.01, 192.98) | 143.20 (64.49, 257.36) | 32.25 (10.05, 64.86) | 2.59 (1.37, 4.19) | 2.64 (1.40, 4.28) | 2.10 (-6.98, 13.04) | 8.63 (3.88, 15.40) | 7.51 (3.34, 13.41) | -12.92 (-21.33, -1.98) | -0.53 (-0.70, -0.36)* |
| Oceania | 0.79 (0.35, 1.38) | 1.69 (0.76, 2.99) | 113.33 (100.69, 126.54) | 4.05 (2.19, 6.42) | 4.06 (2.20, 6.45) | 0.35 (-1.73, 2.64) | 14.47 (6.45, 25.30) | 13.92 (6.31, 24.45) | -3.78 (-8.64, 0.93) | -0.14 (-0.15, -0.12)* |
| Southeast Asia | 39.14 (17.48, 70.35) | 67.10 (30.44, 119.79) | 71.45 (59.67, 84.20) | 3.62 (1.93, 5.87) | 3.66 (1.95, 5.89) | 1.15 (-0.76, 3.12) | 9.65 (4.43, 17.13) | 9.33 (4.24, 16.59) | -3.26 (-5.93, -0.34) | -0.12 (-0.20, -0.05)* |
| Central Sub-Saharan Africa | 22.68 (10.35, 40.07) | 55.06 (24.77, 96.69) | 142.77 (129.50, 156.30) | 6.21 (3.38, 9.72) | 6.19 (3.33, 9.67) | -0.43 (-2.27, 1.50) | 55.79 (25.48, 97.91) | 53.84 (24.41, 94.91) | -3.50 (-8.23, 1.43) | -0.12 (-0.13, -0.11)* |
| Eastern Sub-Saharan Africa | 57.39 (26.77, 99.33) | 128.32 (59.82, 223.97) | 123.58 (116.24, 130.50) | 6.26 (3.39, 9.74) | 6.35 (3.47, 9.82) | 1.33 (-0.47, 3.32) | 46.90 (21.73, 81.80) | 44.74 (20.96, 78.01) | -4.59 (-7.17, -2.00) | -0.16 (-0.19, -0.13)* |
| Southern Sub-Saharan Africa | 10.90 (4.99, 18.83) | 21.66 (10.05, 37.88) | 98.64 (85.12, 111.87) | 4.44 (2.39, 6.94) | 4.92 (2.65, 7.70) | 10.88 (6.82, 14.87) | 25.52 (11.89, 44.57) | 28.38 (13.26, 49.50) | 11.21 (6.40, 16.05) | 0.38 (0.33, 0.42)* |
| Western Sub-Saharan Africa | 87.07 (40.73, 148.38) | 193.96 (90.55, 327.12) | 122.75 (118.05, 127.46) | 10.71 (6.06, 16.43) | 10.56 (5.97, 16.26) | -1.46 (-3.27, 0.63) | 64.27 (29.85, 109.72) | 59.46 (27.48, 101.31) | -7.49 (-9.61, -4.99) | -0.28 (-0.37, -0.19)* |

| Table S13 The number, PAFs and ASDR of MDD attributed to BV in 1990 and 2019, and the corresponding percentage change and AAPC of ASDR during 1990-2019 by gender, SDI regions and GBD regions. | | | | | | | | | | |
| --- | --- | --- | --- | --- | --- | --- | --- | --- | --- | --- |
|  |  |  | Percentage change of |  |  | Percentage change of |  |  | Percentage change of | AAPC (95% CI) |
| Characteristics | BV-related DALYs  number ×10^3^ (95% UI) | | number (%, 95% UI) | PAFs (%, 95% UI) | | PAFs (%, 95% UI) | BV-related ASDR per 100 000 (95% UI) | | ASDR (%, 95% UI) | of BV-related ASDR |
|  | 1990 year | 2019 year | during1990-2019 | 1990 year | 2019 year | during 1990-2019 | 1990 year | 2019 year | during 1990-2019 | during 1990-2019 |
| **Global** | 1066.88 (213.43, 2554.17) | 1710.15 (353.09, 3995.31) | 60.30 (50.10, 79.96) | 3.73 (0.84, 8.11) | 4.86 (1.19, 10.09) | 30.22 (21.63, 51.57) | 17.74 (3.47, 43.11) | 22.46 (4.77, 52.18) | 26.60 (18.14, 47.22) | 0.81 (0.77, 0.85)* |
| **Gender** |  |  |  |  |  |  |  |  |  |  |
| Male | 475.67 (95.39, 1122.15) | 751.82 (153.05, 1770.65) | 58.05 (48.91, 75.00) | 4.43 (0.98, 9.48) | 5.45 (1.32, 11.23) | 23.17 (16.10, 41.11) | 15.64 (3.04, 37.11) | 19.37 (4.03, 45.36) | 23.84 (16.41, 41.27) | 0.73 (0.68, 0.77)* |
| Female | 591.20 (116.81, 1418.05) | 958.33 (200.07, 2245.95) | 62.10 (50.41, 85.70) | 3.34 (0.75, 7.30) | 4.53 (1.13, 9.33) | 35.43 (25.05, 60.49) | 19.89 (3.86, 48.18) | 25.68 (5.57, 60.05) | 29.07 (18.77, 52.90) | 0.89 (0.85, 0.92)* |
| **SDI regions** |  |  |  |  |  |  |  |  |  |  |
| High SDI | 169.09 (32.78, 409.74) | 266.42 (53.78, 609.69) | 57.56 (44.69, 81.63) | 4.52 (1.08, 9.79) | 6.67 (1.69, 13.53) | 47.50 (35.30, 71.85) | 21.53 (4.44, 50.56) | 35.12 (7.94, 78.65) | 63.12 (48.35, 89.14) | 1.68 (1.53, 1.83)* |
| High-middle SDI | 209.17 (40.51, 501.03) | 222.38 (44.48, 529.25) | 6.32 (-6.17, 28.64) | 3.74 (0.84, 8.04) | 4.69 (1.20, 9.48) | 25.48 (13.70, 59.61) | 16.80 (3.40, 40.14) | 19.21 (4.36, 43.73) | 14.37 (3.09, 43.24) | 0.44 (0.33, 0.55)* |
| Middle SDI | 329.03 (67.68, 773.20) | 439.58 (91.33, 1036.90) | 33.60 (20.70, 53.11) | 3.88 (0.88, 8.37) | 4.72 (1.21, 9.67) | 21.52 (13.09, 43.67) | 15.71 (3.11, 37.68) | 18.89 (4.21, 44.39) | 20.28 (11.73, 42.59) | 0.62 (0.56, 0.69)* |
| Low-middle SDI | 264.86 (52.09, 614.29) | 488.77 (99.42, 1142.09) | 84.54 (71.54, 105.68) | 3.57 (0.73, 7.83) | 4.56 (1.08, 9.49) | 27.98 (18.77, 49.89) | 20.76 (3.71, 49.41) | 24.53 (4.88, 57.98) | 18.15 (9.38, 38.43) | 0.57 (0.50, 0.65)* |
| Low SDI | 94.09 (18.94, 225.25) | 292.01 (64.84, 677.75) | 210.35 (188.83, 251.96) | 2.54 (0.54, 5.78) | 3.59 (0.83, 7.65) | 40.96 (31.23, 62.13) | 16.46 (3.01, 41.04) | 22.04 (4.39, 52.25) | 33.88 (24.36, 53.72) | 1.00 (0.95, 1.05)* |
| **GBD regions** |  |  |  |  |  |  |  |  |  |  |
| Central Asia | 5.03 (0.93, 12.43) | 6.35 (1.09, 16.14) | 26.09 (9.80, 38.80) | 1.43 (0.31, 3.31) | 1.56 (0.34, 3.59) | 8.57 (0.65, 18.15) | 6.44 (1.18, 16.01) | 6.64 (1.22, 16.51) | 3.21 (-5.19, 12.53) | 0.11 (0.04, 0.17)* |
| Central Europe | 11.96 (2.27, 29.32) | 8.40 (1.42, 20.56) | -29.72 (-42.43, -8.06) | 2.96 (0.66, 6.57) | 3.39 (0.77, 7.32) | 14.46 (-1.67, 51.84) | 10.39 (1.99, 25.15) | 10.38 (2.02, 24.13) | -0.06 (-14.58, 32.15) | -0.01 (-0.06, 0.04) |
| Eastern Europe | 38.65 (6.24, 98.74) | 32.97 (4.78, 86.10) | -14.69 (-25.12, -7.56) | 3.66 (0.76, 8.10) | 4.65 (0.96, 10.16) | 27.07 (19.84, 35.98) | 18.48 (3.28, 46.58) | 21.20 (3.75, 52.48) | 14.75 (8.10, 22.68) | 0.47 (0.42, 0.53)* |
| Australasia | 5.77 (1.12, 14.01) | 7.43 (1.61, 17.60) | 28.60 (10.52, 65.39) | 4.26 (1.00, 9.29) | 4.77 (1.24, 10.08) | 11.91 (-3.60, 46.85) | 28.57 (5.73, 68.80) | 32.41 (7.57, 73.75) | 13.44 (-2.86, 47.73) | 0.43 (0.39, 0.46)* |
| High-income Asia Pacific | 14.38 (2.73, 35.32) | 11.81 (2.11, 29.68) | -17.87 (-26.53, -11.46) | 2.87 (0.64, 6.45) | 3.24 (0.71, 7.22) | 12.89 (6.97, 21.21) | 8.32 (1.62, 20.44) | 9.94 (1.95, 24.12) | 19.56 (12.62, 28.37) | 0.62 (0.51, 0.73)* |
| High-income North America | 70.93 (13.95, 171.60) | 162.57 (34.83, 370.28) | 129.19 (104.30, 168.85) | 5.39 (1.31, 11.61) | 8.53 (2.06, 17.53) | 58.25 (42.30, 81.31) | 26.62 (5.75, 62.33) | 54.81 (12.49, 123.83) | 105.88 (83.81, 138.02) | 2.50 (2.39, 2.61)* |
| Southern Latin America | 13.73 (2.87, 31.49) | 17.63 (3.54, 40.89) | 28.38 (-4.37, 73.99) | 5.46 (1.32, 11.46) | 6.25 (1.47, 13.16) | 14.43 (-11.89, 57.14) | 26.57 (5.43, 61.36) | 27.60 (5.94, 62.95) | 3.90 (-20.69, 41.41) | 0.14 (0.11, 0.17)* |
| Western Europe | 91.81 (17.58, 219.84) | 85.43 (16.85, 201.21) | -6.96 (-21.08, 19.98) | 4.41 (1.04, 9.55) | 4.88 (1.17, 10.30) | 10.67 (-4.38, 44.67) | 26.35 (5.54, 61.18) | 27.98 (6.13, 64.33) | 6.18 (-9.24, 36.77) | 0.20 (0.14, 0.26)* |
| Andean Latin America | 5.82 (1.24, 13.81) | 9.10 (1.87, 21.49) | 56.18 (31.74, 89.53) | 3.22 (0.75, 7.24) | 3.60 (0.89, 7.78) | 12.03 (-2.05, 37.35) | 12.78 (2.54, 31.09) | 13.45 (2.80, 31.92) | 5.19 (-8.65, 29.67) | 0.17 (0.14, 0.21)* |
| Caribbean | 9.70 (1.97, 23.01) | 10.02 (2.13, 24.20) | 3.34 (-6.26, 15.21) | 3.58 (0.83, 7.98) | 3.75 (0.93, 8.09) | 4.68 (-2.49, 17.71) | 23.28 (4.61, 56.03) | 21.37 (4.64, 51.10) | -8.21 (-16.23, 3.56) | -0.30 (-0.33, -0.28)* |
| Central Latin America | 24.20 (5.10, 57.45) | 46.47 (9.25, 112.51) | 92.04 (66.53, 118.54) | 2.79 (0.63, 6.22) | 3.64 (0.84, 7.93) | 30.31 (20.07, 48.42) | 12.13 (2.39, 29.96) | 17.50 (3.52, 42.48) | 44.28 (32.42, 64.04) | 1.28 (1.26, 1.30)* |
| Tropical Latin America | 31.91 (6.52, 76.71) | 50.59 (11.27, 119.96) | 58.54 (41.78, 82.20) | 2.75 (0.62, 6.24) | 3.93 (1.03, 8.27) | 42.87 (26.73, 77.72) | 17.58 (3.42, 43.22) | 23.65 (5.56, 55.28) | 34.49 (17.36, 68.06) | 1.02 (0.83, 1.21)* |
| North Africa and Middle East | 109.05 (25.91, 245.46) | 243.08 (58.39, 558.69) | 122.90 (86.16, 195.97) | 4.01 (1.03, 8.59) | 5.47 (1.51, 10.89) | 36.46 (17.43, 83.12) | 26.92 (5.94, 62.54) | 36.97 (9.14, 83.85) | 37.34 (18.14, 84.40) | 1.08 (1.00, 1.17)* |
| South Asia | 281.08 (53.21, 659.11) | 517.80 (103.67, 1203.67) | 84.22 (70.04, 107.25) | 3.89 (0.77, 8.72) | 4.71 (1.06, 9.92) | 20.98 (11.51, 42.33) | 23.16 (4.00, 55.04) | 25.08 (4.92, 59.45) | 8.29 (-0.28, 27.54) | 0.26 (0.09, 0.43)* |
| East Asia | 199.67 (37.66, 478.02) | 111.89 (18.79, 286.77) | -43.96 (-53.70, -36.73) | 3.89 (0.85, 8.55) | 3.41 (0.80, 7.24) | -12.48 (-20.45, -0.19) | 13.02 (2.52, 31.64) | 9.72 (1.98, 23.12) | -25.37 (-31.69, -14.60) | -1.04 (-1.17, -0.92)* |
| Oceania | 1.23 (0.24, 2.91) | 2.54 (0.51, 5.96) | 107.27 (84.56, 134.18) | 4.45 (0.99, 9.88) | 4.89 (1.14, 10.70) | 9.83 (-0.21, 24.75) | 16.00 (3.04, 39.12) | 16.85 (3.31, 40.05) | 5.36 (-5.74, 20.76) | 0.18 (0.16, 0.20)* |
| Southeast Asia | 52.73 (11.11, 124.16) | 79.54 (17.13, 190.49) | 50.84 (38.53, 66.37) | 3.48 (0.82, 7.60) | 4.49 (1.15, 9.58) | 28.93 (20.88, 46.16) | 9.32 (1.93, 22.42) | 11.50 (2.53, 27.19) | 23.38 (15.52, 39.41) | 0.71 (0.61, 0.80)* |
| Central Sub-Saharan Africa | 19.46 (3.72, 47.23) | 62.88 (13.69, 145.91) | 223.10 (188.95, 284.88) | 3.53 (0.72, 8.04) | 4.72 (1.08, 10.22) | 33.55 (20.01, 59.12) | 31.83 (5.55, 79.10) | 41.19 (8.14, 99.15) | 29.43 (15.45, 55.29) | 0.90 (0.87, 0.92)* |
| Eastern Sub-Saharan Africa | 36.27 (7.67, 85.82) | 119.68 (27.05, 277.00) | 229.98 (201.79, 277.33) | 2.26 (0.49, 5.11) | 3.40 (0.80, 7.31) | 50.92 (37.25, 74.24) | 16.94 (3.22, 42.39) | 24.09 (4.85, 56.89) | 42.17 (29.18, 64.02) | 1.21 (1.16, 1.26)* |
| Southern Sub-Saharan Africa | 15.41 (3.37, 35.73) | 24.14 (4.57, 58.70) | 56.61 (33.05, 75.11) | 4.22 (0.97, 9.29) | 4.75 (1.08, 10.32) | 12.59 (4.64, 25.45) | 24.36 (5.00, 58.55) | 27.53 (5.41, 66.11) | 13.03 (4.59, 26.33) | 0.43 (0.41, 0.46)* |
| Western Sub-Saharan Africa | 28.07 (5.99, 67.86) | 99.85 (23.28, 231.17) | 255.74 (219.39, 308.40) | 2.20 (0.48, 5.10) | 3.30 (0.81, 7.19) | 50.09 (35.74, 71.58) | 13.23 (2.55, 33.52) | 18.64 (3.91, 44.46) | 40.92 (26.80, 61.90) | 1.19 (1.14, 1.23)* |

| Table S14 The APC of ASDR of MDD attributed to IPV, CSA and BV across globe, SDI regions and GBD regions. | | | | | | | | | | |
| --- | --- | --- | --- | --- | --- | --- | --- | --- | --- | --- |
|  | IPV-related ASDR |  |  |  | CSA-related ASDR |  |  |  | BV-related ASDR |  |
| Characteristics | Period | APC (95% CI) |  | Characteristics | Period | APC (95% CI) |  | Characteristics | Period | APC (95% CI) |
| Global | 1990-1992 | 2.58 (1.73 ,3.43)* |  | Global | 1990-1993 | 0.86 (0.71 ,1.01)* |  | Global | 1990-1994 | 1.87 (1.73 ,2.01)* |
| Global | 1992-1995 | 1.01 (0.17 ,1.85)* |  | Global | 1993-2005 | 0.30 (0.28 ,0.32)* |  | Global | 1994-2000 | 0.79 (0.70 ,0.89)* |
| Global | 1995-2000 | -1.45 (-1.71 ,-1.19)* |  | Global | 2005-2010 | -2.17 (-2.26 ,-2.08)* |  | Global | 2000-2005 | 1.58 (1.45 ,1.72)* |
| Global | 2000-2005 | 0.31 (0.04 ,0.57)* |  | Global | 2010-2015 | 0.98 (0.89 ,1.07)* |  | Global | 2005-2010 | -1.09 (-1.22 ,-0.96)* |
| Global | 2005-2010 | -1.79 (-2.05 ,-1.53)* |  | Global | 2015-2019 | 0.42 (0.32 ,0.51)* |  | Global | 2010-2019 | 0.98 (0.95 ,1.02)* |
| Global | 2010-2019 | 0.50 (0.42 ,0.58)* |  | High SDI | 1990-1996 | 1.99 (1.95 ,2.03)* |  | High SDI | 1990-1993 | 1.15 (0.71 ,1.58)* |
| High SDI | 1990-1998 | 1.22 (1.20 ,1.24)* |  | High SDI | 1996-1999 | 2.95 (2.73 ,3.17)* |  | High SDI | 1993-1996 | 2.46 (1.58 ,3.34)* |
| High SDI | 1998-2004 | 0.54 (0.50 ,0.59)* |  | High SDI | 1999-2002 | 0.90 (0.68 ,1.12)* |  | High SDI | 1996-1999 | 5.62 (4.73 ,6.51)* |
| High SDI | 2004-2009 | 0.01 (-0.05 ,0.08) |  | High SDI | 2002-2010 | 0.27 (0.24 ,0.30)* |  | High SDI | 1999-2002 | 2.57 (1.74 ,3.40)* |
| High SDI | 2009-2014 | -0.36 (-0.43 ,-0.29)* |  | High SDI | 2010-2015 | -1.14 (-1.21 ,-1.07)* |  | High SDI | 2002-2010 | 1.36 (1.26 ,1.47)* |
| High SDI | 2014-2017 | -0.77 (-0.98 ,-0.56)* |  | High SDI | 2015-2019 | 0.21 (0.14 ,0.28)* |  | High SDI | 2010-2019 | 0.32 (0.24 ,0.39)* |
| High SDI | 2017-2019 | 0.31 (0.10 ,0.53)* |  | High-middle SDI | 1990-1997 | -0.01 (-0.07 ,0.05) |  | High-middle SDI | 1990-1993 | 1.17 (0.67 ,1.67)* |
| High-middle SDI | 1990-1995 | 0.94 (0.71 ,1.18)* |  | High-middle SDI | 1997-2005 | -0.53 (-0.59 ,-0.47)* |  | High-middle SDI | 1993-2000 | 0.51 (0.35 ,0.68)* |
| High-middle SDI | 1995-2000 | -2.42 (-2.73 ,-2.10)* |  | High-middle SDI | 2005-2010 | -2.28 (-2.42 ,-2.14)* |  | High-middle SDI | 2000-2005 | 1.27 (0.97 ,1.57)* |
| High-middle SDI | 2000-2005 | -0.27 (-0.60 ,0.05) |  | High-middle SDI | 2010-2015 | 0.98 (0.83 ,1.13)* |  | High-middle SDI | 2005-2010 | -0.94 (-1.23 ,-0.66)* |
| High-middle SDI | 2005-2010 | -2.07 (-2.39 ,-1.76)* |  | High-middle SDI | 2015-2019 | 0.20 (0.05 ,0.35)* |  | High-middle SDI | 2010-2014 | 0.92 (0.46 ,1.38)* |
| High-middle SDI | 2010-2015 | 1.35 (1.03 ,1.68)* |  | Middle SDI | 1990-1995 | 0.64 (0.53 ,0.75)* |  | High-middle SDI | 2014-2019 | 0.10 (-0.11 ,0.31) |
| High-middle SDI | 2015-2019 | -0.83 (-1.16 ,-0.51)* |  | Middle SDI | 1995-2000 | -0.48 (-0.63 ,-0.32)* |  | Middle SDI | 1990-1994 | 1.62 (1.44 ,1.81)* |
| Middle SDI | 1990-1995 | 1.34 (1.02 ,1.66)* |  | Middle SDI | 2000-2005 | 0.60 (0.45 ,0.76)* |  | Middle SDI | 1994-2000 | -0.02 (-0.15 ,0.10) |
| Middle SDI | 1995-2000 | -2.96 (-3.39 ,-2.52)* |  | Middle SDI | 2005-2010 | -1.74 (-1.89 ,-1.58)* |  | Middle SDI | 2000-2005 | 1.62 (1.44 ,1.80)* |
| Middle SDI | 2000-2005 | 0.49 (0.05 ,0.94)* |  | Middle SDI | 2010-2015 | 2.08 (1.92 ,2.24)* |  | Middle SDI | 2005-2010 | -1.20 (-1.37 ,-1.03)* |
| Middle SDI | 2005-2010 | -1.28 (-1.72 ,-0.84)* |  | Middle SDI | 2015-2019 | 0.34 (0.18 ,0.49)* |  | Middle SDI | 2010-2014 | 1.36 (1.08 ,1.64)* |
| Middle SDI | 2010-2015 | 1.90 (1.45 ,2.36)* |  | Low-middle SDI | 1990-1993 | 1.02 (0.73 ,1.32)* |  | Middle SDI | 2014-2019 | 0.87 (0.74 ,0.99)* |
| Middle SDI | 2015-2019 | 0.09 (-0.36 ,0.54) |  | Low-middle SDI | 1993-2001 | -0.27 (-0.35 ,-0.20)* |  | Low-middle SDI | 1990-1994 | 3.26 (3.00 ,3.52)* |
| Low-middle SDI | 1990-1992 | 5.09 (4.62 ,5.57)* |  | Low-middle SDI | 2001-2005 | 0.68 (0.38 ,0.97)* |  | Low-middle SDI | 1994-2000 | -0.07 (-0.25 ,0.11) |
| Low-middle SDI | 1992-1995 | 1.85 (1.39 ,2.31)* |  | Low-middle SDI | 2005-2010 | -4.16 (-4.34 ,-3.99)* |  | Low-middle SDI | 2000-2005 | 1.71 (1.46 ,1.97)* |
| Low-middle SDI | 1995-2000 | -2.08 (-2.22 ,-1.94)* |  | Low-middle SDI | 2010-2015 | 1.31 (1.13 ,1.50)* |  | Low-middle SDI | 2005-2010 | -2.71 (-2.96 ,-2.47)* |
| Low-middle SDI | 2000-2005 | 0.58 (0.43 ,0.72)* |  | Low-middle SDI | 2015-2019 | -0.00 (-0.19 ,0.18) |  | Low-middle SDI | 2010-2019 | 1.05 (0.98 ,1.12)* |
| Low-middle SDI | 2005-2010 | -3.57 (-3.71 ,-3.43)* |  | Low SDI | 1990-1995 | 0.05 (-0.01 ,0.12) |  | Low SDI | 1990-1993 | 2.23 (2.04 ,2.41)* |
| Low-middle SDI | 2010-2019 | 0.17 (0.13 ,0.21)* |  | Low SDI | 1995-2003 | 0.29 (0.24 ,0.33)* |  | Low SDI | 1993-2000 | 1.03 (0.97 ,1.09)* |
| Low SDI | 1990-1994 | 1.40 (1.19 ,1.62)* |  | Low SDI | 2003-2006 | -0.19 (-0.49 ,0.11) |  | Low SDI | 2000-2006 | 1.37 (1.29 ,1.45)* |
| Low SDI | 1994-2006 | -0.11 (-0.16 ,-0.07)* |  | Low SDI | 2006-2009 | -3.00 (-3.30 ,-2.70)* |  | Low SDI | 2006-2009 | -1.81 (-2.15 ,-1.47)* |
| Low SDI | 2006-2009 | -2.77 (-3.41 ,-2.13)* |  | Low SDI | 2009-2012 | -0.66 (-0.96 ,-0.35)* |  | Low SDI | 2009-2012 | 0.69 (0.35 ,1.03)* |
| Low SDI | 2009-2014 | -0.78 (-0.98 ,-0.57)* |  | Low SDI | 2012-2019 | 0.21 (0.17 ,0.25)* |  | Low SDI | 2012-2019 | 1.48 (1.44 ,1.53)* |
| Low SDI | 2014-2019 | 0.56 (0.41 ,0.71)* |  | Central Asia | 1990-1994 | -0.90 (-0.97 ,-0.83)* |  | Central Asia | 1990-1995 | -0.11 (-0.21 ,-0.01)* |
| Central Asia | 1990-1993 | -0.44 (-0.53 ,-0.35)* |  | Central Asia | 1994-2005 | -0.01 (-0.03 ,0.00) |  | Central Asia | 1995-2003 | 0.59 (0.53 ,0.65)* |
| Central Asia | 1993-2001 | -0.15 (-0.18 ,-0.13)* |  | Central Asia | 2005-2010 | -0.74 (-0.81 ,-0.67)* |  | Central Asia | 2003-2006 | 0.16 (-0.30 ,0.62) |
| Central Asia | 2001-2006 | -0.56 (-0.61 ,-0.50)* |  | Central Asia | 2010-2015 | 0.69 (0.62 ,0.77)* |  | Central Asia | 2006-2010 | -0.73 (-0.96 ,-0.49)* |
| Central Asia | 2006-2010 | -0.85 (-0.94 ,-0.76)* |  | Central Asia | 2015-2019 | -0.13 (-0.21 ,-0.06)* |  | Central Asia | 2010-2014 | 0.44 (0.20 ,0.68)* |
| Central Asia | 2010-2017 | 0.56 (0.52 ,0.59)* |  | Central Europe | 1990-1993 | -0.84 (-1.00 ,-0.67)* |  | Central Asia | 2014-2019 | -0.08 (-0.18 ,0.02) |
| Central Asia | 2017-2019 | 0.24 (0.06 ,0.42)* |  | Central Europe | 1993-2000 | -0.61 (-0.67 ,-0.56)* |  | Central Europe | 1990-2002 | 0.00 (-0.02 ,0.03) |
| Central Europe | 1990-1999 | -0.30 (-0.36 ,-0.24)* |  | Central Europe | 2000-2005 | -1.36 (-1.47 ,-1.26)* |  | Central Europe | 2002-2009 | -0.60 (-0.67 ,-0.54)* |
| Central Europe | 1999-2005 | -1.47 (-1.61 ,-1.32)* |  | Central Europe | 2005-2010 | -0.86 (-0.97 ,-0.76)* |  | Central Europe | 2009-2012 | -0.21 (-0.58 ,0.16) |
| Central Europe | 2005-2010 | -2.82 (-3.02 ,-2.63)* |  | Central Europe | 2010-2017 | 0.11 (0.05 ,0.16)* |  | Central Europe | 2012-2017 | 0.15 (0.03 ,0.26)* |
| Central Europe | 2010-2017 | -0.50 (-0.61 ,-0.39)* |  | Central Europe | 2017-2019 | 1.79 (1.45 ,2.14)* |  | Central Europe | 2017-2019 | 1.91 (1.53 ,2.29)* |
| Central Europe | 2017-2019 | 5.12 (4.46 ,5.79)* |  | Eastern Europe | 1990-1993 | 1.10 (0.91 ,1.29)* |  | Eastern Europe | 1990-2001 | 1.14 (1.11 ,1.16)* |
| Eastern Europe | 1990-1996 | 0.18 (0.12 ,0.23)* |  | Eastern Europe | 1993-1996 | 0.28 (-0.10 ,0.66) |  | Eastern Europe | 2001-2005 | 0.40 (0.21 ,0.58)* |
| Eastern Europe | 1996-2002 | -0.56 (-0.63 ,-0.49)* |  | Eastern Europe | 1996-2001 | -0.62 (-0.73 ,-0.50)* |  | Eastern Europe | 2005-2010 | -0.92 (-1.04 ,-0.81)* |
| Eastern Europe | 2002-2010 | -1.25 (-1.30 ,-1.21)* |  | Eastern Europe | 2001-2010 | -1.32 (-1.36 ,-1.28)* |  | Eastern Europe | 2010-2014 | 1.13 (0.94 ,1.31)* |
| Eastern Europe | 2010-2015 | 0.74 (0.64 ,0.84)* |  | Eastern Europe | 2010-2016 | 0.32 (0.24 ,0.41)* |  | Eastern Europe | 2014-2017 | 0.51 (0.14 ,0.89)* |
| Eastern Europe | 2015-2019 | -0.46 (-0.56 ,-0.36)* |  | Eastern Europe | 2016-2019 | -0.44 (-0.64 ,-0.25)* |  | Eastern Europe | 2017-2019 | -0.87 (-1.23 ,-0.50)* |
| Australasia | 1990-1994 | -1.07 (-1.43 ,-0.71)* |  | Australasia | 1990-2000 | 0.32 (0.20 ,0.44)* |  | Australasia | 1990-1995 | -0.65 (-0.69 ,-0.60)* |
| Australasia | 1994-2000 | -0.03 (-0.28 ,0.23) |  | Australasia | 2000-2009 | 1.35 (1.17 ,1.52)* |  | Australasia | 1995-1999 | -1.26 (-1.37 ,-1.15)* |
| Australasia | 2000-2005 | 4.04 (3.66 ,4.42)* |  | Australasia | 2009-2015 | -2.10 (-2.45 ,-1.75)* |  | Australasia | 1999-2002 | 0.81 (0.58 ,1.04)* |
| Australasia | 2005-2010 | -1.64 (-2.01 ,-1.28)* |  | Australasia | 2015-2019 | 2.42 (1.90 ,2.94)* |  | Australasia | 2002-2005 | 1.30 (1.08 ,1.53)* |
| Australasia | 2010-2014 | -0.05 (-0.63 ,0.52) |  | High-income Asia Pacific | 1990-1993 | -2.23 (-2.63 ,-1.81)* |  | Australasia | 2005-2017 | 0.69 (0.67 ,0.70)* |
| Australasia | 2014-2019 | -0.71 (-0.96 ,-0.45)* |  | High-income Asia Pacific | 1993-1996 | 0.08 (-0.75 ,0.92) |  | Australasia | 2017-2019 | 3.11 (2.90 ,3.32)* |
| High-income Asia Pacific | 1990-1992 | -3.99 (-4.87 ,-3.09)* |  | High-income Asia Pacific | 1996-1999 | 3.73 (2.87 ,4.60)* |  | High-income Asia Pacific | 1990-1994 | -0.99 (-1.27 ,-0.71)* |
| High-income Asia Pacific | 1992-1995 | -1.70 (-2.60 ,-0.78)* |  | High-income Asia Pacific | 1999-2003 | 1.15 (0.73 ,1.57)* |  | High-income Asia Pacific | 1994-2006 | 0.28 (0.22 ,0.34)* |
| High-income Asia Pacific | 1995-2000 | 3.54 (3.23 ,3.84)* |  | High-income Asia Pacific | 2003-2010 | -0.17 (-0.31 ,-0.03)* |  | High-income Asia Pacific | 2006-2009 | 3.29 (2.37 ,4.22)* |
| High-income Asia Pacific | 2000-2010 | 0.20 (0.12 ,0.29)* |  | High-income Asia Pacific | 2010-2019 | 0.16 (0.08 ,0.24)* |  | High-income Asia Pacific | 2009-2014 | 1.99 (1.71 ,2.28)* |
| High-income Asia Pacific | 2010-2014 | -2.13 (-2.58 ,-1.68)* |  | High-income North America | 1990-1992 | 7.46 (6.31 ,8.62)* |  | High-income Asia Pacific | 2014-2019 | -0.23 (-0.42 ,-0.03)* |
| High-income Asia Pacific | 2014-2019 | -0.41 (-0.62 ,-0.20)* |  | High-income North America | 1992-1999 | 5.19 (5.00 ,5.38)* |  | High-income North America | 1990-1995 | 3.66 (3.32 ,3.99)* |
| High-income North America | 1990-1993 | 5.48 (5.06 ,5.90)* |  | High-income North America | 1999-2010 | 0.69 (0.61 ,0.77)* |  | High-income North America | 1995-2000 | 7.95 (7.47 ,8.43)* |
| High-income North America | 1993-1996 | 3.12 (2.31 ,3.94)* |  | High-income North America | 2010-2015 | -2.32 (-2.64 ,-2.00)* |  | High-income North America | 2000-2010 | 1.66 (1.54 ,1.79)* |
| High-income North America | 1996-2000 | 1.25 (0.85 ,1.66)* |  | High-income North America | 2015-2019 | 0.23 (-0.10 ,0.56) |  | High-income North America | 2010-2019 | -0.12 (-0.24 ,0.00) |
| High-income North America | 2000-2011 | 0.10 (0.03 ,0.16)* |  | Southern Latin America | 1990-1993 | -1.49 (-1.70 ,-1.28)* |  | Southern Latin America | 1990-1994 | 0.50 (0.43 ,0.57)* |
| High-income North America | 2011-2017 | -1.24 (-1.42 ,-1.06)* |  | Southern Latin America | 1993-2000 | -0.32 (-0.40 ,-0.25)* |  | Southern Latin America | 1994-2003 | 1.03 (1.00 ,1.05)* |
| High-income North America | 2017-2019 | -0.16 (-0.96 ,0.64) |  | Southern Latin America | 2000-2005 | 0.26 (0.12 ,0.39)* |  | Southern Latin America | 2003-2006 | 0.34 (0.14 ,0.55)* |
| Southern Latin America | 1990-2006 | -0.08 (-0.11 ,-0.05)* |  | Southern Latin America | 2005-2010 | -2.18 (-2.31 ,-2.04)* |  | Southern Latin America | 2006-2009 | -0.71 (-0.92 ,-0.51)* |
| Southern Latin America | 2006-2009 | -4.03 (-4.68 ,-3.39)* |  | Southern Latin America | 2010-2015 | 1.41 (1.27 ,1.55)* |  | Southern Latin America | 2009-2015 | -0.35 (-0.40 ,-0.31)* |
| Southern Latin America | 2009-2017 | -0.07 (-0.16 ,0.02) |  | Southern Latin America | 2015-2019 | -0.47 (-0.61 ,-0.34)* |  | Southern Latin America | 2015-2019 | -0.97 (-1.04 ,-0.91)* |
| Southern Latin America | 2017-2019 | -0.93 (-1.60 ,-0.26)* |  | Western Europe | 1990-1995 | -0.34 (-0.39 ,-0.30)* |  | Western Europe | 1990-1992 | -0.26 (-0.64 ,0.12) |
| Western Europe | 1990-2003 | -0.43 (-0.44 ,-0.42)* |  | Western Europe | 1995-2002 | 0.19 (0.15 ,0.22)* |  | Western Europe | 1992-1995 | 0.26 (-0.12 ,0.64) |
| Western Europe | 2003-2007 | -0.54 (-0.64 ,-0.42)* |  | Western Europe | 2002-2009 | -0.39 (-0.42 ,-0.35)* |  | Western Europe | 1995-2000 | 1.04 (0.92 ,1.16)* |
| Western Europe | 2007-2010 | -0.06 (-0.28 ,0.15) |  | Western Europe | 2009-2016 | 0.23 (0.20 ,0.27)* |  | Western Europe | 2000-2004 | 0.45 (0.26 ,0.64)* |
| Western Europe | 2010-2015 | 0.50 (0.43 ,0.57)* |  | Western Europe | 2016-2019 | -0.21 (-0.32 ,-0.11)* |  | Western Europe | 2004-2008 | -0.23 (-0.42 ,-0.05)* |
| Western Europe | 2015-2019 | -0.04 (-0.11 ,0.03) |  | Andean Latin America | 1990-1994 | -1.02 (-1.75 ,-0.29)* |  | Western Europe | 2008-2019 | -0.04 (-0.07 ,-0.02)* |
| Andean Latin America | 1990-2005 | -0.34 (-0.38 ,-0.31)* |  | Andean Latin America | 1994-2004 | -0.22 (-0.43 ,-0.01)* |  | Andean Latin America | 1990-1996 | 0.29 (0.24 ,0.35)* |
| Andean Latin America | 2005-2010 | -2.75 (-2.99 ,-2.51)* |  | Andean Latin America | 2004-2014 | -0.82 (-1.03 ,-0.61)* |  | Andean Latin America | 1996-2000 | -0.33 (-0.48 ,-0.18)* |
| Andean Latin America | 2010-2014 | 1.08 (0.69 ,1.48)* |  | Andean Latin America | 2014-2019 | 2.04 (1.51 ,2.58)* |  | Andean Latin America | 2000-2005 | 0.06 (-0.03 ,0.16) |
| Andean Latin America | 2014-2019 | -0.48 (-0.65 ,-0.30)* |  | Caribbean | 1990-2000 | -0.69 (-0.70 ,-0.68)* |  | Andean Latin America | 2005-2010 | -0.57 (-0.66 ,-0.48)* |
| Caribbean | 1990-1996 | -0.86 (-0.92 ,-0.80)* |  | Caribbean | 2000-2005 | -0.81 (-0.86 ,-0.76)* |  | Andean Latin America | 2010-2015 | 1.04 (0.95 ,1.14)* |
| Caribbean | 1996-2007 | -1.58 (-1.61 ,-1.55)* |  | Caribbean | 2005-2009 | -1.18 (-1.26 ,-1.10)* |  | Andean Latin America | 2015-2019 | 0.47 (0.37 ,0.56)* |
| Caribbean | 2007-2010 | -1.30 (-1.65 ,-0.95)* |  | Caribbean | 2009-2012 | -0.21 (-0.36 ,-0.05)* |  | Caribbean | 1990-1999 | -0.95 (-0.98 ,-0.92)* |
| Caribbean | 2010-2014 | 0.77 (0.59 ,0.95)* |  | Caribbean | 2012-2017 | 0.07 (0.02 ,0.12)* |  | Caribbean | 1999-2005 | -0.43 (-0.51 ,-0.36)* |
| Caribbean | 2014-2017 | 0.15 (-0.21 ,0.51) |  | Caribbean | 2017-2019 | 0.62 (0.47 ,0.78)* |  | Caribbean | 2005-2010 | -0.73 (-0.83 ,-0.62)* |
| Caribbean | 2017-2019 | 0.97 (0.61 ,1.33)* |  | Central Latin America | 1990-1996 | 0.39 (0.34 ,0.44)* |  | Caribbean | 2010-2019 | 0.68 (0.65 ,0.71)* |
| Central Latin America | 1990-1995 | -0.52 (-1.20 ,0.16) |  | Central Latin America | 1996-1999 | 0.87 (0.57 ,1.17)* |  | Central Latin America | 1990-1995 | 1.20 (1.16 ,1.24)* |
| Central Latin America | 1995-1999 | 1.73 (0.20 ,3.28)* |  | Central Latin America | 1999-2006 | 0.41 (0.36 ,0.46)* |  | Central Latin America | 1995-1999 | 1.91 (1.82 ,2.00)* |
| Central Latin America | 1999-2014 | 0.11 (-0.04 ,0.25) |  | Central Latin America | 2006-2009 | 0.93 (0.64 ,1.23)* |  | Central Latin America | 1999-2005 | 1.49 (1.45 ,1.53)* |
| Central Latin America | 2014-2019 | 1.99 (1.29 ,2.69)* |  | Central Latin America | 2009-2019 | 0.49 (0.47 ,0.52)* |  | Central Latin America | 2005-2010 | 0.99 (0.93 ,1.04)* |
| Tropical Latin America | 1990-1993 | 1.68 (1.29 ,2.08)* |  | Tropical Latin America | 1990-1992 | -2.39 (-3.14 ,-1.64)* |  | Central Latin America | 2010-2014 | 2.03 (1.94 ,2.11)* |
| Tropical Latin America | 1993-2005 | 0.66 (0.61 ,0.71)* |  | Tropical Latin America | 1992-1995 | -1.06 (-1.80 ,-0.31)* |  | Central Latin America | 2014-2019 | 0.32 (0.28 ,0.36)* |
| Tropical Latin America | 2005-2010 | -6.06 (-6.29 ,-5.83)* |  | Tropical Latin America | 1995-2000 | 3.40 (3.16 ,3.65)* |  | Tropical Latin America | 1990-1995 | 0.56 (0.22 ,0.91)* |
| Tropical Latin America | 2010-2017 | -0.84 (-0.97 ,-0.71)* |  | Tropical Latin America | 2000-2006 | -0.78 (-0.95 ,-0.61)* |  | Tropical Latin America | 1995-2003 | 4.18 (3.97 ,4.39)* |
| Tropical Latin America | 2017-2019 | 5.32 (4.50 ,6.14)* |  | Tropical Latin America | 2006-2010 | -4.42 (-4.78 ,-4.06)* |  | Tropical Latin America | 2003-2006 | 1.66 (0.17 ,3.16)* |
| North Africa and Middle East | 1990-1995 | -0.10 (-0.13 ,-0.07)* |  | Tropical Latin America | 2010-2019 | -0.43 (-0.50 ,-0.36)* |  | Tropical Latin America | 2006-2010 | -2.63 (-3.33 ,-1.92)* |
| North Africa and Middle East | 1995-2000 | -0.44 (-0.48 ,-0.40)* |  | North Africa and Middle East | 1990-1994 | -1.18 (-1.35 ,-1.02)* |  | Tropical Latin America | 2010-2019 | -0.05 (-0.18 ,0.08) |
| North Africa and Middle East | 2000-2005 | 0.44 (0.40 ,0.48)* |  | North Africa and Middle East | 1994-2000 | -0.07 (-0.19 ,0.05) |  | North Africa and Middle East | 1990-1993 | 0.10 (-0.31 ,0.52) |
| North Africa and Middle East | 2005-2010 | -0.64 (-0.68 ,-0.59)* |  | North Africa and Middle East | 2000-2004 | 0.41 (0.15 ,0.68)* |  | North Africa and Middle East | 1993-1997 | 0.90 (0.48 ,1.31)* |
| North Africa and Middle East | 2010-2015 | 0.46 (0.42 ,0.50)* |  | North Africa and Middle East | 2004-2011 | 0.19 (0.10 ,0.28)* |  | North Africa and Middle East | 1997-2006 | 1.56 (1.48 ,1.65)* |
| North Africa and Middle East | 2015-2019 | -0.63 (-0.67 ,-0.59)* |  | North Africa and Middle East | 2011-2014 | 1.12 (0.58 ,1.66)* |  | North Africa and Middle East | 2006-2014 | 1.31 (1.21 ,1.42)* |
| South Asia | 1990-1992 | 6.95 (5.97 ,7.94)* |  | North Africa and Middle East | 2014-2019 | -0.13 (-0.24 ,-0.01)* |  | North Africa and Middle East | 2014-2019 | 0.61 (0.43 ,0.78)* |
| South Asia | 1992-1995 | 2.57 (1.64 ,3.51)* |  | South Asia | 1990-1994 | 1.17 (0.89 ,1.44)* |  | South Asia | 1990-1994 | 4.34 (3.88 ,4.80)* |
| South Asia | 1995-2000 | -2.61 (-2.89 ,-2.32)* |  | South Asia | 1994-2001 | -0.49 (-0.63 ,-0.34)* |  | South Asia | 1994-2000 | -0.63 (-0.94 ,-0.32)* |
| South Asia | 2000-2005 | 0.87 (0.57 ,1.16)* |  | South Asia | 2001-2005 | 1.06 (0.63 ,1.50)* |  | South Asia | 2000-2006 | 1.28 (0.96 ,1.59)* |
| South Asia | 2005-2010 | -4.96 (-5.24 ,-4.69)* |  | South Asia | 2005-2010 | -5.45 (-5.71 ,-5.20)* |  | South Asia | 2006-2009 | -6.63 (-7.92 ,-5.33)* |
| South Asia | 2010-2019 | -0.05 (-0.14 ,0.03) |  | South Asia | 2010-2015 | 1.76 (1.48 ,2.04)* |  | South Asia | 2009-2019 | 0.73 (0.62 ,0.83)* |
| East Asia | 1990-1993 | 3.73 (1.92 ,5.58)* |  | South Asia | 2015-2019 | -0.20 (-0.47 ,0.07) |  | East Asia | 1990-1995 | 0.65 (0.39 ,0.91)* |
| East Asia | 1993-1996 | -1.36 (-4.75 ,2.15) |  | East Asia | 1990-1995 | 1.73 (1.38 ,2.09)* |  | East Asia | 1995-2000 | -2.71 (-3.06 ,-2.36)* |
| East Asia | 1996-1999 | -10.24 (-13.34 ,-7.02)* |  | East Asia | 1995-2000 | -3.53 (-4.00 ,-3.05)* |  | East Asia | 2000-2005 | 0.35 (0.00 ,0.70)* |
| East Asia | 1999-2010 | -0.76 (-1.03 ,-0.48)* |  | East Asia | 2000-2005 | -0.98 (-1.46 ,-0.49)* |  | East Asia | 2005-2010 | -3.99 (-4.31 ,-3.67)* |
| East Asia | 2010-2015 | 4.32 (3.15 ,5.50)* |  | East Asia | 2005-2010 | -2.17 (-2.65 ,-1.69)* |  | East Asia | 2010-2017 | -0.42 (-0.61 ,-0.24)* |
| East Asia | 2015-2019 | -2.08 (-3.17 ,-0.97)* |  | East Asia | 2010-2015 | 2.81 (2.31 ,3.31)* |  | East Asia | 2017-2019 | 0.84 (-0.27 ,1.97) |
| Oceania | 1990-1996 | 0.20 (0.17 ,0.23)* |  | East Asia | 2015-2019 | -1.02 (-1.51 ,-0.54)* |  | Oceania | 1990-1999 | 0.00 (-0.02 ,0.02) |
| Oceania | 1996-2000 | -0.36 (-0.44 ,-0.27)* |  | Oceania | 1990-1996 | -0.02 (-0.04 ,-0.01)* |  | Oceania | 1999-2006 | 0.23 (0.20 ,0.26)* |
| Oceania | 2000-2005 | 0.41 (0.36 ,0.46)* |  | Oceania | 1996-1999 | -0.64 (-0.72 ,-0.56)* |  | Oceania | 2006-2009 | 0.01 (-0.17 ,0.19) |
| Oceania | 2005-2017 | -0.12 (-0.13 ,-0.11)* |  | Oceania | 1999-2006 | -0.14 (-0.15 ,-0.12)* |  | Oceania | 2009-2015 | 0.42 (0.38 ,0.46)* |
| Oceania | 2017-2019 | -1.10 (-1.26 ,-0.93)* |  | Oceania | 2006-2009 | -0.32 (-0.40 ,-0.24)* |  | Oceania | 2015-2019 | 0.26 (0.21 ,0.32)* |
| Southeast Asia | 1990-1995 | -0.35 (-0.65 ,-0.05)* |  | Oceania | 2009-2017 | -0.07 (-0.08 ,-0.06)* |  | Southeast Asia | 1990-1995 | 0.35 (0.17 ,0.54)* |
| Southeast Asia | 1995-2000 | -0.94 (-1.35 ,-0.52)* |  | Oceania | 2017-2019 | 0.27 (0.19 ,0.35)* |  | Southeast Asia | 1995-2000 | -0.45 (-0.70 ,-0.19)* |
| Southeast Asia | 2000-2005 | -0.30 (-0.72 ,0.12) |  | Southeast Asia | 1990-2000 | -0.65 (-0.68 ,-0.61)* |  | Southeast Asia | 2000-2005 | 1.46 (1.20 ,1.72)* |
| Southeast Asia | 2005-2010 | -1.22 (-1.64 ,-0.80)* |  | Southeast Asia | 2000-2006 | -0.01 (-0.12 ,0.10) |  | Southeast Asia | 2005-2010 | 0.20 (-0.06 ,0.45) |
| Southeast Asia | 2010-2019 | 0.46 (0.34 ,0.59)* |  | Southeast Asia | 2006-2010 | -0.53 (-0.78 ,-0.28)* |  | Southeast Asia | 2010-2014 | 2.02 (1.62 ,2.42)* |
| Central Sub-Saharan Africa | 1990-1998 | -0.01 (-0.09 ,0.06) |  | Southeast Asia | 2010-2014 | 0.81 (0.56 ,1.06)* |  | Southeast Asia | 2014-2019 | 0.94 (0.76 ,1.12)* |
| Central Sub-Saharan Africa | 1998-2011 | -0.35 (-0.39 ,-0.31)* |  | Southeast Asia | 2014-2017 | -0.01 (-0.51 ,0.49) |  | Central Sub-Saharan Africa | 1990-1995 | 0.15 (0.11 ,0.19)* |
| Central Sub-Saharan Africa | 2011-2014 | -0.97 (-1.65 ,-0.29)* |  | Southeast Asia | 2017-2019 | 0.97 (0.46 ,1.49)* |  | Central Sub-Saharan Africa | 1995-2004 | 0.44 (0.42 ,0.46)* |
| Central Sub-Saharan Africa | 2014-2017 | 1.71 (1.02 ,2.41)* |  | Central Sub-Saharan Africa | 1990-2000 | -0.16 (-0.16 ,-0.15)* |  | Central Sub-Saharan Africa | 2004-2007 | 0.82 (0.65 ,0.99)* |
| Central Sub-Saharan Africa | 2017-2019 | 0.40 (-0.28 ,1.08) |  | Central Sub-Saharan Africa | 2000-2010 | -0.27 (-0.28 ,-0.26)* |  | Central Sub-Saharan Africa | 2007-2010 | 1.10 (0.93 ,1.27)* |
| Eastern Sub-Saharan Africa | 1990-1994 | -0.24 (-0.29 ,-0.18)* |  | Central Sub-Saharan Africa | 2010-2014 | 0.13 (0.09 ,0.16)* |  | Central Sub-Saharan Africa | 2010-2015 | 1.62 (1.57 ,1.68)* |
| Eastern Sub-Saharan Africa | 1994-2000 | 0.41 (0.37 ,0.44)* |  | Central Sub-Saharan Africa | 2014-2017 | -0.08 (-0.15 ,-0.00)* |  | Central Sub-Saharan Africa | 2015-2019 | 1.86 (1.80 ,1.91)* |
| Eastern Sub-Saharan Africa | 2000-2006 | -0.46 (-0.49 ,-0.42)* |  | Central Sub-Saharan Africa | 2017-2019 | 0.22 (0.14 ,0.29)* |  | Eastern Sub-Saharan Africa | 1990-2000 | 1.68 (1.64 ,1.72)* |
| Eastern Sub-Saharan Africa | 2006-2010 | -1.11 (-1.19 ,-1.03)* |  | Eastern Sub-Saharan Africa | 1990-1995 | 0.00 (-0.07 ,0.07) |  | Eastern Sub-Saharan Africa | 2000-2006 | 1.18 (1.08 ,1.29)* |
| Eastern Sub-Saharan Africa | 2010-2014 | -0.40 (-0.49 ,-0.32)* |  | Eastern Sub-Saharan Africa | 1995-2000 | 0.42 (0.33 ,0.52)* |  | Eastern Sub-Saharan Africa | 2006-2010 | -0.07 (-0.31 ,0.16) |
| Eastern Sub-Saharan Africa | 2014-2019 | 0.19 (0.15 ,0.23)* |  | Eastern Sub-Saharan Africa | 2000-2006 | -0.36 (-0.43 ,-0.29)* |  | Eastern Sub-Saharan Africa | 2010-2014 | 1.03 (0.79 ,1.27)* |
| Southern Sub-Saharan Africa | 1990-1994 | -1.62 (-1.74 ,-1.50)* |  | Eastern Sub-Saharan Africa | 2006-2014 | -0.82 (-0.86 ,-0.78)* |  | Eastern Sub-Saharan Africa | 2014-2019 | 1.47 (1.36 ,1.58)* |
| Southern Sub-Saharan Africa | 1994-2000 | -0.23 (-0.31 ,-0.14)* |  | Eastern Sub-Saharan Africa | 2014-2019 | 0.40 (0.33 ,0.47)* |  | Southern Sub-Saharan Africa | 1990-1994 | -0.83 (-0.92 ,-0.73)* |
| Southern Sub-Saharan Africa | 2000-2005 | -0.93 (-1.04 ,-0.81)* |  | Southern Sub-Saharan Africa | 1990-1994 | -1.33 (-1.45 ,-1.21)* |  | Southern Sub-Saharan Africa | 1994-2001 | 0.61 (0.56 ,0.66)* |
| Southern Sub-Saharan Africa | 2005-2014 | 0.79 (0.75 ,0.83)* |  | Southern Sub-Saharan Africa | 1994-2000 | -0.39 (-0.48 ,-0.31)* |  | Southern Sub-Saharan Africa | 2001-2005 | 0.11 (-0.04 ,0.26) |
| Southern Sub-Saharan Africa | 2014-2017 | 1.78 (1.39 ,2.17)* |  | Southern Sub-Saharan Africa | 2000-2005 | 0.32 (0.19 ,0.44)* |  | Southern Sub-Saharan Africa | 2005-2015 | 1.14 (1.12 ,1.17)* |
| Southern Sub-Saharan Africa | 2017-2019 | -0.84 (-1.22 ,-0.47)* |  | Southern Sub-Saharan Africa | 2005-2009 | 2.35 (2.16 ,2.55)* |  | Southern Sub-Saharan Africa | 2015-2019 | -0.04 (-0.13 ,0.06) |
| Western Sub-Saharan Africa | 1990-1995 | -0.08 (-0.14 ,-0.02)* |  | Southern Sub-Saharan Africa | 2009-2017 | 1.11 (1.06 ,1.16)* |  | Western Sub-Saharan Africa | 1990-1995 | 1.18 (1.06 ,1.29)* |
| Western Sub-Saharan Africa | 1995-2000 | 1.64 (1.55 ,1.72)* |  | Southern Sub-Saharan Africa | 2017-2019 | -0.53 (-0.91 ,-0.15)* |  | Western Sub-Saharan Africa | 1995-2000 | 4.70 (4.53 ,4.86)* |
| Western Sub-Saharan Africa | 2000-2006 | -0.14 (-0.20 ,-0.08)* |  | Western Sub-Saharan Africa | 1990-1992 | -1.86 (-2.50 ,-1.22)* |  | Western Sub-Saharan Africa | 2000-2006 | 1.57 (1.46 ,1.68)* |
| Western Sub-Saharan Africa | 2006-2010 | -1.36 (-1.49 ,-1.23)* |  | Western Sub-Saharan Africa | 1992-1995 | -0.88 (-1.52 ,-0.23)* |  | Western Sub-Saharan Africa | 2006-2014 | -1.13 (-1.19 ,-1.06)* |
| Western Sub-Saharan Africa | 2010-2014 | -0.88 (-1.01 ,-0.75)* |  | Western Sub-Saharan Africa | 1995-2000 | 1.80 (1.59 ,2.01)* |  | Western Sub-Saharan Africa | 2014-2019 | 1.03 (0.92 ,1.14)* |
| Western Sub-Saharan Africa | 2014-2019 | 0.46 (0.40 ,0.52)* |  | Western Sub-Saharan Africa | 2000-2006 | 0.07 (-0.07 ,0.21) |  |  |  |  |
|  |  |  |  | Western Sub-Saharan Africa | 2006-2011 | -1.86 (-2.06 ,-1.66)* |  |  |  |  |
|  |  |  |  | Western Sub-Saharan Africa | 2011-2019 | -0.20 (-0.27 ,-0.13)* |  |  |  |  |

| Table S15 The number, PAFs and ASDR of MDD attributed to IPV in 1990 and 2019, and the corresponding percentage change and AAPC of ASDR during 1990-2019 across 204 countries and territories. | | | | | | | | | | |
| --- | --- | --- | --- | --- | --- | --- | --- | --- | --- | --- |
|  |  |  | Percentage change of |  |  | Percentage change of |  |  | Percentage change of | AAPC (95% CI) |
| Countries or territories | IPV-related DALYs number ×10^3^ (95% UI) | | number (%, 95% UI) | PAFs (%, 95% UI) | | PAFs (%, 95% UI) | IPV-related ASDR per 100 000 (95% UI) | | ASDR (%, 95% UI) | of IPV-related ASDR |
|  | 1990 year | 2019 year | during 1990-2019 | 1990 year | 2019 year | during 1990-2019 | 1990 year | 2019 year | Period of 1990-2019 | during 1990-2019 |
| Afghanistan | 7.73 (0.04, 17.00) | 24.28 (0.13, 52.26) | 214.18 (172.63, 266.57) | 11.88 (0.07, 24.32) | 11.08 (0.07, 22.77) | -6.70 (-17.00, 6.16) | 93.18 (0.46, 199.54) | 86.62 (0.45, 181.49) | -7.04 (-19.41, 7.29) | -0.27 (-0.34, -0.19)* |
| Albania | 0.40 (0.00, 0.96) | 0.47 (0.00, 1.14) | 19.07 (-1.78, 44.47) | 5.20 (0.02, 12.05) | 5.29 (0.02, 12.40) | 1.74 (-3.91, 7.58) | 13.46 (0.05, 32.80) | 14.57 (0.05, 36.24) | 8.20 (-2.11, 20.51) | 0.27 (0.23, 0.32)* |
| Algeria | 12.56 (0.06, 27.95) | 27.13 (0.13, 60.38) | 115.99 (88.37, 145.28) | 9.99 (0.05, 21.33) | 10.00 (0.05, 21.15) | 0.09 (-5.78, 5.92) | 65.14 (0.30, 140.84) | 62.58 (0.29, 140.32) | -3.93 (-12.48, 5.44) | -0.14 (-0.17, -0.10)* |
| American Samoa | 0.01 (0.00, 0.02) | 0.01 (0.00, 0.03) | 29.12 (11.32, 48.39) | 9.69 (0.05, 20.10) | 10.16 (0.06, 21.47) | 4.94 (-2.28, 12.63) | 26.03 (0.13, 58.19) | 26.36 (0.13, 58.61) | 1.27 (-8.42, 11.83) | 0.04 (0.02, 0.06)* |
| Andorra | 0.02 (0.00, 0.05) | 0.03 (0.00, 0.07) | 56.86 (31.27, 85.10) | 5.52 (0.03, 12.85) | 5.66 (0.03, 13.05) | 2.51 (-3.16, 9.22) | 29.29 (0.12, 70.42) | 29.18 (0.12, 69.72) | -0.37 (-9.07, 9.88) | -0.01 (-0.13, 0.11) |
| Angola | 7.65 (0.04, 17.15) | 22.63 (0.11, 49.73) | 195.89 (167.14, 228.88) | 10.43 (0.06, 21.78) | 11.35 (0.06, 23.38) | 8.83 (2.92, 15.38) | 99.97 (0.47, 219.04) | 103.75 (0.48, 225.04) | 3.78 (-5.86, 14.77) | 0.13 (0.12, 0.14)* |
| Antigua and Barbuda | 0.02 (0.00, 0.05) | 0.04 (0.00, 0.09) | 74.03 (53.24, 98.25) | 8.74 (0.04, 19.20) | 8.60 (0.04, 19.15) | -1.59 (-6.03, 3.26) | 37.86 (0.16, 88.50) | 37.22 (0.15, 87.62) | -1.70 (-9.39, 6.20) | -0.07 (-0.16, 0.03) |
| Argentina | 5.14 (0.02, 12.71) | 6.97 (0.03, 16.61) | 35.45 (17.68, 57.12) | 4.19 (0.02, 10.17) | 3.97 (0.02, 9.58) | -5.37 (-12.87, 2.35) | 16.15 (0.07, 39.96) | 14.56 (0.06, 34.75) | -9.81 (-22.18, 4.89) | -0.35 (-0.38, -0.32)* |
| Armenia | 0.58 (0.00, 1.40) | 0.68 (0.00, 1.63) | 17.05 (1.99, 35.28) | 5.12 (0.02, 12.04) | 4.78 (0.02, 11.42) | -6.55 (-11.58, -1.14) | 17.92 (0.06, 43.50) | 18.25 (0.07, 44.50) | 1.85 (-7.29, 12.61) | 0.05 (-0.02, 0.13) |
| Australia | 7.67 (0.03, 17.79) | 11.39 (0.05, 26.57) | 48.53 (27.06, 74.21) | 6.04 (0.03, 13.82) | 6.29 (0.03, 14.30) | 4.15 (-6.06, 15.87) | 42.02 (0.16, 97.96) | 43.77 (0.19, 103.92) | 4.16 (-11.21, 22.56) | 0.12 (-0.06, 0.29) |
| Austria | 1.86 (0.01, 4.49) | 1.70 (0.01, 4.01) | -8.36 (-21.23, 5.46) | 4.04 (0.02, 9.66) | 3.97 (0.02, 9.54) | -1.73 (-8.94, 5.52) | 20.95 (0.08, 51.29) | 16.41 (0.06, 39.08) | -21.69 (-31.32, -11.36) | -0.86 (-0.94, -0.79)* |
| Azerbaijan | 1.45 (0.01, 3.51) | 2.50 (0.01, 5.91) | 71.77 (50.86, 94.75) | 6.79 (0.03, 15.80) | 6.50 (0.03, 15.23) | -4.26 (-8.99, 0.87) | 22.85 (0.09, 53.75) | 21.74 (0.08, 51.76) | -4.83 (-12.80, 3.91) | -0.19 (-0.34, -0.05)* |
| Bahamas | 0.09 (0.00, 0.22) | 0.16 (0.00, 0.37) | 72.58 (48.49, 97.59) | 8.40 (0.04, 18.67) | 8.53 (0.04, 18.84) | 1.49 (-3.25, 6.61) | 37.66 (0.17, 86.84) | 37.49 (0.16, 87.91) | -0.47 (-8.52, 8.63) | -0.02 (-0.13, 0.09) |
| Bahrain | 0.31 (0.00, 0.70) | 0.91 (0.00, 2.01) | 192.03 (138.28, 254.08) | 7.86 (0.04, 16.64) | 7.52 (0.04, 16.08) | -4.32 (-12.20, 4.94) | 64.79 (0.30, 141.23) | 53.89 (0.24, 119.16) | -16.81 (-25.89, -6.86) | -0.63 (-0.67, -0.60)* |
| Bangladesh | 68.40 (0.35, 147.10) | 138.99 (0.73, 298.26) | 103.21 (74.65, 137.12) | 11.52 (0.06, 23.53) | 12.47 (0.07, 25.49) | 8.21 (1.37, 16.20) | 84.47 (0.43, 181.16) | 87.65 (0.46, 188.47) | 3.76 (-6.70, 16.77) | 0.14 (0.08, 0.21)* |
| Barbados | 0.10 (0.00, 0.23) | 0.13 (0.00, 0.31) | 32.76 (14.33, 53.65) | 8.23 (0.04, 18.34) | 8.12 (0.04, 17.91) | -1.36 (-6.78, 4.52) | 37.75 (0.16, 87.01) | 37.75 (0.16, 89.15) | 0.01 (-7.83, 8.92) | 0.00 (-0.09, 0.09) |
| Belarus | 5.40 (0.02, 12.43) | 5.44 (0.02, 12.52) | 0.85 (-11.24, 14.43) | 7.93 (0.04, 17.72) | 7.92 (0.04, 17.59) | -0.19 (-6.40, 6.06) | 45.60 (0.18, 103.80) | 44.03 (0.18, 99.53) | -3.45 (-14.70, 8.52) | -0.12 (-0.13, -0.10)* |
| Belgium | 3.55 (0.01, 8.19) | 4.43 (0.02, 10.04) | 24.94 (7.63, 45.20) | 6.66 (0.03, 15.14) | 6.73 (0.03, 14.99) | 1.02 (-5.62, 8.69) | 31.21 (0.11, 71.33) | 34.89 (0.14, 79.77) | 11.78 (-3.99, 31.33) | 0.39 (0.27, 0.52)* |
| Belize | 0.04 (0.00, 0.09) | 0.11 (0.00, 0.27) | 195.72 (161.43, 233.05) | 5.51 (0.03, 12.67) | 5.57 (0.02, 12.88) | 1.20 (-5.12, 7.57) | 26.05 (0.10, 62.26) | 27.42 (0.11, 64.57) | 5.27 (-3.71, 14.93) | 0.19 (0.13, 0.25)* |
| Benin | 1.91 (0.01, 4.30) | 5.18 (0.02, 11.50) | 171.80 (145.65, 198.99) | 10.49 (0.05, 22.34) | 10.04 (0.05, 21.47) | -4.23 (-9.38, 1.67) | 64.25 (0.27, 141.96) | 64.20 (0.27, 141.28) | -0.07 (-9.25, 10.09) | -0.00 (-0.02, 0.01) |
| Bermuda | 0.04 (0.00, 0.09) | 0.04 (0.00, 0.09) | -3.53 (-19.60, 15.72) | 11.01 (0.06, 23.33) | 10.94 (0.06, 23.30) | -0.65 (-7.83, 6.13) | 60.94 (0.29, 134.99) | 51.06 (0.25, 114.15) | -16.22 (-25.78, -5.69) | -0.61 (-0.64, -0.58)* |
| Bhutan | 0.10 (0.00, 0.25) | 0.16 (0.00, 0.39) | 57.43 (39.74, 77.89) | 3.83 (0.02, 9.20) | 3.70 (0.01, 8.98) | -3.39 (-10.24, 3.60) | 23.97 (0.09, 57.45) | 21.37 (0.08, 51.42) | -10.83 (-20.32, -1.11) | -0.39 (-0.43, -0.35)* |
| Bolivia (Plurinational State of) | 2.91 (0.01, 6.48) | 5.88 (0.03, 13.11) | 102.08 (82.32, 125.55) | 10.65 (0.05, 22.78) | 10.48 (0.05, 22.55) | -1.60 (-6.76, 3.86) | 60.07 (0.26, 130.70) | 53.35 (0.24, 118.76) | -11.19 (-19.04, -2.19) | -0.40 (-0.48, -0.33)* |
| Bosnia and Herzegovina | 1.45 (0.01, 3.38) | 1.03 (0.00, 2.42) | -29.21 (-41.63, -14.20) | 6.84 (0.03, 15.92) | 7.11 (0.03, 16.23) | 4.02 (-2.07, 10.61) | 30.39 (0.12, 70.70) | 23.82 (0.10, 56.29) | -21.60 (-30.87, -10.22) | -0.83 (-0.93, -0.72)* |
| Botswana | 0.65 (0.00, 1.45) | 1.54 (0.01, 3.33) | 136.84 (110.44, 165.94) | 12.54 (0.07, 25.81) | 12.14 (0.07, 25.21) | -3.25 (-8.51, 2.30) | 72.25 (0.33, 157.41) | 71.71 (0.36, 154.55) | -0.75 (-10.59, 9.28) | -0.03 (-0.06, 0.00) |
| Brazil | 47.03 (0.19, 112.94) | 75.63 (0.31, 176.19) | 60.81 (41.89, 79.60) | 5.59 (0.03, 13.17) | 5.22 (0.03, 12.33) | -6.60 (-9.92, -3.38) | 35.57 (0.15, 83.21) | 31.18 (0.12, 72.80) | -12.35 (-16.72, -7.85) | -0.49 (-0.57, -0.41)* |
| Brunei Darussalam | 0.03 (0.00, 0.07) | 0.07 (0.00, 0.15) | 116.83 (85.83, 153.06) | 6.72 (0.03, 15.05) | 6.89 (0.03, 15.41) | 2.52 (-3.99, 9.24) | 13.18 (0.06, 30.44) | 13.59 (0.05, 30.93) | 3.12 (-7.06, 13.96) | 0.10 (0.00, 0.20)* |
| Bulgaria | 3.62 (0.02, 8.12) | 2.81 (0.01, 6.38) | -22.51 (-34.21, -9.35) | 9.21 (0.04, 20.36) | 9.08 (0.04, 20.18) | -1.40 (-7.79, 5.46) | 35.36 (0.15, 79.18) | 29.19 (0.11, 65.85) | -17.44 (-28.47, -4.94) | -0.67 (-0.72, -0.63)* |
| Burkina Faso | 2.67 (0.01, 6.29) | 6.40 (0.03, 15.22) | 139.67 (114.43, 168.11) | 7.04 (0.03, 15.82) | 7.10 (0.03, 16.15) | 0.86 (-5.83, 8.18) | 44.57 (0.18, 101.15) | 44.25 (0.18, 100.22) | -0.71 (-10.48, 10.78) | -0.00 (-0.10, 0.10) |
| Burundi | 2.96 (0.01, 6.56) | 4.88 (0.02, 11.06) | 64.76 (47.87, 82.89) | 9.85 (0.05, 21.08) | 9.15 (0.04, 19.40) | -7.15 (-13.19, -0.98) | 83.11 (0.34, 183.98) | 61.25 (0.25, 135.33) | -26.31 (-34.07, -17.88) | -1.06 (-1.10, -1.02)* |
| Cabo Verde | 0.16 (0.00, 0.37) | 0.35 (0.00, 0.78) | 116.89 (91.27, 145.58) | 9.91 (0.05, 21.04) | 9.13 (0.04, 19.43) | -7.80 (-14.40, -1.00) | 66.65 (0.28, 145.20) | 66.59 (0.28, 146.29) | -0.09 (-10.14, 11.02) | -0.01 (-0.03, 0.01) |
| Cambodia | 1.73 (0.01, 4.34) | 2.55 (0.01, 6.24) | 47.28 (21.05, 79.03) | 5.84 (0.03, 13.75) | 4.98 (0.02, 11.61) | -14.69 (-26.27, -2.17) | 21.91 (0.09, 52.75) | 15.88 (0.06, 37.96) | -27.53 (-38.32, -14.91) | -1.10 (-1.17, -1.03)* |
| Cameroon | 5.80 (0.03, 12.38) | 17.43 (0.09, 37.51) | 200.43 (172.58, 233.07) | 13.16 (0.07, 26.40) | 12.76 (0.07, 25.65) | -3.01 (-8.50, 2.61) | 84.55 (0.41, 179.49) | 85.73 (0.43, 183.45) | 1.39 (-8.35, 11.41) | 0.05 (0.03, 0.07)* |
| Canada | 8.68 (0.03, 20.62) | 8.80 (0.03, 21.31) | 1.36 (-15.88, 21.76) | 6.54 (0.03, 14.73) | 5.48 (0.03, 13.11) | -16.22 (-30.92, 0.37) | 28.68 (0.11, 67.81) | 23.90 (0.10, 59.40) | -16.64 (-31.38, 1.29) | -0.64 (-0.73, -0.56)* |
| Central African Republic | 1.82 (0.01, 4.13) | 3.59 (0.02, 8.05) | 97.23 (80.66, 117.82) | 9.10 (0.04, 19.69) | 9.12 (0.05, 19.81) | 0.25 (-4.95, 5.94) | 87.21 (0.36, 195.80) | 86.24 (0.38, 188.37) | -1.11 (-9.12, 7.84) | -0.05 (-0.11, 0.01) |
| Chad | 2.74 (0.01, 6.29) | 6.55 (0.03, 15.10) | 138.87 (106.49, 173.11) | 9.52 (0.05, 20.50) | 8.73 (0.04, 19.05) | -8.29 (-17.19, 2.05) | 72.24 (0.31, 163.27) | 68.31 (0.29, 152.06) | -5.44 (-16.86, 8.24) | -0.17 (-0.39, 0.05) |
| Chile | 11.07 (0.05, 24.16) | 13.73 (0.07, 30.34) | 24.01 (6.24, 44.81) | 11.29 (0.06, 24.25) | 11.12 (0.06, 23.36) | -1.49 (-7.81, 4.77) | 85.19 (0.40, 185.63) | 67.46 (0.33, 149.71) | -20.81 (-31.37, -7.55) | -0.81 (-0.87, -0.76)* |
| China | 309.35 (1.24, 715.83) | 380.09 (1.48, 878.53) | 22.87 (-2.63, 52.91) | 7.68 (0.04, 17.34) | 7.18 (0.03, 16.31) | -6.60 (-15.58, 3.25) | 25.72 (0.10, 58.19) | 20.43 (0.08, 46.74) | -20.57 (-28.77, -10.92) | -0.80 (-1.18, -0.42)* |
| Colombia | 8.87 (0.04, 19.90) | 12.96 (0.06, 28.53) | 46.11 (19.00, 77.90) | 9.56 (0.05, 20.30) | 8.76 (0.04, 18.54) | -8.38 (-18.06, 1.52) | 31.10 (0.15, 69.14) | 24.99 (0.11, 55.41) | -19.65 (-32.00, -6.42) | -0.76 (-0.86, -0.66)* |
| Comoros | 0.06 (0.00, 0.15) | 0.11 (0.00, 0.27) | 93.71 (73.22, 118.19) | 3.12 (0.01, 7.71) | 3.15 (0.01, 7.78) | 1.10 (-5.65, 8.72) | 19.03 (0.07, 47.20) | 18.30 (0.07, 44.33) | -3.80 (-13.60, 6.49) | -0.15 (-0.19, -0.10)* |
| Congo | 1.88 (0.01, 4.09) | 4.36 (0.02, 9.45) | 131.52 (106.66, 158.63) | 11.01 (0.06, 22.72) | 11.05 (0.06, 22.65) | 0.31 (-5.58, 6.38) | 105.15 (0.48, 227.55) | 95.85 (0.45, 208.03) | -8.84 (-17.16, -0.12) | -0.32 (-0.35, -0.29)* |
| Cook Islands | 0.01 (0.00, 0.01) | 0.01 (0.00, 0.02) | 19.54 (4.38, 38.17) | 9.67 (0.05, 20.19) | 10.53 (0.06, 22.05) | 8.83 (3.26, 14.87) | 35.08 (0.17, 77.78) | 38.31 (0.20, 84.12) | 9.21 (0.79, 18.25) | 0.30 (0.24, 0.35)* |
| Costa Rica | 1.08 (0.00, 2.45) | 2.36 (0.01, 5.29) | 117.70 (91.95, 148.02) | 9.13 (0.05, 19.50) | 9.35 (0.05, 20.14) | 2.35 (-3.86, 8.81) | 41.75 (0.18, 94.12) | 45.19 (0.20, 101.44) | 8.23 (-1.26, 17.65) | 0.28 (0.25, 0.30)* |
| Croatia | 2.00 (0.01, 4.63) | 1.56 (0.01, 3.67) | -22.12 (-32.89, -7.78) | 7.22 (0.03, 16.30) | 7.01 (0.03, 16.06) | -2.79 (-8.78, 4.45) | 34.64 (0.14, 81.29) | 27.02 (0.12, 62.62) | -21.98 (-31.02, -9.55) | -0.93 (-1.24, -0.61)* |
| Cuba | 8.39 (0.03, 18.92) | 6.29 (0.03, 14.18) | -24.98 (-40.63, -6.17) | 8.71 (0.04, 19.52) | 7.54 (0.04, 16.62) | -13.44 (-20.48, -6.43) | 71.72 (0.28, 159.62) | 44.77 (0.20, 100.53) | -37.58 (-46.71, -28.22) | -1.64 (-1.71, -1.56)* |
| Cyprus | 0.14 (0.00, 0.34) | 0.26 (0.00, 0.64) | 93.37 (71.06, 115.23) | 4.08 (0.02, 9.82) | 4.13 (0.02, 9.99) | 1.38 (-4.47, 6.85) | 16.76 (0.07, 41.67) | 16.98 (0.07, 41.32) | 1.32 (-7.70, 10.64) | 0.03 (-0.07, 0.13) |
| Czechia | 4.75 (0.02, 10.77) | 4.58 (0.02, 10.44) | -3.50 (-18.29, 12.40) | 9.18 (0.04, 19.95) | 9.10 (0.05, 20.10) | -0.91 (-10.59, 10.60) | 40.57 (0.16, 91.64) | 32.68 (0.15, 73.50) | -19.44 (-31.27, -6.64) | -0.78 (-0.89, -0.66)* |
| Côte d’Ivoire | 4.23 (0.02, 9.29) | 10.50 (0.05, 23.34) | 148.40 (125.56, 172.55) | 10.23 (0.05, 21.22) | 10.45 (0.06, 21.75) | 2.12 (-4.32, 9.23) | 53.74 (0.25, 116.07) | 55.74 (0.26, 120.60) | 3.72 (-5.99, 13.98) | 0.12 (0.07, 0.16)* |
| Democratic People's Republic of Korea | 4.59 (0.02, 10.78) | 5.72 (0.02, 13.14) | 24.54 (9.40, 41.60) | 7.48 (0.04, 16.99) | 6.96 (0.03, 15.85) | -6.93 (-12.18, -1.04) | 22.67 (0.09, 52.05) | 18.26 (0.07, 41.44) | -19.45 (-27.73, -10.26) | -0.75 (-0.78, -0.72)* |
| Democratic Republic of the Congo | 27.11 (0.13, 59.41) | 63.68 (0.29, 137.37) | 134.88 (105.22, 173.76) | 11.30 (0.06, 23.40) | 11.22 (0.06, 22.99) | -0.78 (-10.08, 10.28) | 98.21 (0.47, 212.40) | 95.13 (0.43, 203.92) | -3.14 (-13.86, 10.17) | -0.10 (-0.26, 0.05) |
| Denmark | 3.43 (0.01, 7.62) | 2.63 (0.01, 6.01) | -23.48 (-34.72, -11.26) | 8.56 (0.04, 19.22) | 8.12 (0.04, 18.02) | -5.16 (-12.56, 1.97) | 57.90 (0.23, 129.93) | 39.56 (0.17, 89.43) | -31.68 (-40.97, -20.63) | -1.32 (-1.43, -1.21)* |
| Djibouti | 0.17 (0.00, 0.37) | 0.56 (0.00, 1.24) | 234.40 (194.55, 275.29) | 8.90 (0.04, 18.95) | 8.71 (0.04, 18.65) | -2.15 (-8.12, 4.70) | 55.46 (0.24, 122.07) | 54.68 (0.22, 118.40) | -1.42 (-10.24, 8.06) | -0.06 (-0.08, -0.04)* |
| Dominica | 0.02 (0.00, 0.06) | 0.03 (0.00, 0.06) | 8.33 (-2.59, 20.10) | 8.35 (0.04, 18.55) | 7.84 (0.04, 17.07) | -6.02 (-11.37, 0.19) | 36.87 (0.16, 85.41) | 34.89 (0.15, 78.84) | -5.37 (-13.43, 3.37) | -0.19 (-0.29, -0.09)* |
| Dominican Republic | 2.78 (0.01, 6.59) | 4.75 (0.02, 11.04) | 71.15 (51.82, 93.85) | 7.65 (0.04, 17.14) | 7.20 (0.03, 16.23) | -5.93 (-11.58, -0.89) | 45.12 (0.19, 105.40) | 43.14 (0.18, 100.90) | -4.38 (-12.71, 4.34) | -0.17 (-0.23, -0.10)* |
| Ecuador | 2.47 (0.01, 6.00) | 5.09 (0.02, 11.89) | 105.92 (79.07, 136.85) | 6.04 (0.03, 13.97) | 5.89 (0.03, 13.87) | -2.47 (-8.53, 4.15) | 29.47 (0.12, 70.01) | 29.23 (0.12, 67.81) | -0.81 (-10.49, 10.62) | -0.03 (-0.14, 0.07) |
| Egypt | 21.95 (0.09, 50.89) | 43.47 (0.18, 100.44) | 98.01 (65.82, 130.39) | 8.73 (0.04, 19.22) | 8.02 (0.04, 17.77) | -8.09 (-20.03, 4.60) | 48.37 (0.19, 109.98) | 47.01 (0.20, 107.03) | -2.82 (-17.39, 11.31) | -0.13 (-0.20, -0.06)* |
| El Salvador | 2.00 (0.01, 4.84) | 2.62 (0.01, 6.09) | 30.79 (13.36, 49.51) | 7.89 (0.04, 17.95) | 7.97 (0.04, 17.95) | 1.03 (-5.42, 7.91) | 46.14 (0.19, 106.94) | 41.54 (0.17, 95.43) | -9.97 (-19.70, 0.33) | -0.36 (-0.42, -0.31)* |
| Equatorial Guinea | 0.34 (0.00, 0.73) | 1.07 (0.01, 2.39) | 214.57 (184.49, 246.03) | 11.52 (0.06, 23.70) | 10.89 (0.06, 22.77) | -5.50 (-11.81, 1.17) | 111.51 (0.51, 238.67) | 99.10 (0.46, 216.76) | -11.13 (-19.51, -2.39) | -0.41 (-0.43, -0.39)* |
| Eritrea | 1.32 (0.01, 2.89) | 3.15 (0.01, 7.06) | 138.84 (115.12, 165.62) | 9.97 (0.05, 21.39) | 9.56 (0.04, 20.69) | -4.12 (-9.74, 1.86) | 72.98 (0.30, 158.86) | 66.60 (0.29, 146.49) | -8.73 (-17.73, 0.62) | -0.32 (-0.33, -0.30)* |
| Estonia | 0.86 (0.00, 1.99) | 0.58 (0.00, 1.39) | -32.28 (-41.73, -20.62) | 6.54 (0.03, 14.97) | 6.20 (0.03, 14.38) | -5.19 (-11.87, 2.34) | 47.87 (0.19, 110.84) | 32.73 (0.13, 75.57) | -31.63 (-40.16, -20.97) | -1.30 (-1.47, -1.14)* |
| Eswatini | 0.29 (0.00, 0.66) | 0.56 (0.00, 1.27) | 90.09 (70.47, 113.99) | 9.96 (0.05, 21.40) | 9.73 (0.05, 20.53) | -2.32 (-8.39, 4.03) | 57.01 (0.25, 125.53) | 60.40 (0.27, 134.80) | 5.94 (-3.79, 17.94) | 0.20 (0.12, 0.29)* |
| Ethiopia | 25.32 (0.13, 54.75) | 47.71 (0.24, 105.35) | 88.42 (74.21, 101.32) | 10.31 (0.06, 21.05) | 10.23 (0.06, 20.67) | -0.80 (-7.11, 6.47) | 77.03 (0.39, 165.80) | 68.51 (0.33, 146.69) | -11.06 (-17.50, -3.79) | -0.40 (-0.45, -0.35)* |
| Fiji | 0.21 (0.00, 0.48) | 0.28 (0.00, 0.63) | 32.79 (17.05, 52.41) | 10.08 (0.06, 21.12) | 9.88 (0.05, 20.99) | -1.97 (-8.37, 4.72) | 31.51 (0.15, 69.17) | 31.02 (0.15, 69.13) | -1.55 (-10.98, 9.00) | -0.06 (-0.08, -0.03)* |
| Finland | 3.50 (0.02, 7.86) | 2.93 (0.01, 6.62) | -16.22 (-29.68, -2.81) | 8.11 (0.04, 17.56) | 8.27 (0.04, 18.04) | 2.06 (-6.61, 10.20) | 62.18 (0.27, 142.99) | 49.52 (0.21, 113.33) | -20.36 (-31.05, -8.85) | -0.81 (-1.04, -0.57)* |
| France | 28.33 (0.10, 65.52) | 26.39 (0.11, 61.21) | -6.86 (-20.46, 8.52) | 6.46 (0.03, 14.93) | 6.47 (0.03, 14.79) | 0.25 (-7.16, 8.49) | 44.42 (0.16, 102.80) | 36.40 (0.16, 85.14) | -18.06 (-30.33, -3.66) | -0.66 (-0.78, -0.54)* |
| Gabon | 0.80 (0.00, 1.75) | 1.65 (0.01, 3.55) | 105.31 (83.30, 129.59) | 11.46 (0.07, 23.41) | 11.90 (0.07, 23.84) | 3.83 (-2.75, 10.59) | 104.77 (0.58, 220.34) | 103.53 (0.55, 218.92) | -1.19 (-10.72, 8.45) | -0.04 (-0.09, 0.02) |
| Gambia | 0.50 (0.00, 1.22) | 1.24 (0.01, 2.99) | 146.94 (123.64, 172.62) | 9.37 (0.04, 20.93) | 9.43 (0.04, 21.39) | 0.67 (-3.76, 5.56) | 81.48 (0.34, 186.12) | 81.11 (0.33, 187.83) | -0.45 (-9.15, 10.00) | -0.02 (-0.06, 0.01) |
| Georgia | 0.43 (0.00, 1.10) | 0.32 (0.00, 0.83) | -26.99 (-35.46, -17.58) | 1.61 (0.01, 4.08) | 1.49 (0.01, 3.81) | -7.21 (-13.04, -1.54) | 7.17 (0.03, 18.31) | 6.70 (0.02, 17.41) | -6.57 (-15.68, 3.21) | -0.23 (-0.33, -0.13)* |
| Germany | 31.67 (0.13, 71.16) | 37.15 (0.15, 83.10) | 17.28 (1.93, 34.42) | 7.62 (0.04, 16.68) | 7.86 (0.04, 17.26) | 3.12 (-4.31, 11.22) | 33.42 (0.14, 75.42) | 37.87 (0.15, 85.99) | 13.32 (-1.42, 30.67) | 0.41 (0.29, 0.53)* |
| Ghana | 7.98 (0.04, 16.85) | 20.00 (0.10, 44.20) | 150.66 (131.54, 173.93) | 12.70 (0.07, 25.88) | 12.90 (0.08, 25.75) | 1.58 (-3.13, 6.85) | 78.68 (0.39, 169.80) | 80.07 (0.41, 173.75) | 1.77 (-5.72, 10.10) | 0.06 (0.03, 0.10)* |
| Greece | 5.30 (0.02, 12.74) | 5.44 (0.02, 13.00) | 2.68 (-8.27, 15.56) | 5.69 (0.03, 13.08) | 5.63 (0.03, 12.95) | -1.04 (-6.22, 5.19) | 45.81 (0.17, 110.64) | 45.93 (0.18, 110.82) | 0.27 (-8.29, 10.77) | -0.07 (-0.41, 0.27) |
| Greenland | 0.05 (0.00, 0.12) | 0.04 (0.00, 0.10) | -20.51 (-31.59, -7.94) | 6.79 (0.03, 15.31) | 6.89 (0.03, 15.67) | 1.52 (-5.54, 9.35) | 74.44 (0.28, 175.47) | 67.56 (0.27, 161.12) | -9.25 (-19.34, 0.54) | -0.32 (-0.40, -0.25)* |
| Grenada | 0.03 (0.00, 0.06) | 0.04 (0.00, 0.09) | 49.87 (34.02, 68.04) | 7.92 (0.04, 17.45) | 7.46 (0.04, 16.47) | -5.80 (-11.34, -0.34) | 37.74 (0.15, 86.75) | 35.56 (0.15, 80.91) | -5.78 (-13.58, 1.94) | -0.21 (-0.25, -0.16)* |
| Guam | 0.04 (0.00, 0.09) | 0.06 (0.00, 0.12) | 31.51 (13.38, 53.65) | 8.24 (0.04, 17.44) | 8.34 (0.04, 17.55) | 1.22 (-7.28, 9.69) | 30.92 (0.14, 67.80) | 31.56 (0.15, 68.74) | 2.06 (-8.60, 13.12) | 0.07 (0.03, 0.11)* |
| Guatemala | 1.54 (0.01, 3.69) | 4.70 (0.02, 11.32) | 205.86 (159.19, 265.57) | 4.59 (0.02, 11.13) | 5.16 (0.02, 12.01) | 12.48 (-1.30, 31.26) | 27.29 (0.10, 65.35) | 29.55 (0.11, 72.51) | 8.27 (-7.27, 28.89) | 0.28 (0.09, 0.47)* |
| Guinea | 2.75 (0.01, 6.10) | 5.52 (0.02, 12.29) | 100.40 (81.33, 120.62) | 10.80 (0.05, 23.09) | 10.50 (0.05, 22.33) | -2.71 (-7.76, 2.85) | 64.01 (0.28, 140.83) | 65.36 (0.28, 142.59) | 2.09 (-7.31, 12.64) | 0.07 (0.05, 0.09)* |
| Guinea-Bissau | 0.43 (0.00, 0.96) | 0.90 (0.00, 2.08) | 111.50 (92.84, 132.40) | 10.83 (0.05, 23.18) | 10.81 (0.05, 23.21) | -0.13 (-5.53, 5.60) | 66.78 (0.29, 147.96) | 69.96 (0.30, 155.19) | 4.76 (-5.10, 15.27) | 0.17 (0.14, 0.19)* |
| Guyana | 0.42 (0.00, 1.00) | 0.52 (0.00, 1.22) | 24.13 (7.75, 41.63) | 8.04 (0.04, 18.22) | 7.89 (0.04, 17.36) | -1.78 (-8.47, 4.80) | 60.60 (0.26, 141.78) | 65.01 (0.26, 152.73) | 7.27 (-5.87, 19.17) | 0.25 (0.20, 0.30)* |
| Haiti | 2.40 (0.01, 5.69) | 5.13 (0.02, 12.02) | 113.57 (93.18, 136.41) | 8.06 (0.04, 18.37) | 7.98 (0.04, 18.03) | -0.96 (-5.97, 4.26) | 46.16 (0.20, 108.89) | 44.13 (0.18, 101.24) | -4.39 (-12.93, 4.94) | -0.20 (-0.34, -0.05)* |
| Honduras | 0.85 (0.00, 2.10) | 2.42 (0.01, 5.91) | 185.39 (158.18, 218.17) | 5.97 (0.03, 13.59) | 6.06 (0.03, 14.16) | 1.56 (-3.79, 7.76) | 26.46 (0.11, 63.52) | 28.31 (0.11, 67.11) | 6.98 (-2.87, 17.71) | 0.25 (0.07, 0.43)* |
| Hungary | 2.94 (0.01, 6.93) | 2.31 (0.01, 5.44) | -21.44 (-34.01, -8.48) | 5.20 (0.02, 12.03) | 5.04 (0.02, 11.96) | -2.94 (-11.10, 4.76) | 24.36 (0.09, 57.34) | 17.52 (0.06, 40.93) | -28.05 (-38.12, -16.68) | -1.12 (-1.27, -0.97)* |
| Iceland | 0.03 (0.00, 0.08) | 0.04 (0.00, 0.09) | 18.33 (0.91, 38.87) | 2.68 (0.01, 6.69) | 2.56 (0.01, 6.20) | -4.59 (-11.56, 2.81) | 11.59 (0.04, 29.38) | 9.61 (0.04, 23.78) | -17.09 (-27.19, -5.83) | -0.65 (-0.78, -0.52)* |
| India | 354.02 (1.54, 795.50) | 564.72 (2.38, 1245.07) | 59.52 (44.71, 77.69) | 8.82 (0.04, 19.18) | 8.22 (0.04, 17.97) | -6.83 (-12.90, -0.06) | 50.50 (0.22, 110.24) | 41.19 (0.17, 90.67) | -18.44 (-24.68, -11.82) | -0.69 (-0.83, -0.55)* |
| Indonesia | 24.95 (0.11, 59.14) | 41.13 (0.18, 94.91) | 64.81 (50.43, 79.99) | 6.97 (0.03, 15.62) | 6.84 (0.03, 15.35) | -1.92 (-3.89, -0.08) | 15.23 (0.06, 34.81) | 14.85 (0.06, 34.25) | -2.50 (-5.33, 0.24) | -0.09 (-0.12, -0.06)* |
| Iran (Islamic Republic of) | 43.86 (0.24, 92.29) | 94.70 (0.52, 202.47) | 115.91 (90.72, 142.68) | 13.12 (0.08, 26.47) | 12.95 (0.08, 26.13) | -1.25 (-3.22, 1.00) | 96.99 (0.52, 207.53) | 101.10 (0.54, 218.12) | 4.24 (1.42, 7.31) | 0.14 (0.06, 0.22)* |
| Iraq | 4.71 (0.02, 11.26) | 14.42 (0.07, 34.24) | 206.06 (175.15, 240.68) | 6.44 (0.03, 14.85) | 6.30 (0.03, 14.46) | -2.03 (-7.98, 4.82) | 37.10 (0.16, 88.39) | 36.62 (0.16, 84.59) | -1.29 (-10.97, 9.69) | -0.06 (-0.18, 0.07) |
| Ireland | 1.21 (0.00, 2.81) | 1.69 (0.01, 4.01) | 39.33 (17.61, 64.83) | 5.71 (0.03, 12.92) | 5.08 (0.02, 11.82) | -11.12 (-23.32, 1.93) | 34.23 (0.13, 78.95) | 32.27 (0.13, 77.76) | -5.74 (-21.53, 12.84) | -0.23 (-0.40, -0.07)* |
| Israel | 2.78 (0.01, 6.21) | 4.91 (0.02, 11.03) | 76.98 (57.23, 100.28) | 8.78 (0.04, 19.45) | 8.49 (0.04, 18.48) | -3.27 (-9.40, 3.87) | 59.21 (0.27, 131.52) | 52.89 (0.22, 119.68) | -10.68 (-20.54, 1.00) | -0.42 (-0.49, -0.35)* |
| Italy | 37.40 (0.16, 84.70) | 38.48 (0.16, 85.29) | 2.90 (-3.97, 9.13) | 10.27 (0.05, 22.00) | 10.37 (0.05, 22.14) | 0.95 (-0.77, 2.72) | 56.27 (0.25, 127.53) | 53.52 (0.24, 120.58) | -4.89 (-7.46, -2.31) | -0.24 (-0.44, -0.05)* |
| Jamaica | 0.76 (0.00, 1.81) | 1.11 (0.00, 2.58) | 44.96 (29.43, 61.79) | 8.43 (0.04, 18.61) | 8.13 (0.04, 17.87) | -3.58 (-8.71, 1.61) | 36.71 (0.15, 86.43) | 35.84 (0.14, 84.05) | -2.37 (-9.81, 5.90) | -0.09 (-0.22, 0.04) |
| Japan | 42.66 (0.19, 93.04) | 47.61 (0.21, 103.06) | 11.61 (4.26, 20.44) | 10.06 (0.05, 21.48) | 9.94 (0.05, 21.10) | -1.20 (-3.68, 1.27) | 29.55 (0.13, 64.33) | 31.47 (0.14, 68.68) | 6.52 (2.99, 10.53) | 0.23 (0.06, 0.39)* |
| Jordan | 1.45 (0.01, 3.43) | 4.98 (0.02, 11.76) | 243.54 (200.69, 291.39) | 7.56 (0.04, 16.99) | 7.17 (0.04, 16.19) | -5.21 (-11.90, 1.90) | 54.12 (0.24, 124.78) | 45.42 (0.20, 106.98) | -16.09 (-25.84, -5.43) | -0.63 (-0.72, -0.54)* |
| Kazakhstan | 3.64 (0.01, 8.56) | 4.42 (0.02, 10.48) | 21.45 (7.03, 38.42) | 5.03 (0.02, 11.82) | 4.87 (0.02, 11.48) | -3.12 (-10.41, 4.01) | 24.36 (0.10, 56.71) | 23.14 (0.09, 54.83) | -4.99 (-16.34, 8.61) | -0.17 (-0.31, -0.03)* |
| Kenya | 10.74 (0.05, 22.96) | 26.01 (0.12, 56.43) | 142.17 (129.63, 154.38) | 10.60 (0.06, 22.08) | 10.53 (0.06, 21.81) | -0.66 (-4.20, 3.06) | 78.04 (0.35, 166.70) | 71.63 (0.33, 154.56) | -8.22 (-11.93, -4.32) | -0.29 (-0.33, -0.26)* |
| Kiribati | 0.02 (0.00, 0.05) | 0.04 (0.00, 0.08) | 52.59 (34.41, 72.92) | 10.84 (0.06, 22.48) | 10.84 (0.06, 22.31) | -0.04 (-7.83, 7.75) | 38.53 (0.20, 83.48) | 34.81 (0.18, 74.44) | -9.67 (-19.58, 1.34) | -0.36 (-0.41, -0.31)* |
| Kuwait | 0.86 (0.00, 1.93) | 3.05 (0.01, 6.73) | 255.79 (203.79, 317.97) | 8.19 (0.04, 17.51) | 9.11 (0.05, 19.13) | 11.17 (3.95, 19.36) | 48.42 (0.22, 106.15) | 55.99 (0.25, 123.18) | 15.63 (5.97, 25.91) | 0.50 (0.40, 0.60)* |
| Kyrgyzstan | 1.39 (0.01, 3.13) | 2.03 (0.01, 4.70) | 46.00 (28.07, 66.55) | 7.48 (0.03, 16.67) | 7.34 (0.03, 16.59) | -1.89 (-7.88, 4.71) | 38.87 (0.16, 87.74) | 34.17 (0.14, 77.78) | -12.08 (-21.69, -0.11) | -0.45 (-0.49, -0.41)* |
| Lao People's Democratic Republic | 0.55 (0.00, 1.38) | 0.90 (0.00, 2.07) | 65.13 (47.13, 86.95) | 5.14 (0.02, 12.06) | 4.52 (0.02, 10.37) | -11.95 (-19.56, -4.14) | 16.40 (0.07, 38.74) | 12.71 (0.05, 29.03) | -22.50 (-30.92, -13.15) | -0.88 (-0.94, -0.81)* |
| Latvia | 1.96 (0.01, 4.37) | 1.28 (0.01, 2.86) | -34.83 (-43.65, -24.73) | 9.41 (0.04, 20.60) | 9.06 (0.04, 19.61) | -3.71 (-10.00, 3.72) | 62.55 (0.25, 140.27) | 48.95 (0.19, 107.57) | -21.74 (-30.96, -9.30) | -0.84 (-0.89, -0.80)* |
| Lebanon | 1.88 (0.01, 4.12) | 4.10 (0.02, 9.24) | 117.71 (87.72, 147.88) | 9.90 (0.05, 21.03) | 10.37 (0.05, 21.95) | 4.78 (-2.33, 11.59) | 67.75 (0.30, 147.52) | 75.72 (0.33, 169.44) | 11.77 (-1.87, 25.16) | 0.39 (0.32, 0.47)* |
| Lesotho | 1.31 (0.01, 2.95) | 1.67 (0.01, 3.66) | 27.62 (11.16, 44.19) | 11.56 (0.06, 24.73) | 10.76 (0.06, 22.91) | -6.97 (-12.46, -1.11) | 97.71 (0.41, 216.32) | 91.14 (0.42, 195.66) | -6.72 (-17.18, 4.73) | -0.26 (-0.36, -0.17)* |
| Liberia | 1.34 (0.01, 2.93) | 3.41 (0.02, 7.43) | 155.32 (129.78, 185.24) | 14.83 (0.09, 30.16) | 14.63 (0.09, 29.55) | -1.35 (-5.58, 3.10) | 97.25 (0.51, 208.79) | 95.97 (0.50, 203.92) | -1.32 (-10.55, 9.25) | -0.01 (-0.09, 0.07) |
| Libya | 1.86 (0.01, 4.18) | 4.85 (0.02, 10.79) | 160.43 (126.97, 200.13) | 9.07 (0.05, 18.90) | 9.43 (0.05, 20.06) | 3.91 (-1.91, 10.44) | 59.03 (0.27, 131.23) | 64.22 (0.30, 141.16) | 8.80 (0.16, 18.10) | 0.29 (0.27, 0.31)* |
| Lithuania | 1.91 (0.01, 4.40) | 1.54 (0.01, 3.54) | -19.28 (-31.23, -7.30) | 7.23 (0.03, 16.31) | 6.95 (0.03, 15.52) | -3.83 (-10.44, 3.07) | 46.46 (0.19, 107.09) | 41.07 (0.18, 93.39) | -11.59 (-22.12, 0.39) | -0.42 (-0.47, -0.37)* |
| Luxembourg | 0.14 (0.00, 0.33) | 0.17 (0.00, 0.38) | 21.88 (7.31, 39.05) | 5.46 (0.03, 12.57) | 5.30 (0.03, 12.49) | -3.02 (-9.73, 4.10) | 30.63 (0.14, 75.45) | 22.87 (0.09, 53.00) | -25.33 (-33.82, -15.47) | -1.00 (-1.05, -0.96)* |
| Madagascar | 5.12 (0.02, 11.48) | 11.91 (0.05, 26.89) | 132.58 (108.40, 157.56) | 9.25 (0.04, 19.85) | 9.19 (0.04, 19.35) | -0.69 (-6.88, 5.92) | 65.09 (0.26, 143.83) | 62.47 (0.26, 138.95) | -4.02 (-13.50, 6.03) | -0.14 (-0.16, -0.12)* |
| Malawi | 3.83 (0.02, 8.44) | 6.89 (0.03, 15.29) | 80.00 (56.27, 107.88) | 10.71 (0.05, 22.86) | 10.38 (0.05, 21.90) | -3.05 (-12.59, 7.98) | 61.58 (0.26, 135.28) | 56.16 (0.24, 123.74) | -8.80 (-20.37, 4.73) | -0.30 (-0.39, -0.21)* |
| Malaysia | 3.25 (0.01, 7.71) | 7.97 (0.03, 18.87) | 144.98 (115.16, 180.88) | 5.58 (0.03, 12.63) | 5.04 (0.02, 11.37) | -9.73 (-17.60, -0.79) | 21.80 (0.09, 50.24) | 23.95 (0.09, 56.74) | 9.82 (-3.58, 25.81) | 0.31 (0.23, 0.38)* |
| Maldives | 0.05 (0.00, 0.12) | 0.09 (0.00, 0.21) | 77.80 (45.59, 111.91) | 6.40 (0.03, 14.74) | 5.03 (0.02, 11.53) | -21.40 (-28.90, -12.65) | 28.94 (0.11, 67.75) | 16.48 (0.07, 38.21) | -43.05 (-49.80, -35.20) | -1.93 (-1.99, -1.88)* |
| Mali | 2.43 (0.01, 5.60) | 5.51 (0.02, 13.03) | 126.37 (99.07, 154.45) | 9.19 (0.04, 20.12) | 8.85 (0.04, 19.57) | -3.70 (-9.36, 2.95) | 42.29 (0.17, 95.65) | 39.86 (0.17, 91.17) | -5.76 (-16.18, 5.45) | -0.18 (-0.29, -0.07)* |
| Malta | 0.08 (0.00, 0.19) | 0.09 (0.00, 0.23) | 20.11 (7.24, 35.49) | 4.51 (0.02, 10.81) | 4.46 (0.02, 10.54) | -1.19 (-7.19, 4.79) | 19.25 (0.08, 46.74) | 18.50 (0.07, 45.18) | -3.86 (-12.00, 5.35) | -0.15 (-0.22, -0.09)* |
| Marshall Islands | 0.01 (0.00, 0.02) | 0.01 (0.00, 0.03) | 56.67 (36.24, 82.34) | 6.84 (0.03, 15.25) | 6.86 (0.03, 15.40) | 0.27 (-6.80, 8.62) | 22.43 (0.09, 50.62) | 21.17 (0.09, 48.45) | -5.62 (-14.12, 5.07) | -0.20 (-0.22, -0.18)* |
| Mauritania | 0.62 (0.00, 1.37) | 1.26 (0.01, 2.83) | 101.98 (82.23, 123.54) | 9.02 (0.05, 19.15) | 8.92 (0.05, 19.14) | -1.07 (-7.88, 6.20) | 44.88 (0.21, 96.63) | 42.49 (0.20, 93.99) | -5.33 (-14.70, 4.53) | -0.19 (-0.22, -0.16)* |
| Mauritius | 0.44 (0.00, 1.07) | 0.49 (0.00, 1.13) | 11.84 (-6.76, 35.72) | 6.82 (0.03, 15.76) | 6.57 (0.03, 15.07) | -3.62 (-10.31, 3.43) | 39.83 (0.15, 92.58) | 32.86 (0.13, 76.11) | -17.50 (-26.97, -7.09) | -0.66 (-0.75, -0.58)* |
| Mexico | 28.13 (0.12, 63.04) | 72.22 (0.32, 160.65) | 156.73 (136.57, 176.62) | 9.49 (0.05, 20.47) | 10.17 (0.05, 21.79) | 7.21 (3.61, 10.56) | 41.63 (0.18, 92.51) | 55.16 (0.24, 122.46) | 32.50 (26.69, 38.64) | 1.10 (0.57, 1.62)* |
| Micronesia (Federated States of) | 0.03 (0.00, 0.06) | 0.03 (0.00, 0.06) | 15.20 (0.92, 33.00) | 9.37 (0.05, 19.54) | 9.38 (0.05, 19.75) | 0.14 (-7.16, 8.67) | 31.69 (0.15, 69.98) | 29.38 (0.14, 65.67) | -7.30 (-17.24, 3.67) | -0.26 (-0.30, -0.23)* |
| Monaco | 0.01 (0.00, 0.03) | 0.02 (0.00, 0.04) | 14.80 (4.33, 26.47) | 5.95 (0.03, 13.61) | 5.88 (0.03, 13.47) | -1.07 (-5.41, 3.64) | 36.41 (0.16, 88.42) | 35.81 (0.16, 86.22) | -1.65 (-8.46, 5.54) | -0.06 (-0.08, -0.03)* |
| Mongolia | 0.44 (0.00, 1.11) | 0.92 (0.00, 2.21) | 108.41 (76.72, 142.38) | 4.72 (0.02, 11.21) | 4.67 (0.02, 11.18) | -1.10 (-6.88, 4.81) | 27.92 (0.11, 67.26) | 27.48 (0.10, 64.52) | -1.58 (-10.45, 8.91) | -0.06 (-0.08, -0.03)* |
| Montenegro | 0.17 (0.00, 0.38) | 0.19 (0.00, 0.44) | 13.25 (-1.44, 28.37) | 7.12 (0.03, 16.47) | 7.04 (0.03, 16.01) | -1.09 (-6.27, 4.13) | 25.47 (0.11, 59.19) | 24.50 (0.10, 57.23) | -3.80 (-13.71, 6.49) | -0.12 (-0.14, -0.11)* |
| Morocco | 17.78 (0.08, 38.88) | 30.79 (0.13, 68.19) | 73.11 (52.71, 96.58) | 10.14 (0.05, 21.54) | 9.92 (0.05, 21.05) | -2.20 (-8.11, 3.97) | 85.73 (0.40, 185.86) | 81.63 (0.35, 180.39) | -4.78 (-13.98, 5.05) | -0.16 (-0.18, -0.15)* |
| Mozambique | 5.42 (0.02, 12.38) | 11.77 (0.05, 26.82) | 117.33 (94.84, 140.33) | 8.99 (0.04, 19.62) | 8.81 (0.04, 19.38) | -1.94 (-8.32, 4.31) | 61.74 (0.24, 137.51) | 63.64 (0.24, 141.22) | 3.07 (-7.28, 14.33) | 0.09 (0.03, 0.15)* |
| Myanmar | 3.63 (0.02, 8.64) | 5.30 (0.02, 12.55) | 46.32 (22.18, 74.01) | 6.31 (0.03, 14.23) | 5.92 (0.03, 13.47) | -6.22 (-18.58, 7.08) | 10.30 (0.04, 23.65) | 9.50 (0.04, 22.47) | -7.74 (-21.64, 6.72) | -0.32 (-0.50, -0.15)* |
| Namibia | 0.41 (0.00, 0.91) | 0.79 (0.00, 1.79) | 93.54 (71.65, 119.10) | 8.48 (0.04, 18.76) | 8.45 (0.04, 18.57) | -0.34 (-7.52, 6.67) | 39.89 (0.16, 89.01) | 38.89 (0.16, 87.85) | -2.50 (-12.41, 8.20) | -0.10 (-0.20, 0.00) |
| Nauru | 0.00 (0.00, 0.01) | 0.00 (0.00, 0.01) | 18.89 (8.46, 29.76) | 9.70 (0.06, 20.19) | 10.22 (0.06, 21.48) | 5.39 (-0.63, 11.15) | 34.90 (0.17, 78.26) | 36.96 (0.19, 81.61) | 5.91 (-2.56, 14.28) | 0.19 (0.16, 0.21)* |
| Nepal | 8.64 (0.04, 19.41) | 19.87 (0.08, 43.57) | 130.04 (106.29, 156.93) | 8.69 (0.04, 18.78) | 9.46 (0.05, 20.46) | 8.82 (2.95, 15.30) | 60.91 (0.26, 137.57) | 71.29 (0.30, 155.75) | 17.04 (5.57, 29.63) | 0.58 (0.46, 0.70)* |
| Netherlands | 6.85 (0.03, 15.79) | 7.12 (0.03, 16.13) | 3.94 (-13.92, 24.58) | 7.50 (0.04, 16.93) | 7.23 (0.03, 15.92) | -3.51 (-15.45, 10.66) | 39.98 (0.15, 91.34) | 36.99 (0.15, 83.43) | -7.49 (-23.90, 10.71) | -0.26 (-0.34, -0.19)* |
| New Zealand | 2.00 (0.01, 4.34) | 2.56 (0.01, 5.62) | 28.15 (15.04, 42.14) | 10.69 (0.06, 22.32) | 10.53 (0.06, 22.13) | -1.50 (-7.66, 3.91) | 55.09 (0.28, 119.69) | 58.16 (0.31, 128.14) | 5.57 (-4.20, 16.49) | 0.18 (0.15, 0.22)* |
| Nicaragua | 0.97 (0.00, 2.29) | 2.21 (0.01, 5.27) | 126.96 (97.92, 157.18) | 7.29 (0.03, 16.42) | 7.18 (0.03, 16.58) | -1.63 (-7.82, 4.13) | 35.16 (0.14, 81.26) | 35.26 (0.14, 83.64) | 0.29 (-8.98, 10.06) | -0.01 (-0.12, 0.10) |
| Niger | 2.87 (0.01, 6.40) | 8.13 (0.03, 18.28) | 183.39 (155.54, 214.43) | 10.30 (0.05, 21.94) | 10.45 (0.05, 22.29) | 1.49 (-3.95, 7.95) | 59.53 (0.26, 133.03) | 62.34 (0.26, 137.86) | 4.72 (-5.23, 15.76) | 0.16 (0.14, 0.18)* |
| Nigeria | 28.65 (0.12, 66.15) | 60.83 (0.27, 137.83) | 112.33 (100.96, 126.82) | 7.57 (0.04, 16.63) | 8.50 (0.04, 18.47) | 12.22 (6.17, 19.01) | 45.54 (0.19, 103.16) | 43.22 (0.19, 96.80) | -5.09 (-10.32, 1.03) | -0.17 (-0.28, -0.06)* |
| Niue | 0.00 (0.00, 0.00) | 0.00 (0.00, 0.00) | -15.27 (-23.43, -6.01) | 10.02 (0.06, 20.92) | 10.04 (0.05, 20.65) | 0.14 (-5.33, 5.76) | 36.43 (0.18, 80.79) | 36.21 (0.18, 80.38) | -0.61 (-8.66, 8.52) | -0.02 (-0.04, 0.00) |
| North Macedonia | 0.48 (0.00, 1.13) | 0.55 (0.00, 1.28) | 16.17 (0.39, 35.58) | 7.11 (0.03, 16.21) | 6.87 (0.03, 15.77) | -3.37 (-8.74, 2.87) | 23.17 (0.09, 53.65) | 20.37 (0.08, 46.44) | -12.10 (-21.31, 0.12) | -0.44 (-0.50, -0.39)* |
| Northern Mariana Islands | 0.01 (0.00, 0.02) | 0.01 (0.00, 0.03) | 9.72 (-15.73, 42.90) | 8.06 (0.05, 17.04) | 8.31 (0.04, 17.46) | 3.07 (-6.04, 12.84) | 22.01 (0.11, 49.15) | 24.13 (0.11, 53.74) | 9.59 (-1.45, 22.31) | 0.29 (0.12, 0.47)* |
| Norway | 1.01 (0.00, 2.37) | 1.39 (0.01, 3.31) | 38.19 (27.25, 48.22) | 5.68 (0.03, 12.93) | 5.39 (0.03, 12.53) | -5.15 (-11.72, 0.45) | 21.94 (0.09, 52.67) | 23.98 (0.10, 58.98) | 9.29 (1.69, 16.13) | 0.35 (0.09, 0.62)* |
| Oman | 0.67 (0.00, 1.51) | 2.00 (0.01, 4.48) | 197.82 (169.77, 228.75) | 7.66 (0.04, 16.61) | 7.05 (0.04, 15.13) | -7.97 (-14.85, -1.05) | 45.75 (0.20, 104.06) | 42.46 (0.19, 94.63) | -7.19 (-13.98, 0.24) | -0.22 (-0.72, 0.28) |
| Pakistan | 57.25 (0.30, 123.19) | 128.01 (0.68, 275.13) | 123.60 (113.60, 133.79) | 12.47 (0.08, 25.09) | 13.13 (0.08, 26.56) | 5.30 (2.06, 8.43) | 73.26 (0.38, 159.10) | 73.83 (0.38, 158.07) | 0.78 (-3.56, 5.26) | 0.04 (-0.07, 0.16) |
| Palau | 0.01 (0.00, 0.01) | 0.01 (0.00, 0.02) | 25.94 (5.54, 52.40) | 9.75 (0.05, 20.26) | 8.85 (0.05, 18.73) | -9.28 (-15.00, -3.74) | 35.18 (0.17, 78.07) | 31.51 (0.16, 70.95) | -10.42 (-17.73, -2.75) | -0.39 (-0.53, -0.25)* |
| Palestine | 1.53 (0.01, 3.32) | 3.93 (0.02, 8.69) | 156.83 (117.61, 192.04) | 10.28 (0.06, 21.46) | 8.90 (0.05, 18.61) | -13.38 (-24.67, -3.89) | 110.75 (0.55, 242.88) | 94.14 (0.44, 214.80) | -15.00 (-26.84, -5.22) | -0.55 (-0.63, -0.47)* |
| Panama | 0.65 (0.00, 1.49) | 1.32 (0.01, 3.00) | 103.01 (80.75, 127.40) | 7.24 (0.04, 16.08) | 7.36 (0.03, 16.40) | 1.68 (-3.90, 7.57) | 31.35 (0.14, 70.81) | 31.36 (0.12, 70.86) | 0.03 (-8.00, 8.36) | 0.00 (-0.03, 0.03) |
| Papua New Guinea | 1.46 (0.01, 3.11) | 3.54 (0.02, 7.36) | 143.03 (119.11, 169.31) | 11.40 (0.07, 22.97) | 11.46 (0.08, 22.65) | 0.58 (-6.51, 8.17) | 42.32 (0.24, 89.02) | 39.93 (0.23, 82.10) | -5.63 (-14.09, 3.15) | -0.20 (-0.22, -0.17)* |
| Paraguay | 0.71 (0.00, 1.80) | 1.81 (0.01, 4.42) | 152.72 (114.00, 198.23) | 4.25 (0.02, 10.24) | 4.54 (0.02, 10.65) | 6.73 (-6.60, 23.63) | 22.41 (0.09, 54.90) | 25.90 (0.11, 62.84) | 15.53 (-1.48, 36.35) | 0.50 (0.45, 0.55)* |
| Peru | 5.36 (0.02, 11.95) | 7.53 (0.03, 17.27) | 40.49 (11.32, 69.07) | 9.90 (0.05, 21.56) | 8.24 (0.04, 18.05) | -16.72 (-29.44, -5.47) | 30.31 (0.13, 67.30) | 21.77 (0.10, 49.86) | -28.18 (-41.40, -16.31) | -1.18 (-1.32, -1.03)* |
| Philippines | 9.64 (0.04, 23.28) | 17.04 (0.07, 41.00) | 76.85 (66.96, 87.91) | 5.32 (0.03, 12.30) | 5.50 (0.03, 12.61) | 3.28 (1.34, 5.17) | 18.88 (0.08, 44.64) | 16.01 (0.06, 38.25) | -15.24 (-17.11, -13.24) | -0.59 (-0.77, -0.40)* |
| Poland | 4.68 (0.02, 11.09) | 5.28 (0.02, 12.49) | 12.81 (3.18, 22.47) | 4.79 (0.02, 11.36) | 4.49 (0.02, 10.63) | -6.28 (-8.49, -3.95) | 11.43 (0.04, 27.12) | 10.80 (0.04, 25.66) | -5.57 (-8.78, -2.43) | -0.25 (-0.32, -0.19)* |
| Portugal | 3.51 (0.01, 8.74) | 3.45 (0.01, 8.56) | -1.58 (-17.31, 14.84) | 4.07 (0.02, 9.78) | 4.00 (0.02, 9.62) | -1.59 (-8.80, 5.47) | 31.93 (0.13, 80.38) | 27.08 (0.11, 65.80) | -15.18 (-26.05, -3.71) | -0.48 (-0.98, 0.02) |
| Puerto Rico | 1.23 (0.01, 2.76) | 1.35 (0.01, 3.07) | 9.12 (-7.43, 26.09) | 8.05 (0.04, 17.31) | 8.21 (0.04, 18.09) | 1.90 (-10.41, 13.76) | 34.09 (0.15, 76.27) | 33.02 (0.14, 75.63) | -3.13 (-17.50, 10.66) | -0.11 (-0.22, -0.00)* |
| Qatar | 0.18 (0.00, 0.40) | 1.04 (0.00, 2.30) | 475.61 (402.25, 555.50) | 6.03 (0.03, 12.82) | 4.77 (0.02, 10.15) | -20.91 (-29.09, -12.00) | 39.62 (0.17, 88.04) | 28.95 (0.12, 64.61) | -26.94 (-34.48, -18.42) | -1.09 (-1.19, -0.99)* |
| Republic of Korea | 5.98 (0.02, 14.37) | 7.25 (0.03, 17.74) | 21.16 (-7.54, 52.81) | 4.99 (0.02, 11.81) | 3.76 (0.02, 8.91) | -24.68 (-35.82, -13.21) | 12.98 (0.04, 30.54) | 10.86 (0.04, 26.12) | -16.36 (-30.33, 0.26) | -0.61 (-0.73, -0.49)* |
| Republic of Moldova | 2.15 (0.01, 4.84) | 1.75 (0.01, 3.99) | -18.63 (-30.88, -5.71) | 8.95 (0.04, 19.81) | 8.41 (0.04, 18.32) | -6.10 (-13.41, 0.76) | 47.03 (0.20, 107.24) | 36.18 (0.15, 80.79) | -23.08 (-33.85, -11.78) | -0.90 (-1.00, -0.80)* |
| Romania | 7.95 (0.03, 18.02) | 7.23 (0.03, 16.49) | -9.04 (-21.31, 4.92) | 9.51 (0.05, 20.63) | 9.11 (0.04, 20.21) | -4.25 (-10.09, 1.51) | 31.05 (0.13, 69.65) | 28.57 (0.11, 62.79) | -7.99 (-18.94, 4.24) | -0.32 (-0.40, -0.25)* |
| Russian Federation | 65.57 (0.27, 145.97) | 64.03 (0.26, 142.38) | -2.35 (-6.09, 0.86) | 8.91 (0.04, 19.50) | 8.71 (0.04, 19.05) | -2.21 (-5.23, 0.59) | 38.64 (0.16, 87.28) | 35.21 (0.15, 79.76) | -8.87 (-12.14, -5.74) | -0.31 (-0.36, -0.26)* |
| Rwanda | 4.64 (0.02, 9.97) | 7.92 (0.04, 17.35) | 70.84 (47.52, 96.10) | 11.56 (0.06, 24.30) | 11.20 (0.06, 23.71) | -3.07 (-12.57, 5.23) | 102.90 (0.46, 220.40) | 84.11 (0.40, 185.53) | -18.26 (-29.06, -7.33) | -0.71 (-0.89, -0.53)* |
| Saint Kitts and Nevis | 0.02 (0.00, 0.04) | 0.03 (0.00, 0.08) | 81.46 (58.61, 109.33) | 8.21 (0.04, 18.28) | 8.00 (0.04, 17.85) | -2.46 (-6.34, 1.64) | 51.32 (0.21, 120.68) | 49.62 (0.21, 117.67) | -3.32 (-9.80, 3.61) | -0.11 (-0.13, -0.09)* |
| Saint Lucia | 0.04 (0.00, 0.10) | 0.07 (0.00, 0.17) | 70.28 (47.40, 98.13) | 7.88 (0.04, 17.55) | 7.52 (0.04, 16.70) | -4.52 (-9.58, 1.54) | 38.10 (0.16, 86.30) | 36.95 (0.16, 84.36) | -3.02 (-10.49, 5.12) | -0.11 (-0.15, -0.07)* |
| Saint Vincent and the Grenadines | 0.03 (0.00, 0.08) | 0.04 (0.00, 0.10) | 30.13 (11.81, 49.80) | 7.71 (0.04, 17.20) | 7.14 (0.03, 15.96) | -7.36 (-12.68, -1.32) | 37.12 (0.16, 83.73) | 35.95 (0.15, 83.49) | -3.15 (-11.60, 5.58) | -0.11 (-0.20, -0.03)* |
| Samoa | 0.03 (0.00, 0.06) | 0.03 (0.00, 0.08) | 31.31 (17.21, 48.96) | 6.02 (0.03, 13.43) | 6.25 (0.03, 14.01) | 3.80 (-3.02, 12.43) | 19.83 (0.08, 46.52) | 18.29 (0.07, 43.03) | -7.75 (-16.36, 3.35) | -0.28 (-0.33, -0.23)* |
| San Marino | 0.01 (0.00, 0.02) | 0.01 (0.00, 0.03) | 50.43 (34.66, 66.75) | 5.80 (0.03, 13.42) | 6.04 (0.03, 13.86) | 4.07 (-1.14, 9.70) | 35.30 (0.14, 84.68) | 37.17 (0.15, 88.95) | 5.28 (-3.15, 13.43) | 0.18 (0.17, 0.19)* |
| Sao Tome and Principe | 0.04 (0.00, 0.09) | 0.08 (0.00, 0.17) | 98.90 (79.81, 119.81) | 9.86 (0.05, 21.56) | 9.64 (0.05, 20.75) | -2.23 (-7.28, 3.19) | 47.66 (0.20, 106.13) | 47.02 (0.20, 104.98) | -1.36 (-9.16, 7.72) | -0.05 (-0.13, 0.03) |
| Saudi Arabia | 6.38 (0.03, 14.33) | 22.29 (0.10, 49.48) | 249.61 (205.00, 297.75) | 8.30 (0.04, 17.51) | 8.34 (0.04, 17.61) | 0.39 (-7.06, 7.84) | 49.57 (0.22, 111.54) | 53.01 (0.23, 117.32) | 6.93 (-1.32, 16.70) | 0.23 (0.19, 0.28)* |
| Senegal | 2.46 (0.01, 5.45) | 5.71 (0.02, 12.76) | 131.66 (110.87, 156.53) | 9.38 (0.04, 20.02) | 9.51 (0.05, 20.44) | 1.38 (-5.45, 8.92) | 51.01 (0.21, 111.21) | 52.37 (0.22, 115.31) | 2.68 (-7.00, 14.00) | 0.09 (0.04, 0.13)* |
| Serbia | 2.33 (0.01, 5.54) | 2.05 (0.01, 4.88) | -11.73 (-24.23, 0.85) | 5.39 (0.02, 12.76) | 5.33 (0.02, 12.57) | -1.26 (-7.05, 4.60) | 21.88 (0.08, 51.85) | 18.44 (0.07, 43.60) | -15.73 (-25.76, -5.45) | -0.58 (-0.62, -0.54)* |
| Seychelles | 0.01 (0.00, 0.02) | 0.01 (0.00, 0.03) | 49.95 (25.92, 75.32) | 4.89 (0.02, 11.34) | 4.84 (0.02, 11.05) | -1.00 (-8.89, 8.06) | 14.37 (0.06, 33.66) | 12.72 (0.06, 30.39) | -11.51 (-21.32, -1.62) | -0.42 (-0.47, -0.38)* |
| Sierra Leone | 2.00 (0.01, 4.36) | 4.99 (0.03, 10.98) | 149.59 (124.50, 177.83) | 13.16 (0.08, 26.39) | 13.26 (0.08, 26.58) | 0.81 (-4.54, 6.43) | 76.40 (0.41, 164.10) | 83.39 (0.44, 176.41) | 9.15 (-1.35, 21.33) | 0.30 (0.28, 0.32)* |
| Singapore | 1.16 (0.00, 2.62) | 1.31 (0.00, 3.01) | 13.37 (-3.18, 33.57) | 7.35 (0.03, 16.51) | 6.38 (0.03, 14.39) | -13.14 (-20.58, -4.12) | 34.17 (0.13, 77.15) | 19.00 (0.07, 43.82) | -44.39 (-51.99, -35.41) | -2.03 (-2.19, -1.86)* |
| Slovakia | 1.61 (0.01, 3.79) | 1.77 (0.01, 4.07) | 10.02 (-4.25, 25.04) | 7.75 (0.04, 17.65) | 7.71 (0.04, 17.65) | -0.51 (-6.04, 4.69) | 28.58 (0.13, 67.54) | 24.84 (0.11, 57.67) | -13.08 (-23.30, -2.26) | -0.50 (-0.54, -0.47)* |
| Slovenia | 0.88 (0.00, 2.01) | 0.80 (0.00, 1.88) | -8.81 (-22.23, 7.68) | 7.12 (0.03, 16.21) | 6.99 (0.03, 15.98) | -1.80 (-7.47, 4.15) | 38.90 (0.16, 90.14) | 28.56 (0.13, 65.84) | -26.57 (-35.85, -16.88) | -1.07 (-1.13, -1.02)* |
| Solomon Islands | 0.10 (0.00, 0.22) | 0.21 (0.00, 0.46) | 108.21 (84.92, 136.92) | 11.19 (0.07, 22.86) | 11.48 (0.07, 23.03) | 2.66 (-4.30, 9.99) | 39.67 (0.20, 85.39) | 38.35 (0.21, 81.89) | -3.32 (-12.71, 7.00) | -0.12 (-0.15, -0.10)* |
| Somalia | 3.05 (0.01, 6.83) | 8.46 (0.04, 19.23) | 177.03 (145.86, 206.30) | 9.92 (0.05, 21.30) | 10.00 (0.05, 21.37) | 0.83 (-5.24, 7.15) | 69.74 (0.31, 154.91) | 69.93 (0.31, 157.24) | 0.27 (-9.90, 10.27) | 0.00 (-0.02, 0.03) |
| South Africa | 21.59 (0.10, 48.30) | 38.70 (0.18, 83.24) | 79.27 (65.32, 94.57) | 11.73 (0.06, 24.48) | 11.38 (0.06, 23.95) | -2.99 (-7.42, 2.03) | 70.96 (0.33, 151.46) | 68.93 (0.32, 148.00) | -2.86 (-8.36, 2.72) | -0.08 (-0.15, -0.01)* |
| South Sudan | 2.25 (0.01, 5.09) | 4.03 (0.02, 8.96) | 78.82 (59.23, 102.63) | 8.78 (0.04, 18.80) | 9.57 (0.05, 20.40) | 9.00 (2.33, 16.48) | 57.07 (0.24, 126.66) | 64.12 (0.28, 140.26) | 12.36 (1.57, 23.80) | 0.40 (0.38, 0.42)* |
| Spain | 7.23 (0.03, 17.89) | 11.91 (0.04, 29.13) | 64.61 (44.89, 84.51) | 2.83 (0.01, 7.00) | 3.03 (0.01, 7.43) | 6.93 (0.89, 13.64) | 17.09 (0.06, 42.42) | 22.40 (0.08, 55.13) | 31.04 (17.65, 43.43) | 1.02 (0.74, 1.30)* |
| Sri Lanka | 4.75 (0.02, 11.36) | 3.99 (0.02, 9.17) | -16.01 (-32.35, 2.69) | 6.76 (0.03, 15.16) | 5.67 (0.03, 13.08) | -16.12 (-27.80, -4.95) | 27.89 (0.12, 64.65) | 17.21 (0.07, 40.18) | -38.28 (-48.67, -27.97) | -1.66 (-1.92, -1.40)* |
| Sudan | 13.06 (0.07, 28.03) | 28.02 (0.15, 59.04) | 114.59 (89.60, 146.72) | 12.02 (0.08, 24.07) | 11.70 (0.07, 23.32) | -2.60 (-10.49, 7.15) | 84.73 (0.46, 181.83) | 80.50 (0.43, 166.73) | -4.99 (-15.14, 7.97) | -0.18 (-0.21, -0.14)* |
| Suriname | 0.21 (0.00, 0.50) | 0.37 (0.00, 0.85) | 75.39 (55.98, 102.53) | 7.91 (0.04, 17.94) | 7.83 (0.04, 17.26) | -1.04 (-6.74, 4.92) | 58.15 (0.25, 136.97) | 60.90 (0.25, 141.43) | 4.72 (-5.08, 16.16) | 0.17 (0.14, 0.20)* |
| Sweden | 4.57 (0.02, 10.42) | 5.18 (0.02, 11.71) | 13.41 (3.18, 24.86) | 7.68 (0.04, 17.29) | 7.67 (0.04, 17.35) | -0.08 (-5.20, 5.32) | 48.26 (0.19, 109.55) | 46.90 (0.19, 107.48) | -2.83 (-10.96, 6.31) | 0.01 (-0.36, 0.37) |
| Switzerland | 1.78 (0.01, 4.36) | 1.75 (0.01, 4.16) | -1.87 (-14.64, 12.95) | 3.37 (0.01, 8.06) | 3.31 (0.01, 8.07) | -1.81 (-8.54, 5.79) | 22.34 (0.08, 55.21) | 17.03 (0.06, 40.96) | -23.76 (-31.89, -13.68) | -0.93 (-1.08, -0.78)* |
| Syrian Arab Republic | 5.34 (0.02, 12.01) | 9.07 (0.04, 20.70) | 70.06 (48.25, 96.24) | 9.49 (0.05, 20.05) | 10.35 (0.05, 21.85) | 9.05 (3.07, 14.78) | 59.13 (0.26, 132.12) | 64.21 (0.30, 145.18) | 8.60 (-0.17, 17.56) | 0.28 (0.22, 0.35)* |
| Taiwan (Province of China) | 2.93 (0.01, 6.76) | 5.15 (0.02, 11.81) | 75.99 (44.74, 111.99) | 6.42 (0.03, 14.58) | 6.99 (0.03, 16.07) | 8.90 (1.69, 17.08) | 14.21 (0.06, 32.44) | 16.77 (0.07, 39.28) | 18.08 (4.50, 32.80) | 0.58 (0.55, 0.60)* |
| Tajikistan | 1.01 (0.00, 2.52) | 1.91 (0.01, 4.52) | 88.45 (67.18, 113.83) | 6.90 (0.03, 15.75) | 6.79 (0.03, 15.67) | -1.57 (-6.91, 4.34) | 26.78 (0.11, 63.62) | 23.68 (0.10, 56.11) | -11.57 (-20.54, -1.08) | -0.43 (-0.49, -0.38)* |
| Thailand | 4.93 (0.02, 13.15) | 7.27 (0.03, 17.88) | 47.36 (15.24, 87.21) | 2.89 (0.01, 7.18) | 2.79 (0.01, 6.92) | -3.64 (-12.38, 5.91) | 8.66 (0.03, 22.42) | 8.37 (0.03, 20.86) | -3.31 (-15.20, 11.40) | -0.13 (-0.23, -0.02)* |
| Timor-Leste | 0.20 (0.00, 0.44) | 0.28 (0.00, 0.62) | 43.97 (25.06, 64.67) | 8.64 (0.05, 17.93) | 8.46 (0.05, 17.80) | -2.07 (-9.91, 6.27) | 30.09 (0.14, 66.30) | 24.72 (0.11, 54.89) | -17.86 (-26.77, -8.15) | -0.66 (-0.84, -0.47)* |
| Togo | 1.18 (0.00, 2.71) | 3.11 (0.01, 7.10) | 163.96 (119.42, 212.46) | 8.67 (0.04, 19.08) | 8.45 (0.04, 18.75) | -2.52 (-13.61, 10.64) | 53.92 (0.22, 120.77) | 54.87 (0.24, 124.63) | 1.77 (-12.89, 17.92) | 0.06 (0.02, 0.11)* |
| Tokelau | 0.00 (0.00, 0.00) | 0.00 (0.00, 0.00) | -7.73 (-15.04, 1.14) | 10.37 (0.06, 21.73) | 9.84 (0.05, 20.63) | -5.11 (-10.50, 0.53) | 37.93 (0.18, 85.08) | 35.41 (0.18, 78.14) | -6.67 (-13.89, 1.65) | -0.24 (-0.33, -0.14)* |
| Tonga | 0.02 (0.00, 0.04) | 0.02 (0.00, 0.05) | 13.10 (0.27, 26.67) | 9.09 (0.05, 19.78) | 8.89 (0.05, 19.13) | -2.15 (-8.93, 4.87) | 24.74 (0.11, 55.36) | 23.78 (0.11, 54.19) | -3.87 (-13.54, 6.27) | -0.14 (-0.20, -0.10)* |
| Trinidad and Tobago | 0.57 (0.00, 1.36) | 0.69 (0.00, 1.59) | 20.95 (2.30, 43.00) | 7.79 (0.04, 17.48) | 7.36 (0.04, 16.45) | -5.45 (-11.94, 2.12) | 49.85 (0.22, 116.72) | 44.17 (0.18, 103.54) | -11.40 (-21.99, 0.21) | -0.42 (-0.45, -0.38)* |
| Tunisia | 5.54 (0.02, 12.33) | 10.17 (0.05, 22.67) | 83.32 (60.13, 110.16) | 9.66 (0.05, 20.54) | 9.63 (0.05, 20.59) | -0.24 (-5.96, 6.22) | 79.36 (0.35, 173.53) | 78.82 (0.35, 175.82) | -0.68 (-9.76, 9.34) | -0.03 (-0.06, -0.01)* |
| Turkey | 36.40 (0.17, 80.02) | 59.26 (0.29, 130.69) | 62.79 (35.85, 93.14) | 11.52 (0.06, 24.00) | 10.83 (0.06, 22.77) | -5.93 (-15.01, 3.29) | 69.90 (0.32, 152.57) | 63.41 (0.30, 141.06) | -9.28 (-23.12, 6.04) | -0.35 (-0.44, -0.26)* |
| Turkmenistan | 0.59 (0.00, 1.42) | 0.91 (0.00, 2.18) | 55.16 (32.52, 81.15) | 4.97 (0.02, 11.73) | 4.74 (0.02, 11.24) | -4.54 (-10.67, 1.33) | 21.72 (0.08, 52.34) | 18.86 (0.07, 44.90) | -13.15 (-23.06, -2.18) | -0.49 (-0.52, -0.46)* |
| Tuvalu | 0.00 (0.00, 0.01) | 0.00 (0.00, 0.01) | 21.26 (10.83, 32.85) | 10.85 (0.06, 22.57) | 9.69 (0.05, 20.59) | -10.64 (-15.50, -5.35) | 39.66 (0.19, 88.00) | 34.91 (0.16, 77.89) | -11.99 (-18.97, -4.16) | -0.44 (-0.47, -0.41)* |
| Uganda | 14.30 (0.07, 31.50) | 33.30 (0.17, 71.88) | 132.93 (108.80, 158.07) | 12.37 (0.07, 25.21) | 12.44 (0.07, 25.35) | 0.59 (-5.99, 7.27) | 135.46 (0.66, 289.23) | 132.68 (0.68, 278.76) | -2.05 (-11.59, 8.41) | -0.09 (-0.15, -0.03)* |
| Ukraine | 21.37 (0.08, 51.74) | 17.60 (0.06, 42.45) | -17.60 (-24.26, -10.52) | 5.37 (0.02, 13.41) | 5.26 (0.02, 13.00) | -1.93 (-6.00, 2.80) | 34.67 (0.12, 85.11) | 30.15 (0.10, 75.01) | -13.04 (-18.50, -6.59) | -0.48 (-0.57, -0.40)* |
| United Arab Emirates | 0.63 (0.00, 1.42) | 3.23 (0.01, 7.24) | 412.78 (308.87, 536.13) | 5.99 (0.03, 12.68) | 5.52 (0.03, 11.76) | -7.93 (-17.56, 1.47) | 34.52 (0.16, 76.95) | 29.01 (0.13, 65.14) | -15.96 (-25.17, -6.57) | -0.61 (-0.68, -0.54)* |
| United Kingdom | 33.68 (0.14, 75.41) | 34.52 (0.14, 77.71) | 2.50 (-1.31, 6.36) | 7.49 (0.04, 16.48) | 7.59 (0.04, 16.60) | 1.30 (-0.45, 3.12) | 53.06 (0.22, 117.81) | 46.22 (0.20, 102.66) | -12.88 (-14.98, -10.56) | -0.49 (-0.58, -0.40)* |
| United Republic of Tanzania | 12.00 (0.05, 26.92) | 27.76 (0.13, 61.51) | 131.41 (100.89, 165.16) | 10.48 (0.05, 22.39) | 10.66 (0.06, 22.59) | 1.73 (-7.42, 12.08) | 72.05 (0.31, 156.17) | 70.25 (0.31, 153.28) | -2.50 (-14.33, 11.10) | -0.08 (-0.11, -0.04)* |
| United States Virgin Islands | 0.04 (0.00, 0.10) | 0.05 (0.00, 0.11) | 12.24 (-3.97, 32.27) | 8.54 (0.04, 18.72) | 8.36 (0.04, 18.40) | -2.10 (-7.07, 3.72) | 41.25 (0.17, 91.36) | 41.87 (0.17, 94.24) | 1.50 (-7.86, 10.74) | 0.04 (-0.15, 0.23) |
| United States of America | 133.60 (0.60, 292.92) | 215.18 (0.96, 467.31) | 61.06 (52.63, 70.65) | 9.79 (0.05, 20.81) | 9.57 (0.05, 20.50) | -2.27 (-6.36, 1.62) | 48.56 (0.22, 107.21) | 63.14 (0.29, 139.83) | 30.02 (23.76, 36.07) | 0.89 (0.78, 1.00)* |
| Uruguay | 0.68 (0.00, 1.60) | 0.91 (0.00, 2.18) | 33.18 (18.49, 50.55) | 5.65 (0.03, 13.42) | 5.62 (0.03, 13.02) | -0.52 (-6.98, 6.76) | 21.57 (0.09, 50.73) | 24.56 (0.10, 59.75) | 13.89 (1.04, 28.97) | 0.47 (0.21, 0.74)* |
| Uzbekistan | 3.43 (0.01, 8.35) | 6.22 (0.02, 15.15) | 81.68 (57.94, 110.38) | 4.92 (0.02, 11.57) | 4.75 (0.02, 11.49) | -3.39 (-9.47, 2.78) | 22.22 (0.08, 51.95) | 19.95 (0.08, 48.14) | -10.23 (-20.82, 1.88) | -0.37 (-0.40, -0.34)* |
| Vanuatu | 0.05 (0.00, 0.10) | 0.10 (0.00, 0.21) | 101.55 (81.00, 125.31) | 11.07 (0.07, 22.53) | 11.20 (0.07, 22.68) | 1.19 (-5.10, 8.47) | 39.16 (0.21, 84.69) | 36.75 (0.18, 77.50) | -6.15 (-14.94, 3.84) | -0.23 (-0.28, -0.18)* |
| Venezuela (Bolivarian Republic of) | 5.48 (0.02, 12.85) | 10.56 (0.04, 24.32) | 92.59 (67.08, 120.58) | 7.46 (0.04, 16.86) | 7.29 (0.03, 16.39) | -2.22 (-7.85, 3.43) | 35.37 (0.14, 81.27) | 35.23 (0.14, 81.64) | -0.39 (-8.46, 8.61) | -0.01 (-0.04, 0.02) |
| Viet Nam | 11.47 (0.05, 27.54) | 17.80 (0.07, 41.13) | 55.20 (31.46, 83.01) | 7.97 (0.04, 17.68) | 7.44 (0.04, 16.71) | -6.69 (-12.29, -0.68) | 20.00 (0.08, 46.92) | 16.55 (0.07, 37.90) | -17.24 (-25.49, -7.08) | -0.65 (-0.73, -0.58)* |
| Yemen | 7.13 (0.03, 15.75) | 20.01 (0.09, 45.07) | 180.54 (154.77, 210.67) | 10.29 (0.05, 21.95) | 10.13 (0.05, 21.57) | -1.57 (-7.04, 4.26) | 79.51 (0.36, 175.60) | 79.03 (0.36, 172.74) | -0.60 (-8.92, 9.03) | -0.02 (-0.04, -0.00)* |
| Zambia | 3.43 (0.02, 7.55) | 8.09 (0.04, 17.22) | 136.15 (106.45, 168.75) | 12.13 (0.07, 24.49) | 11.60 (0.07, 23.82) | -4.42 (-13.69, 5.87) | 68.75 (0.37, 148.17) | 65.74 (0.35, 137.32) | -4.37 (-16.22, 7.96) | -0.16 (-0.19, -0.13)* |
| Zimbabwe | 2.59 (0.01, 5.75) | 4.48 (0.02, 10.00) | 73.22 (50.87, 98.69) | 9.93 (0.05, 21.26) | 9.92 (0.05, 21.12) | -0.10 (-10.78, 10.56) | 39.58 (0.17, 86.25) | 40.87 (0.19, 92.57) | 3.27 (-10.22, 16.27) | 0.11 (0.04, 0.18)* |

| Table S16 The number, PAFs and ASDR of MDD attributed to CSA in 1990 and 2019, and the corresponding percentage change, and AAPC of ASDR during 1990-2019 across 204 countries and territories. | | | | | | | | | | |
| --- | --- | --- | --- | --- | --- | --- | --- | --- | --- | --- |
|  |  |  | Percentage change of |  |  | Percentage change of |  |  | Percentage change of | AAPC (95% CI) |
| Countries or territories | CSA-related DALYs  number ×10^3^ (95% UI) | | number (%, 95% UI) | PAFs (%, 95% UI) | | PAFs (%, 95% UI) | CSA-related ASDR per 100 000 (95% UI) | | ASDR (%, 95% UI) | of CSA-related ASDR |
|  | 1990 year | 2019 year | during 1990-2019 | 1990 year | 2019 year | during 1990-2019 | 1990 year | 2019 year | during 1990-2019 | during 1990-2019 |
| Afghanistan | 1.98 (0.86, 3.50) | 6.97 (3.16, 12.45) | 251.80 (211.42, 297.83) | 3.03 (1.58, 4.98) | 3.16 (1.65, 5.17) | 4.34 (-1.54, 11.04) | 23.84 (10.51, 42.32) | 24.76 (11.29, 44.14) | 3.85 (-5.80, 13.74) | 0.13 (0.12, 0.14)* |
| Albania | 0.30 (0.13, 0.54) | 0.32 (0.14, 0.57) | 6.41 (-8.03, 26.78) | 3.78 (2.03, 6.02) | 3.79 (2.04, 6.00) | 0.42 (-3.10, 3.91) | 9.81 (4.31, 17.46) | 10.48 (4.71, 18.53) | 6.84 (-0.75, 15.67) | 0.23 (0.20, 0.27)* |
| Algeria | 3.72 (1.66, 6.57) | 8.05 (3.52, 14.27) | 116.47 (91.36, 142.00) | 2.96 (1.55, 4.86) | 2.94 (1.54, 4.79) | -0.80 (-5.58, 3.40) | 19.39 (8.76, 34.38) | 18.46 (8.03, 32.46) | -4.79 (-12.73, 2.85) | -0.17 (-0.20, -0.14)* |
| American Samoa | 0.00 (0.00, 0.01) | 0.01 (0.00, 0.01) | 26.52 (12.16, 43.34) | 3.94 (2.10, 6.24) | 3.98 (2.13, 6.35) | 0.89 (-2.52, 4.35) | 10.61 (4.85, 18.51) | 10.34 (4.65, 18.25) | -2.53 (-10.14, 5.73) | -0.09 (-0.14, -0.04)* |
| Andorra | 0.02 (0.01, 0.03) | 0.03 (0.01, 0.05) | 57.10 (36.00, 80.74) | 4.92 (2.68, 7.87) | 4.95 (2.69, 7.92) | 0.63 (-1.75, 3.35) | 26.19 (11.74, 46.11) | 25.63 (11.40, 45.30) | -2.14 (-8.53, 4.65) | -0.08 (-0.13, -0.02)* |
| Angola | 4.62 (2.12, 7.99) | 12.64 (5.72, 22.07) | 173.27 (151.14, 197.64) | 6.27 (3.43, 9.83) | 6.19 (3.34, 9.71) | -1.22 (-4.53, 1.87) | 60.18 (27.26, 105.33) | 56.69 (26.01, 99.05) | -5.80 (-12.62, 2.20) | -0.21 (-0.22, -0.19)* |
| Antigua and Barbuda | 0.01 (0.00, 0.01) | 0.01 (0.01, 0.03) | 75.67 (55.88, 96.34) | 3.20 (1.70, 5.21) | 3.21 (1.71, 5.24) | 0.10 (-2.26, 2.64) | 13.93 (6.02, 24.91) | 13.93 (5.94, 24.84) | -0.02 (-6.37, 6.53) | -0.00 (-0.08, 0.07) |
| Argentina | 7.12 (3.26, 12.62) | 9.81 (4.49, 17.31) | 37.75 (22.77, 54.90) | 5.74 (3.12, 9.32) | 5.58 (3.04, 8.91) | -2.83 (-8.10, 2.04) | 22.19 (10.16, 39.60) | 20.56 (9.41, 36.26) | -7.37 (-17.94, 4.73) | -0.25 (-0.31, -0.18)* |
| Armenia | 0.18 (0.08, 0.32) | 0.21 (0.09, 0.37) | 16.39 (3.66, 31.51) | 1.55 (0.82, 2.52) | 1.53 (0.81, 2.49) | -0.82 (-4.26, 2.87) | 5.43 (2.36, 9.66) | 5.87 (2.56, 10.39) | 8.11 (-0.37, 16.79) | 0.28 (0.21, 0.34)* |
| Australia | 5.76 (2.72, 9.85) | 9.11 (4.18, 16.04) | 58.01 (30.67, 84.65) | 4.55 (2.45, 7.05) | 5.01 (2.70, 7.88) | 10.02 (-6.15, 27.60) | 31.79 (14.97, 54.48) | 34.99 (16.20, 61.37) | 10.08 (-9.27, 30.20) | 0.40 (0.14, 0.66)* |
| Austria | 2.24 (1.02, 3.96) | 2.08 (0.96, 3.65) | -7.26 (-16.94, 3.82) | 5.01 (2.68, 7.93) | 4.98 (2.67, 7.87) | -0.63 (-4.28, 2.50) | 26.05 (11.83, 46.02) | 20.62 (9.17, 36.27) | -20.85 (-28.35, -13.25) | -0.81 (-0.85, -0.76)* |
| Azerbaijan | 0.34 (0.15, 0.61) | 0.59 (0.26, 1.07) | 72.06 (52.37, 93.23) | 1.54 (0.81, 2.48) | 1.55 (0.82, 2.53) | 0.55 (-2.24, 3.47) | 5.20 (2.27, 9.29) | 5.20 (2.29, 9.16) | -0.07 (-6.99, 8.12) | -0.02 (-0.15, 0.10) |
| Bahamas | 0.04 (0.02, 0.06) | 0.06 (0.03, 0.11) | 69.58 (49.21, 92.58) | 3.21 (1.70, 5.23) | 3.21 (1.70, 5.23) | 0.04 (-2.45, 2.56) | 14.43 (6.30, 25.78) | 14.16 (6.13, 25.15) | -1.86 (-8.30, 5.32) | -0.07 (-0.16, 0.03) |
| Bahrain | 0.14 (0.06, 0.26) | 0.50 (0.22, 0.92) | 245.23 (175.36, 322.70) | 3.42 (1.76, 5.66) | 3.47 (1.77, 5.78) | 1.68 (-3.90, 7.73) | 28.28 (12.67, 50.98) | 24.98 (11.02, 45.53) | -11.65 (-20.07, -1.86) | -0.43 (-0.47, -0.39)* |
| Bangladesh | 21.23 (9.37, 37.84) | 41.04 (18.37, 73.81) | 93.30 (70.06, 119.48) | 3.76 (2.00, 5.99) | 3.76 (2.01, 6.06) | -0.14 (-5.18, 4.84) | 27.60 (12.38, 49.27) | 26.44 (12.00, 47.62) | -4.21 (-12.31, 5.37) | -0.14 (-0.18, -0.10)* |
| Barbados | 0.04 (0.02, 0.07) | 0.05 (0.02, 0.09) | 33.08 (16.95, 50.65) | 3.19 (1.70, 5.12) | 3.19 (1.68, 5.17) | -0.08 (-2.55, 2.47) | 14.69 (6.40, 26.74) | 14.86 (6.51, 26.31) | 1.19 (-5.25, 8.54) | 0.04 (-0.00, 0.08) |
| Belarus | 2.70 (1.22, 4.87) | 2.62 (1.18, 4.59) | -2.82 (-13.13, 7.70) | 4.10 (2.22, 6.62) | 4.09 (2.21, 6.54) | -0.16 (-3.52, 3.48) | 23.62 (10.66, 41.89) | 22.84 (10.08, 40.15) | -3.32 (-12.59, 5.75) | -0.11 (-0.14, -0.07)* |
| Belgium | 2.57 (1.18, 4.50) | 3.26 (1.50, 5.71) | 26.65 (11.30, 43.35) | 4.93 (2.65, 7.81) | 5.02 (2.70, 8.01) | 1.69 (-1.70, 6.07) | 23.19 (10.69, 40.81) | 26.09 (11.61, 45.82) | 12.48 (-1.77, 27.23) | 0.41 (0.37, 0.45)* |
| Belize | 0.02 (0.01, 0.04) | 0.06 (0.03, 0.11) | 192.71 (164.84, 224.07) | 3.19 (1.69, 5.08) | 3.19 (1.68, 5.16) | 0.25 (-2.14, 3.00) | 15.14 (6.69, 26.87) | 15.78 (6.92, 27.97) | 4.22 (-3.50, 11.93) | 0.17 (-0.01, 0.36) |
| Benin | 1.95 (0.88, 3.37) | 5.62 (2.62, 9.62) | 188.16 (167.84, 211.98) | 10.08 (5.66, 15.52) | 10.01 (5.64, 15.61) | -0.65 (-3.25, 2.01) | 61.89 (28.42, 107.49) | 64.15 (30.39, 109.17) | 3.65 (-3.45, 12.23) | 0.12 (0.10, 0.14)* |
| Bermuda | 0.01 (0.01, 0.02) | 0.01 (0.01, 0.02) | -5.35 (-19.00, 10.37) | 3.21 (1.70, 5.21) | 3.17 (1.68, 5.14) | -1.23 (-4.68, 2.05) | 17.78 (7.80, 31.70) | 14.81 (6.42, 26.27) | -16.69 (-24.30, -8.33) | -0.63 (-0.67, -0.59)* |
| Bhutan | 0.14 (0.06, 0.25) | 0.22 (0.10, 0.40) | 61.87 (47.28, 78.17) | 5.33 (2.92, 8.51) | 5.31 (2.88, 8.46) | -0.37 (-3.16, 2.23) | 33.47 (15.10, 59.71) | 30.80 (13.82, 54.63) | -7.99 (-14.94, -0.15) | -0.29 (-0.32, -0.26)* |
| Bolivia (Plurinational State of) | 2.49 (1.17, 4.29) | 4.93 (2.29, 8.52) | 98.35 (80.66, 118.91) | 8.76 (4.84, 13.78) | 8.66 (4.82, 13.52) | -1.14 (-4.60, 2.26) | 49.55 (22.97, 85.44) | 44.20 (20.41, 77.38) | -10.81 (-17.98, -2.71) | -0.39 (-0.41, -0.38)* |
| Bosnia and Herzegovina | 0.82 (0.37, 1.46) | 0.50 (0.22, 0.88) | -39.05 (-48.48, -27.54) | 3.80 (2.04, 6.05) | 3.75 (2.01, 5.96) | -1.46 (-6.32, 2.70) | 16.95 (7.67, 29.87) | 12.60 (5.59, 22.32) | -25.63 (-34.05, -16.49) | -1.02 (-1.11, -0.94)* |
| Botswana | 0.37 (0.17, 0.64) | 0.83 (0.37, 1.46) | 125.22 (93.72, 157.27) | 6.37 (3.44, 9.95) | 6.23 (3.39, 9.73) | -2.24 (-8.07, 3.73) | 36.74 (16.89, 64.50) | 36.87 (16.69, 64.27) | 0.35 (-9.38, 10.02) | 0.01 (-0.02, 0.04) |
| Brazil | 36.02 (15.60, 64.84) | 53.03 (23.32, 95.01) | 47.22 (32.73, 64.25) | 4.15 (2.16, 6.81) | 3.69 (1.96, 6.06) | -11.06 (-18.72, -3.52) | 26.52 (11.78, 47.62) | 22.14 (9.86, 39.51) | -16.50 (-24.46, -8.11) | -0.63 (-0.73, -0.52)* |
| Brunei Darussalam | 0.02 (0.01, 0.04) | 0.05 (0.02, 0.09) | 109.73 (84.64, 138.48) | 5.07 (2.77, 8.05) | 5.07 (2.75, 8.19) | 0.15 (-3.10, 3.65) | 9.97 (4.42, 17.41) | 10.04 (4.46, 17.42) | 0.73 (-7.86, 9.24) | 0.02 (-0.03, 0.06) |
| Bulgaria | 1.42 (0.63, 2.54) | 1.04 (0.47, 1.84) | -26.61 (-34.35, -17.22) | 3.74 (1.99, 6.01) | 3.74 (2.01, 6.05) | -0.11 (-3.83, 4.04) | 14.41 (6.44, 25.47) | 12.05 (5.41, 21.38) | -16.35 (-24.36, -7.43) | -0.62 (-0.65, -0.59)* |
| Burkina Faso | 4.00 (1.82, 6.91) | 9.74 (4.44, 16.92) | 143.80 (125.81, 164.24) | 10.03 (5.66, 15.49) | 9.99 (5.61, 15.42) | -0.40 (-2.71, 1.96) | 63.66 (29.24, 109.53) | 62.42 (29.22, 108.51) | -1.96 (-8.88, 5.79) | -0.03 (-0.14, 0.09) |
| Burundi | 1.71 (0.78, 3.07) | 3.07 (1.39, 5.43) | 79.17 (65.31, 93.92) | 5.50 (2.95, 8.59) | 5.68 (3.05, 8.91) | 3.26 (-1.38, 8.24) | 46.55 (21.30, 80.78) | 38.11 (16.86, 67.31) | -18.12 (-24.72, -10.72) | -0.69 (-0.72, -0.66)* |
| Cabo Verde | 0.17 (0.08, 0.30) | 0.40 (0.19, 0.69) | 132.69 (108.64, 159.03) | 10.05 (5.67, 15.44) | 9.95 (5.64, 15.46) | -0.98 (-5.06, 2.91) | 67.73 (31.13, 118.17) | 72.67 (34.20, 125.99) | 7.30 (-1.29, 16.24) | 0.26 (0.19, 0.32)* |
| Cambodia | 0.87 (0.38, 1.60) | 1.59 (0.69, 2.94) | 82.74 (60.30, 107.50) | 2.89 (1.50, 4.75) | 3.01 (1.53, 5.02) | 3.84 (-6.16, 13.19) | 10.91 (4.85, 19.48) | 9.61 (4.27, 17.63) | -11.89 (-22.05, -1.52) | -0.43 (-0.51, -0.34)* |
| Cameroon | 4.66 (2.18, 7.91) | 14.64 (6.73, 24.95) | 213.94 (190.68, 239.97) | 10.07 (5.66, 15.58) | 10.02 (5.66, 15.43) | -0.46 (-2.63, 1.71) | 64.74 (30.24, 111.15) | 67.38 (31.86, 115.24) | 4.08 (-3.01, 11.79) | 0.14 (0.12, 0.16)* |
| Canada | 4.21 (1.89, 7.51) | 8.99 (4.38, 15.32) | 113.74 (77.46, 155.40) | 3.19 (1.70, 5.07) | 5.48 (2.96, 8.44) | 71.71 (45.86, 103.56) | 14.02 (6.33, 25.01) | 24.03 (11.64, 41.35) | 71.42 (41.05, 105.98) | 1.83 (1.54, 2.12)* |
| Central African Republic | 1.26 (0.56, 2.20) | 2.50 (1.13, 4.42) | 99.03 (85.67, 113.98) | 6.24 (3.37, 9.77) | 6.23 (3.33, 9.71) | -0.20 (-2.50, 2.19) | 59.94 (27.27, 104.49) | 59.05 (27.15, 104.68) | -1.48 (-7.98, 5.33) | -0.06 (-0.08, -0.03)* |
| Chad | 3.09 (1.43, 5.30) | 8.05 (3.71, 13.82) | 160.89 (138.89, 181.89) | 10.08 (5.70, 15.57) | 10.07 (5.68, 15.52) | -0.13 (-2.83, 2.60) | 76.67 (35.59, 134.15) | 78.95 (36.40, 136.27) | 2.97 (-5.37, 11.42) | 0.10 (0.07, 0.12)* |
| Chile | 5.13 (2.35, 9.29) | 6.41 (2.90, 11.26) | 24.98 (8.94, 43.30) | 5.29 (2.85, 8.54) | 5.21 (2.84, 8.32) | -1.58 (-6.70, 2.85) | 40.03 (18.38, 71.89) | 31.64 (14.40, 54.96) | -20.95 (-30.63, -10.01) | -0.81 (-0.85, -0.76)* |
| China | 106.30 (47.19, 189.42) | 140.35 (63.28, 252.25) | 32.04 (9.64, 65.12) | 2.61 (1.38, 4.22) | 2.67 (1.41, 4.32) | 2.22 (-7.03, 13.39) | 8.76 (3.94, 15.63) | 7.61 (3.38, 13.57) | -13.08 (-21.67, -1.96) | -0.54 (-0.72, -0.36)* |
| Colombia | 4.96 (2.24, 8.60) | 7.79 (3.57, 13.58) | 57.03 (35.47, 81.81) | 5.35 (2.92, 8.45) | 5.29 (2.89, 8.27) | -1.09 (-4.74, 2.34) | 17.43 (7.92, 30.73) | 15.13 (6.95, 26.50) | -13.21 (-22.43, -4.03) | -0.49 (-0.58, -0.40)* |
| Comoros | 0.11 (0.05, 0.19) | 0.20 (0.09, 0.36) | 88.78 (71.36, 108.38) | 5.59 (2.97, 8.88) | 5.50 (2.94, 8.72) | -1.66 (-6.38, 3.06) | 34.22 (15.62, 60.57) | 32.05 (14.49, 56.17) | -6.33 (-14.52, 3.08) | -0.22 (-0.25, -0.20)* |
| Congo | 1.08 (0.49, 1.91) | 2.49 (1.14, 4.42) | 129.62 (106.66, 152.45) | 6.21 (3.34, 9.71) | 6.20 (3.35, 9.65) | -0.12 (-2.74, 2.65) | 59.42 (27.32, 102.97) | 53.91 (24.80, 95.71) | -9.28 (-15.98, -2.60) | -0.34 (-0.37, -0.31)* |
| Cook Islands | 0.00 (0.00, 0.00) | 0.00 (0.00, 0.01) | 12.81 (-0.54, 28.45) | 4.03 (2.16, 6.45) | 4.06 (2.19, 6.47) | 0.75 (-2.24, 4.11) | 14.64 (6.32, 26.49) | 14.82 (6.31, 26.48) | 1.20 (-4.74, 7.67) | 0.04 (0.00, 0.07)* |
| Costa Rica | 1.37 (0.64, 2.32) | 2.89 (1.35, 5.00) | 111.45 (88.72, 135.95) | 11.48 (6.44, 17.49) | 11.55 (6.49, 17.65) | 0.60 (-1.80, 3.34) | 52.56 (24.73, 89.64) | 55.98 (26.07, 96.45) | 6.49 (-1.61, 14.12) | 0.24 (0.19, 0.29)* |
| Croatia | 1.02 (0.46, 1.80) | 0.77 (0.34, 1.34) | -24.44 (-32.76, -14.50) | 3.79 (2.01, 6.11) | 3.80 (2.03, 6.12) | 0.14 (-3.65, 4.04) | 18.26 (8.30, 32.20) | 14.67 (6.63, 25.80) | -19.65 (-26.93, -10.87) | -0.76 (-0.79, -0.73)* |
| Cuba | 3.11 (1.40, 5.68) | 2.59 (1.16, 4.57) | -16.86 (-31.12, -0.03) | 3.26 (1.74, 5.35) | 3.14 (1.68, 5.09) | -3.68 (-9.49, 2.42) | 26.99 (11.98, 49.30) | 18.71 (8.21, 33.23) | -30.68 (-38.63, -22.48) | -1.26 (-1.29, -1.23)* |
| Cyprus | 0.17 (0.07, 0.29) | 0.31 (0.14, 0.54) | 86.44 (68.78, 105.17) | 4.97 (2.71, 7.89) | 4.99 (2.71, 7.85) | 0.52 (-1.92, 3.59) | 20.49 (9.08, 36.01) | 20.60 (9.22, 36.45) | 0.53 (-6.25, 8.11) | 0.01 (-0.05, 0.07) |
| Czechia | 3.40 (1.58, 5.98) | 3.03 (1.36, 5.27) | -10.78 (-20.38, 0.47) | 6.71 (3.66, 10.59) | 6.63 (3.63, 10.36) | -1.17 (-5.15, 2.49) | 29.73 (13.70, 52.35) | 23.88 (10.72, 41.39) | -19.67 (-27.57, -10.88) | -0.76 (-0.80, -0.71)* |
| Côte d’Ivoire | 4.27 (1.95, 7.35) | 10.38 (4.79, 17.86) | 142.99 (124.14, 164.02) | 9.79 (5.56, 15.06) | 9.75 (5.55, 15.03) | -0.40 (-2.42, 1.71) | 51.54 (24.17, 89.94) | 52.08 (24.08, 89.74) | 1.05 (-5.71, 8.81) | 0.04 (0.00, 0.07)* |
| Democratic People's Republic of Korea | 1.19 (0.52, 2.17) | 1.57 (0.68, 2.92) | 32.43 (19.54, 47.22) | 1.92 (1.00, 3.20) | 1.93 (1.00, 3.17) | 0.52 (-3.12, 4.68) | 5.84 (2.55, 10.58) | 5.08 (2.22, 9.36) | -12.97 (-20.28, -4.88) | -0.48 (-0.51, -0.46)* |
| Democratic Republic of the Congo | 15.08 (6.82, 26.66) | 35.93 (16.12, 62.95) | 138.16 (120.30, 157.46) | 6.20 (3.37, 9.71) | 6.18 (3.33, 9.64) | -0.22 (-3.00, 2.95) | 53.94 (24.76, 94.69) | 52.54 (23.62, 92.48) | -2.59 (-9.15, 4.69) | -0.09 (-0.11, -0.08)* |
| Denmark | 1.39 (0.62, 2.45) | 1.10 (0.49, 1.97) | -21.07 (-29.89, -11.04) | 3.50 (1.88, 5.66) | 3.46 (1.85, 5.53) | -1.30 (-5.18, 1.99) | 23.78 (10.45, 41.87) | 16.89 (7.48, 29.70) | -28.95 (-36.00, -20.57) | -1.19 (-1.25, -1.13)* |
| Djibouti | 0.11 (0.05, 0.20) | 0.37 (0.17, 0.66) | 228.48 (191.43, 267.04) | 5.71 (3.06, 9.00) | 5.80 (3.08, 9.23) | 1.60 (-2.61, 6.05) | 35.66 (16.07, 63.63) | 36.49 (16.88, 65.18) | 2.32 (-5.65, 10.29) | 0.08 (0.07, 0.10)* |
| Dominica | 0.01 (0.00, 0.02) | 0.01 (0.00, 0.02) | 13.77 (3.93, 24.88) | 3.18 (1.70, 5.13) | 3.19 (1.69, 5.17) | 0.33 (-3.13, 3.90) | 14.11 (6.09, 25.13) | 14.24 (6.20, 25.22) | 0.96 (-6.08, 8.20) | 0.03 (-0.02, 0.08) |
| Dominican Republic | 1.16 (0.50, 2.08) | 2.09 (0.89, 3.73) | 80.46 (60.70, 102.66) | 3.22 (1.70, 5.21) | 3.18 (1.69, 5.09) | -1.30 (-4.58, 1.63) | 19.04 (8.27, 34.24) | 19.11 (8.24, 34.14) | 0.35 (-7.25, 8.51) | 0.00 (-0.04, 0.05) |
| Ecuador | 3.62 (1.64, 6.31) | 7.36 (3.40, 12.69) | 103.16 (80.17, 129.56) | 8.56 (4.72, 13.24) | 8.41 (4.70, 13.10) | -1.76 (-5.35, 1.93) | 41.87 (19.30, 72.78) | 41.94 (19.39, 72.20) | 0.15 (-8.58, 9.78) | 0.00 (-0.02, 0.02) |
| Egypt | 7.34 (3.24, 13.22) | 15.95 (7.04, 27.98) | 117.24 (99.57, 138.10) | 2.88 (1.50, 4.70) | 2.92 (1.52, 4.81) | 1.40 (-3.24, 6.68) | 16.03 (7.07, 28.55) | 17.19 (7.63, 30.10) | 7.23 (-1.07, 16.60) | 0.23 (0.08, 0.38)* |
| El Salvador | 0.89 (0.41, 1.60) | 1.31 (0.59, 2.32) | 47.47 (27.00, 71.22) | 3.53 (1.89, 5.61) | 3.98 (2.11, 6.38) | 12.74 (1.53, 27.92) | 20.73 (9.58, 36.92) | 20.83 (9.42, 37.26) | 0.49 (-12.24, 16.05) | 0.01 (-0.05, 0.08) |
| Equatorial Guinea | 0.19 (0.09, 0.33) | 0.63 (0.29, 1.13) | 240.18 (208.81, 273.23) | 6.21 (3.31, 9.77) | 6.12 (3.30, 9.55) | -1.36 (-5.50, 2.66) | 60.19 (27.40, 105.42) | 55.83 (25.55, 97.56) | -7.25 (-14.08, 0.41) | -0.26 (-0.29, -0.23)* |
| Eritrea | 0.78 (0.35, 1.37) | 1.96 (0.89, 3.49) | 153.04 (132.68, 174.13) | 5.36 (2.86, 8.55) | 5.49 (2.93, 8.65) | 2.43 (-1.70, 7.12) | 39.32 (18.08, 68.30) | 38.38 (17.71, 66.66) | -2.37 (-9.99, 5.76) | -0.08 (-0.10, -0.06)* |
| Estonia | 0.53 (0.24, 0.95) | 0.34 (0.16, 0.60) | -35.33 (-43.15, -26.46) | 4.13 (2.23, 6.60) | 4.00 (2.16, 6.46) | -3.06 (-7.42, 1.00) | 30.37 (13.62, 54.12) | 21.23 (9.59, 36.88) | -30.10 (-38.21, -21.47) | -1.22 (-1.28, -1.15)* |
| Eswatini | 0.09 (0.04, 0.16) | 0.13 (0.06, 0.23) | 38.70 (14.24, 70.46) | 2.77 (1.47, 4.41) | 2.13 (1.13, 3.45) | -23.02 (-33.33, -10.05) | 15.89 (7.06, 28.38) | 13.26 (6.05, 23.73) | -16.52 (-28.99, 0.17) | -0.59 (-0.67, -0.51)* |
| Ethiopia | 12.87 (5.89, 23.12) | 25.89 (11.86, 45.79) | 101.13 (92.77, 110.10) | 5.47 (2.92, 8.61) | 5.51 (2.96, 8.69) | 0.64 (-1.82, 3.25) | 40.99 (18.79, 74.60) | 36.99 (16.97, 66.86) | -9.76 (-12.98, -6.63) | -0.34 (-0.41, -0.28)* |
| Fiji | 0.09 (0.04, 0.15) | 0.12 (0.05, 0.21) | 36.50 (22.94, 50.69) | 4.02 (2.16, 6.39) | 3.99 (2.14, 6.34) | -0.59 (-3.50, 2.47) | 12.58 (5.70, 22.13) | 12.56 (5.77, 22.26) | -0.14 (-8.09, 7.87) | -0.01 (-0.02, 0.01) |
| Finland | 1.45 (0.64, 2.64) | 1.18 (0.54, 2.06) | -19.06 (-29.07, -8.13) | 3.41 (1.83, 5.58) | 3.35 (1.81, 5.36) | -1.75 (-7.89, 4.62) | 26.28 (11.76, 47.24) | 20.15 (9.04, 35.93) | -23.31 (-31.61, -14.26) | -0.93 (-1.07, -0.79)* |
| France | 10.84 (4.92, 19.38) | 10.59 (4.76, 18.73) | -2.29 (-15.86, 11.48) | 2.48 (1.31, 4.07) | 2.60 (1.39, 4.25) | 5.02 (-2.22, 13.16) | 17.08 (7.76, 30.54) | 14.69 (6.51, 25.98) | -14.03 (-26.15, -1.76) | -0.50 (-0.57, -0.44)* |
| Gabon | 0.45 (0.21, 0.79) | 0.88 (0.40, 1.55) | 96.84 (81.75, 115.36) | 6.24 (3.38, 9.79) | 6.21 (3.37, 9.74) | -0.44 (-3.71, 2.59) | 57.14 (26.17, 101.36) | 54.10 (24.69, 94.75) | -5.31 (-12.27, 2.87) | -0.19 (-0.21, -0.16)* |
| Gambia | 0.59 (0.27, 1.01) | 1.46 (0.65, 2.52) | 147.11 (129.78, 167.19) | 10.09 (5.69, 15.74) | 10.09 (5.69, 15.70) | 0.03 (-2.20, 2.83) | 87.99 (40.94, 150.72) | 87.12 (40.67, 149.99) | -0.99 (-7.88, 6.95) | -0.04 (-0.07, -0.01)* |
| Georgia | 0.41 (0.18, 0.73) | 0.31 (0.13, 0.54) | -25.12 (-31.73, -18.25) | 1.53 (0.80, 2.51) | 1.53 (0.81, 2.50) | 0.09 (-2.96, 3.20) | 6.87 (3.05, 12.14) | 6.93 (3.05, 12.22) | 0.79 (-6.81, 8.05) | 0.03 (-0.01, 0.07) |
| Germany | 27.97 (12.83, 48.00) | 32.54 (15.56, 56.29) | 16.34 (2.12, 32.30) | 7.02 (3.93, 10.96) | 7.10 (3.97, 11.05) | 1.14 (-5.35, 8.68) | 30.85 (13.96, 53.14) | 34.27 (16.00, 59.16) | 11.10 (-2.11, 26.52) | 0.40 (0.27, 0.52)* |
| Ghana | 6.67 (3.06, 11.41) | 16.37 (7.49, 27.96) | 145.39 (128.98, 161.39) | 10.01 (5.65, 15.48) | 9.98 (5.63, 15.56) | -0.32 (-2.27, 1.56) | 62.13 (28.53, 107.23) | 62.10 (28.57, 107.38) | -0.05 (-6.39, 6.08) | -0.00 (-0.02, 0.01) |
| Greece | 4.58 (2.03, 8.12) | 4.83 (2.24, 8.63) | 5.35 (-4.04, 16.66) | 4.91 (2.66, 7.82) | 4.93 (2.66, 7.85) | 0.52 (-1.94, 3.12) | 39.67 (17.66, 70.76) | 40.40 (18.04, 72.69) | 1.84 (-5.47, 11.15) | 0.04 (-0.11, 0.19) |
| Greenland | 0.04 (0.02, 0.08) | 0.04 (0.02, 0.07) | -16.71 (-25.95, -4.34) | 6.18 (3.33, 9.89) | 6.23 (3.41, 9.86) | 0.86 (-2.44, 4.82) | 67.99 (31.33, 120.46) | 61.29 (28.23, 108.12) | -9.86 (-17.59, -2.11) | -0.35 (-0.39, -0.31)* |
| Grenada | 0.01 (0.00, 0.02) | 0.02 (0.01, 0.03) | 55.60 (40.55, 73.30) | 3.19 (1.70, 5.14) | 3.17 (1.68, 5.13) | -0.60 (-3.60, 2.32) | 15.25 (6.70, 27.26) | 15.14 (6.57, 26.87) | -0.71 (-7.62, 6.30) | -0.02 (-0.06, 0.02) |
| Guam | 0.02 (0.01, 0.04) | 0.03 (0.01, 0.05) | 27.17 (12.95, 46.77) | 3.90 (2.12, 6.28) | 3.90 (2.10, 6.28) | 0.07 (-2.99, 2.92) | 14.65 (6.60, 26.44) | 14.80 (6.78, 26.09) | 0.98 (-6.53, 9.16) | 0.03 (0.00, 0.06)* |
| Guatemala | 1.85 (0.84, 3.27) | 4.41 (1.95, 7.88) | 138.53 (108.43, 170.59) | 5.41 (2.90, 8.61) | 4.88 (2.62, 7.70) | -9.82 (-19.35, -1.79) | 32.34 (14.62, 57.58) | 28.03 (12.55, 50.02) | -13.33 (-23.87, -2.21) | -0.47 (-0.58, -0.36)* |
| Guinea | 2.68 (1.22, 4.56) | 5.62 (2.60, 9.65) | 109.88 (94.10, 126.32) | 10.09 (5.68, 15.60) | 10.09 (5.68, 15.58) | 0.00 (-2.26, 2.24) | 60.03 (28.02, 103.44) | 62.95 (29.45, 109.07) | 4.87 (-2.92, 13.54) | 0.16 (0.15, 0.18)* |
| Guinea-Bissau | 0.43 (0.19, 0.73) | 0.92 (0.41, 1.60) | 114.14 (99.51, 129.83) | 10.12 (5.71, 15.64) | 10.07 (5.71, 15.64) | -0.52 (-2.73, 1.86) | 62.60 (29.05, 109.06) | 65.24 (29.85, 112.97) | 4.21 (-3.18, 12.27) | 0.14 (0.13, 0.16)* |
| Guyana | 0.17 (0.07, 0.31) | 0.21 (0.10, 0.38) | 26.53 (13.73, 41.08) | 3.25 (1.72, 5.26) | 3.26 (1.72, 5.24) | 0.28 (-2.44, 3.52) | 24.58 (10.89, 44.16) | 26.91 (12.10, 47.38) | 9.47 (0.25, 18.75) | 0.32 (0.26, 0.38)* |
| Haiti | 0.97 (0.42, 1.75) | 2.09 (0.89, 3.81) | 114.30 (97.65, 131.39) | 3.27 (1.71, 5.39) | 3.25 (1.72, 5.29) | -0.50 (-3.16, 2.14) | 18.81 (8.16, 33.70) | 18.06 (7.66, 32.70) | -3.98 (-10.63, 3.13) | -0.14 (-0.16, -0.11)* |
| Honduras | 0.87 (0.38, 1.51) | 2.40 (1.05, 4.14) | 176.60 (155.01, 200.19) | 5.96 (3.18, 9.54) | 5.96 (3.25, 9.54) | -0.06 (-2.56, 2.71) | 26.51 (11.91, 46.35) | 27.88 (12.41, 48.95) | 5.18 (-2.54, 13.12) | 0.13 (-0.26, 0.52) |
| Hungary | 2.14 (0.96, 3.81) | 1.60 (0.73, 2.82) | -25.13 (-33.35, -14.92) | 3.90 (2.08, 6.25) | 3.81 (2.06, 6.12) | -2.22 (-6.74, 2.31) | 18.35 (8.33, 32.63) | 13.28 (6.05, 23.36) | -27.62 (-34.77, -19.33) | -1.11 (-1.15, -1.06)* |
| Iceland | 0.05 (0.02, 0.08) | 0.05 (0.02, 0.10) | 15.72 (-3.52, 37.06) | 4.15 (2.22, 6.58) | 3.85 (2.04, 6.23) | -7.23 (-19.34, 4.35) | 18.02 (7.96, 31.93) | 14.52 (6.49, 25.37) | -19.41 (-31.51, -5.99) | -0.74 (-0.83, -0.66)* |
| India | 349.83 (163.79, 611.31) | 594.73 (280.99, 1031.97) | 70.01 (57.63, 84.67) | 8.84 (4.89, 13.75) | 8.66 (4.76, 13.33) | -2.03 (-7.12, 3.38) | 50.75 (24.33, 87.55) | 43.53 (20.42, 75.71) | -14.23 (-19.57, -8.65) | -0.55 (-0.67, -0.43)* |
| Indonesia | 13.44 (5.98, 24.30) | 23.31 (10.35, 42.35) | 73.45 (61.44, 86.43) | 3.81 (2.02, 6.13) | 3.84 (2.04, 6.22) | 0.61 (-0.70, 2.21) | 8.36 (3.77, 15.14) | 8.36 (3.76, 15.14) | -0.02 (-2.38, 2.55) | -0.01 (-0.03, 0.02) |
| Iran (Islamic Republic of) | 10.72 (4.61, 19.44) | 25.05 (10.93, 44.90) | 133.71 (105.12, 161.69) | 3.28 (1.72, 5.29) | 3.33 (1.75, 5.36) | 1.43 (-0.35, 3.45) | 24.32 (10.61, 43.57) | 26.05 (11.32, 46.61) | 7.08 (4.38, 9.85) | 0.26 (0.19, 0.32)* |
| Iraq | 2.24 (0.98, 3.96) | 7.00 (3.16, 12.62) | 212.53 (185.16, 243.35) | 3.01 (1.56, 4.97) | 3.04 (1.56, 4.97) | 0.73 (-4.23, 5.45) | 17.44 (7.76, 30.81) | 17.69 (8.05, 31.74) | 1.42 (-7.05, 10.79) | 0.06 (-0.00, 0.12) |
| Ireland | 0.97 (0.43, 1.71) | 1.52 (0.70, 2.66) | 56.63 (42.18, 74.86) | 4.48 (2.44, 7.11) | 4.47 (2.43, 7.11) | -0.15 (-4.08, 3.61) | 26.97 (12.12, 48.01) | 28.53 (12.85, 50.51) | 5.78 (-3.84, 16.19) | 0.21 (0.12, 0.32)* |
| Israel | 1.60 (0.71, 2.82) | 2.93 (1.30, 5.12) | 83.33 (68.01, 101.10) | 4.95 (2.68, 7.78) | 5.03 (2.73, 8.00) | 1.69 (-1.33, 5.17) | 33.48 (15.10, 58.99) | 31.42 (13.86, 55.20) | -6.14 (-13.71, 2.00) | -0.24 (-0.30, -0.19)* |
| Italy | 19.96 (9.01, 34.70) | 20.47 (9.25, 36.29) | 2.57 (-4.44, 9.34) | 5.59 (3.03, 8.90) | 5.64 (3.06, 8.98) | 0.84 (-0.39, 2.07) | 30.72 (14.04, 53.80) | 29.20 (13.07, 51.19) | -4.97 (-7.02, -2.94) | -0.21 (-0.36, -0.07)* |
| Jamaica | 0.29 (0.13, 0.52) | 0.43 (0.19, 0.77) | 48.11 (32.96, 64.83) | 3.21 (1.71, 5.25) | 3.20 (1.70, 5.24) | -0.19 (-2.90, 2.64) | 14.02 (6.05, 24.86) | 14.17 (6.27, 25.13) | 1.04 (-5.74, 8.85) | 0.03 (-0.02, 0.08) |
| Japan | 23.53 (10.86, 41.30) | 26.67 (12.20, 46.82) | 13.34 (6.58, 20.60) | 5.50 (2.96, 8.71) | 5.87 (3.18, 9.32) | 6.77 (2.41, 11.54) | 16.19 (7.44, 28.38) | 18.64 (8.43, 32.67) | 15.16 (10.00, 20.87) | 0.51 (0.38, 0.65)* |
| Jordan | 0.56 (0.24, 1.00) | 2.05 (0.90, 3.65) | 267.34 (227.61, 315.21) | 2.81 (1.48, 4.64) | 2.87 (1.50, 4.68) | 2.15 (-2.47, 7.94) | 20.19 (8.94, 35.38) | 18.26 (8.11, 32.02) | -9.58 (-16.88, -1.14) | -0.36 (-0.40, -0.32)* |
| Kazakhstan | 1.16 (0.52, 2.05) | 1.42 (0.63, 2.52) | 22.34 (10.86, 34.97) | 1.55 (0.81, 2.53) | 1.57 (0.83, 2.57) | 1.33 (-2.09, 5.19) | 7.52 (3.39, 13.49) | 7.47 (3.33, 13.26) | -0.75 (-9.69, 8.79) | -0.00 (-0.08, 0.07) |
| Kenya | 10.24 (4.86, 17.34) | 26.46 (12.56, 44.60) | 158.33 (146.45, 171.11) | 9.89 (5.54, 15.13) | 10.03 (5.61, 15.36) | 1.38 (-2.21, 5.65) | 72.98 (35.00, 125.96) | 68.34 (32.51, 116.62) | -6.36 (-9.88, -2.25) | -0.23 (-0.26, -0.20)* |
| Kiribati | 0.02 (0.01, 0.03) | 0.03 (0.01, 0.05) | 55.80 (40.15, 73.55) | 7.29 (4.04, 11.38) | 7.32 (4.02, 11.39) | 0.41 (-4.18, 5.12) | 25.98 (11.87, 45.46) | 23.57 (10.84, 41.52) | -9.27 (-17.57, -0.22) | -0.35 (-0.37, -0.33)* |
| Kuwait | 0.34 (0.15, 0.62) | 1.08 (0.47, 2.01) | 220.57 (177.42, 271.63) | 3.30 (1.68, 5.44) | 3.13 (1.63, 5.13) | -5.17 (-10.44, -0.23) | 19.60 (8.80, 35.38) | 19.31 (8.63, 34.88) | -1.48 (-10.05, 8.45) | -0.05 (-0.07, -0.04)* |
| Kyrgyzstan | 0.30 (0.13, 0.54) | 0.45 (0.20, 0.81) | 48.85 (34.32, 64.60) | 1.55 (0.83, 2.52) | 1.56 (0.83, 2.55) | 0.77 (-2.47, 4.12) | 8.07 (3.65, 14.31) | 7.30 (3.20, 13.13) | -9.59 (-17.59, -0.90) | -0.35 (-0.37, -0.32)* |
| Lao People's Democratic Republic | 0.40 (0.18, 0.74) | 0.78 (0.35, 1.46) | 94.00 (75.36, 115.47) | 3.85 (1.99, 6.29) | 3.95 (2.04, 6.44) | 2.77 (-1.62, 7.44) | 12.34 (5.48, 22.43) | 11.16 (4.99, 20.24) | -9.58 (-18.07, -0.32) | -0.36 (-0.43, -0.29)* |
| Latvia | 0.81 (0.38, 1.45) | 0.51 (0.24, 0.91) | -36.91 (-43.82, -28.99) | 4.06 (2.19, 6.53) | 4.05 (2.19, 6.50) | -0.41 (-4.52, 3.54) | 27.04 (12.35, 48.17) | 21.89 (9.86, 38.72) | -19.05 (-26.80, -9.00) | -0.73 (-0.80, -0.65)* |
| Lebanon | 0.57 (0.26, 1.00) | 1.14 (0.50, 2.00) | 100.22 (79.61, 123.16) | 2.97 (1.53, 4.80) | 2.88 (1.51, 4.64) | -3.21 (-7.85, 1.20) | 20.44 (9.30, 35.68) | 21.07 (9.21, 36.80) | 3.05 (-7.37, 13.73) | 0.12 (0.04, 0.20)* |
| Lesotho | 0.46 (0.21, 0.83) | 0.61 (0.28, 1.04) | 31.34 (16.23, 47.12) | 3.73 (1.98, 5.96) | 3.64 (1.93, 5.78) | -2.47 (-6.87, 1.59) | 31.64 (14.35, 55.40) | 30.92 (14.08, 53.12) | -2.28 (-12.02, 7.59) | -0.08 (-0.11, -0.05)* |
| Liberia | 0.96 (0.44, 1.65) | 2.53 (1.13, 4.37) | 164.50 (141.91, 189.11) | 10.19 (5.77, 15.83) | 10.11 (5.68, 15.71) | -0.87 (-3.47, 1.64) | 66.90 (31.39, 116.18) | 66.45 (30.91, 116.64) | -0.68 (-8.92, 8.33) | 0.01 (-0.04, 0.06) |
| Libya | 0.64 (0.28, 1.14) | 1.64 (0.73, 2.93) | 155.57 (125.77, 189.74) | 3.13 (1.62, 5.07) | 3.07 (1.60, 5.03) | -2.03 (-6.53, 2.13) | 20.44 (9.15, 36.08) | 20.98 (9.34, 36.83) | 2.64 (-4.35, 11.61) | 0.09 (0.05, 0.12)* |
| Lithuania | 1.07 (0.49, 1.93) | 0.82 (0.39, 1.45) | -23.72 (-32.28, -12.98) | 4.15 (2.25, 6.69) | 4.07 (2.21, 6.50) | -1.96 (-6.23, 1.74) | 26.81 (12.03, 47.67) | 24.14 (11.01, 42.93) | -9.97 (-18.52, -0.39) | -0.35 (-0.39, -0.32)* |
| Luxembourg | 0.12 (0.06, 0.22) | 0.15 (0.07, 0.27) | 25.03 (12.90, 38.47) | 4.98 (2.68, 7.94) | 4.94 (2.68, 7.88) | -0.92 (-4.16, 2.05) | 28.06 (12.71, 49.99) | 21.40 (9.49, 37.64) | -23.75 (-31.15, -16.08) | -0.94 (-0.97, -0.90)* |
| Madagascar | 3.19 (1.47, 5.65) | 7.55 (3.43, 13.35) | 136.91 (117.57, 155.71) | 5.66 (3.04, 9.00) | 5.65 (3.03, 8.92) | -0.31 (-4.23, 3.68) | 39.93 (18.24, 69.74) | 38.47 (17.54, 67.74) | -3.64 (-10.96, 4.20) | -0.13 (-0.14, -0.12)* |
| Malawi | 1.98 (0.91, 3.47) | 3.78 (1.72, 6.62) | 90.75 (76.02, 107.86) | 5.37 (2.90, 8.51) | 5.38 (2.87, 8.43) | 0.17 (-4.20, 5.00) | 30.92 (14.30, 53.75) | 29.18 (13.41, 50.56) | -5.63 (-12.25, 2.42) | -0.21 (-0.23, -0.18)* |
| Malaysia | 3.27 (1.47, 5.83) | 8.56 (3.80, 15.05) | 161.86 (136.35, 195.17) | 5.53 (3.00, 8.77) | 5.41 (2.95, 8.51) | -2.13 (-7.36, 3.23) | 21.68 (9.82, 38.34) | 25.81 (11.45, 45.58) | 19.05 (7.12, 34.09) | 0.56 (0.33, 0.79)* |
| Maldives | 0.03 (0.01, 0.05) | 0.07 (0.03, 0.13) | 176.13 (131.47, 228.12) | 3.50 (1.87, 5.60) | 3.79 (1.96, 6.19) | 8.37 (0.31, 16.73) | 15.88 (7.27, 28.24) | 12.46 (5.63, 22.27) | -21.52 (-29.29, -12.80) | -0.84 (-0.88, -0.80)* |
| Mali | 2.81 (1.29, 4.79) | 6.71 (3.10, 11.54) | 138.91 (117.68, 159.41) | 10.07 (5.70, 15.58) | 10.03 (5.65, 15.56) | -0.35 (-2.60, 1.89) | 46.48 (21.76, 80.19) | 45.33 (20.68, 78.73) | -2.47 (-10.83, 5.43) | -0.09 (-0.14, -0.04)* |
| Malta | 0.08 (0.04, 0.15) | 0.10 (0.05, 0.18) | 19.63 (9.70, 32.56) | 4.94 (2.68, 7.84) | 4.95 (2.66, 7.86) | 0.04 (-2.37, 2.42) | 21.18 (9.57, 37.57) | 20.63 (9.23, 36.07) | -2.63 (-9.47, 4.53) | -0.10 (-0.14, -0.05)* |
| Marshall Islands | 0.00 (0.00, 0.01) | 0.01 (0.00, 0.01) | 58.63 (43.05, 79.90) | 3.96 (2.14, 6.30) | 4.00 (2.16, 6.33) | 1.02 (-1.74, 4.20) | 13.01 (5.82, 23.20) | 12.38 (5.57, 21.92) | -4.83 (-11.12, 2.67) | -0.17 (-0.19, -0.15)* |
| Mauritania | 0.73 (0.34, 1.27) | 1.47 (0.67, 2.52) | 102.34 (86.92, 120.82) | 9.96 (5.62, 15.33) | 9.96 (5.62, 15.47) | -0.00 (-2.38, 2.38) | 49.67 (23.28, 85.90) | 47.51 (21.77, 81.05) | -4.34 (-11.69, 4.20) | -0.16 (-0.20, -0.12)* |
| Mauritius | 0.21 (0.09, 0.39) | 0.25 (0.11, 0.43) | 16.94 (2.24, 35.01) | 3.28 (1.77, 5.29) | 3.33 (1.75, 5.40) | 1.41 (-3.02, 6.79) | 19.24 (8.69, 34.55) | 16.72 (7.61, 29.66) | -13.09 (-19.75, -5.26) | -0.48 (-0.54, -0.42)* |
| Mexico | 12.94 (5.67, 22.58) | 34.92 (15.67, 61.80) | 169.89 (145.29, 191.97) | 4.33 (2.30, 7.08) | 4.89 (2.62, 8.06) | 12.90 (6.05, 20.92) | 19.08 (8.52, 33.82) | 26.63 (11.97, 47.14) | 39.53 (29.66, 49.68) | 1.17 (1.08, 1.26)* |
| Micronesia (Federated States of) | 0.01 (0.00, 0.02) | 0.01 (0.01, 0.02) | 15.96 (3.79, 30.98) | 3.98 (2.14, 6.37) | 3.98 (2.15, 6.38) | 0.16 (-2.60, 3.48) | 13.49 (6.06, 24.17) | 12.51 (5.80, 22.41) | -7.31 (-14.88, 1.20) | -0.27 (-0.29, -0.24)* |
| Monaco | 0.01 (0.00, 0.02) | 0.01 (0.01, 0.02) | 18.20 (10.28, 28.60) | 4.97 (2.70, 7.97) | 4.98 (2.71, 7.97) | 0.23 (-1.60, 2.33) | 30.56 (12.98, 55.38) | 30.43 (12.98, 54.07) | -0.42 (-6.22, 5.16) | -0.01 (-0.03, 0.00) |
| Mongolia | 0.16 (0.07, 0.29) | 0.32 (0.14, 0.57) | 100.93 (71.69, 133.44) | 1.59 (0.84, 2.60) | 1.58 (0.84, 2.61) | -0.31 (-3.36, 2.73) | 9.42 (4.18, 17.00) | 9.35 (4.15, 16.75) | -0.73 (-7.79, 7.08) | -0.02 (-0.06, 0.01) |
| Montenegro | 0.09 (0.04, 0.16) | 0.10 (0.04, 0.17) | 8.72 (-2.86, 21.43) | 3.75 (2.00, 5.96) | 3.79 (2.04, 6.08) | 0.92 (-2.33, 4.48) | 13.48 (6.04, 24.02) | 13.21 (5.83, 23.59) | -1.98 (-9.77, 6.26) | -0.06 (-0.08, -0.04)* |
| Morocco | 5.31 (2.35, 9.30) | 9.52 (4.26, 16.93) | 79.50 (61.22, 102.45) | 3.01 (1.58, 4.86) | 3.04 (1.59, 4.99) | 0.84 (-3.95, 5.94) | 25.57 (11.51, 45.09) | 25.08 (11.21, 44.44) | -1.90 (-10.34, 7.14) | -0.05 (-0.10, -0.01)* |
| Mozambique | 2.61 (1.16, 4.65) | 6.05 (2.66, 10.84) | 132.00 (106.38, 155.95) | 4.31 (2.28, 6.90) | 4.39 (2.35, 7.14) | 1.99 (-4.60, 9.21) | 29.65 (13.16, 53.24) | 31.76 (14.30, 55.70) | 7.12 (-3.78, 18.33) | 0.23 (0.21, 0.26)* |
| Myanmar | 2.13 (0.94, 3.92) | 3.26 (1.43, 5.76) | 52.87 (35.48, 71.26) | 3.69 (1.94, 5.96) | 3.61 (1.90, 5.83) | -2.36 (-6.99, 1.96) | 6.05 (2.67, 10.90) | 5.81 (2.55, 10.38) | -3.88 (-12.40, 5.41) | -0.14 (-0.20, -0.09)* |
| Namibia | 0.15 (0.07, 0.26) | 0.28 (0.12, 0.50) | 89.20 (72.18, 108.44) | 2.84 (1.49, 4.53) | 2.82 (1.48, 4.48) | -0.57 (-3.20, 1.92) | 13.39 (5.99, 23.58) | 13.03 (5.87, 23.43) | -2.68 (-9.70, 5.27) | -0.09 (-0.18, 0.01) |
| Nauru | 0.00 (0.00, 0.00) | 0.00 (0.00, 0.00) | 15.32 (7.01, 23.64) | 4.03 (2.15, 6.40) | 4.06 (2.18, 6.49) | 0.75 (-1.78, 3.67) | 14.53 (6.21, 26.81) | 14.71 (6.23, 26.51) | 1.24 (-5.32, 8.34) | 0.04 (0.02, 0.05)* |
| Nepal | 5.17 (2.32, 9.24) | 11.13 (5.07, 19.87) | 115.33 (95.16, 138.30) | 5.29 (2.86, 8.44) | 5.38 (2.90, 8.57) | 1.64 (-1.66, 5.58) | 37.22 (16.47, 66.49) | 40.64 (18.39, 72.24) | 9.16 (0.11, 19.22) | 0.30 (0.23, 0.37)* |
| Netherlands | 7.47 (3.48, 13.15) | 6.88 (3.38, 11.67) | -7.93 (-30.79, 19.88) | 8.32 (4.63, 12.91) | 6.94 (3.87, 10.41) | -16.66 (-34.13, 4.00) | 44.48 (20.70, 77.30) | 35.56 (17.59, 60.30) | -20.06 (-39.63, 3.62) | -0.73 (-1.03, -0.44)* |
| New Zealand | 1.96 (0.88, 3.32) | 2.99 (1.43, 4.95) | 52.43 (37.28, 70.71) | 10.51 (5.94, 16.16) | 12.22 (6.85, 18.55) | 16.24 (7.08, 25.17) | 54.26 (24.38, 91.86) | 67.61 (32.08, 112.39) | 24.61 (12.14, 39.04) | 0.74 (0.62, 0.87)* |
| Nicaragua | 0.63 (0.28, 1.11) | 1.27 (0.56, 2.21) | 100.65 (75.78, 129.14) | 4.62 (2.48, 7.51) | 4.09 (2.19, 6.48) | -11.47 (-19.77, -4.47) | 22.39 (9.94, 39.65) | 20.16 (8.85, 35.26) | -9.94 (-20.29, 1.66) | -0.40 (-0.54, -0.26)* |
| Niger | 3.01 (1.38, 5.15) | 8.52 (3.88, 14.58) | 183.27 (162.62, 208.02) | 10.05 (5.66, 15.55) | 10.06 (5.66, 15.48) | 0.11 (-2.19, 2.84) | 58.26 (26.86, 100.78) | 60.17 (28.14, 102.98) | 3.27 (-4.16, 11.26) | 0.11 (0.09, 0.13)* |
| Nigeria | 45.17 (21.02, 77.19) | 87.09 (40.77, 148.80) | 92.80 (86.58, 99.19) | 11.43 (6.45, 17.48) | 11.32 (6.37, 17.35) | -0.93 (-4.47, 2.87) | 68.94 (32.52, 117.75) | 57.74 (27.17, 96.35) | -16.23 (-19.23, -12.74) | -0.57 (-0.71, -0.43)* |
| Niue | 0.00 (0.00, 0.00) | 0.00 (0.00, 0.00) | -14.45 (-21.25, -7.24) | 4.03 (2.16, 6.42) | 4.03 (2.16, 6.42) | 0.00 (-2.30, 2.51) | 14.68 (6.39, 26.43) | 14.57 (6.28, 26.08) | -0.75 (-6.87, 5.79) | -0.03 (-0.05, -0.01)* |
| North Macedonia | 0.25 (0.11, 0.44) | 0.28 (0.13, 0.49) | 12.33 (-0.81, 27.19) | 3.63 (1.96, 5.77) | 3.60 (1.90, 5.81) | -0.93 (-7.98, 5.51) | 11.86 (5.35, 21.19) | 10.71 (4.97, 19.08) | -9.67 (-19.15, 0.16) | -0.35 (-0.39, -0.31)* |
| Northern Mariana Islands | 0.01 (0.00, 0.01) | 0.01 (0.00, 0.01) | 8.81 (-11.23, 40.23) | 3.88 (2.08, 6.23) | 3.92 (2.12, 6.24) | 1.02 (-2.90, 5.39) | 10.63 (4.82, 18.86) | 11.42 (5.26, 20.36) | 7.41 (-1.22, 17.26) | 0.25 (0.15, 0.34)* |
| Norway | 0.84 (0.38, 1.45) | 1.52 (0.67, 2.61) | 81.39 (67.92, 95.91) | 4.70 (2.61, 7.40) | 5.91 (3.22, 9.27) | 25.69 (17.61, 35.34) | 18.21 (8.25, 31.65) | 26.38 (11.46, 45.89) | 44.85 (35.31, 56.40) | 1.26 (0.97, 1.55)* |
| Oman | 0.32 (0.14, 0.59) | 1.12 (0.48, 2.13) | 249.13 (211.29, 293.29) | 3.37 (1.71, 5.62) | 3.45 (1.77, 5.74) | 2.47 (-2.07, 7.35) | 20.21 (8.89, 37.32) | 20.87 (9.42, 38.03) | 3.27 (-5.61, 13.91) | 0.10 (-0.00, 0.20) |
| Pakistan | 27.02 (12.05, 47.99) | 58.07 (26.13, 103.69) | 114.90 (105.57, 123.91) | 6.03 (3.21, 9.49) | 6.04 (3.22, 9.50) | 0.19 (-1.55, 1.92) | 35.48 (16.04, 62.49) | 34.02 (15.38, 60.14) | -4.14 (-7.47, -0.51) | -0.15 (-0.19, -0.10)* |
| Palau | 0.00 (0.00, 0.00) | 0.00 (0.00, 0.01) | 40.44 (19.46, 67.80) | 4.04 (2.15, 6.45) | 4.02 (2.15, 6.47) | -0.42 (-4.09, 3.20) | 14.61 (6.13, 26.28) | 14.37 (6.13, 26.07) | -1.59 (-7.89, 5.49) | -0.06 (-0.08, -0.04)* |
| Palestine | 0.46 (0.20, 0.83) | 1.44 (0.63, 2.62) | 212.53 (183.82, 244.26) | 3.09 (1.63, 5.03) | 3.23 (1.67, 5.31) | 4.44 (-0.40, 10.99) | 33.42 (14.92, 59.24) | 34.26 (15.02, 61.73) | 2.51 (-5.74, 11.42) | 0.08 (-0.02, 0.19) |
| Panama | 0.49 (0.22, 0.86) | 0.97 (0.44, 1.70) | 98.45 (81.63, 121.20) | 5.38 (2.92, 8.50) | 5.38 (2.91, 8.55) | -0.02 (-2.10, 2.29) | 23.40 (10.61, 41.16) | 23.00 (10.31, 40.22) | -1.72 (-8.53, 5.15) | -0.06 (-0.07, -0.04)* |
| Papua New Guinea | 0.51 (0.22, 0.89) | 1.24 (0.56, 2.19) | 143.68 (125.32, 162.18) | 3.99 (2.16, 6.35) | 4.00 (2.16, 6.36) | 0.16 (-2.61, 3.19) | 14.83 (6.66, 25.85) | 13.94 (6.26, 24.50) | -5.99 (-12.21, 0.31) | -0.21 (-0.22, -0.20)* |
| Paraguay | 0.55 (0.24, 0.99) | 1.23 (0.54, 2.21) | 123.05 (100.61, 145.66) | 3.04 (1.59, 4.96) | 3.04 (1.58, 4.93) | -0.12 (-3.45, 3.51) | 16.10 (7.02, 28.74) | 17.41 (7.62, 31.27) | 8.12 (-0.89, 16.98) | 0.25 (0.16, 0.35)* |
| Peru | 4.64 (2.16, 7.92) | 7.84 (3.50, 13.58) | 69.12 (38.84, 107.28) | 8.28 (4.61, 12.65) | 8.49 (4.72, 13.07) | 2.57 (-13.25, 22.95) | 25.41 (11.99, 44.16) | 22.50 (10.09, 38.88) | -11.45 (-26.68, 7.72) | -0.39 (-0.60, -0.18)* |
| Philippines | 6.01 (2.66, 11.06) | 9.80 (4.36, 18.01) | 63.02 (54.67, 73.02) | 3.35 (1.75, 5.53) | 3.15 (1.66, 5.18) | -5.74 (-7.37, -4.19) | 11.92 (5.38, 21.71) | 9.22 (4.19, 16.94) | -22.65 (-24.40, -20.88) | -0.88 (-0.94, -0.82)* |
| Poland | 3.50 (1.58, 6.21) | 4.12 (1.87, 7.54) | 17.79 (10.59, 26.38) | 3.58 (1.91, 5.74) | 3.68 (1.95, 5.85) | 2.80 (0.52, 5.23) | 8.59 (3.87, 15.32) | 8.90 (4.03, 16.01) | 3.58 (0.53, 6.68) | 0.11 (0.02, 0.20)* |
| Portugal | 4.32 (1.98, 7.55) | 4.30 (1.96, 7.70) | -0.46 (-12.83, 13.17) | 4.98 (2.70, 7.77) | 4.98 (2.68, 7.93) | 0.03 (-3.88, 4.15) | 39.27 (17.85, 68.95) | 33.83 (15.23, 59.51) | -13.86 (-22.41, -4.90) | -0.50 (-0.66, -0.34)* |
| Puerto Rico | 0.49 (0.22, 0.86) | 0.52 (0.23, 0.93) | 6.18 (-4.49, 20.46) | 3.18 (1.68, 5.11) | 3.18 (1.68, 5.11) | -0.12 (-3.20, 3.13) | 13.49 (5.95, 23.61) | 12.82 (5.56, 22.87) | -4.98 (-12.62, 3.34) | -0.18 (-0.24, -0.12)* |
| Qatar | 0.12 (0.05, 0.24) | 0.91 (0.39, 1.76) | 633.97 (550.31, 727.81) | 3.75 (1.93, 6.25) | 3.89 (1.97, 6.55) | 3.78 (-1.08, 9.01) | 24.75 (11.17, 45.57) | 23.75 (10.48, 43.68) | -4.04 (-13.38, 6.49) | -0.14 (-0.17, -0.11)* |
| Republic of Korea | 6.34 (2.84, 11.17) | 9.68 (4.42, 17.18) | 52.68 (29.83, 79.99) | 5.23 (2.86, 8.44) | 5.06 (2.74, 8.13) | -3.30 (-6.89, 0.24) | 13.67 (6.20, 23.77) | 14.66 (6.64, 25.68) | 7.29 (-2.60, 16.87) | 0.26 (0.21, 0.32)* |
| Republic of Moldova | 0.99 (0.45, 1.78) | 0.80 (0.36, 1.44) | -19.77 (-29.45, -9.16) | 4.15 (2.23, 6.70) | 4.06 (2.17, 6.55) | -2.35 (-7.08, 1.83) | 21.87 (9.83, 39.03) | 17.48 (7.82, 31.50) | -20.07 (-28.74, -11.13) | -0.76 (-0.82, -0.70)* |
| Romania | 3.14 (1.41, 5.59) | 2.78 (1.24, 4.94) | -11.36 (-20.65, -0.22) | 3.83 (2.05, 6.18) | 3.84 (2.08, 6.14) | 0.34 (-3.40, 4.37) | 12.53 (5.66, 22.23) | 12.10 (5.35, 21.42) | -3.41 (-12.25, 5.73) | -0.12 (-0.14, -0.10)* |
| Russian Federation | 33.17 (14.68, 58.64) | 31.85 (14.18, 57.21) | -3.96 (-9.69, 1.20) | 4.62 (2.48, 7.42) | 4.61 (2.47, 7.38) | -0.19 (-6.35, 4.97) | 20.10 (8.91, 35.07) | 18.70 (8.21, 33.10) | -6.98 (-13.17, -2.06) | -0.24 (-0.29, -0.20)* |
| Rwanda | 2.22 (1.01, 3.92) | 3.92 (1.78, 6.99) | 76.55 (63.71, 89.97) | 5.26 (2.85, 8.32) | 5.21 (2.80, 8.11) | -0.99 (-5.05, 3.21) | 46.96 (21.63, 82.20) | 39.19 (17.89, 67.46) | -16.54 (-22.29, -10.51) | -0.63 (-0.69, -0.57)* |
| Saint Kitts and Nevis | 0.01 (0.00, 0.01) | 0.01 (0.01, 0.02) | 85.31 (62.91, 113.95) | 3.20 (1.71, 5.24) | 3.20 (1.71, 5.23) | -0.06 (-2.05, 2.05) | 20.11 (8.46, 36.10) | 19.92 (8.43, 35.47) | -0.94 (-5.71, 4.48) | -0.03 (-0.05, -0.01)* |
| Saint Lucia | 0.02 (0.01, 0.03) | 0.03 (0.01, 0.06) | 76.12 (53.79, 103.52) | 3.18 (1.69, 5.14) | 3.17 (1.69, 5.09) | -0.30 (-3.07, 2.27) | 15.46 (6.83, 27.70) | 15.66 (6.85, 27.53) | 1.30 (-5.63, 8.51) | 0.04 (0.00, 0.08)* |
| Saint Vincent and the Grenadines | 0.01 (0.01, 0.03) | 0.02 (0.01, 0.04) | 38.92 (21.25, 60.37) | 3.18 (1.68, 5.11) | 3.16 (1.66, 5.10) | -0.40 (-3.23, 2.40) | 15.35 (6.66, 27.11) | 15.98 (6.93, 28.45) | 4.09 (-3.40, 12.12) | 0.14 (0.09, 0.18)* |
| Samoa | 0.01 (0.00, 0.02) | 0.01 (0.01, 0.03) | 32.25 (17.71, 49.25) | 2.52 (1.30, 4.16) | 2.62 (1.37, 4.26) | 4.02 (-3.34, 12.85) | 8.34 (3.65, 15.27) | 7.71 (3.48, 13.74) | -7.54 (-16.78, 3.06) | -0.28 (-0.35, -0.21)* |
| San Marino | 0.01 (0.00, 0.01) | 0.01 (0.00, 0.02) | 46.97 (34.86, 60.32) | 4.96 (2.69, 7.85) | 5.01 (2.71, 7.99) | 1.01 (-1.34, 4.07) | 30.29 (12.79, 54.88) | 30.93 (13.34, 55.01) | 2.09 (-3.94, 8.19) | 0.07 (0.07, 0.08)* |
| Sao Tome and Principe | 0.04 (0.02, 0.07) | 0.09 (0.04, 0.15) | 105.45 (88.68, 123.73) | 10.06 (5.72, 15.59) | 10.03 (5.67, 15.58) | -0.24 (-2.50, 1.93) | 48.76 (22.13, 83.87) | 49.09 (22.27, 85.20) | 0.69 (-5.71, 7.79) | 0.02 (-0.01, 0.05) |
| Saudi Arabia | 2.24 (0.98, 4.19) | 8.28 (3.64, 15.51) | 269.39 (215.93, 324.36) | 2.96 (1.53, 4.95) | 2.97 (1.51, 4.90) | 0.19 (-6.22, 6.72) | 17.71 (7.83, 31.92) | 18.89 (8.56, 34.30) | 6.67 (-4.81, 18.74) | 0.21 (0.16, 0.26)* |
| Senegal | 2.77 (1.27, 4.81) | 6.33 (2.97, 11.05) | 128.90 (111.61, 147.67) | 10.01 (5.71, 15.39) | 9.98 (5.61, 15.41) | -0.30 (-2.50, 2.01) | 54.53 (25.01, 95.12) | 55.05 (25.45, 94.64) | 0.96 (-6.41, 9.28) | 0.03 (-0.02, 0.08) |
| Serbia | 1.29 (0.59, 2.32) | 1.17 (0.54, 2.04) | -9.51 (-19.89, 2.29) | 3.08 (1.64, 4.88) | 3.25 (1.72, 5.29) | 5.34 (-3.94, 16.93) | 12.55 (5.70, 22.52) | 11.28 (5.00, 19.74) | -10.17 (-19.71, 1.59) | -0.37 (-0.41, -0.33)* |
| Seychelles | 0.01 (0.00, 0.01) | 0.01 (0.01, 0.02) | 50.50 (32.17, 71.04) | 3.85 (1.99, 6.36) | 3.82 (1.97, 6.28) | -0.78 (-5.11, 3.53) | 11.38 (5.03, 20.58) | 10.06 (4.46, 18.40) | -11.60 (-19.40, -2.48) | -0.43 (-0.46, -0.39)* |
| Sierra Leone | 1.59 (0.74, 2.72) | 3.99 (1.83, 6.76) | 151.49 (132.02, 174.54) | 10.06 (5.67, 15.60) | 10.05 (5.68, 15.53) | -0.16 (-2.16, 1.92) | 58.51 (26.63, 101.60) | 63.20 (29.62, 108.88) | 8.03 (0.24, 17.85) | 0.27 (0.25, 0.29)* |
| Singapore | 0.84 (0.39, 1.48) | 1.03 (0.47, 1.82) | 22.65 (7.66, 40.26) | 5.29 (2.88, 8.54) | 5.01 (2.73, 7.94) | -5.38 (-11.64, 0.31) | 24.66 (11.46, 42.77) | 14.96 (6.74, 26.35) | -39.33 (-46.58, -31.24) | -1.73 (-1.85, -1.60)* |
| Slovakia | 0.78 (0.35, 1.38) | 0.80 (0.36, 1.41) | 2.31 (-7.41, 14.23) | 3.79 (2.04, 6.12) | 3.75 (2.01, 6.06) | -0.92 (-4.37, 2.59) | 14.00 (6.25, 24.87) | 12.11 (5.40, 21.16) | -13.52 (-21.04, -5.10) | -0.50 (-0.52, -0.48)* |
| Slovenia | 0.46 (0.21, 0.81) | 0.39 (0.18, 0.69) | -14.44 (-24.51, -0.77) | 3.83 (2.03, 6.14) | 3.80 (2.04, 6.10) | -0.82 (-4.55, 2.89) | 20.99 (9.50, 36.89) | 15.61 (7.02, 27.38) | -25.62 (-32.63, -17.21) | -1.01 (-1.07, -0.96)* |
| Solomon Islands | 0.05 (0.02, 0.09) | 0.10 (0.05, 0.18) | 109.13 (89.20, 130.16) | 5.38 (2.94, 8.50) | 5.43 (2.93, 8.62) | 0.90 (-2.30, 4.88) | 19.11 (8.64, 33.45) | 18.15 (8.21, 32.39) | -5.03 (-11.67, 2.14) | -0.20 (-0.26, -0.14)* |
| Somalia | 1.77 (0.82, 3.12) | 5.00 (2.30, 8.67) | 182.26 (159.62, 207.39) | 5.42 (2.91, 8.57) | 5.36 (2.86, 8.52) | -1.25 (-5.22, 3.29) | 38.29 (17.69, 66.63) | 37.57 (17.55, 65.69) | -1.88 (-9.12, 6.27) | -0.06 (-0.09, -0.04)* |
| South Africa | 8.81 (3.99, 15.30) | 18.09 (8.37, 31.60) | 105.27 (89.87, 119.76) | 4.61 (2.48, 7.23) | 5.23 (2.84, 8.20) | 13.34 (8.16, 18.55) | 27.96 (12.99, 48.74) | 31.71 (14.72, 55.40) | 13.45 (7.65, 19.59) | 0.45 (0.39, 0.51)* |
| South Sudan | 1.50 (0.68, 2.62) | 2.38 (1.09, 4.16) | 59.17 (44.27, 74.38) | 5.82 (3.10, 9.14) | 5.57 (2.99, 8.84) | -4.15 (-8.64, 0.18) | 37.90 (17.48, 66.73) | 37.46 (17.20, 66.36) | -1.17 (-9.70, 7.10) | -0.04 (-0.06, -0.03)* |
| Spain | 6.85 (3.21, 11.85) | 11.49 (5.30, 20.21) | 67.68 (42.33, 97.17) | 2.68 (1.43, 4.27) | 2.89 (1.55, 4.63) | 7.84 (-5.26, 25.06) | 16.25 (7.62, 28.30) | 21.47 (9.85, 37.70) | 32.15 (13.09, 55.09) | 1.00 (0.85, 1.15)* |
| Sri Lanka | 2.93 (1.33, 5.25) | 3.02 (1.33, 5.39) | 2.98 (-8.95, 18.02) | 4.23 (2.26, 6.88) | 4.25 (2.23, 6.91) | 0.44 (-5.18, 6.39) | 17.51 (8.05, 31.25) | 12.94 (5.66, 23.18) | -26.07 (-32.67, -18.86) | -1.04 (-1.16, -0.92)* |
| Sudan | 3.27 (1.47, 5.79) | 7.35 (3.30, 13.13) | 124.93 (106.31, 146.34) | 3.09 (1.60, 5.06) | 3.13 (1.63, 5.09) | 1.48 (-2.67, 6.35) | 21.80 (9.64, 38.41) | 21.57 (9.68, 38.74) | -1.02 (-9.09, 7.97) | -0.03 (-0.06, -0.01)* |
| Suriname | 0.08 (0.04, 0.15) | 0.15 (0.07, 0.27) | 78.70 (61.83, 99.94) | 3.22 (1.72, 5.20) | 3.22 (1.70, 5.21) | -0.02 (-2.78, 2.76) | 23.80 (10.42, 43.26) | 25.16 (11.16, 44.75) | 5.72 (-2.16, 15.23) | 0.20 (0.18, 0.22)* |
| Sweden | 3.31 (1.50, 5.81) | 4.61 (2.14, 7.96) | 39.20 (25.69, 53.31) | 5.53 (2.95, 8.70) | 6.80 (3.68, 10.77) | 23.10 (15.09, 32.15) | 34.85 (15.68, 60.59) | 41.73 (19.18, 72.26) | 19.76 (9.33, 31.70) | 0.63 (0.54, 0.72)* |
| Switzerland | 2.95 (1.34, 5.21) | 2.99 (1.39, 5.30) | 1.14 (-11.17, 16.17) | 5.63 (2.97, 9.24) | 5.73 (3.05, 9.25) | 1.77 (-6.65, 10.74) | 37.52 (16.87, 66.14) | 29.64 (13.64, 52.52) | -21.00 (-30.01, -9.86) | -0.77 (-0.94, -0.59)* |
| Syrian Arab Republic | 1.71 (0.75, 3.07) | 2.65 (1.16, 4.81) | 54.97 (35.19, 78.06) | 3.05 (1.58, 4.96) | 2.91 (1.53, 4.72) | -4.67 (-10.12, 0.07) | 19.05 (8.43, 33.74) | 18.06 (7.91, 32.44) | -5.19 (-12.83, 2.46) | -0.19 (-0.22, -0.15)* |
| Taiwan (Province of China) | 0.79 (0.35, 1.46) | 1.27 (0.56, 2.36) | 60.18 (36.79, 90.18) | 1.72 (0.90, 2.86) | 1.73 (0.90, 2.87) | 0.74 (-3.51, 5.31) | 3.83 (1.71, 7.04) | 4.18 (1.86, 7.65) | 9.09 (-0.71, 19.62) | 0.30 (0.28, 0.32)* |
| Tajikistan | 0.25 (0.11, 0.44) | 0.46 (0.20, 0.84) | 87.55 (68.88, 108.87) | 1.57 (0.83, 2.54) | 1.56 (0.82, 2.54) | -0.28 (-3.14, 2.97) | 6.09 (2.70, 10.96) | 5.45 (2.43, 9.66) | -10.46 (-17.92, -1.69) | -0.39 (-0.44, -0.35)* |
| Thailand | 5.98 (2.72, 10.74) | 10.03 (4.47, 17.81) | 67.67 (39.49, 103.69) | 3.57 (1.93, 5.71) | 3.84 (2.08, 6.18) | 7.44 (-4.01, 23.39) | 10.75 (4.98, 19.42) | 11.58 (5.14, 20.40) | 7.76 (-6.43, 26.86) | 0.26 (0.23, 0.29)* |
| Timor-Leste | 0.11 (0.05, 0.20) | 0.17 (0.07, 0.29) | 47.46 (33.90, 62.59) | 4.98 (2.67, 7.87) | 4.96 (2.68, 7.88) | -0.41 (-3.24, 2.58) | 17.42 (7.88, 30.25) | 14.54 (6.41, 25.63) | -16.52 (-22.66, -10.44) | -0.61 (-0.66, -0.56)* |
| Togo | 1.50 (0.69, 2.58) | 4.03 (1.82, 6.96) | 168.72 (145.61, 193.07) | 10.07 (5.67, 15.60) | 9.96 (5.57, 15.49) | -1.14 (-3.72, 1.31) | 62.85 (28.93, 110.42) | 64.81 (30.25, 111.89) | 3.12 (-4.33, 10.54) | 0.11 (0.09, 0.12)* |
| Tokelau | 0.00 (0.00, 0.00) | 0.00 (0.00, 0.00) | -3.74 (-10.56, 3.45) | 4.07 (2.18, 6.52) | 4.04 (2.15, 6.43) | -0.75 (-3.71, 2.14) | 14.95 (6.44, 27.02) | 14.58 (6.25, 26.21) | -2.43 (-8.09, 3.84) | -0.09 (-0.14, -0.05)* |
| Tonga | 0.01 (0.00, 0.01) | 0.01 (0.00, 0.01) | 7.53 (-7.57, 24.38) | 2.89 (1.52, 4.68) | 2.69 (1.44, 4.39) | -6.78 (-18.02, 4.04) | 7.89 (3.57, 14.28) | 7.23 (3.24, 13.08) | -8.37 (-20.15, 3.98) | -0.31 (-0.36, -0.27)* |
| Trinidad and Tobago | 0.24 (0.10, 0.43) | 0.30 (0.13, 0.53) | 27.89 (10.53, 47.69) | 3.23 (1.71, 5.18) | 3.21 (1.70, 5.12) | -0.58 (-3.90, 2.95) | 20.73 (9.21, 37.25) | 19.32 (8.62, 34.23) | -6.84 (-16.02, 2.21) | -0.24 (-0.28, -0.21)* |
| Tunisia | 1.71 (0.76, 3.03) | 3.13 (1.41, 5.55) | 82.46 (61.00, 106.49) | 2.98 (1.56, 4.87) | 2.94 (1.52, 4.78) | -1.41 (-5.60, 2.97) | 24.59 (10.88, 43.91) | 24.12 (10.89, 42.13) | -1.90 (-9.13, 6.18) | -0.07 (-0.11, -0.03)* |
| Turkey | 11.55 (5.42, 19.85) | 18.33 (8.36, 32.15) | 58.64 (35.77, 81.91) | 3.51 (1.83, 5.64) | 3.35 (1.78, 5.35) | -4.59 (-12.90, 3.79) | 21.36 (9.91, 37.17) | 19.65 (8.94, 34.29) | -7.97 (-20.20, 5.07) | -0.28 (-0.46, -0.11)* |
| Turkmenistan | 0.20 (0.09, 0.35) | 0.31 (0.14, 0.55) | 54.74 (36.07, 76.89) | 1.55 (0.81, 2.50) | 1.54 (0.82, 2.49) | -0.48 (-3.76, 3.27) | 6.78 (2.96, 12.19) | 6.15 (2.72, 11.08) | -9.28 (-16.97, -0.41) | -0.34 (-0.36, -0.32)* |
| Tuvalu | 0.00 (0.00, 0.00) | 0.00 (0.00, 0.00) | 34.04 (25.24, 43.44) | 4.06 (2.17, 6.51) | 4.04 (2.18, 6.45) | -0.60 (-4.74, 3.52) | 14.89 (6.43, 27.00) | 14.57 (6.35, 26.13) | -2.13 (-8.35, 4.39) | -0.08 (-0.11, -0.05)* |
| Uganda | 8.09 (3.68, 14.03) | 19.14 (8.72, 33.52) | 136.57 (117.20, 155.86) | 6.67 (3.70, 10.45) | 6.61 (3.67, 10.35) | -0.96 (-3.47, 1.67) | 73.19 (33.47, 125.94) | 70.62 (32.50, 123.04) | -3.51 (-10.84, 4.71) | -0.10 (-0.28, 0.08) |
| Ukraine | 17.77 (7.96, 31.60) | 14.44 (6.44, 25.84) | -18.74 (-23.92, -13.20) | 4.62 (2.47, 7.39) | 4.60 (2.47, 7.32) | -0.43 (-2.71, 1.89) | 30.02 (13.35, 53.15) | 26.51 (11.75, 47.08) | -11.71 (-16.30, -6.61) | -0.43 (-0.48, -0.38)* |
| United Arab Emirates | 0.42 (0.18, 0.80) | 2.94 (1.26, 5.63) | 593.99 (466.48, 728.91) | 3.75 (1.89, 6.26) | 3.86 (1.98, 6.50) | 2.84 (-1.89, 7.83) | 21.70 (9.65, 39.88) | 20.36 (8.89, 37.99) | -6.14 (-14.90, 3.52) | -0.23 (-0.26, -0.19)* |
| United Kingdom | 28.45 (12.78, 48.60) | 30.74 (14.00, 53.92) | 8.05 (0.44, 16.03) | 6.47 (3.53, 10.16) | 6.80 (3.70, 10.72) | 5.09 (-1.13, 11.02) | 45.96 (20.60, 79.51) | 41.54 (18.86, 72.31) | -9.62 (-15.00, -4.15) | -0.36 (-0.43, -0.30)* |
| United Republic of Tanzania | 8.75 (4.14, 14.97) | 18.75 (8.67, 33.38) | 114.26 (86.94, 144.52) | 7.21 (3.94, 11.31) | 6.96 (3.82, 11.02) | -3.45 (-12.40, 6.39) | 49.67 (23.49, 85.74) | 45.95 (21.36, 80.68) | -7.48 (-18.41, 4.56) | -0.27 (-0.32, -0.22)* |
| United States Virgin Islands | 0.02 (0.01, 0.03) | 0.02 (0.01, 0.03) | 12.77 (-0.38, 29.32) | 3.20 (1.69, 5.15) | 3.19 (1.70, 5.10) | -0.23 (-3.05, 2.37) | 15.53 (6.73, 27.30) | 16.05 (6.93, 28.56) | 3.36 (-4.59, 10.96) | 0.11 (0.07, 0.15)* |
| United States of America | 80.41 (36.71, 142.06) | 159.57 (73.73, 277.31) | 98.45 (82.82, 116.76) | 5.90 (3.21, 9.44) | 7.06 (3.88, 11.10) | 19.68 (12.39, 29.12) | 29.34 (13.49, 52.01) | 46.72 (21.83, 81.14) | 59.24 (49.25, 72.33) | 1.60 (1.50, 1.71)* |
| Uruguay | 0.57 (0.26, 1.04) | 0.77 (0.34, 1.37) | 34.35 (22.25, 48.58) | 4.65 (2.55, 7.53) | 4.67 (2.53, 7.54) | 0.29 (-2.92, 3.54) | 17.84 (8.15, 32.38) | 20.48 (9.08, 36.31) | 14.78 (4.45, 26.84) | 0.50 (0.44, 0.57)* |
| Uzbekistan | 1.15 (0.51, 2.07) | 2.12 (0.94, 3.85) | 83.96 (62.05, 106.61) | 1.56 (0.82, 2.54) | 1.56 (0.81, 2.52) | -0.11 (-3.45, 3.11) | 7.06 (3.22, 12.65) | 6.55 (2.90, 11.78) | -7.19 (-15.87, 1.76) | -0.26 (-0.27, -0.24)* |
| Vanuatu | 0.02 (0.01, 0.03) | 0.03 (0.02, 0.06) | 101.28 (85.58, 118.62) | 3.99 (2.13, 6.37) | 3.99 (2.16, 6.31) | -0.11 (-2.73, 2.70) | 14.16 (6.37, 24.90) | 13.12 (5.94, 23.78) | -7.35 (-13.84, -0.20) | -0.27 (-0.29, -0.24)* |
| Venezuela (Bolivarian Republic of) | 4.01 (1.77, 7.01) | 7.79 (3.48, 13.90) | 94.17 (73.01, 118.73) | 5.38 (2.90, 8.47) | 5.37 (2.89, 8.53) | -0.29 (-2.83, 2.13) | 25.60 (11.37, 45.33) | 26.01 (11.76, 46.06) | 1.62 (-5.43, 8.72) | 0.05 (0.03, 0.08)* |
| Viet Nam | 3.69 (1.66, 6.68) | 6.17 (2.76, 11.18) | 67.09 (40.80, 97.46) | 2.52 (1.32, 4.07) | 2.56 (1.32, 4.22) | 1.51 (-10.14, 12.71) | 6.34 (2.87, 11.42) | 5.71 (2.55, 10.32) | -9.91 (-20.54, 2.05) | -0.36 (-0.42, -0.30)* |
| Yemen | 2.07 (0.89, 3.68) | 6.04 (2.65, 10.59) | 190.97 (167.00, 218.50) | 2.98 (1.55, 4.86) | 3.00 (1.56, 4.97) | 0.69 (-4.01, 5.34) | 23.09 (10.04, 40.90) | 23.47 (10.40, 41.77) | 1.67 (-6.61, 10.06) | 0.06 (0.04, 0.07)* |
| Zambia | 1.41 (0.64, 2.51) | 3.68 (1.66, 6.53) | 159.92 (134.00, 190.06) | 5.07 (2.69, 8.05) | 5.14 (2.74, 8.32) | 1.42 (-4.45, 7.64) | 28.77 (13.09, 50.61) | 29.20 (13.50, 52.35) | 1.53 (-8.45, 12.77) | 0.05 (0.01, 0.09)* |
| Zimbabwe | 1.02 (0.45, 1.78) | 1.73 (0.76, 3.00) | 69.06 (55.66, 84.33) | 3.53 (1.88, 5.63) | 3.52 (1.88, 5.62) | -0.16 (-2.97, 2.88) | 14.10 (6.25, 24.76) | 14.55 (6.55, 25.37) | 3.14 (-3.78, 11.18) | 0.11 (0.08, 0.14)* |

| Table S17 The number, PAFs and ASDR of MDD attributed to BV in 1990 and 2019, and the corresponding percentage change and AAPC of ASDR during 1990-2019 across 204 countries and territories. | | | | | | | | | | |
| --- | --- | --- | --- | --- | --- | --- | --- | --- | --- | --- |
|  |  |  | Percentage change of |  |  | Percentage change of |  |  | Percentage change of | AAPC (95% CI) |
| Countries or territories | BV-related DALYs  number ×10^3^ (95% UI) | | number (%, 95% UI) | PAFs (%, 95% UI) | | PAFs (%, 95% UI) | BV-related ASDR per 100 000 (95% UI) | | ASDR (%, 95% UI) | of BV-related ASDR |
|  | 1990 year | 2019 year | during 1990-2019 | 1990 year | 2019 year | during 1990-2019 | 1990 year | 2019 year | during 1990-2019 | during 1990-2019 |
| Afghanistan | 2.28 (0.48, 5.57) | 11.51 (2.50, 27.51) | 405.21 (317.13, 526.18) | 2.23 (0.49, 5.11) | 3.13 (0.76, 6.95) | 40.20 (18.79, 74.87) | 17.68 (3.16, 45.94) | 24.64 (4.89, 61.70) | 39.36 (16.61, 73.81) | 1.15 (1.06, 1.24)* |
| Albania | 0.32 (0.06, 0.78) | 0.22 (0.04, 0.54) | -30.02 (-41.50, -19.57) | 3.11 (0.67, 6.92) | 3.23 (0.69, 7.28) | 4.07 (-8.13, 16.76) | 8.10 (1.53, 20.27) | 8.98 (1.76, 21.69) | 10.84 (-3.38, 26.75) | 0.35 (0.31, 0.39)* |
| Algeria | 7.96 (1.79, 18.87) | 15.24 (3.28, 35.53) | 91.48 (36.26, 197.74) | 3.89 (0.97, 8.58) | 5.83 (1.53, 11.73) | 49.72 (14.80, 139.54) | 25.57 (5.41, 62.11) | 36.77 (8.37, 83.80) | 43.80 (9.72, 130.00) | 1.27 (1.16, 1.37)* |
| American Samoa | 0.01 (0.00, 0.02) | 0.01 (0.00, 0.02) | 10.05 (-2.56, 27.23) | 5.48 (1.30, 11.73) | 5.77 (1.38, 12.55) | 5.20 (-4.20, 16.27) | 14.84 (3.04, 36.09) | 15.08 (3.09, 36.25) | 1.59 (-9.88, 14.15) | 0.05 (0.02, 0.09)* |
| Andorra | 0.01 (0.00, 0.03) | 0.01 (0.00, 0.03) | -2.73 (-12.02, 9.62) | 4.55 (1.01, 10.06) | 4.58 (1.01, 9.96) | 0.69 (-7.54, 9.12) | 24.28 (4.74, 58.32) | 23.78 (4.62, 56.14) | -2.06 (-11.68, 8.52) | -0.08 (-0.12, -0.04)* |
| Angola | 3.78 (0.71, 9.01) | 13.49 (3.01, 31.18) | 256.79 (206.35, 342.32) | 3.46 (0.71, 7.89) | 4.40 (1.00, 9.73) | 27.22 (11.55, 55.17) | 33.31 (5.73, 81.53) | 40.43 (8.22, 96.58) | 21.39 (4.15, 49.46) | 0.68 (0.64, 0.71)* |
| Antigua and Barbuda | 0.01 (0.00, 0.03) | 0.01 (0.00, 0.04) | 31.72 (16.53, 47.33) | 3.56 (0.86, 8.02) | 3.83 (0.90, 8.32) | 7.52 (-2.20, 19.21) | 15.53 (3.22, 37.44) | 16.68 (3.45, 40.70) | 7.36 (-3.33, 20.41) | 0.24 (0.15, 0.34)* |
| Argentina | 5.86 (1.26, 13.64) | 9.66 (2.06, 22.43) | 64.99 (16.05, 142.73) | 4.49 (1.09, 9.60) | 5.93 (1.45, 12.46) | 31.99 (-4.24, 97.00) | 17.46 (3.59, 40.99) | 21.97 (4.91, 50.63) | 25.85 (-10.44, 86.77) | 0.79 (0.73, 0.84)* |
| Armenia | 0.12 (0.02, 0.32) | 0.11 (0.02, 0.29) | -8.42 (-31.97, 43.77) | 0.95 (0.19, 2.24) | 1.16 (0.25, 2.70) | 22.78 (-4.23, 91.52) | 3.33 (0.58, 8.56) | 4.46 (0.84, 11.07) | 33.77 (3.63, 113.30) | 1.01 (0.87, 1.16)* |
| Australia | 5.11 (0.98, 12.38) | 6.49 (1.38, 15.47) | 26.99 (5.91, 67.02) | 4.37 (1.04, 9.50) | 4.79 (1.27, 10.13) | 9.67 (-7.14, 47.74) | 30.60 (6.22, 73.07) | 33.57 (7.76, 76.14) | 9.70 (-8.15, 45.59) | 0.32 (0.26, 0.36)* |
| Austria | 1.86 (0.33, 4.60) | 1.67 (0.35, 3.79) | -10.17 (-30.89, 57.60) | 4.95 (1.14, 10.90) | 6.45 (1.72, 13.16) | 30.27 (1.87, 126.94) | 25.86 (5.08, 62.26) | 26.90 (6.44, 59.92) | 4.00 (-19.49, 81.55) | 0.13 (0.10, 0.16)* |
| Azerbaijan | 0.37 (0.07, 0.93) | 0.47 (0.07, 1.25) | 27.40 (3.78, 47.39) | 1.26 (0.28, 2.96) | 1.40 (0.29, 3.30) | 10.81 (-2.12, 26.38) | 4.26 (0.79, 10.77) | 4.69 (0.82, 11.96) | 10.04 (-2.63, 26.05) | 0.33 (0.23, 0.43)* |
| Bahamas | 0.05 (0.01, 0.13) | 0.06 (0.01, 0.15) | 22.09 (-3.40, 65.88) | 3.65 (0.82, 8.00) | 3.84 (0.97, 8.01) | 5.15 (-15.24, 43.53) | 16.47 (3.25, 40.51) | 17.01 (3.60, 40.12) | 3.28 (-17.10, 42.31) | 0.11 (0.05, 0.16)* |
| Bahrain | 0.25 (0.04, 0.64) | 0.44 (0.08, 1.16) | 78.81 (56.19, 103.29) | 4.81 (1.11, 10.76) | 5.09 (1.23, 11.06) | 5.78 (-3.67, 17.13) | 40.01 (7.86, 98.40) | 36.77 (7.65, 90.96) | -8.11 (-16.93, 2.91) | -0.31 (-0.39, -0.22)* |
| Bangladesh | 44.25 (8.70, 102.01) | 68.43 (12.71, 156.78) | 54.65 (29.47, 82.44) | 4.80 (0.91, 10.44) | 5.35 (1.13, 11.32) | 11.50 (-4.00, 33.24) | 35.33 (6.29, 86.04) | 37.78 (7.03, 86.94) | 6.95 (-8.12, 29.84) | 0.23 (0.19, 0.26)* |
| Barbados | 0.02 (0.00, 0.05) | 0.02 (0.00, 0.05) | -2.27 (-23.49, 40.41) | 1.60 (0.34, 3.78) | 1.78 (0.43, 3.91) | 11.25 (-13.20, 58.93) | 7.40 (1.34, 19.04) | 8.35 (1.69, 20.49) | 12.79 (-12.17, 62.31) | 0.40 (0.33, 0.48)* |
| Belarus | 2.84 (0.49, 6.92) | 1.95 (0.27, 5.14) | -31.22 (-46.25, -21.38) | 5.00 (1.07, 10.78) | 5.00 (1.02, 10.95) | -0.10 (-13.44, 10.65) | 28.97 (5.41, 70.41) | 28.02 (4.85, 67.28) | -3.28 (-16.43, 7.82) | -0.10 (-0.20, 0.00) |
| Belgium | 2.29 (0.43, 5.52) | 2.79 (0.59, 6.47) | 21.77 (-7.85, 87.75) | 5.58 (1.28, 12.02) | 6.43 (1.64, 13.52) | 15.34 (-11.42, 78.68) | 26.34 (5.52, 61.19) | 33.65 (7.73, 76.11) | 27.73 (-3.09, 94.46) | 0.85 (0.79, 0.91)* |
| Belize | 0.04 (0.01, 0.10) | 0.11 (0.02, 0.26) | 153.12 (91.41, 248.36) | 4.07 (0.97, 9.05) | 4.50 (1.10, 9.51) | 10.63 (-11.85, 52.76) | 19.41 (4.01, 46.71) | 22.31 (4.82, 52.26) | 14.93 (-8.74, 59.80) | 0.50 (0.45, 0.56)* |
| Benin | 0.56 (0.11, 1.42) | 2.63 (0.59, 6.10) | 370.08 (252.72, 636.96) | 1.79 (0.36, 4.14) | 2.79 (0.64, 6.13) | 56.19 (15.99, 145.61) | 11.00 (1.94, 28.72) | 17.95 (3.61, 42.05) | 63.09 (22.31, 157.86) | 1.71 (1.67, 1.75)* |
| Bermuda | 0.01 (0.00, 0.03) | 0.01 (0.00, 0.02) | -37.26 (-45.34, -25.89) | 3.66 (0.91, 8.19) | 3.88 (0.93, 8.45) | 5.99 (-6.07, 19.65) | 20.37 (4.35, 48.95) | 18.21 (3.78, 43.73) | -10.59 (-22.28, 3.24) | -0.39 (-0.41, -0.36)* |
| Bhutan | 0.15 (0.03, 0.36) | 0.24 (0.04, 0.56) | 57.63 (28.87, 94.93) | 3.17 (0.61, 7.55) | 4.51 (1.02, 9.60) | 42.28 (19.28, 86.19) | 19.93 (3.48, 50.37) | 26.22 (5.28, 61.12) | 31.58 (8.81, 71.83) | 0.95 (0.86, 1.04)* |
| Bolivia (Plurinational State of) | 1.04 (0.20, 2.52) | 1.83 (0.34, 4.39) | 75.72 (29.22, 146.17) | 2.56 (0.53, 5.95) | 2.76 (0.58, 6.05) | 7.78 (-17.03, 54.99) | 14.55 (2.59, 36.63) | 14.14 (2.69, 34.00) | -2.82 (-25.64, 40.87) | -0.10 (-0.13, -0.07)* |
| Bosnia and Herzegovina | 0.73 (0.14, 1.85) | 0.26 (0.04, 0.68) | -64.25 (-70.44, -58.83) | 3.32 (0.71, 7.53) | 3.11 (0.67, 7.08) | -6.18 (-18.71, 5.76) | 14.82 (2.90, 36.84) | 10.49 (1.95, 26.44) | -29.19 (-40.12, -18.10) | -1.20 (-1.24, -1.16)* |
| Botswana | 0.37 (0.09, 0.85) | 0.99 (0.20, 2.27) | 165.44 (85.73, 262.59) | 4.01 (0.99, 8.71) | 6.16 (1.50, 12.86) | 53.73 (17.75, 112.42) | 23.20 (5.01, 54.52) | 36.62 (7.83, 83.65) | 57.86 (21.38, 121.04) | 1.59 (1.55, 1.62)* |
| Brazil | 31.08 (6.32, 75.07) | 48.79 (10.88, 115.53) | 57.02 (39.76, 81.61) | 2.74 (0.62, 6.21) | 3.93 (1.04, 8.26) | 43.55 (26.89, 79.62) | 17.57 (3.41, 43.31) | 23.67 (5.59, 55.38) | 34.73 (16.93, 69.87) | 1.02 (0.82, 1.23)* |
| Brunei Darussalam | 0.03 (0.01, 0.08) | 0.06 (0.01, 0.13) | 71.75 (32.43, 115.35) | 5.20 (1.34, 11.07) | 6.00 (1.55, 12.39) | 15.34 (-8.63, 52.69) | 10.29 (2.21, 23.95) | 11.94 (2.65, 27.31) | 16.05 (-9.17, 48.65) | 0.51 (0.43, 0.58)* |
| Bulgaria | 1.44 (0.29, 3.37) | 0.72 (0.13, 1.71) | -50.11 (-62.18, -32.92) | 4.98 (1.17, 10.73) | 5.03 (1.19, 10.42) | 0.99 (-18.00, 38.16) | 19.23 (3.97, 44.61) | 16.28 (3.40, 37.12) | -15.33 (-31.41, 16.56) | -0.58 (-0.62, -0.54)* |
| Burkina Faso | 0.69 (0.15, 1.81) | 3.96 (0.91, 9.38) | 470.68 (345.68, 668.85) | 1.08 (0.23, 2.68) | 2.39 (0.59, 5.32) | 121.05 (71.35, 207.51) | 6.91 (1.28, 18.39) | 15.01 (3.21, 36.57) | 117.34 (68.35, 204.35) | 2.74 (2.46, 3.03)* |
| Burundi | 0.78 (0.15, 1.94) | 3.03 (0.67, 7.18) | 290.17 (220.26, 410.54) | 1.49 (0.31, 3.60) | 3.25 (0.74, 7.29) | 117.45 (80.42, 184.09) | 12.69 (2.30, 32.51) | 21.87 (4.42, 53.56) | 72.34 (41.52, 124.65) | 1.90 (1.85, 1.94)* |
| Cabo Verde | 0.10 (0.02, 0.25) | 0.21 (0.04, 0.52) | 105.88 (67.48, 136.83) | 3.79 (0.78, 8.84) | 4.47 (0.96, 10.02) | 17.85 (5.87, 36.25) | 25.62 (4.55, 64.47) | 32.76 (6.15, 79.91) | 27.88 (14.79, 47.64) | 0.86 (0.80, 0.92)* |
| Cambodia | 0.89 (0.17, 2.18) | 1.56 (0.33, 3.76) | 74.08 (32.65, 138.17) | 2.01 (0.40, 4.75) | 2.62 (0.65, 5.66) | 30.17 (0.76, 81.42) | 7.60 (1.32, 18.98) | 8.40 (1.86, 20.27) | 10.55 (-13.79, 57.19) | 0.35 (0.32, 0.38)* |
| Cameroon | 2.26 (0.48, 5.38) | 9.29 (1.93, 22.23) | 310.43 (260.48, 362.66) | 3.05 (0.68, 6.95) | 3.93 (0.87, 8.88) | 28.85 (15.19, 43.27) | 19.69 (3.86, 48.33) | 26.50 (5.02, 64.06) | 34.60 (18.65, 50.53) | 1.03 (0.99, 1.06)* |
| Canada | 5.47 (0.97, 13.52) | 9.73 (2.06, 22.05) | 77.92 (38.15, 165.17) | 4.76 (1.12, 10.39) | 8.15 (2.11, 15.85) | 71.07 (37.68, 147.35) | 21.03 (4.26, 50.86) | 35.96 (8.32, 78.98) | 71.00 (33.64, 147.98) | 1.88 (1.82, 1.93)* |
| Central African Republic | 0.78 (0.14, 1.83) | 1.98 (0.40, 4.66) | 154.91 (125.77, 211.21) | 2.61 (0.50, 6.16) | 3.33 (0.72, 7.47) | 27.80 (13.76, 52.01) | 25.12 (4.28, 61.93) | 31.72 (5.90, 77.48) | 26.30 (12.21, 51.03) | 0.81 (0.78, 0.84)* |
| Chad | 0.74 (0.15, 1.81) | 3.80 (0.88, 8.90) | 413.77 (315.48, 579.67) | 1.52 (0.32, 3.61) | 2.60 (0.58, 5.86) | 70.68 (37.80, 125.09) | 11.61 (2.08, 29.03) | 20.47 (4.15, 50.44) | 76.28 (41.05, 132.29) | 1.97 (1.92, 2.02)* |
| Chile | 7.47 (1.59, 16.86) | 7.49 (1.40, 17.37) | 0.25 (-28.89, 34.18) | 6.44 (1.50, 13.47) | 7.31 (1.63, 15.44) | 13.58 (-12.42, 52.31) | 48.89 (10.41, 111.06) | 44.65 (9.43, 102.36) | -8.67 (-31.27, 22.97) | -0.30 (-0.38, -0.23)* |
| China | 193.10 (36.31, 461.68) | 106.26 (17.83, 272.69) | -44.97 (-54.68, -37.60) | 3.85 (0.84, 8.47) | 3.34 (0.78, 7.11) | -13.18 (-21.18, -0.41) | 12.98 (2.50, 31.50) | 9.58 (1.94, 22.77) | -26.19 (-32.53, -14.99) | -1.08 (-1.22, -0.95)* |
| Colombia | 4.20 (0.85, 10.35) | 5.57 (1.09, 13.20) | 32.48 (2.62, 90.68) | 3.26 (0.74, 7.46) | 3.96 (0.97, 8.37) | 21.32 (-3.45, 76.23) | 10.66 (2.10, 26.82) | 11.37 (2.35, 26.76) | 6.63 (-16.08, 53.85) | 0.24 (0.18, 0.29)* |
| Comoros | 0.09 (0.02, 0.23) | 0.19 (0.04, 0.43) | 96.39 (66.16, 134.16) | 2.86 (0.59, 6.64) | 3.84 (0.85, 8.37) | 34.49 (15.62, 67.51) | 17.56 (3.28, 43.66) | 22.49 (4.52, 52.52) | 28.08 (9.26, 58.94) | 0.85 (0.81, 0.90)* |
| Congo | 1.31 (0.26, 3.07) | 2.62 (0.53, 6.35) | 99.75 (76.37, 125.66) | 4.85 (0.98, 10.88) | 5.21 (1.18, 11.52) | 7.46 (-2.98, 25.30) | 46.62 (8.38, 113.87) | 45.49 (8.95, 111.47) | -2.43 (-12.63, 13.25) | -0.08 (-0.12, -0.04)* |
| Cook Islands | 0.00 (0.00, 0.01) | 0.00 (0.00, 0.01) | -18.54 (-35.06, 19.13) | 5.09 (1.10, 11.23) | 5.67 (1.28, 11.96) | 11.53 (-10.07, 64.21) | 18.58 (3.39, 46.41) | 20.80 (4.39, 49.75) | 11.93 (-9.94, 64.93) | 0.39 (0.37, 0.42)* |
| Costa Rica | 0.29 (0.05, 0.73) | 0.54 (0.10, 1.36) | 86.77 (36.08, 198.55) | 1.77 (0.35, 4.23) | 2.34 (0.53, 5.24) | 32.02 (0.55, 115.67) | 8.13 (1.38, 20.37) | 11.36 (2.24, 28.05) | 39.70 (4.36, 128.24) | 1.16 (1.11, 1.20)* |
| Croatia | 0.60 (0.10, 1.52) | 0.35 (0.06, 0.86) | -41.55 (-59.05, -5.00) | 2.83 (0.56, 6.53) | 3.02 (0.69, 6.48) | 6.96 (-23.71, 72.69) | 13.65 (2.40, 33.87) | 11.73 (2.30, 27.85) | -14.06 (-39.44, 39.21) | -0.53 (-0.60, -0.46)* |
| Cuba | 4.05 (0.79, 9.99) | 1.82 (0.35, 4.56) | -54.97 (-62.70, -48.05) | 3.69 (0.83, 8.34) | 3.48 (0.82, 7.74) | -5.83 (-16.33, 6.98) | 30.65 (6.12, 75.40) | 20.77 (4.26, 50.61) | -32.21 (-42.01, -20.70) | -1.34 (-1.42, -1.25)* |
| Cyprus | 0.13 (0.03, 0.32) | 0.20 (0.03, 0.50) | 51.63 (22.03, 73.03) | 4.04 (0.98, 8.83) | 4.75 (1.08, 10.23) | 17.47 (7.28, 28.55) | 16.75 (3.45, 40.53) | 19.65 (3.99, 46.13) | 17.30 (6.27, 29.21) | 0.54 (0.41, 0.68)* |
| Czechia | 0.82 (0.15, 2.12) | 0.55 (0.09, 1.40) | -33.25 (-53.25, -3.62) | 1.96 (0.41, 4.61) | 2.21 (0.47, 5.01) | 12.67 (-15.14, 63.54) | 8.71 (1.56, 22.24) | 8.00 (1.50, 19.24) | -8.25 (-30.22, 33.27) | -0.30 (-0.38, -0.21)* |
| Côte d’Ivoire | 1.68 (0.34, 4.08) | 4.96 (1.06, 11.84) | 195.89 (144.42, 264.48) | 2.26 (0.48, 5.38) | 3.05 (0.71, 6.94) | 34.72 (13.05, 69.24) | 11.95 (2.27, 30.08) | 16.34 (3.36, 39.53) | 36.73 (13.54, 69.88) | 1.09 (1.05, 1.12)* |
| Democratic People's Republic of Korea | 3.36 (0.68, 8.04) | 3.14 (0.59, 7.81) | -6.49 (-21.42, 5.67) | 4.92 (1.14, 10.52) | 4.86 (1.17, 10.36) | -1.20 (-11.18, 10.59) | 15.03 (3.12, 36.00) | 12.88 (2.70, 30.91) | -14.35 (-24.28, -2.99) | -0.54 (-0.58, -0.50)* |
| Democratic Republic of the Congo | 12.97 (2.40, 31.57) | 42.98 (9.26, 98.38) | 231.46 (184.86, 313.84) | 3.50 (0.70, 7.92) | 4.87 (1.11, 10.69) | 39.20 (20.14, 73.99) | 30.61 (5.15, 77.63) | 41.58 (8.23, 98.69) | 35.86 (16.85, 71.52) | 1.06 (1.02, 1.10)* |
| Denmark | 1.08 (0.19, 2.55) | 0.83 (0.16, 1.99) | -22.84 (-33.79, -5.75) | 3.40 (0.71, 7.63) | 3.79 (0.85, 8.15) | 11.48 (-4.63, 34.23) | 23.18 (4.50, 54.59) | 18.62 (3.80, 44.54) | -19.65 (-31.12, -3.21) | -0.75 (-0.81, -0.68)* |
| Djibouti | 0.05 (0.01, 0.13) | 0.21 (0.04, 0.49) | 319.40 (217.20, 557.04) | 1.31 (0.27, 3.22) | 2.54 (0.63, 5.41) | 93.97 (45.87, 217.06) | 8.20 (1.42, 21.96) | 16.02 (3.48, 37.53) | 95.21 (47.68, 218.39) | 2.33 (2.26, 2.40)* |
| Dominica | 0.02 (0.00, 0.04) | 0.01 (0.00, 0.03) | -12.81 (-34.18, 22.05) | 4.22 (1.02, 9.26) | 4.67 (1.17, 9.83) | 10.75 (-13.77, 57.50) | 18.76 (3.99, 45.54) | 20.91 (4.45, 48.41) | 11.44 (-13.57, 58.01) | 0.37 (0.29, 0.44)* |
| Dominican Republic | 1.79 (0.39, 4.25) | 2.55 (0.51, 6.22) | 42.24 (23.64, 61.76) | 3.36 (0.79, 7.43) | 3.58 (0.87, 7.97) | 6.57 (-3.01, 20.19) | 19.96 (4.11, 49.29) | 21.63 (4.36, 52.40) | 8.33 (-3.65, 24.17) | 0.25 (0.16, 0.34)* |
| Ecuador | 2.22 (0.48, 5.26) | 4.16 (0.90, 9.64) | 87.23 (46.42, 151.87) | 3.72 (0.86, 8.33) | 4.27 (1.09, 9.06) | 14.72 (-6.65, 54.60) | 18.26 (3.69, 44.02) | 21.36 (4.62, 49.62) | 16.98 (-5.91, 57.58) | 0.54 (0.49, 0.60)* |
| Egypt | 23.36 (5.95, 52.36) | 63.51 (17.60, 134.42) | 171.89 (111.55, 300.26) | 6.52 (1.85, 13.18) | 9.71 (2.93, 18.33) | 48.81 (17.34, 126.05) | 36.41 (8.78, 82.81) | 57.37 (15.64, 121.72) | 57.57 (23.13, 136.29) | 1.57 (1.51, 1.64)* |
| El Salvador | 0.73 (0.15, 1.80) | 0.90 (0.18, 2.18) | 24.01 (-6.90, 75.13) | 1.94 (0.41, 4.49) | 2.43 (0.54, 5.30) | 25.25 (-1.67, 82.27) | 11.43 (2.17, 29.09) | 12.76 (2.53, 31.30) | 11.66 (-13.34, 61.44) | 0.38 (0.34, 0.43)* |
| Equatorial Guinea | 0.10 (0.02, 0.24) | 0.76 (0.16, 1.78) | 654.00 (508.92, 893.02) | 2.33 (0.48, 5.22) | 4.31 (0.99, 9.56) | 85.52 (51.16, 144.38) | 22.66 (4.14, 56.34) | 39.49 (8.05, 95.21) | 74.33 (41.92, 132.12) | 1.93 (1.89, 1.98)* |
| Eritrea | 0.42 (0.09, 1.04) | 1.49 (0.30, 3.58) | 256.79 (185.36, 347.24) | 1.63 (0.34, 3.86) | 2.55 (0.54, 5.89) | 56.36 (26.21, 99.69) | 12.01 (2.26, 31.24) | 17.89 (3.35, 44.59) | 48.95 (19.26, 89.92) | 1.38 (1.35, 1.42)* |
| Estonia | 0.41 (0.07, 1.03) | 0.26 (0.05, 0.60) | -37.60 (-53.18, 2.17) | 4.01 (0.89, 9.19) | 5.28 (1.29, 10.79) | 31.76 (-0.71, 120.44) | 29.56 (5.67, 71.94) | 28.13 (6.03, 62.91) | -4.85 (-28.70, 54.77) | -0.17 (-0.21, -0.13)* |
| Eswatini | 0.17 (0.04, 0.40) | 0.36 (0.07, 0.83) | 114.74 (55.87, 208.86) | 3.04 (0.68, 7.03) | 4.07 (0.90, 8.70) | 33.55 (0.16, 96.05) | 17.54 (3.34, 44.28) | 25.43 (4.97, 59.80) | 44.95 (7.93, 113.97) | 1.28 (1.25, 1.31)* |
| Ethiopia | 6.53 (1.27, 16.63) | 23.21 (4.96, 53.63) | 255.32 (196.50, 355.33) | 1.57 (0.31, 3.70) | 2.57 (0.56, 5.67) | 64.38 (36.03, 117.01) | 11.74 (2.06, 31.37) | 17.31 (3.38, 41.96) | 47.41 (22.07, 92.62) | 1.35 (1.28, 1.41)* |
| Fiji | 0.18 (0.04, 0.41) | 0.20 (0.04, 0.46) | 15.40 (-9.10, 60.19) | 6.20 (1.44, 13.34) | 6.90 (1.67, 14.08) | 11.27 (-9.71, 54.75) | 19.52 (4.01, 46.50) | 21.80 (4.65, 49.19) | 11.69 (-11.21, 57.95) | 0.38 (0.35, 0.40)* |
| Finland | 1.24 (0.23, 3.07) | 1.30 (0.27, 3.01) | 5.00 (-14.07, 39.99) | 3.83 (0.88, 8.44) | 5.53 (1.38, 11.50) | 44.46 (20.95, 88.88) | 29.58 (5.92, 71.45) | 33.43 (7.65, 76.05) | 13.02 (-7.26, 49.17) | 0.42 (0.35, 0.49)* |
| France | 18.71 (3.57, 45.12) | 17.16 (3.77, 39.12) | -8.24 (-32.34, 51.19) | 5.09 (1.12, 11.04) | 6.15 (1.58, 12.85) | 20.78 (-9.22, 98.33) | 35.24 (7.21, 83.52) | 34.88 (7.84, 78.22) | -1.02 (-27.44, 61.89) | -0.02 (-0.11, 0.07) |
| Gabon | 0.52 (0.10, 1.24) | 1.04 (0.20, 2.51) | 99.51 (79.08, 123.14) | 5.16 (1.08, 11.42) | 5.80 (1.24, 12.62) | 12.56 (2.93, 27.05) | 47.40 (8.70, 115.36) | 50.76 (9.50, 124.65) | 7.08 (-3.31, 21.12) | 0.25 (0.20, 0.30)* |
| Gambia | 0.23 (0.05, 0.57) | 0.86 (0.19, 2.05) | 269.91 (210.30, 358.69) | 2.33 (0.53, 5.46) | 3.56 (0.83, 7.89) | 52.47 (26.76, 88.29) | 20.41 (4.19, 51.73) | 30.82 (6.40, 74.93) | 50.99 (25.61, 86.94) | 1.42 (1.38, 1.45)* |
| Georgia | 0.30 (0.05, 0.77) | 0.17 (0.03, 0.44) | -42.24 (-49.69, -33.90) | 1.23 (0.24, 2.86) | 1.38 (0.30, 3.22) | 12.56 (0.67, 28.52) | 5.52 (0.96, 13.86) | 6.26 (1.16, 15.92) | 13.30 (0.24, 31.46) | 0.42 (0.36, 0.48)* |
| Germany | 15.45 (2.73, 38.44) | 17.01 (3.37, 39.77) | 10.09 (-12.57, 52.97) | 5.23 (1.31, 11.26) | 6.15 (1.52, 13.00) | 17.52 (-6.24, 56.83) | 23.12 (4.85, 54.90) | 29.88 (6.78, 67.46) | 29.22 (3.00, 77.22) | 0.90 (0.74, 1.05)* |
| Ghana | 4.53 (1.02, 10.71) | 14.83 (3.63, 31.99) | 227.08 (130.75, 420.45) | 4.29 (0.99, 9.58) | 6.26 (1.65, 12.53) | 45.82 (5.28, 128.77) | 26.70 (5.60, 64.17) | 39.06 (9.20, 85.01) | 46.29 (2.92, 134.43) | 1.31 (1.23, 1.39)* |
| Greece | 2.51 (0.47, 6.19) | 2.33 (0.47, 5.47) | -7.03 (-23.13, 24.69) | 3.27 (0.70, 7.44) | 4.35 (1.03, 9.18) | 33.12 (12.02, 78.58) | 26.49 (5.00, 65.06) | 35.75 (7.61, 82.13) | 34.94 (13.26, 82.55) | 1.03 (0.88, 1.17)* |
| Greenland | 0.05 (0.01, 0.12) | 0.05 (0.01, 0.12) | 0.45 (-15.43, 38.14) | 7.22 (1.58, 15.25) | 10.60 (2.74, 20.80) | 46.85 (25.90, 90.57) | 79.91 (15.36, 180.85) | 104.85 (24.36, 227.30) | 31.21 (11.36, 70.96) | 0.97 (0.92, 1.02)* |
| Grenada | 0.02 (0.00, 0.05) | 0.03 (0.01, 0.06) | 21.05 (-7.29, 57.09) | 4.71 (1.10, 10.24) | 5.13 (1.31, 10.76) | 8.92 (-12.68, 45.22) | 22.61 (4.60, 54.04) | 24.60 (5.23, 56.50) | 8.79 (-12.36, 44.09) | 0.29 (0.26, 0.32)* |
| Guam | 0.04 (0.01, 0.10) | 0.04 (0.01, 0.10) | 2.58 (-8.29, 16.48) | 6.21 (1.39, 13.73) | 6.37 (1.43, 14.06) | 2.51 (-5.65, 11.13) | 23.47 (4.67, 57.98) | 24.29 (4.68, 58.21) | 3.48 (-7.60, 15.54) | 0.12 (0.09, 0.14)* |
| Guatemala | 0.93 (0.19, 2.25) | 3.74 (0.75, 9.15) | 303.25 (243.88, 369.93) | 1.74 (0.38, 4.03) | 2.94 (0.68, 6.76) | 69.56 (52.30, 96.75) | 10.40 (2.01, 26.67) | 16.93 (3.33, 41.91) | 62.89 (43.58, 89.76) | 1.69 (1.62, 1.76)* |
| Guinea | 0.56 (0.10, 1.39) | 2.60 (0.56, 6.05) | 367.54 (283.13, 510.05) | 1.47 (0.29, 3.52) | 2.84 (0.64, 6.36) | 92.79 (58.93, 152.15) | 8.79 (1.50, 22.48) | 17.75 (3.56, 42.77) | 101.92 (66.37, 166.04) | 2.46 (2.40, 2.51)* |
| Guinea-Bissau | 0.13 (0.03, 0.32) | 0.47 (0.10, 1.12) | 259.91 (193.80, 344.07) | 1.84 (0.40, 4.35) | 3.08 (0.71, 6.96) | 67.47 (36.98, 112.14) | 11.43 (2.04, 29.41) | 20.06 (4.10, 49.22) | 75.58 (42.78, 120.92) | 1.95 (1.91, 2.00)* |
| Guyana | 0.36 (0.08, 0.85) | 0.42 (0.09, 0.94) | 15.34 (-14.42, 70.93) | 4.87 (1.11, 10.65) | 5.62 (1.34, 11.54) | 15.46 (-11.52, 71.82) | 36.94 (7.35, 89.52) | 46.60 (10.00, 106.55) | 26.16 (-5.24, 88.00) | 0.82 (0.77, 0.88)* |
| Haiti | 1.13 (0.25, 2.70) | 2.82 (0.57, 6.91) | 149.26 (112.66, 189.95) | 2.77 (0.68, 6.45) | 3.52 (0.85, 7.79) | 26.81 (10.93, 48.60) | 16.01 (3.31, 39.37) | 19.59 (3.92, 48.16) | 22.34 (6.53, 44.05) | 0.70 (0.67, 0.73)* |
| Honduras | 0.60 (0.13, 1.43) | 1.60 (0.33, 3.89) | 165.34 (100.33, 265.38) | 2.51 (0.53, 5.73) | 2.86 (0.64, 6.30) | 13.65 (-9.79, 58.26) | 11.23 (2.21, 27.24) | 13.42 (2.62, 32.96) | 19.57 (-5.08, 67.70) | 0.62 (0.59, 0.66)* |
| Hungary | 0.83 (0.15, 2.13) | 0.55 (0.10, 1.34) | -33.98 (-51.16, 4.13) | 1.93 (0.40, 4.62) | 2.38 (0.52, 5.25) | 23.30 (-6.51, 91.96) | 9.09 (1.59, 22.92) | 8.30 (1.62, 20.00) | -8.63 (-32.08, 43.72) | -0.31 (-0.36, -0.26)* |
| Iceland | 0.03 (0.01, 0.08) | 0.03 (0.01, 0.08) | 8.37 (-18.77, 80.18) | 2.82 (0.59, 6.25) | 3.20 (0.74, 6.98) | 13.67 (-12.65, 98.01) | 12.26 (2.30, 30.39) | 12.14 (2.60, 28.62) | -0.98 (-25.50, 68.84) | -0.03 (-0.09, 0.02) |
| India | 208.00 (37.78, 480.55) | 365.98 (69.36, 847.78) | 75.95 (60.19, 101.78) | 3.82 (0.74, 8.61) | 4.58 (1.02, 9.85) | 20.05 (9.67, 43.90) | 21.98 (3.75, 52.02) | 23.11 (4.37, 53.81) | 5.13 (-4.77, 26.94) | 0.16 (-0.09, 0.42) |
| Indonesia | 17.96 (4.05, 42.25) | 28.47 (5.77, 68.61) | 58.48 (38.02, 77.43) | 3.60 (0.91, 7.90) | 4.78 (1.17, 10.45) | 32.73 (20.83, 47.13) | 7.92 (1.71, 18.97) | 10.45 (2.13, 25.11) | 32.02 (19.52, 46.15) | 0.95 (0.92, 0.99)* |
| Iran (Islamic Republic of) | 20.05 (4.35, 45.43) | 30.45 (5.30, 76.63) | 51.82 (10.78, 83.19) | 3.96 (0.92, 8.56) | 4.79 (1.13, 10.05) | 20.89 (9.49, 34.67) | 29.47 (5.83, 70.82) | 37.60 (7.60, 89.80) | 27.59 (15.60, 42.21) | 0.86 (0.82, 0.91)* |
| Iraq | 2.93 (0.65, 7.09) | 9.97 (2.21, 22.84) | 240.03 (159.29, 396.67) | 2.48 (0.58, 5.56) | 3.27 (0.82, 6.80) | 31.82 (4.51, 94.62) | 14.42 (2.86, 35.97) | 19.13 (4.23, 44.28) | 32.64 (5.33, 93.74) | 0.98 (0.92, 1.04)* |
| Ireland | 0.81 (0.15, 1.96) | 1.12 (0.24, 2.66) | 38.20 (6.98, 132.85) | 3.58 (0.71, 8.04) | 4.55 (1.07, 9.74) | 27.22 (-1.75, 113.46) | 21.60 (4.00, 53.27) | 29.16 (6.25, 68.96) | 34.99 (3.93, 130.12) | 1.01 (0.70, 1.33)* |
| Israel | 1.67 (0.36, 3.82) | 2.83 (0.62, 6.50) | 69.64 (40.15, 121.86) | 4.69 (1.12, 9.90) | 5.29 (1.30, 11.37) | 12.97 (-6.07, 45.66) | 31.85 (6.62, 74.08) | 33.20 (7.11, 76.21) | 4.22 (-12.92, 37.74) | 0.13 (0.09, 0.17)* |
| Italy | 11.05 (2.10, 26.78) | 7.30 (1.34, 18.10) | -34.00 (-42.25, -28.91) | 3.78 (0.83, 8.32) | 3.78 (0.82, 8.33) | -0.13 (-11.33, 7.98) | 20.82 (4.25, 49.56) | 19.59 (3.87, 47.23) | -5.90 (-16.58, 1.58) | -0.26 (-0.45, -0.06)* |
| Jamaica | 0.78 (0.17, 1.74) | 0.88 (0.19, 1.98) | 12.34 (-14.70, 50.09) | 6.22 (1.57, 13.07) | 6.45 (1.64, 13.11) | 3.69 (-17.24, 41.35) | 27.27 (5.79, 62.95) | 28.60 (6.42, 63.97) | 4.88 (-16.85, 40.44) | 0.17 (0.06, 0.28)* |
| Japan | 8.37 (1.58, 20.93) | 6.62 (1.16, 16.96) | -20.97 (-30.72, -13.81) | 2.48 (0.53, 5.65) | 2.72 (0.58, 6.21) | 9.65 (1.44, 20.48) | 7.32 (1.37, 18.22) | 8.66 (1.63, 21.58) | 18.25 (8.88, 30.33) | 0.58 (0.47, 0.69)* |
| Jordan | 1.48 (0.36, 3.40) | 5.24 (1.23, 11.77) | 254.13 (162.39, 412.54) | 4.19 (1.04, 8.95) | 5.98 (1.52, 12.29) | 42.83 (9.70, 111.02) | 30.16 (6.58, 71.94) | 38.21 (8.76, 86.24) | 26.70 (-2.97, 87.94) | 0.82 (0.69, 0.94)* |
| Kazakhstan | 1.39 (0.25, 3.62) | 1.36 (0.22, 3.66) | -2.04 (-18.20, 10.15) | 1.60 (0.33, 3.77) | 1.68 (0.35, 3.95) | 4.91 (-6.91, 17.40) | 7.82 (1.43, 20.26) | 8.05 (1.44, 21.09) | 2.89 (-9.31, 14.97) | 0.12 (0.05, 0.19)* |
| Kenya | 7.05 (1.62, 16.32) | 19.44 (4.25, 45.22) | 175.59 (149.22, 206.74) | 3.60 (0.84, 8.07) | 4.61 (1.06, 9.80) | 28.07 (16.94, 42.62) | 26.64 (5.33, 65.68) | 31.53 (6.31, 75.35) | 18.35 (7.97, 31.73) | 0.59 (0.56, 0.61)* |
| Kiribati | 0.01 (0.00, 0.03) | 0.02 (0.00, 0.05) | 51.00 (17.17, 105.99) | 4.48 (0.97, 9.97) | 4.84 (1.11, 10.24) | 8.15 (-15.29, 46.25) | 16.00 (3.07, 39.05) | 15.65 (3.19, 36.54) | -2.19 (-23.53, 34.68) | -0.07 (-0.10, -0.04)* |
| Kuwait | 0.35 (0.06, 0.87) | 0.87 (0.14, 2.22) | 148.40 (78.62, 257.00) | 2.79 (0.65, 6.17) | 3.41 (0.83, 7.12) | 22.13 (-6.62, 86.58) | 16.62 (3.29, 40.98) | 21.12 (4.60, 50.32) | 27.10 (-2.50, 93.73) | 0.82 (0.72, 0.93)* |
| Kyrgyzstan | 0.35 (0.06, 0.88) | 0.48 (0.08, 1.23) | 36.59 (15.27, 54.87) | 1.33 (0.28, 3.12) | 1.45 (0.29, 3.41) | 9.03 (-4.07, 22.71) | 6.93 (1.24, 17.70) | 6.79 (1.21, 17.36) | -2.08 (-14.33, 9.88) | -0.09 (-0.16, -0.02)* |
| Lao People's Democratic Republic | 0.52 (0.11, 1.25) | 1.14 (0.23, 2.67) | 117.75 (85.75, 158.75) | 3.50 (0.78, 7.76) | 4.76 (1.11, 10.26) | 36.07 (19.76, 63.01) | 11.27 (2.14, 27.03) | 13.50 (2.71, 31.47) | 19.82 (3.54, 45.89) | 0.62 (0.56, 0.69)* |
| Latvia | 0.64 (0.11, 1.59) | 0.38 (0.06, 0.90) | -40.44 (-56.27, -7.83) | 4.00 (0.85, 9.07) | 5.29 (1.21, 11.14) | 32.13 (0.49, 103.56) | 26.74 (5.03, 65.51) | 28.74 (5.87, 65.79) | 7.50 (-18.28, 68.82) | 0.26 (0.18, 0.34)* |
| Lebanon | 0.64 (0.14, 1.55) | 1.15 (0.22, 2.79) | 80.53 (18.39, 182.35) | 2.63 (0.63, 5.87) | 3.22 (0.78, 6.83) | 22.35 (-12.79, 93.97) | 18.15 (3.67, 44.96) | 23.66 (5.05, 55.85) | 30.34 (-7.12, 104.01) | 0.91 (0.83, 1.00)* |
| Lesotho | 0.82 (0.19, 1.89) | 1.08 (0.22, 2.58) | 31.16 (5.59, 51.52) | 4.63 (1.09, 10.09) | 4.91 (1.13, 10.81) | 6.13 (-6.06, 16.84) | 39.33 (8.07, 93.82) | 41.87 (8.52, 100.72) | 6.47 (-8.47, 20.67) | 0.20 (0.15, 0.26)* |
| Liberia | 0.30 (0.07, 0.74) | 1.34 (0.31, 3.25) | 340.06 (249.89, 486.91) | 2.34 (0.53, 5.54) | 3.53 (0.86, 8.02) | 50.70 (21.82, 100.43) | 15.44 (2.91, 38.83) | 23.30 (4.92, 58.55) | 50.83 (21.60, 100.95) | 1.52 (1.39, 1.65)* |
| Libya | 1.36 (0.32, 3.14) | 2.38 (0.49, 5.67) | 75.22 (25.62, 144.09) | 4.08 (0.99, 8.82) | 4.73 (1.22, 9.72) | 16.01 (-7.83, 64.18) | 26.70 (5.69, 63.77) | 32.48 (7.11, 75.64) | 21.61 (-4.25, 69.63) | 0.67 (0.61, 0.73)* |
| Lithuania | 1.80 (0.36, 4.07) | 0.94 (0.16, 2.13) | -47.80 (-60.32, -37.65) | 7.81 (1.84, 15.65) | 7.61 (1.72, 15.36) | -2.55 (-21.05, 16.34) | 50.58 (10.56, 112.17) | 45.34 (9.37, 100.77) | -10.35 (-28.05, 6.53) | -0.36 (-0.47, -0.25)* |
| Luxembourg | 0.10 (0.02, 0.25) | 0.13 (0.02, 0.30) | 24.64 (-10.29, 103.46) | 5.40 (1.19, 11.60) | 6.20 (1.59, 12.59) | 14.82 (-13.99, 80.20) | 30.53 (5.85, 71.92) | 27.04 (6.18, 60.13) | -11.44 (-35.77, 37.56) | -0.41 (-0.47, -0.36)* |
| Madagascar | 2.59 (0.52, 6.18) | 8.36 (1.80, 19.74) | 222.64 (175.23, 298.61) | 2.68 (0.58, 6.25) | 3.79 (0.88, 8.28) | 41.37 (21.69, 77.34) | 18.96 (3.59, 46.39) | 25.93 (5.30, 62.51) | 36.79 (16.70, 71.03) | 1.08 (1.04, 1.11)* |
| Malawi | 2.11 (0.45, 5.00) | 5.52 (1.33, 12.12) | 161.50 (105.04, 285.16) | 3.33 (0.73, 7.71) | 4.36 (1.07, 8.93) | 31.08 (3.78, 86.99) | 19.24 (3.76, 47.41) | 23.75 (5.11, 54.75) | 23.43 (-2.65, 79.13) | 0.72 (0.66, 0.79)* |
| Malaysia | 2.42 (0.51, 5.76) | 7.22 (1.74, 16.81) | 198.72 (115.47, 341.11) | 2.96 (0.68, 6.59) | 4.49 (1.24, 9.30) | 51.81 (11.52, 128.30) | 11.62 (2.40, 27.88) | 21.52 (5.35, 49.33) | 85.19 (34.94, 183.08) | 2.16 (2.03, 2.29)* |
| Maldives | 0.07 (0.02, 0.16) | 0.12 (0.02, 0.29) | 73.74 (10.27, 141.85) | 6.12 (1.44, 12.91) | 7.19 (1.89, 14.33) | 17.44 (-10.95, 69.89) | 27.92 (5.69, 63.82) | 23.76 (5.53, 53.00) | -14.90 (-35.34, 23.06) | -0.57 (-0.63, -0.50)* |
| Mali | 0.62 (0.13, 1.58) | 3.30 (0.74, 7.86) | 430.34 (336.67, 582.70) | 1.47 (0.33, 3.43) | 2.83 (0.67, 6.29) | 92.48 (59.44, 143.49) | 6.79 (1.31, 17.92) | 12.81 (2.55, 31.02) | 88.49 (53.73, 142.28) | 2.19 (2.09, 2.29)* |
| Malta | 0.04 (0.01, 0.10) | 0.04 (0.01, 0.10) | -3.73 (-27.48, 35.77) | 2.88 (0.68, 6.30) | 3.27 (0.77, 6.93) | 13.60 (-6.27, 56.57) | 12.37 (2.53, 29.30) | 13.70 (2.86, 32.36) | 10.82 (-10.44, 51.29) | 0.35 (0.29, 0.41)* |
| Marshall Islands | 0.01 (0.00, 0.02) | 0.01 (0.00, 0.03) | 13.17 (-6.51, 28.89) | 5.30 (1.25, 11.48) | 5.25 (1.14, 11.74) | -0.84 (-11.72, 9.05) | 17.51 (3.61, 41.95) | 16.35 (3.21, 40.08) | -6.59 (-18.33, 5.46) | -0.23 (-0.25, -0.21)* |
| Mauritania | 0.21 (0.04, 0.54) | 0.74 (0.17, 1.66) | 244.62 (146.02, 451.58) | 1.87 (0.40, 4.50) | 3.17 (0.78, 6.57) | 69.04 (21.78, 167.39) | 9.36 (1.72, 24.36) | 15.14 (3.30, 35.12) | 61.73 (14.98, 158.49) | 1.66 (1.60, 1.73)* |
| Mauritius | 0.34 (0.07, 0.83) | 0.35 (0.07, 0.80) | 2.09 (-21.39, 43.99) | 4.43 (1.10, 9.63) | 6.04 (1.59, 12.27) | 36.18 (6.76, 94.72) | 26.08 (5.35, 62.60) | 30.50 (6.98, 69.08) | 16.92 (-8.32, 66.01) | 0.54 (0.50, 0.57)* |
| Mexico | 11.80 (2.42, 28.28) | 24.05 (4.45, 59.18) | 103.76 (71.38, 130.77) | 2.54 (0.54, 5.76) | 3.34 (0.73, 7.63) | 31.86 (20.15, 48.21) | 11.20 (2.13, 27.77) | 18.26 (3.41, 45.00) | 63.09 (47.74, 83.72) | 1.72 (1.67, 1.77)* |
| Micronesia (Federated States of) | 0.02 (0.00, 0.04) | 0.02 (0.00, 0.05) | 2.31 (-12.99, 19.76) | 4.56 (1.07, 10.23) | 5.00 (1.14, 10.99) | 9.78 (-2.30, 24.38) | 15.52 (3.21, 38.43) | 15.79 (3.07, 37.79) | 1.69 (-11.95, 19.12) | 0.05 (0.01, 0.08)* |
| Monaco | 0.01 (0.00, 0.01) | 0.01 (0.00, 0.02) | 13.77 (3.24, 37.40) | 4.29 (0.92, 9.61) | 4.43 (0.92, 9.84) | 3.31 (-3.78, 10.67) | 26.42 (4.95, 66.53) | 27.12 (5.03, 66.32) | 2.64 (-6.41, 11.13) | 0.09 (0.07, 0.11)* |
| Mongolia | 0.55 (0.11, 1.32) | 0.74 (0.13, 1.78) | 35.34 (-4.54, 85.97) | 3.54 (0.76, 7.88) | 3.89 (0.89, 8.38) | 9.75 (-16.03, 57.84) | 21.07 (3.87, 52.09) | 23.03 (4.66, 53.43) | 9.34 (-17.58, 59.44) | 0.31 (0.27, 0.34)* |
| Montenegro | 0.07 (0.01, 0.17) | 0.05 (0.01, 0.14) | -23.74 (-33.35, -14.62) | 3.06 (0.63, 6.90) | 3.04 (0.64, 7.04) | -0.70 (-11.45, 10.47) | 11.02 (2.00, 26.77) | 10.62 (1.93, 26.68) | -3.66 (-14.82, 8.23) | -0.12 (-0.16, -0.09)* |
| Morocco | 3.68 (0.74, 9.16) | 7.06 (1.52, 17.03) | 92.20 (35.27, 213.21) | 1.43 (0.30, 3.33) | 2.26 (0.58, 4.91) | 58.90 (11.02, 159.59) | 12.14 (2.31, 31.07) | 18.77 (4.11, 44.99) | 54.60 (10.73, 149.63) | 1.51 (1.48, 1.55)* |
| Mozambique | 2.09 (0.43, 5.06) | 8.59 (1.86, 20.17) | 310.94 (253.28, 394.57) | 2.18 (0.48, 5.05) | 3.41 (0.80, 7.63) | 56.68 (34.46, 92.28) | 15.05 (2.71, 37.39) | 24.73 (4.83, 59.23) | 64.32 (40.53, 101.13) | 1.72 (1.68, 1.76)* |
| Myanmar | 1.06 (0.20, 2.56) | 1.73 (0.36, 4.19) | 62.96 (24.88, 131.84) | 1.31 (0.28, 3.05) | 1.83 (0.43, 3.94) | 39.85 (10.18, 97.37) | 2.15 (0.40, 5.23) | 2.96 (0.62, 7.19) | 37.78 (6.98, 98.96) | 1.08 (0.94, 1.23)* |
| Namibia | 0.45 (0.10, 1.03) | 0.89 (0.19, 1.95) | 97.51 (52.59, 165.58) | 5.63 (1.37, 11.94) | 6.81 (1.66, 14.00) | 20.93 (-2.61, 61.27) | 26.68 (5.39, 64.19) | 31.57 (6.59, 70.62) | 18.34 (-6.83, 60.98) | 0.57 (0.50, 0.64)* |
| Nauru | 0.00 (0.00, 0.01) | 0.00 (0.00, 0.01) | 29.50 (5.77, 82.09) | 5.88 (1.24, 12.80) | 6.75 (1.58, 14.21) | 14.85 (-4.20, 60.99) | 21.32 (3.99, 53.96) | 24.60 (5.13, 58.17) | 15.38 (-5.88, 63.39) | 0.49 (0.46, 0.52)* |
| Nepal | 6.75 (1.35, 15.61) | 14.29 (2.86, 32.52) | 111.89 (83.78, 145.89) | 4.58 (0.94, 9.97) | 5.22 (1.09, 10.99) | 13.94 (1.34, 32.13) | 32.26 (6.05, 77.44) | 39.50 (7.49, 91.42) | 22.44 (7.83, 43.41) | 0.70 (0.64, 0.76)* |
| Netherlands | 3.57 (0.62, 8.81) | 3.10 (0.57, 7.34) | -13.27 (-36.11, 17.87) | 4.58 (0.94, 10.31) | 4.73 (1.02, 10.10) | 3.31 (-23.59, 34.76) | 24.56 (4.78, 59.33) | 24.35 (4.78, 56.70) | -0.83 (-26.90, 31.56) | -0.03 (-0.08, 0.03) |
| New Zealand | 0.67 (0.12, 1.63) | 0.94 (0.19, 2.26) | 40.91 (25.09, 73.29) | 3.64 (0.76, 8.35) | 4.71 (1.12, 10.56) | 29.32 (16.41, 53.36) | 18.89 (3.41, 45.97) | 26.18 (5.41, 62.49) | 38.56 (22.83, 69.05) | 1.13 (1.09, 1.16)* |
| Nicaragua | 1.46 (0.35, 3.25) | 3.47 (0.83, 7.46) | 137.56 (90.73, 220.92) | 6.56 (1.66, 13.80) | 9.29 (2.47, 18.30) | 41.69 (22.06, 93.88) | 31.83 (7.04, 73.48) | 45.93 (10.91, 99.11) | 44.30 (23.60, 100.46) | 1.28 (1.23, 1.33)* |
| Niger | 0.42 (0.08, 1.07) | 3.09 (0.74, 7.15) | 641.24 (506.30, 863.09) | 0.83 (0.17, 2.03) | 1.88 (0.45, 4.18) | 127.34 (86.70, 197.52) | 4.81 (0.87, 13.01) | 11.26 (2.42, 27.08) | 134.31 (93.06, 203.11) | 2.99 (2.90, 3.07)* |
| Nigeria | 12.75 (2.63, 31.76) | 40.07 (9.08, 94.57) | 214.21 (166.74, 290.38) | 2.09 (0.44, 5.02) | 3.09 (0.71, 6.88) | 47.87 (26.80, 83.36) | 12.65 (2.36, 32.72) | 15.81 (3.19, 39.15) | 24.97 (6.44, 54.70) | 0.80 (0.70, 0.89)* |
| Niue | 0.00 (0.00, 0.00) | 0.00 (0.00, 0.00) | -28.58 (-40.42, -7.85) | 4.27 (0.83, 9.78) | 4.70 (1.00, 10.65) | 9.99 (-5.07, 40.53) | 15.64 (2.68, 39.82) | 17.09 (3.22, 42.47) | 9.31 (-7.23, 42.04) | 0.31 (0.29, 0.33)* |
| North Macedonia | 0.17 (0.03, 0.43) | 0.15 (0.02, 0.38) | -11.89 (-39.34, 41.71) | 2.52 (0.54, 5.74) | 2.88 (0.63, 6.41) | 14.38 (-16.62, 86.60) | 8.24 (1.53, 20.21) | 8.60 (1.63, 20.27) | 4.33 (-23.26, 69.72) | 0.14 (0.05, 0.22)* |
| Northern Mariana Islands | 0.01 (0.00, 0.02) | 0.01 (0.00, 0.02) | -19.32 (-29.45, -1.65) | 5.79 (1.41, 12.41) | 5.97 (1.42, 13.13) | 2.99 (-7.57, 13.10) | 15.95 (3.46, 38.49) | 17.46 (3.50, 41.00) | 9.49 (-3.93, 23.30) | 0.32 (0.20, 0.43)* |
| Norway | 0.64 (0.12, 1.58) | 0.84 (0.15, 2.12) | 33.01 (22.60, 41.08) | 4.20 (0.92, 9.28) | 4.45 (0.97, 9.81) | 6.11 (-1.00, 12.31) | 16.32 (3.23, 39.92) | 19.96 (3.80, 48.47) | 22.32 (13.15, 29.75) | 0.69 (0.48, 0.90)* |
| Oman | 0.34 (0.08, 0.85) | 1.73 (0.29, 4.24) | 408.55 (191.51, 698.07) | 2.62 (0.68, 5.98) | 5.54 (1.53, 11.22) | 111.40 (56.92, 225.98) | 15.77 (3.48, 39.52) | 33.70 (7.90, 75.00) | 113.65 (57.04, 230.36) | 2.65 (2.58, 2.72)* |
| Pakistan | 21.93 (4.26, 51.64) | 68.86 (15.71, 150.65) | 213.92 (158.11, 321.01) | 3.07 (0.59, 6.89) | 4.65 (1.04, 9.69) | 51.39 (25.54, 109.80) | 18.10 (3.04, 44.74) | 26.23 (5.34, 58.95) | 44.92 (20.14, 99.43) | 1.30 (1.16, 1.45)* |
| Palau | 0.00 (0.00, 0.01) | 0.00 (0.00, 0.01) | -18.43 (-28.54, -9.49) | 5.23 (1.14, 11.71) | 5.51 (1.19, 12.16) | 5.29 (-3.02, 14.92) | 19.05 (3.51, 46.76) | 19.79 (3.57, 48.98) | 3.88 (-6.43, 15.19) | 0.13 (0.09, 0.17)* |
| Palestine | 0.92 (0.21, 2.19) | 3.02 (0.67, 7.20) | 227.23 (177.38, 282.72) | 3.48 (0.86, 7.73) | 4.68 (1.17, 10.20) | 34.28 (18.18, 56.38) | 37.77 (8.09, 94.14) | 49.81 (10.87, 122.02) | 31.87 (14.37, 54.30) | 0.96 (0.86, 1.05)* |
| Panama | 0.41 (0.08, 0.99) | 0.68 (0.13, 1.71) | 67.39 (46.81, 88.23) | 3.25 (0.72, 7.39) | 3.74 (0.82, 8.50) | 15.11 (3.93, 28.64) | 14.14 (2.76, 35.22) | 16.00 (3.10, 40.44) | 13.10 (0.68, 28.18) | 0.43 (0.41, 0.45)* |
| Papua New Guinea | 0.73 (0.14, 1.77) | 1.87 (0.36, 4.50) | 155.90 (120.04, 200.36) | 4.05 (0.88, 9.32) | 4.70 (1.05, 10.53) | 16.17 (1.99, 35.56) | 15.13 (2.80, 37.72) | 16.51 (3.13, 40.18) | 9.11 (-5.67, 29.25) | 0.30 (0.27, 0.34)* |
| Paraguay | 0.84 (0.19, 1.97) | 1.80 (0.38, 4.35) | 115.16 (83.79, 145.72) | 3.36 (0.84, 7.38) | 4.01 (1.02, 8.77) | 19.14 (7.14, 32.74) | 17.87 (3.83, 42.79) | 23.06 (4.99, 55.39) | 29.02 (14.63, 46.15) | 0.88 (0.81, 0.95)* |
| Peru | 2.56 (0.54, 6.13) | 3.10 (0.58, 7.78) | 21.26 (1.17, 39.66) | 3.17 (0.76, 7.18) | 3.32 (0.72, 7.55) | 4.55 (-6.77, 15.79) | 9.77 (1.93, 23.94) | 8.82 (1.69, 21.95) | -9.76 (-20.97, 2.41) | -0.35 (-0.40, -0.31)* |
| Philippines | 11.78 (2.53, 27.83) | 20.01 (4.27, 47.64) | 69.83 (58.41, 86.55) | 4.34 (0.97, 9.60) | 5.41 (1.34, 11.61) | 24.45 (15.32, 41.86) | 15.53 (3.09, 37.64) | 15.87 (3.34, 38.09) | 2.19 (-5.50, 16.45) | 0.04 (-0.07, 0.16) |
| Poland | 2.11 (0.35, 5.53) | 2.13 (0.31, 5.80) | 0.90 (-16.47, 11.10) | 2.51 (0.50, 5.74) | 3.14 (0.62, 7.08) | 25.19 (14.93, 37.06) | 6.03 (1.03, 15.43) | 7.61 (1.33, 19.18) | 26.15 (15.72, 39.23) | 0.80 (0.71, 0.90)* |
| Portugal | 3.27 (0.69, 7.82) | 2.13 (0.41, 5.02) | -34.87 (-50.53, 5.62) | 4.17 (0.99, 9.18) | 4.44 (0.98, 9.32) | 6.59 (-16.53, 78.73) | 32.97 (6.93, 78.76) | 30.27 (6.28, 69.92) | -8.18 (-27.17, 53.52) | -0.30 (-0.42, -0.18)* |
| Puerto Rico | 0.59 (0.12, 1.45) | 0.42 (0.08, 1.02) | -29.09 (-39.11, -19.91) | 3.64 (0.87, 8.09) | 3.72 (0.90, 8.28) | 2.20 (-8.08, 13.43) | 15.50 (3.14, 38.45) | 15.04 (3.10, 36.50) | -2.92 (-14.26, 10.23) | -0.12 (-0.17, -0.07)* |
| Qatar | 0.20 (0.03, 0.51) | 1.55 (0.20, 3.95) | 670.36 (422.71, 942.19) | 6.03 (1.50, 12.73) | 7.54 (1.96, 15.09) | 24.99 (2.08, 82.60) | 39.95 (8.46, 93.54) | 46.18 (10.42, 106.58) | 15.59 (-6.00, 63.56) | 0.50 (0.42, 0.59)* |
| Republic of Korea | 5.33 (1.00, 13.03) | 4.61 (0.83, 11.63) | -13.52 (-25.72, -3.51) | 3.73 (0.87, 8.31) | 4.22 (1.02, 9.05) | 13.12 (2.80, 29.45) | 9.79 (1.92, 23.88) | 12.31 (2.63, 29.45) | 25.81 (11.00, 43.07) | 0.78 (0.68, 0.87)* |
| Republic of Moldova | 0.99 (0.18, 2.34) | 0.58 (0.09, 1.51) | -41.10 (-54.09, -31.08) | 4.33 (0.95, 9.44) | 4.57 (1.00, 10.11) | 5.37 (-9.86, 20.65) | 22.90 (4.45, 53.54) | 19.75 (3.85, 47.18) | -13.73 (-26.31, 0.11) | -0.51 (-0.55, -0.47)* |
| Romania | 3.17 (0.68, 7.59) | 2.22 (0.45, 5.14) | -30.01 (-50.41, 12.68) | 4.24 (0.97, 9.42) | 5.31 (1.23, 10.90) | 25.23 (-8.82, 101.01) | 13.91 (2.94, 33.38) | 16.79 (3.62, 37.48) | 20.68 (-12.16, 97.65) | 0.65 (0.56, 0.74)* |
| Russian Federation | 20.30 (3.11, 52.66) | 20.57 (3.03, 53.71) | 1.34 (-9.09, 11.51) | 3.31 (0.66, 7.53) | 4.67 (0.97, 10.42) | 41.25 (31.80, 56.08) | 14.43 (2.46, 36.25) | 19.01 (3.34, 47.06) | 31.71 (22.71, 45.74) | 0.96 (0.86, 1.05)* |
| Rwanda | 2.04 (0.42, 4.96) | 4.79 (0.99, 11.22) | 134.19 (103.41, 177.73) | 2.82 (0.61, 6.48) | 4.10 (0.93, 9.26) | 45.46 (25.74, 74.76) | 25.26 (4.82, 62.89) | 30.99 (6.10, 74.85) | 22.69 (6.32, 46.51) | 0.70 (0.63, 0.76)* |
| Saint Kitts and Nevis | 0.01 (0.00, 0.03) | 0.01 (0.00, 0.04) | 29.13 (0.14, 96.86) | 3.63 (0.78, 8.16) | 4.11 (0.95, 8.78) | 13.13 (-8.78, 73.79) | 22.84 (4.10, 56.73) | 25.61 (5.29, 61.49) | 12.16 (-10.48, 73.45) | 0.39 (0.35, 0.43)* |
| Saint Lucia | 0.03 (0.01, 0.07) | 0.04 (0.01, 0.09) | 19.17 (-9.50, 71.12) | 3.69 (0.89, 8.23) | 4.38 (1.07, 9.14) | 18.61 (-6.06, 75.77) | 17.98 (3.57, 44.30) | 21.68 (4.70, 50.96) | 20.59 (-3.99, 77.88) | 0.64 (0.57, 0.70)* |
| Saint Vincent and the Grenadines | 0.03 (0.01, 0.07) | 0.03 (0.01, 0.07) | -5.74 (-25.57, 27.72) | 4.53 (1.04, 10.01) | 5.16 (1.30, 10.57) | 13.79 (-6.71, 54.49) | 22.00 (4.53, 53.04) | 26.17 (5.72, 60.30) | 18.97 (-3.80, 63.22) | 0.59 (0.52, 0.66)* |
| Samoa | 0.04 (0.01, 0.09) | 0.04 (0.01, 0.10) | 12.11 (-0.78, 27.44) | 5.59 (1.27, 12.08) | 5.90 (1.40, 12.85) | 5.58 (-3.44, 18.48) | 18.58 (3.71, 44.43) | 17.42 (3.68, 41.48) | -6.24 (-16.17, 7.59) | -0.22 (-0.25, -0.20)* |
| San Marino | 0.01 (0.00, 0.02) | 0.01 (0.00, 0.02) | 10.32 (1.14, 19.54) | 4.40 (0.92, 9.86) | 4.41 (0.94, 9.91) | 0.20 (-6.90, 7.37) | 26.94 (5.07, 66.20) | 27.29 (5.24, 67.75) | 1.28 (-6.77, 10.31) | 0.04 (0.03, 0.06)* |
| Sao Tome and Principe | 0.03 (0.01, 0.06) | 0.06 (0.01, 0.14) | 122.80 (93.87, 157.98) | 4.02 (0.91, 9.09) | 4.93 (1.21, 10.66) | 22.86 (9.78, 44.67) | 19.56 (3.84, 48.23) | 24.23 (5.18, 57.65) | 23.90 (10.49, 47.44) | 0.74 (0.68, 0.79)* |
| Saudi Arabia | 3.64 (0.80, 9.02) | 12.19 (2.17, 31.43) | 235.23 (136.19, 310.59) | 3.13 (0.79, 6.92) | 4.84 (1.22, 10.37) | 54.73 (36.75, 79.23) | 18.79 (3.95, 46.88) | 30.95 (6.57, 74.30) | 64.74 (42.77, 92.32) | 1.74 (1.68, 1.79)* |
| Senegal | 0.92 (0.19, 2.28) | 3.03 (0.68, 7.12) | 230.18 (169.95, 314.00) | 1.97 (0.43, 4.59) | 2.98 (0.70, 6.68) | 51.75 (26.10, 93.44) | 10.75 (2.12, 27.30) | 16.52 (3.43, 40.02) | 53.73 (26.18, 94.53) | 1.49 (1.40, 1.58)* |
| Serbia | 1.06 (0.19, 2.63) | 0.68 (0.11, 1.75) | -35.44 (-44.13, -27.39) | 3.01 (0.62, 6.83) | 2.88 (0.58, 6.57) | -4.28 (-14.99, 9.02) | 12.29 (2.25, 30.17) | 10.04 (1.78, 25.04) | -18.28 (-28.77, -6.82) | -0.70 (-0.74, -0.67)* |
| Seychelles | 0.02 (0.00, 0.05) | 0.02 (0.01, 0.05) | 11.25 (-12.41, 54.08) | 7.91 (2.11, 15.97) | 9.48 (2.67, 18.24) | 19.81 (-2.27, 74.69) | 23.50 (5.35, 53.02) | 25.09 (6.33, 52.97) | 6.75 (-12.83, 52.56) | 0.22 (0.13, 0.32)* |
| Sierra Leone | 0.59 (0.12, 1.50) | 2.31 (0.50, 5.45) | 290.30 (230.29, 385.15) | 2.58 (0.53, 6.19) | 3.60 (0.83, 8.04) | 39.54 (18.28, 74.74) | 15.04 (2.81, 38.83) | 22.70 (4.62, 55.00) | 50.91 (27.66, 87.15) | 1.42 (1.37, 1.48)* |
| Singapore | 0.64 (0.11, 1.57) | 0.53 (0.08, 1.38) | -18.07 (-31.18, -7.26) | 3.78 (0.86, 8.47) | 4.54 (1.02, 9.70) | 20.15 (7.77, 37.26) | 17.64 (3.43, 42.69) | 13.61 (2.78, 32.21) | -22.83 (-32.67, -10.02) | -0.91 (-1.00, -0.82)* |
| Slovakia | 0.36 (0.06, 0.94) | 0.36 (0.07, 0.88) | -0.56 (-24.96, 92.98) | 1.90 (0.38, 4.49) | 2.86 (0.64, 6.31) | 51.06 (17.35, 211.53) | 7.03 (1.19, 17.73) | 9.29 (1.95, 21.87) | 32.18 (1.34, 175.25) | 0.96 (0.91, 1.02)* |
| Slovenia | 0.28 (0.05, 0.72) | 0.16 (0.03, 0.38) | -42.75 (-60.73, -6.29) | 2.66 (0.54, 6.16) | 2.91 (0.63, 6.29) | 9.22 (-23.13, 77.91) | 14.63 (2.62, 37.19) | 12.00 (2.15, 27.82) | -17.96 (-42.59, 33.53) | -0.69 (-0.76, -0.62)* |
| Solomon Islands | 0.03 (0.01, 0.07) | 0.06 (0.01, 0.15) | 102.65 (52.87, 194.80) | 2.09 (0.42, 4.89) | 2.41 (0.53, 5.33) | 15.72 (-11.27, 69.09) | 7.43 (1.32, 19.25) | 8.11 (1.44, 20.04) | 9.12 (-16.82, 59.22) | 0.30 (0.28, 0.32)* |
| Somalia | 0.71 (0.15, 1.73) | 4.18 (0.93, 9.96) | 489.75 (371.68, 662.44) | 1.31 (0.27, 3.09) | 2.46 (0.52, 5.86) | 87.27 (51.49, 142.95) | 9.28 (1.68, 23.74) | 17.28 (3.47, 42.11) | 86.10 (50.40, 139.55) | 2.16 (2.10, 2.22)* |
| South Africa | 11.27 (2.38, 26.53) | 16.84 (3.04, 41.93) | 49.44 (24.68, 65.58) | 4.13 (0.93, 9.21) | 4.56 (0.99, 10.14) | 10.45 (1.99, 19.34) | 25.10 (5.04, 60.11) | 27.76 (5.36, 68.81) | 10.61 (1.87, 19.88) | 0.36 (0.32, 0.41)* |
| South Sudan | 0.91 (0.18, 2.26) | 1.68 (0.37, 3.92) | 83.83 (46.62, 152.98) | 1.96 (0.40, 4.88) | 2.32 (0.51, 5.41) | 18.32 (-4.99, 57.96) | 12.83 (2.33, 33.05) | 15.66 (2.93, 38.66) | 22.00 (-3.10, 63.72) | 0.69 (0.65, 0.73)* |
| Spain | 8.46 (1.97, 20.09) | 7.47 (1.47, 17.84) | -11.70 (-37.88, 28.99) | 3.63 (0.93, 7.89) | 3.46 (0.80, 7.47) | -4.64 (-29.40, 39.37) | 22.06 (5.28, 52.38) | 25.80 (5.39, 59.90) | 16.96 (-15.12, 69.39) | 0.57 (0.47, 0.67)* |
| Sri Lanka | 3.61 (0.72, 8.49) | 3.39 (0.73, 7.91) | -6.05 (-26.76, 30.84) | 4.29 (0.97, 9.37) | 5.38 (1.34, 11.00) | 25.25 (-0.49, 77.26) | 17.85 (3.53, 42.32) | 16.47 (3.56, 38.46) | -7.70 (-27.62, 29.83) | -0.29 (-0.35, -0.22)* |
| Sudan | 5.27 (1.12, 12.69) | 13.02 (2.90, 31.36) | 147.16 (108.20, 193.59) | 3.20 (0.72, 7.19) | 3.74 (0.96, 8.18) | 16.71 (1.40, 39.49) | 22.72 (4.41, 56.76) | 25.88 (5.42, 64.49) | 13.90 (-4.26, 37.08) | 0.44 (0.40, 0.48)* |
| Suriname | 0.14 (0.03, 0.33) | 0.19 (0.04, 0.45) | 37.76 (7.67, 89.31) | 4.06 (0.93, 8.86) | 4.38 (1.07, 9.11) | 7.97 (-13.83, 48.02) | 30.04 (6.26, 71.83) | 34.33 (7.12, 80.50) | 14.28 (-9.12, 55.70) | 0.47 (0.43, 0.51)* |
| Sweden | 2.00 (0.35, 4.93) | 1.89 (0.33, 4.80) | -5.44 (-17.24, 8.01) | 4.41 (0.98, 9.71) | 4.03 (0.91, 8.91) | -8.49 (-18.22, 3.99) | 27.89 (5.28, 67.27) | 24.82 (4.78, 61.38) | -10.98 (-21.13, 1.53) | -0.41 (-0.49, -0.34)* |
| Switzerland | 1.89 (0.31, 4.76) | 1.73 (0.33, 4.07) | -8.75 (-28.50, 49.45) | 4.64 (1.03, 10.12) | 5.49 (1.40, 11.39) | 18.44 (-5.79, 90.92) | 30.98 (5.95, 73.27) | 28.56 (6.34, 64.16) | -7.82 (-27.57, 49.26) | -0.27 (-0.38, -0.15)* |
| Syrian Arab Republic | 3.61 (0.86, 8.31) | 4.45 (1.11, 10.38) | 23.05 (9.83, 41.44) | 3.72 (0.94, 8.03) | 4.19 (1.09, 9.14) | 12.68 (3.38, 25.63) | 23.33 (4.97, 55.74) | 26.14 (5.76, 62.71) | 12.06 (0.84, 25.81) | 0.40 (0.33, 0.47)* |
| Taiwan (Province of China) | 3.22 (0.65, 7.44) | 2.50 (0.45, 5.85) | -22.51 (-44.23, 2.37) | 6.13 (1.50, 12.92) | 5.80 (1.40, 11.91) | -5.34 (-26.41, 24.08) | 13.67 (2.88, 31.01) | 14.02 (2.99, 31.39) | 2.54 (-20.01, 35.51) | 0.09 (0.02, 0.16)* |
| Tajikistan | 0.15 (0.02, 0.37) | 0.29 (0.05, 0.74) | 99.24 (55.74, 187.99) | 0.60 (0.11, 1.47) | 0.76 (0.17, 1.78) | 27.61 (1.92, 85.52) | 2.34 (0.38, 6.11) | 2.68 (0.49, 6.81) | 14.61 (-8.24, 66.97) | 0.48 (0.37, 0.59)* |
| Thailand | 5.82 (1.23, 14.18) | 6.03 (1.16, 14.44) | 3.63 (-24.96, 52.82) | 2.71 (0.68, 6.01) | 3.52 (0.85, 7.39) | 29.65 (-1.28, 91.55) | 8.20 (1.74, 20.07) | 10.66 (2.26, 24.89) | 29.97 (-1.65, 92.46) | 0.90 (0.82, 0.97)* |
| Timor-Leste | 0.10 (0.02, 0.24) | 0.21 (0.05, 0.48) | 110.88 (74.76, 187.56) | 3.14 (0.67, 7.21) | 4.18 (1.02, 9.14) | 33.08 (13.51, 70.51) | 11.03 (2.07, 27.15) | 12.30 (2.68, 28.78) | 11.57 (-5.76, 45.42) | 0.36 (0.30, 0.43)* |
| Togo | 0.73 (0.15, 1.74) | 2.30 (0.50, 5.54) | 214.08 (172.41, 267.95) | 2.80 (0.61, 6.46) | 3.87 (0.89, 8.57) | 38.27 (20.90, 63.43) | 17.52 (3.42, 43.36) | 25.29 (5.20, 62.17) | 44.30 (25.46, 70.88) | 1.27 (1.24, 1.30)* |
| Tokelau | 0.00 (0.00, 0.00) | 0.00 (0.00, 0.00) | -16.24 (-26.07, -7.65) | 5.16 (1.12, 11.39) | 5.42 (1.20, 12.00) | 5.00 (-3.09, 13.29) | 19.04 (3.62, 47.31) | 19.66 (3.60, 48.75) | 3.23 (-6.57, 14.42) | 0.12 (0.10, 0.15)* |
| Tonga | 0.03 (0.01, 0.06) | 0.03 (0.01, 0.06) | 2.12 (-20.68, 45.20) | 9.11 (2.28, 18.48) | 9.82 (2.63, 19.13) | 7.81 (-14.69, 52.62) | 25.01 (5.33, 57.17) | 26.50 (6.12, 57.41) | 5.96 (-16.81, 53.38) | 0.20 (0.16, 0.23)* |
| Trinidad and Tobago | 0.25 (0.05, 0.65) | 0.23 (0.04, 0.56) | -10.29 (-33.88, 32.33) | 2.91 (0.64, 6.58) | 3.20 (0.76, 7.01) | 9.93 (-14.37, 61.56) | 18.73 (3.64, 47.68) | 19.32 (3.93, 45.82) | 3.16 (-21.27, 55.27) | 0.11 (0.06, 0.15)* |
| Tunisia | 2.62 (0.57, 6.27) | 3.52 (0.76, 8.36) | 34.78 (2.45, 90.19) | 3.14 (0.76, 6.92) | 4.07 (1.08, 8.44) | 29.90 (1.42, 85.69) | 25.94 (5.37, 63.64) | 33.55 (7.61, 77.54) | 29.36 (2.32, 83.82) | 0.88 (0.82, 0.94)* |
| Turkey | 23.59 (5.69, 51.96) | 40.24 (9.63, 89.78) | 70.56 (24.81, 172.06) | 5.39 (1.42, 11.40) | 8.34 (2.41, 16.53) | 54.89 (20.83, 150.11) | 32.92 (7.65, 74.49) | 49.14 (12.82, 107.90) | 49.27 (14.97, 136.11) | 1.33 (1.15, 1.51)* |
| Turkmenistan | 0.30 (0.06, 0.75) | 0.34 (0.06, 0.85) | 13.55 (-4.97, 27.60) | 1.55 (0.32, 3.61) | 1.63 (0.34, 3.78) | 5.38 (-7.22, 18.62) | 6.79 (1.28, 17.35) | 6.52 (1.18, 16.19) | -4.02 (-15.53, 8.90) | -0.14 (-0.18, -0.10)* |
| Tuvalu | 0.00 (0.00, 0.00) | 0.00 (0.00, 0.01) | 68.58 (34.44, 143.63) | 3.60 (0.73, 8.22) | 4.50 (0.97, 9.81) | 24.98 (0.94, 79.42) | 13.27 (2.35, 33.74) | 16.34 (3.18, 40.28) | 23.10 (-2.20, 76.49) | 0.72 (0.69, 0.74)* |
| Uganda | 5.10 (1.16, 12.06) | 20.78 (4.64, 46.50) | 307.40 (226.03, 446.57) | 2.32 (0.50, 5.27) | 3.92 (0.87, 8.23) | 68.66 (33.30, 133.85) | 25.53 (5.23, 61.97) | 42.01 (8.54, 97.24) | 64.51 (29.20, 128.06) | 1.70 (1.58, 1.82)* |
| Ukraine | 11.67 (1.88, 30.23) | 8.29 (1.12, 22.31) | -28.97 (-43.62, -18.70) | 3.81 (0.76, 8.74) | 4.36 (0.87, 9.86) | 14.42 (1.76, 27.89) | 24.81 (4.32, 62.22) | 25.18 (4.21, 62.83) | 1.53 (-9.53, 14.59) | 0.05 (0.01, 0.08)* |
| United Arab Emirates | 0.30 (0.05, 0.77) | 1.27 (0.19, 3.42) | 326.08 (160.07, 532.02) | 2.55 (0.61, 5.77) | 3.75 (0.96, 7.81) | 46.95 (7.76, 121.94) | 14.80 (3.09, 35.78) | 19.86 (4.51, 45.62) | 34.17 (-1.42, 104.20) | 1.03 (0.92, 1.14)* |
| United Kingdom | 14.93 (2.58, 36.97) | 13.43 (2.25, 33.34) | -10.00 (-18.00, -3.13) | 4.09 (0.88, 8.85) | 4.24 (0.92, 9.25) | 3.80 (-3.67, 11.44) | 29.12 (5.65, 70.35) | 26.01 (4.98, 63.34) | -10.69 (-17.67, -4.23) | -0.39 (-0.41, -0.36)* |
| United Republic of Tanzania | 2.98 (0.59, 7.25) | 9.59 (2.09, 22.05) | 221.65 (154.46, 370.59) | 1.43 (0.29, 3.33) | 2.18 (0.50, 4.70) | 52.64 (18.81, 124.89) | 9.88 (1.73, 24.89) | 14.46 (2.99, 34.06) | 46.34 (15.49, 116.93) | 1.32 (1.28, 1.35)* |
| United States Virgin Islands | 0.02 (0.00, 0.05) | 0.02 (0.00, 0.04) | -24.12 (-33.97, -15.92) | 4.06 (1.00, 8.83) | 4.16 (1.02, 9.05) | 2.47 (-6.45, 11.46) | 19.79 (4.12, 47.94) | 21.01 (4.37, 51.04) | 6.15 (-5.11, 17.52) | 0.21 (0.17, 0.24)* |
| United States of America | 65.41 (12.70, 157.57) | 152.78 (32.04, 354.10) | 133.58 (108.00, 171.60) | 5.45 (1.33, 11.77) | 8.53 (2.03, 17.64) | 56.59 (39.88, 78.69) | 27.20 (5.74, 62.89) | 56.67 (12.27, 129.15) | 108.31 (84.66, 140.65) | 2.56 (2.47, 2.66)* |
| Uruguay | 0.41 (0.08, 0.97) | 0.48 (0.09, 1.13) | 17.70 (-13.14, 67.35) | 3.48 (0.77, 7.57) | 3.54 (0.76, 7.74) | 1.74 (-22.95, 46.89) | 13.40 (2.55, 32.34) | 15.59 (2.94, 36.91) | 16.31 (-12.84, 64.01) | 0.52 (0.47, 0.57)* |
| Uzbekistan | 1.52 (0.28, 3.76) | 2.39 (0.43, 6.06) | 57.40 (33.06, 79.43) | 1.36 (0.28, 3.25) | 1.53 (0.33, 3.59) | 12.14 (-1.34, 26.88) | 6.20 (1.11, 15.70) | 6.46 (1.19, 16.32) | 4.24 (-9.37, 18.19) | 0.14 (0.10, 0.18)* |
| Vanuatu | 0.04 (0.01, 0.09) | 0.09 (0.02, 0.20) | 119.99 (82.36, 199.80) | 6.80 (1.59, 14.27) | 8.21 (2.10, 16.08) | 20.66 (0.50, 66.34) | 24.22 (4.65, 56.25) | 27.14 (6.19, 61.62) | 12.04 (-6.55, 55.00) | 0.39 (0.36, 0.42)* |
| Venezuela (Bolivarian Republic of) | 3.77 (0.80, 9.05) | 5.91 (1.16, 13.82) | 56.70 (23.76, 99.61) | 3.48 (0.83, 7.77) | 4.50 (1.02, 9.76) | 29.30 (5.77, 64.58) | 16.58 (3.39, 40.83) | 21.88 (4.33, 50.35) | 31.94 (7.98, 67.06) | 0.96 (0.92, 1.00)* |
| Viet Nam | 8.06 (1.61, 18.73) | 9.19 (1.74, 22.53) | 13.99 (-6.19, 30.79) | 3.86 (0.88, 8.41) | 4.44 (1.08, 9.80) | 14.98 (4.20, 31.43) | 9.77 (1.87, 22.93) | 9.98 (2.04, 23.60) | 2.15 (-9.72, 17.81) | 0.06 (-0.05, 0.16) |
| Yemen | 4.16 (0.92, 9.60) | 14.01 (3.29, 31.64) | 236.83 (170.43, 373.38) | 3.72 (0.89, 8.29) | 4.77 (1.25, 9.70) | 28.09 (4.33, 85.80) | 28.94 (5.72, 69.43) | 37.47 (8.40, 86.99) | 29.48 (4.33, 86.90) | 0.89 (0.87, 0.91)* |
| Zambia | 2.78 (0.65, 6.28) | 8.54 (1.98, 18.13) | 207.30 (152.95, 308.42) | 5.10 (1.26, 10.87) | 6.76 (1.68, 13.40) | 32.38 (9.73, 80.05) | 29.05 (6.04, 67.92) | 38.56 (8.23, 84.85) | 32.75 (9.17, 80.98) | 0.98 (0.95, 1.02)* |
| Zimbabwe | 2.34 (0.57, 5.16) | 3.99 (0.90, 8.80) | 70.80 (36.52, 125.00) | 4.72 (1.14, 10.22) | 5.44 (1.32, 11.08) | 15.12 (-6.45, 54.56) | 18.92 (4.06, 43.55) | 22.55 (4.74, 50.24) | 19.22 (-3.27, 60.30) | 0.60 (0.56, 0.64)* |
